# Association between *microRNA-146a, -499a* and *-196a-2* SNPs and non-small cell lung cancer: a case–control study involving 2249 subjects

**DOI:** 10.1042/BSR20201158

**Published:** 2021-02-17

**Authors:** Hao Qiu, Zhiqiang Xie, Weifeng Tang, Chao Liu, Yafeng Wang, Haiyong Gu, Qingfeng Zheng

**Affiliations:** 1 Department of Immunology, School of Medicine, Jiangsu University, Zhenjiang, Jiangsu Province, China; 2 Department of Clinical Laboratory, Fujian Medical University Union Hospital, Fuzhou, Fujian Province, China; 3 Department of Cardiothoracic Surgery, Affiliated People’s Hospital of Jiangsu University, Zhenjiang, Jiangsu Province, China; 4 Department of Cardiology, The People’s Hospital of Xishuangbanna Dai Autonomous Prefecture, Jinghong, Yunnan Province, China; 5 Department of Thoracic Surgery, Shanghai Chest Hospital, Shanghai Jiaotong University, Shanghai, China; 6 Department of Thoracic Surgery, Fujian Medical University Cancer Hospital, Fujian Cancer Hospital, Fuzhou, Fujian Province, China

**Keywords:** microRNA, non-small cell lung cancer, polymorphism, susceptibility

## Abstract

*MicroRNA* (*miR*) acts as a negative regulator of gene expression. Many literatures have suggested that *miRs* may be involved in the process of cell proliferation, inflammation, oxidative stress, energy metabolism and epithelial–mesenchymal transition. Thus, *miRs* may be implicated in the occurrence of non-small cell lung cancer (NSCLC). In the current investigation, we included 2249 subjects (1193 NSCLC patients and 1056 controls) and designed a study to identify the relationship of *miR-146a* rs2910164 C/G, *-499a* rs3746444 A/G and *-196a-2* rs11614913 T/C with the risk of NSCLC. The risk factors (e.g., body mass index (BMI), sex, smoking, drinking and age) was used to adjust the odds ratios (ORs) and 95% confidence intervals (CIs). After conducting a power value assessment, we did not confirm that the *miR-*single nucleotide polymorphisms (SNPs) genotypic distributions were different in NSCLC cases and controls. However, the association of *miR-196a-2* rs11614913 with a decreased risk of NSCLC was identified in the female subgroup (adjusted *P*=0.005, power = 0.809 for TC vs. TT, and adjusted *P*=0.004, power = 0.849 for CC/TC vs. TT). In addition, gene–gene interaction analysis showed that rs11614913 TC/3746444 AA and rs11614913 CC/rs3746444 AA could also reduce the susceptibility to NSCLC (rs11614913 TC/rs3746444 AA vs. rs11614913 TT/rs3746444 AA, *P*=0.001, power = 0.912 and rs11614913 CC/rs3746444 AA vs. rs11614913 TT/rs3746444 AA, *P*=0.003, power = 0.836). In conclusion, in overall comparisons, we did not confirm that the rs2910164, rs3746444, and rs11614913 SNPs genotypic distributions were different in NSCLC cases and controls. However, this case–control study demonstrates that *miR-196a-2* rs11614913 may be a protective factor for the development of NSCLC among female patients.

## Introduction

Lung cancer (LC) caused ∼11.6% of all new cancer cases and 18.4% of all cancer-related deaths worldwide [1]. In China, 733.3 thousand new LC patients and 610.2 thousand LC-related deaths were assessed to occur in 2015 [2]. The etiology of LC was unclear. It is reported that a number of genetic and environmental risk factors may cause the development of LC [3–5]. Non-small cell lung cancer (NSCLC) is the most common type of LC. The individual’s hereditary factor may be implicated in the occurrence of NSCLC.

*MicroRNA* (*miR*), a small non-coding RNA, acts as a negative regulator of gene expression. In the nucleus, the Drosha/DiGeorge syndrome critical region 8 complex cleaves pri-miRNAs [6]. Then, in the cytoplasm, Dicer crops these formed pre-miRNAs [7]. Finally, they are incorporated into the Argonaute-containing RNA-induced silencing complexes [8]. Mature *miR* is composed of ∼22 nucleic acids, which is generated from primary *miRs* and further changed to mature *miRs* in cytoplasm. The target mRNAs located in 3′-untranslated regions (3′-UTRs). Matured *miRs* can recognize the 3′-UTRs of mRNA and bind to them, and then result in a weakened expression of target genes. The mechanism of the process is hybridization of seed sequences of matured *miRs* with 3′-UTRs. An individual *miR* can bind to masses of targets, and regulate a number of pathways. Many investigations have suggested that *miRs* may be involved in the process of cell proliferation, inflammation, oxidative stress, energy metabolism and epithelial–mesenchymal transition (EMT) [9–16]. Of late, some previous investigations have indicated that *miRs* have been implicated in the occurrence of NSCLC [17,18]. There are single nucleotide polymorphisms (SNPs) in certain *miRs*. These SNPs might influence the generation process of *miRs* or alter target recognition/hybridization. Thus, *miR* polymorphisms may be implicated in the occurrence and/or progress of cancer [19–25].

Park *et al.* reported that *miR-146a* could restrain EMT progression in NSCLC by repressing the expression of insulin receptor substrate-2 [14]. It was found that *miR-146a* inhibited migratory capacity, downstream signaling of epidermal growth factor receptor and NSCLC cell growth; however, it could promote the apoptosis process of NSCLC cell lines [13]. Xiong *et al.* reported that *miR-146a* rs2910164 C>G locus could affect its maturation in peripheral blood mononuclear cells [26]. A recent study reported that G allele of rs2910164 mgiht increase *miR-146a* level [27]. A previous study suggested that rs2910164 locus might influence the toxicity in LC chemotherapy [28]. Several reports indicated that rs2910164 polymorphism in *miR-146a* could decrease the risk to LC [29,30]. However, other case–control studies suggested that rs2910164 might not influence the occurrence of LC [31,32]. These controversial observations may be due to the limited sample sizes. Here, we explored the role of *miR-146a* rs2910164 SNP with the development of NSCLC and a potential interaction of this SNP with risk factors to identify whether this locus could be used as a biomarker for susceptibility to NSCLC in Chinese populations.

Rs11614913 T>C was widely explored in malignancy as a candidate locus of *miR-196a-2* [33,34]. Hu *et al.* reported that that the rs11614913 T→C variant in *miR-196a-2* could affect the binding ability of mature *hsa-mir-196a-2-3p* binding with its target mRNA [35]. Recently, this polymorphism was thought to alter LC cases’ sensitivity to platinum-based chemotherapy [23]. A functional study highlighted that rs11614913 might be involved in the development of LC through altering the secondary structure and the expression of *miR-196a-2* [36]. Thus, rs11614913 polymorphism might be implicated in carcinogenesis of LC and could affect an individual’s susceptibility of LC. Indeed, several case–control studies have investigated the role of rs11614913 in the occurrence of LC [23,36]. However, the observations were conflicting, even in the same ethnicity. For example, some recent studies indicated a significant relationship between *miR-196a-2* rs11614913 and the development of LC [36–38], whereas others did not confirm the potential correlation [23,32].

A previous investigation reported that *miR-499a* rs3746444 SNP could affect the process of *miR-499-5p* maturation and the role of antiapoptosis [39]. The relationship between *miR-499a* rs3746444 A>G and the susceptibility and progress of LC has been explored. Ge *et al.* reported that *miR-499a* rs3746444 AA genotype could inhibit the expression of *miR-499a* gene and CD200 [40]. And then this SNP could influence the survival of NSCLC cases. Several studies have focused on the role of *miR-499a* rs3746444 in the development of LC [40,41]. However, recent meta-analyses have reported contradictory findings [42–44]. Thus, the correlation of *miR-499a* rs3746444 with the development of LC was more inconsistent.

In the current investigation, we designed a larger sample size study to identify the correlation of rs3746444, rs2910164 and rs11614913 with the occurrence of NSCLC.

## Materials and methods

### Study population and ethical approval

Each participant donated a peripheral blood sample. NSCLC cases in the current investigation were recruited from the Zhenjiang Medical College of Nanjing Medical University (Jiangsu Province, China) and the Union Medical College of Fujian Medical University (Fujian Province, China) between January 2014 and June 2018. All NSCLC cases were diagnosed via histopathological examination. In the present study, the selection criteria were defined as the following: (1) Chinese Han populations, (2) sporadic cases and (3) without any history of other cancer. And the exclusion criteria were summarized as: (1) a patient who had an autoimmune disease, (2) NSCLC patients who underwent chemoradiotherapy and/or targeted therapy, (3) NSCLC recurrent cases and (4) heterochronous NSCLC. In total, 1193 NSCLC cases were enrolled. At the same time, 1056 participants without a history of cancer were included as controls in the Medical Colleges mentioned above. The data of demographics and potential risk factors were collected by a pre-structured questionnaire. During the recruitment, each participant signed a written informed consent. The present study was approved by the Ethics Review Committee of Fujian Union Hospital (2018KY023).

### Isolation of DNA and genotyping

Using DNA Isolation Kit (Promega, Madison, U.S.A.), we extracted genomic DNA. The obtained DNA was kept at −80°C. The quality of DNA sample was assessed by Nanodrop ND-1000 UV. A custom-SNPscan™ Kit (Genesky Biotechnologies Inc., Shanghai, China) was used to analyze the genotypes. Briefly, no less than 120 ng DNA sample was used to conduct a double ligation and multiplex fluorescence polymerase chain reaction (PCR). ABI-3730XL sequencer (PE Applied Biosystems, Foster City, CA, U.S.A.) was used to detect the PCR products. The obtained raw data were analyzed by harnessing GeneMapper 4.1 (Applied Biosystems, U.S.A.). To conduct a quality control, 90 samples were randomly chosen and repeated genotyped in the same PCR method. The results indicated that 100% concordant results were observed.

### Statistical analysis

Hardy–Weinberg equilibrium (HWE) (https://ihg.gsf.de/cgi-bin/hw/hwa1.pl) [45] and SAS 9.4 (SAS Institute, Cary, North Carolina) software were harnessed to analyze HWE and genetic data. Odds ratios (ORs) and 95% confidence intervals (CIs) were used to estimate the relationship of rs2910164, rs11614913 and rs3746444 with the risk of NSCLC. We also calculated adjusted ORs and 95% CIs using logistic regression analyses. In the current study, five risk factors [e.g., body mass index (BMI), smoking, drinking, age and gender] were included. Power Calculator (http://biostat.mc.vanderbilt.edu/twiki/bin/view/Main/PowerSampleSize) was used to calculate the power of sample size [19,46]. We also used the false-positive report probability (FPRP) to evaluate the findings [47].

## Results

### Characteristics of the study population

In the current study, 1193 cases with NSCLC (mean ± SD age, 58.92 ± 10.44 years) and 1056 controls (mean ± SD age, 59.36 ± 9.19 years) were collected (Table 1). In NSCLC group, 642 males and 551 females were included. While in controls, there were 586 males and 470 females. The age and gender were well-mathed (*P* = 0.960 and 0.425, respectively). The distribution of smoking, drinking and BMI were different between two groups (all *P*<0.001). Raw data of genotypes and characteristics were summarized in Supplementary Table S1.

**Table 1:** Distribution of selected demographic variables and risk factors in NSCLC cases and controls

Variable	NSCLC cases (*n*=1193)		Controls (*n*=1056)		*P*^a^
	*n*	%	*n*	%	
Age (years)	58.92 ± 10.44		59.36 ± 9.19		0.293
Age (years)					0.330
<59	535	44.84	452	42.80	
≥59	658	55.16	604	57.20	
Sex					0.425
Male	642	53.81	586	55.65	
Female	551	46.19	470	44.35	
Smoking status					**<0.001**
Never	757	63.45	857	81.16	
Ever	436	36.55	199	18.84	
Alcohol use					**<0.001**
Never	946	79.30	967	91.83	
Ever	247	20.70	89	8.17	
BMI (kg/m^2^)					**<0.001**
<24	801	67.14	571	54.07	
≥24	392	32.86	485	45.93	
Type of NSCLC					
SCC	182	15.26			
Non-SCC	1,011	84.74			
Stage					
I	703	58.93			
II	87	7.29			
III	222	18.61			
IV	181	15.17			
Lymph node status					
Positive	381	31.94			
Negative	812	68.06			

Bold values are statistically significant (*P*<0.05). Abbreviation: SCC, squamous cell carcinoma.

^a^Two-sided *χ*^2^ test and Student’s *t* test.

### Information of rs3746444, rs2910164 and rs11614913 SNPs

The successful ratio of genotyping was more than 99.00%. Table 2 has summarized some vital information for rs2910164, rs11614913 and rs3746444. In controls, these included *miR*-SNPs genotype distributions met HWE (*P*>0.05). Supplementary Table S1 summarized the detailed information and genotypes for each individual.

**Table 2:** Primary information for *miR-146a* rs2910164 C>G, *miR-196a-2* rs11614913 T>C and *miR-499a* rs3746444 A>G polymorphisms

Genotyped SNPs	*miR-146a* rs2910164 C>G	*miR-196a-2* rs11614913 T>C	*miR-499a* rs3746444 A>G
Chromosome	5	12	20
Function	nc-transcript-variant	nc-transcript-variant	nc-transcript-variant
Chr Pos (NCBI Build 38)	160485411	53991815	3499048
MAF^1^ for Chinese in database	0.35	0.34	0.15
MAF in our controls (*n*=1056)	0.36	0.46	0.15
*P*-value for HWE^2^ test in our controls	0.217	0.208	0.898
Genotyping method	SNPscan	SNPscan	SNPscan
% Genotyping value	99.47%	99.47%	99.29%

1 MAF, minor allele frequency.

2 HWE, Hardy–Weinberg equilibrium.

### Rs3746444, rs2910164 and rs11614913 SNPs and NSCLC susceptibility

The number of *miR-146a* rs2910164 allele and genotype in NSCLC cases and controls is summarized in Table 3. In this case–control study, for overall comparisons, we identified that the *miR-146a* genotype frequency was not significantly different among the two groups. As well, we also found that the *miR-499a* rs3746444 genotypic distribution was not different in NSCLC cases and controls.

**Table 3:** The frequencies of *miR-146a* rs2910164 C>G, *miR-196a-2* rs11614913 T>C and *miR-499a* rs3746444 A>G polymorphisms in CAD patients and controls

Genotype	Overall NSCLC cases (*n*=1193)		SCC cases (*n*=182)		Non-SCC cases (*n*=1011)		Controls (*n*=1056)	
	*n*	%	*n*	%	*n*	%	*n*	%
*miR-146*a rs2910164 C>G								
CC	460	38.85	68	37.57	392	39.08	440	41.79
CG	555	46.88	91	50.28	464	46.26	467	44.35
GG	169	14.27	22	12.15	147	14.66	146	13.87
G allele	893	37.71	135	37.29	758	37.79	759	36.04
*miR-499a* rs3746444 A>G								
AA	814	68.98	128	71.11	686	68.60	757	71.89
AG	330	27.97	47	26.11	283	28.30	271	25.74
GG	36	3.05	5	2.78	31	3.10	25	2.37
G allele	402	17.03	57	15.83	345	17.25	321	15.24
m*iR-196a-2* rs11614913 T>C								
TT	392	33.11	59	32.60	333	33.20	293	27.83
TC	572	48.31	90	49.72	482	48.06	544	51.66
CC	220	18.58	32	17.68	188	18.74	216	20.51
C allele	1,012	42.74	154	42.54	858	42.77	976	46.34

Abbreviation: SCC, squamous cell carcinoma.

Table 3 lists the *miR-196a-2* rs11614913 genotype distribution in NSCLC cases and controls. It was notable that there was statistical significance in comparison of rs11614913 genotypes in three genetic models among NSCLC cases and controls. The decreased genotype frequencies of rs11614913 TC, CC and TC/CC were found in NSCLC patients. In relation to rs11614913 TT, individuals carrying rs11614913 TC genotypes had a decreased 21% susceptibility to the cocurrence of NSCLC (*P*=0.014, Table 4). Additionally, compared with rs11614913 TT, rs11614913 CC and TC/CC genotypes were also protective factors for the co-ocurrence of NSCLC (CC vs. TT: *P*=0.027 and TC/CC vs. TT: *P*=0.007, Table 4). When we adjusted for risk factors, the decreased susceptibility for the occurrence of NSCLC was not changed (Table 4).

**Table 4:** Overall and stratified analyses of *miR-146a* rs2910164 C>G, *miR-196a-2* rs11614913 T>C and *miR-499a* rs3746444 A>G polymorphisms with NSCLC

Genotype	Overall NSCLC cases (*n*=1193) vs. Controls (1056)				Non-SCC cases cases (*n*=1011) vs. Controls (1056)				SCC cases cases (*n*=182) vs. Controls (1056)			
	Crude OR (95% CI)	*P*	Adjusted OR^1^ (95% CI)	*P*	Crude OR (95% CI)	*P*	Adjusted OR^1^ (95% CI)	*P*	Crude OR (95% CI)	*P*	Adjusted OR^1^ (95% CI)	*P*
*miR-146a* rs2910164 C>G												
CG vs. CC	1.14 (0.95–1.36)	0.162	1.11 (0.92–1.34)	0.268	1.12 (0.93–1.35)	0.254	1.07 (0.88–1.30)	0.498	1.26 (0.90–1.77)	0.182	1.22 (0.82–1.81)	0.323
GG vs. CC	1.11 (0.86–1.43)	0.437	1.17 (0.90–1.54)	0.243	1.13 (0.87–1.48)	0.368	1.15 (0.87–1.51)	0.329	0.98 (0.58–1.63)	0.924	1.24 (0.68–2.27)	0.477
GG/CG vs. CC	1.13 (0.95–1.34)	0.158	1.13 (0.94–1.34)	0.188	1.12 (0.94–1.33)	0.212	1.09 (0.91–1.31)	0.367	1.19 (0.86–1.65)	0.287	1.23 (0.84–1.79)	0.291
GG vs. CC/CG	1.03 (0.82–1.31)	0.782	1.11 (0.87–1.42)	0.415	1.07 (0.83–1.37)	0.608	1.11 (0.86–1.43)	0.436	0.86 (0.53–1.39)	0.536	1.12 (0.64–1.96)	0.700
*miR-499a* rs3746444 A>G												
AG vs. AA	1.13 (0.94–1.37)	0.196	1.14 (0.93–1.39)	0.201	1.15 (0.95–1.40)	0.156	1.16 (0.94–1.42)	0.164	1.03 (0.71–1.47)	0.891	0.92 (0.61–1.41)	0.707
GG vs. AA	1.34 (0.80–2.25)	0.271	1.63 (0.94–2.81)	0.081	1.37 (0.80–2.34)	0.253	1.64 (0.94–2.88)	0.083	1.18 (0.45–3.15)	0.737	1.18 (0.37–3.70)	0.780
GG/AG vs. AA	1.15 (0.96–1.38)	0.133	1.18 (0.97–1.42)	0.098	1.17 (0.97–1.42)	0.103	1.19 (0.98–1.45)	0.080	1.04 (0.73–1.47)	0.829	0.94 (0.63–1.42)	0.778
GG vs. AA/AG	1.29 (0.77–2.17)	0.329	1.57 (0.91–2.71)	0.104	1.32 (0.77–2.24)	0.315	1.58 (0.90–2.76)	0.109	1.18 (0.44–3.11)	0.746	1.20 (0.38–3.76)	0.752
*miR-196a-2* rs11614913 T>C												
TC vs. TT	**0.79 (0.65–0.95)**	**0.014**	**0.79 (0.65–0.97)**	**0.024**	**0.78 (0.64–0.95)**	**0.014**	**0.79 (0.64–0.97)**	**0.026**	0.82 (0.58–1.18)	0.282	0.82 (0.54–1.24)	0.336
CC vs. TT	**0.76 (0.60–0.97)**	**0.027**	**0.77 (0.60–0.99)**	**0.042**	**0.77 (0.60–0.98)**	**0.037**	0.77 (0.60–1.00)	0.052	0.74 (0.46–1.17)	0.196	0.83 (0.48–1.42)	0.490
CC/ TC vs. TT	**0.78 (0.65–0.93)**	**0.007**	**0.79 (0.65–0.95)**	**0.014**	**0.78 (0.64–0.94)**	**0.008**	**0.79 (0.65–0.96)**	**0.015**	0.80 (0.57–1.12)	0.190	0.82 (0.55–1.21)	0.319
CC vs. TT/TC	0.88 (0.72–1.09)	0.249	0.89 (0.71–1.11)	0.286	0.89 (0.72–1.11)	0.314	0.90 (0.71–1.12)	0.333	0.83 (0.55–1.25)	0.380	0.94 (0.59–1.51)	0.795

Bold values are statistically significant (*P*<0.05). Abbreviation: SCC, squamous cell carcinoma.

1 Adjusted for age, sex, smoking, drinking and BMI.

### MiR-SNPs and NSCLC susceptibility in different types of pathology

Supplementary Tables S2 and S3 summarized the detailed information and genotypes for squamous cell carcinoma (SCC) and non-SCC cases, respectively. When we conducted a subgroup analysis by type of pathology, for rs11614913 SNP, the decreased susceptibility for the occurrence of NSCLC was also found in non-SCC subgroup (TC vs. TT: adjusted *P*=0.026 and TC/CC vs. TT: adjusted *P*=0.015, Table 4). For rs2910164 and rs3746444 polymorphisms, no significant association between these SNPs and NSCLC risk was found (Table 4).

### Stratification analysis of miR-SNPs and NSCLC susceptibility

#### MiR-146a rs2910164 C>G locus

When we conducted stratification analyses by risk factors, an increased risk for the occurrence of NSCLC was identified in never drinking subgroup (CG vs. CC: adjusted *P*=0.043 and GG/CG vs. CC: adjusted *P*=0.028, Table 5).

**Table 5:** Stratified analyses between *miR-146a* rs2910164 C>G polymorphism and NSCLC risk by age, sex, smoking, drinking and BMI

Variable	*miRNA-146a* rs2910164 C>G (case/control)^1^			Adjusted OR^2^ (95% CI); *P*			
	CC	CG	GG	CG vs. CC	GG vs. CC	GG/CG vs. CC	GG vs. CC/CG
Sex							
Male	260/249	289/255	89/80	1.06 (0.81–1.37); *P*: 0.685	1.19 (0.82–1.73); *P*: 0.361	1.09 (0.85–-1.39); *P*: 0.508	1.16 (0.82–1.64); *P*: 0.411
Female	200/191	266/212	80/66	1.15 (0.88–1.52); *P*: 0.309	1.21 (0.82–1.78); *P*: 0.347	1.17 (0.90–1.51); *P*: 0.247	1.12 (0.78–1.60); *P*: 0.550
Age							
<59	203/192	258/198	69/60	1.16 (0.87–1.54); *P*: 0.313	1.17 (0.76–1.78); *P*: 0.478	1.16 (0.89–1.52); *P*: 0.282	1.08 (0.73–1.60); *P*: 0.709
≥59	257/248	297/269	100/86	1.06 (0.83–1.37); *P*: 0.627	1.22 (0.86–1.73); *P*: 0.272	1.10 (0.87–1.39); *P*: 0.426	1.18 (0.85–1.63); *P*: 0.323
Smoking status							
Never	280/358	360/371	111/125	1.22 (0.98–1.52); *P*: 0.080	1.15 (0.85–1.57); *P*: 0.274	1.20 (0.98–1.48); *P*: 0.084	1.04 (0.78–1.38); *P*: 0.809
Ever	180/82	195/96	58/21	0.88 (0.61–1.27); *P*: 0.507	1.32 (0.74–2.33); *P*: 0.352	0.96 (0.68–1.36); *P*: 0.814	1.40 (0.82–2.40); *P*: 0.221
Alcohol consumption							
Never	354/410	447/420	139/135	**1.23 (1.01–1.21); *P*: 0.043**	1.26 (0.94–1.67); *P*: 0.120	**1.24 (1.02–1.50); *P*: 0.028**	1.12 (0.86–1.47); *P*: 0.390
Ever	106/30	108/47	30/11	0.59 (0.34–1.02); *P*: 0.061	0.77 (0.34–1.73); *P*: 0.527	0.63 (0.37–1.06); *P*: 0.079	1.02 (0.48–2.16); *P*: 0.956
BMI (kg/m^2^)							
<24	303/236	381/260	110/73	1.12 (0.88–1.42); *P*: 0.373	1.27 (0.89–1.80); *P*: 0.191	1.15 (0.91–1.44); *P*: 0.236	1.19 (0.86–1.66); *P*: 0.292
≥24	157/204	174/207	59/73	1.11 (0.82–1.50); *P*: 0.493	1.06 (0.70–1.61); *P*: 0.790	1.10 (0.83–1.46); *P*: 0.518	1.00 (0.68–1.48); *P*: 0.988

1 For *miRNA-146a* rs2910164 C>G, the genotyping was successful in 1184 (99.25%) NSCLC cases and 1053 (99.72%) controls.

2 Adjusted for age, sex, smoking, drinking and BMI (besides stratified factors accordingly) in a multiple logistic regression model.

#### MiR-499a rs3746444 A>G locus

Table 6 listed the findings of stratification analyses for rs3746444 polymorphism. We identified that rs3746444 polymorphism elevated the susceptibility of NSCLC (never smoking subgroup: adjusted *P*=0.035 for GG vs. AA genetic model and adjusted *P*=0.049 for GG vs. AA/AG genetic model; never drinking subgroup: adjusted *P*=0.032 for GG vs. AA genetic model, adjusted *P*=0.035 for GG/AG vs. AA genetic model and adjusted *P*=0.047 for GG vs. AA/AG genetic model; BMI < 24 (kg/m^2^) subgroup: adjusted *P*=0.042 for AG vs. AA genetic model and adjusted *P*=0.034 for GG vs. AA/AG genetic model and never BMI ≥ 24 (kg/m^2^) subgroup: adjusted *P*=0.046 for GG vs. AA/AG genetic model).

**Table 6:** Stratified analyses between *miR-499a* rs3746444 A>G polymorphism and NSCLC risk by age, sex, smoking, drinking and BMI

Variable	*miRNA-499a* rs3746444 A>G (case/control)^1^			Adjusted OR^2^ (95% CI); *P*			
	AA	AG	GG	AG vs. AA	GG vs. AA	GG/AG vs. AA	GG vs. AA/AG
Sex							
Male	444/415	172/152	20/17	1.05 (0.79–1.38); *P*: 0.744	1.59 (0.79–3.21); *P*: 0.199	1.09 (0.84–1.43); *P*: 0.509	1.57 (0.78–3.16); *P*: 0.209
Female	370/342	158/119	16/8	1.21 (0.91–1.61); *P*: 0.194	1.84 (0.77–4.41); *P*: 0.172	1.25 (0.95–1.65); *P*: 0.118	1.74 (0.73–4.17); *P*: 0.211
Age							
<59	367/338	144/101	15/11	1.30 (0.95–1.78); *P*: 0.096	1.68 (0.72–3.92); *P*: 0.233	1.33 (0.99–1.80); *P*: 0.060	1.57 (0.67–3.65); *P*: 0.297
≥59	447/419	186/170	21/14	1.03 (0.79-1.33); *P*: 0.854	1.71 (0.84-3.51); *P*: 0.141	1.07 (0.84–1.38); *P*: 0.583	1.70 (0.83–3.47); *P*: 0.144
Smoking status							
Never	511/618	209/215	28/21	1.17 (0.93–1.48); *P*: 0.176	**1.91 (1.08–3.48); *P*: 0.035**	1.23 (0.99–1.54); *P*: 0.066	**1.82 (1.00–3.32); *P*: 0.049**
Ever	303/139	121/56	8/4	1.04 (0.71–1.52); *P*: 0.856	0.90 (0.26–3.13); *P*: 0.873	1.03 (0.71–1.49); *P*: 0.889	0.90 (0.26–3.09); *P*: 0.861
Alcohol consumption							
Never	629/695	274/247	33/23	1.19 (0.97–1.47); *P*: 0.101	**1.86 (1.06–3.29); *P*: 0.032**	**1.25 (1.02–1.53); *P*: 0.035**	**1.77 (1.01–3.12); *P*: 0.047**
Ever	185/62	56/24	3/2	0.75 (0.42–1.32); *P*: 0.314	0.43 (0.07–2.65); *P*: 0.360	0.72 (0.41–1.25); *P*: 0.245	0.46 (0.07–2.82); *P*: 0.398
BMI (kg/m2)							
<24	535/413	230/139	25/17	**1.30 (1.01–1.68); *P*: 0.042**	1.31 (0.68–2.52); *P*: 0.419	**1.30 (1.02–1.67); *P*: 0.034**	1.22 (0.64–2.33); *P*: 0.555
≥24	279/344	100/132	11/8	0.90 (0.65–1.23); *P*: 0.495	2.54 (0.98–6.55); *P*: 0.054	0.97 (0.71–1.32); *P*: 0.854	**2.61 (1.02–6.73); *P*: 0.046**

1 For *miR-499a* rs3746444 A>G, the genotyping was successful in 1180 (98.91%) NSCLC cases and 1053 (99.72%) controls.

2 Adjusted for age, sex, smoking, drinking and BMI (besides stratified factors accordingly) in a multiple logistic regression model.

#### MiR-196a-2 rs11614913 T>C locus

For *miR-196a-2* rs11614913, significant difference in frequency of its genotype was found between NSCLC cases and controls. We identified that rs11614913 polymorphism may be a protective factor for the occurrence of NSCLC (female subgroup: adjusted *P*=0.005 for TC vs. TT genetic model, adjusted *P*=0.038 for CC vs. TT genetic model and adjusted *P*=0.004 for CC/TC vs. TT genetic model; never smoking subgroup: adjusted *P*=0.038 for CC vs. TT genetic model and adjusted *P*=0.049 for CC/TC vs. TT genetic model; never drinking subgroup: adjusted *P*=0.024 for TC vs. TT genetic model, adjusted *P*=0.018 for CC vs. TT genetic model and adjusted *P*=0.009 for CC/TC vs. TT genetic model, Table 7).

**Table 7:** Stratified analyses between *miR-196a-2* rs11614913 T>C polymorphism and NSCLC risk by age, sex, smoking, drinking and BMI

Variable	*miR-196a-2* rs11614913 T>C (case/control)^1^			Adjusted OR^2^ (95% CI); *P*			
	TT	TC	CC	TC vs. TT	CC vs. TT	CC/TC vs. TT	CC vs. TT/TC
Sex							
Male	204/176	315/287	119/121	0.96 (0.73–1.26); *P*: 0.761	0.87 (0.61–1.23); *P*: 0.428	0.93 (0.72–1.21); *P*: 0.594	0.89 (0.66–1.21); *P*: 0.461
Female	188/117	257/257	101/95	**0.66 (0.49–0.88); *P*: 0.005**	**0.68 (0.47–0.98); *P*: 0.038**	**0.66 (0.50–0.87); *P*: 0.004**	0.88 (0.64–1.22); *P*: 0.445
Age							
<59	184/141	246/218	100/91	0.81 (0.60–1.09); *P*: 0.165	0.81 (0.56–1.19); *P*: 0.279	0.81 (0.61–1.07); *P*: 0.142	0.92 (0.66–1.29); *P*: 0.625
≥59	208/152	326/326	120/125	0.79 (0.60–1.03); *P*: 0.083	0.74 (0.53–1.04); *P*: 0.081	0.77 (0.60–1.00); *P*: 0.050	0.86 (0.64–1.15); *P*: 0.317
Smoking status							
Never	246/237	365/436	140/181	0.83 (0.66–1.05); *P*: 0.121	**0.73 (0.55–0.98); *P*: 0.038**	**0.80 (0.64–1.00); *P*: 0.049**	0.82 (0.64–1.06); *P*: 0.131
Ever	146/56	207/108	80/35	0.73 (0.49–1.08); *P*: 0.116	0.88 (0.53–1.47); *P*: 0.624	0.77 (0.53**–**1.11); *P*: 0.163	1.07 (0.69–1.67); *P*: 0.765
Alcohol consumption							
Never	312/264	456/501	172/200	**0.78 (0.63–0.97); *P*: 0.024**	**0.72 (0.55–0.95); *P*: 0.018**	**0.76 (0.62–0.94); *P*: 0.009**	0.84 (0.67**–**1.07); *P*: 0.151
Ever	80/29	116/43	48/16	0.97 (0.55–1.70); *P*: 0.908	1.19 (0.58**–**2.45); *P*: 0.640	1.03 (0.61**–**1.74); *P*: 0.923	1.21 (0.64**–**2.30); *P*: 0.558
BMI (kg/m^2^)							
<24	258/165	382/282	154/122	0.83 (0.64–1.08); *P*: 0.167	0.82 (0.59**–**1.12); *P*: 0.207	0.83 (0.65**–**1.06); *P*: 0.128	0.91 (0.69**–**1.20); *P*: 0.505
≥24	134/128	190/262	66/94	0.75 (0.55**–**1.03); *P*: 0.079	0.70 (0.47**–**1.07); *P*: 0.097	0.74 (0.55**–**1.00); *P*: 0.051	0.84 (0.59**–**1.21); *P*: 0.358

1 For *miR-196a-2* rs11614913 T>C, the genotyping was successful in 1184 (99.25%) NSCLC cases and 1053 (99.72%) controls.

2 Adjusted for age, sex, smoking, drinking and BMI (besides stratified factors accordingly) in a multiple logistic regression model.

### Gene–gene interaction analysis

We also conducted *miR*-SNPs combined analysis for three included SNPs. Three potential types (rs11614913/rs2910164, rs11614913/rs3746444, rs2910164/rs3746444 and rs11614913/rs2910164/rs3746444) were combined to explore the gene–gene interaction and their roles on the occurrence of NSCLC.

In analysis of rs11614913/rs2910164 loci combination, we used rs11614913 TT/rs2910164 CC as reference. It was notable that the rs11614913 CC/rs2910164 CC combination was a protective factor for the development of NSCLC (*P*=0.010, Table 8). In another analysis of rs11614913/rs3746444 loci combination, compared with rs11614913 TT/rs3746444 AA, frequency of rs11614913 TC/rs3746444 AA was lower in NSCLC patients 32.54% (384/1080) than in controls 37.70% (397/1053). When rs11614913 TT/rs3746444 AA was used as a reference, frequency of rs11614913 CC/rs3746444 AA was also lower in NSCLC patients 12.46% (147/1080) than in controls 15.19% (160/1053). When rs11614913 TT/rs2910164 CC/rs3746444 AA was used as a reference, TC/CC/AA, TC/GG/AA and CC/CC/AA genotype combinations might decrease the risk of NSCLC (Table 8).

**Table 8:** Combination analysis of miR polymorphisms (rs2910164, rs11614913 and rs3746444) in NSCLC patients and controls

Genotype	Case		Control		OR (95% CI)	*P*-value
	*n*	%	*n*	%		
rs11614913/rs2910164						
TT/CC	159	13.43	122	11.59	1.00	
TT/CG	177	14.95	133	12.63	1.02 (0.74–1.41)	0.900
TT/GG	56	4.73	38	3.61	1.13 (0.70–1.82)	0.612
TC/CC	227	19.17	224	21.27	0.78 (0.58–1.02)	0.110
TC/CG	268	22.64	239	22.70	0.86 (0.64–1.15)	0.315
TC/GG	77	6.50	81	7.69	0.73 (0.49–1.08)	0.113
CC/CC	74	6.25	94	8.93	**0.60 (0.41–0.89)**	**0.010**
CC/CG	110	9.29	95	9.02	0.89 (0.62–1.28)	0.522
CC/GG	36	3.04	27	2.56	1.02(0.59–1.78)	0.936
rs11614913/rs3746444						
TT/AA	283	23.98	200	18.99	1.00	
TT/AG	97	8.22	86	8.17	0.80 (0.57–1.12)	0.194
TT/GG	11	0.93	7	0.66	1.11 (0.42–2.91)	0.831
TC/AA	384	32.54	397	37.70	**0.68 (0.54–0.86)**	**0.001**
TC/AG	166	14.07	137	13.01	0.86 (0.64–1.14)	0.294
TC/GG	19	1.61	10	0.95	1.34 (0.61–2.95)	0.462
CC/AA	147	12.46	160	15.19	**0.65 (0.49–0.87)**	**0.003**
CC/AG	67	5.68	48	4.56	0.99 (0.65–1.49)	0.948
CC/GG	6	0.51	8	0.76	0.53(0.18–1.55)	0.239
rs2910164/rs3746444						
CC/AA	322	27.29	324	30.77	1.00	
CC/AG	124	10.51	108	10.26	1.16 (0.86–1.56)	0.346
CC/GG	13	1.10	8	0.76	1.64 (0.67–4.00)	0.277
CG/AA	374	31.69	320	30.89	1.18 (0.95–1.46)	0.139
CG/AG	161	13.64	135	12.82	1.20 (0.91–1.58)	0.195
CG/GG	18	1.53	12	1.14	1.51 (0.72–3.18)	0.277
GG/AA	118	10.00	113	10.73	1.05 (0.78–1.42)	0.747
GG/AG	45	3.81	28	2.66	1.62 (0.98–2.66)	0.056
GG/GG	5	0.42	5	0.47	1.01 (0.29–3.51)	0.992
rs11614913/rs2910164/rs3746444						
TT/CC/AA	114	9.66	86	8.17	1.00	
TT/CC/AG	41	3.47	35	3.32	0.88 (0.52–1.50)	0.648
TT/CC/GG	4	0.34	1	0.09	3.02 (0.33–27.55)	0.304
TT/CG/AA	128	10.85	89	8.45	1.08 (0.74–1.60)	0.681
TT/CG/AG	44	3.73	40	3.80	0.83 (0.50–1.38)	0.475
TT/CG/GG	5	0.42	4	0.38	0.94 (0.25–3.62)	0.932
TT/GG/AA	41	3.47	25	2.37	1.24 (0.70–2.19)	0.464
TT/GG/AG	12	1.02	11	1.04	0.82 (0.35–1.95)	0.658
TT/GG/GG	2	0.17	2	0.19	0.75 (0.10–5.47)	0.780
TC/CC/AA	155	13.14	167	15.86	**0.70 (0.49–1.00)**	**0.049**
TC/CC/AG	64	5.42	54	5.13	0.89 (0.57–1.41)	0.632
TC/CC/GG	7	0.59	3	0.28	1.76 (0.44–7.01)	0.417
TC/CG/AA	174	14.75	163	15.48	0.81 (0.57–1.15)	0.228
TC/CG/AG	83	7.03	71	6.74	0.88 (0.58–1.35)	0.560
TC/CG/GG	9	0.76	5	0.47	1.36 (0.44–4.20)	0.594
TC/GG/AA	55	4.66	67	6.36	**0.62 (0.39–0.97)**	**0.038**
TC/GG/AG	19	1.61	12	1.14	1.19 (0.55–2.59)	0.653
TC/GG/GG	3	0.25	2	0.19	1.13 (0.18–6.92)	0.894
CC/CC/AA	53	4.49	71	6.74	**0.56 (0.36–0.89)**	**0.013**
CC/CC/AG	19	1.61	19	1.80	0.75 (0.38–1.51)	0.426
CC/CC/GG	2	0.17	4	0.38	0.38 (0.07–2.11)	0.250
CC/CG/AA	72	6.10	68	6.46	0.80 (0.52–1.23)	0.310
CC/CG/AG	34	2.88	24	2.28	1.07 (0.59–1.93)	0.826
CC/CG/GG	4	0.34	3	0.28	1.01 (0.22–4.61)	0.994
CC/GG/AA	22	1.86	21	1.99	0.79 (0.41–1.53)	0.484
CC/GG/AG	14	1.19	5	0.47	2.26 (0.79–6.47)	0.119
CC/GG/GG	0	0.0	1	0.09	0.25 (0.01–6.26)	0.251

Values in bold are statistically significant (*P*<0.05).

### Study power (α = 0.05) and FPRP mothed

For overall comparisons, these *miR-*SNPs did not confer a risk to NSCLC. Each power value for overall positive report was less than 0.8 (data not shown). For the comparison of *miR*-SNPs and NSCLC susceptibility in different types of pathology, we also could not confirm the positive report (data not shown). In stratification analysis of *miR*-SNPs with NSCLC susceptibility, we only confirmed that rs11614913 polymorphism could be a protective factor for the occurrence of NSCLC in the female subgroup (the power values were 0.809 in TC vs. TT and 0.848 in CC/TC vs. TT). In these *miR-*SNPs combination analysis, compared with rs11614913 TT/3746444 AA, rs11614913 TC/3746444 AA and rs11614913 CC/rs3746444 AA could decrease the susceptibility of NSCLC (power value: 0.912 and 0.836, respectively). Other power values less than 0.8 were not shown. After assessing power value and FPRP, we highlighted that *miR-196a-2* rs11614913 decreased the risk to NSCLC in the female subgroup. As well, gene–gene interaction analysis showed that rs11614913 TC/3746444 AA and rs11614913 CC/rs3746444 AA could also reduce the susceptibility to NSCLC.

## Discussion

LC is a common malignancy with 18.4% of overall cancer-related deaths worldwide [1]. The etiology of LC is not well-known. NSCLC is the most common subtype of LC. *MiR* is a negative regulator of gene expression. It may involve in the development of cancer. Some investigations have focused on the role of *miRs* on the occurrence and survival of NSCLC [40,48,49]. The individual’s hereditary factor may be implicated in the occurrence of NSCLC. In this investigation, we designed a study to identify the correlation of *miR-*SNPs (rs3746444, rs2910164 and rs11614913) with the risk of NSCLC in Chinese populations. We highlighted that rs11614913, in the female subgroup, could decrease the risk to NSCLC. As well, gene–gene interaction analysis showed that rs11614913 TC/3746444 AA and rs11614913 CC/rs3746444 AA could also reduce the susceptibility to NSCLC.

Rs11614913 locates on the 3p strand region of mature *miR-196a-2* [50]*.* Thus, this locus could participate in the process of *pre-miR* maturation and affect the combination of *miR-196a-2* with target genes [51]. Hu *et al.* reported that that the T>C variant in rs11614913 locus could alter the ability of mature *hsa-mir-196a-2-3p* binding to its target mRNA [35]. Therefore, this SNP could be used as an important biomarker for NSCLC prognosis [35]. A previous study has suggested that annexin A1 (ANXA1), a regulator of inflammation, could be regulated by *miR-196a-2* [52]. A bioinformatics analysis suggested that the expression of ANXA1 could influence the survival of NSCLC cases [53]. Additionally, knockdown of ANXA1 could inhibit the invasion, migration and proliferation of NSCLC cells. Thus, *miR-196a-2* could be implicated in the occurrence of cancer. Fang *et al.* reported that variants of rs11614913 could alter the response of LC case to platinum-based chemotherapy [23]. Toraih *et al.* found that individuals carrying the rs11614913 C allele might be a protective factor of LC, which was associated with *miR-196a-2* low-expression in tissue [54]. A recent investigation indicated that the polymorphism of rs11614913, through influencing the level of *miR-196a-2* and secondary structure, conferred risk to LC in females [36]. In the current invstigation, we found that the *miR-196a-2* rs11614913 could reduce the susceptibility to NSCLC in female. In view of these investigations mentioned above, we might conclude that rs11614913 C allele could be a protective factor to the occurrence of NSCLC though altering the level of *miR-196a-2* and secondary structure. It is well known that smoking is a major risk for LC. However, in the present study, we did not find the interaction of tobacco using and rs11614913 SNP with the development of NSCLC. In the future, these conclusions should be confirmed by further studies.

Several literatures have focused on the relationship between gene–gene interaction and the occurrence of human diseases [55–57]. In this study, we analyzed the combined effect of these *miR*-SNPs. Gene–gene interaction analyses showed that rs11614913 TC/3746444 AA and rs11614913 CC/rs3746444 AA could also decrease the susceptibility of NSCLC, which suggested that rs11614913 C allele could inhibit the development of NSCLC. We first confirmed that rs11614913 TC/3746444 AA and rs11614913 CC/rs3746444 AA combinations could decrease the risk of NSCLC. However, this combination did not influence the risk of cervical cancer [56]. Therefore, the effect of rs11614913 TC/3746444 AA combination could be different in different cancer. In the future, the possible correlation is needed to verify in other studies.

Several limitations, in this investigation, should be pointed out. Firstly, some vital data were unknown; thus, a more extensively stratified analysis for other risk factors (e.g., vegetable and fruit intake, air pollution, lifestyle and occupational exposure) could not be done. Second, due to the hospital-based study, bias might have happened in our analysis. Third, the number of participants in the present study was moderate. Last, we only included three *miR-*SNPs in the present study, and other important *miR-*SNPs should not be ignored.

In conclusion, the present study highlights that *miR-196a-2* rs11614913 decreases the risk to NSCLC among female subgroup. Additionally, combined gene–gene analyses suggest that rs11614913 TC/3746444 AA and rs11614913 CC/rs3746444 AA are protective factors for the development of NSCLC. More investigations are needed to validate the potential effect of these *miR-*SNPs in NSCLC. And more functional studies should also be done.

## Supplementary Material

Supplementary Tables S1-S3

## Data Availability

Full data are available via an online supplementary material. Raw data of genotypes and characteristics were summarized in Supplementary Table S1. Supplementary Tables S2 and S3 summarized the detailed information and genotypes for SCC and non-SCC cases, respectively.

## Abbreviations

ANXA1: annexin A1
BMI: body mass index
CI: confidence interval
EMT: epithelial–mesenchymal transition
FPRP: false-positive report probability
HWE: Hardy–Weinberg equilibrium
LC: lung cancer
*miR*: *microRNA*
NSCLC: non-small cell lung cancer
OR: odds ratio
PCR: polymerase chain reaction
SCC: squamous cell carcinoma
SNP: single nucleotide polymorphism
3′-UTR: 3′-untranslated region
