# Supplementary material for: Association between microRNA-146a, -499a and -196a-2 SNPs and non-small cell lung cancer: a case–control study involving 2249 subjects
Source: Biosci Rep. 2021 Feb 17;41(2):BSR20201158. doi: 10.1042/BSR20201158 (PMC7890400; doi:10.1042/BSR20201158)
Supplement: Supplementary Tables S1-S3 [file BSR-2020-1158_supp.pdf]

Table S1 Raw data and characteristics of study

Subjects	Sex (male: 1; female: 2)	Age (year)	Smoking(Yes: 1, No: 0)	Drinking (Yes: 1, No: 0)	BMI (≥ 24: 1, <24: 0)	rs11614913	rs2910164	rs3746444
NSCLC case-0001	1	67	1	0	0	C/C	G/C	G/A
NSCLC case-0002	2	82	0	0	0	T/T	C/C	A/A
NSCLC case-0003	2	47	0	0	0	T/T	G/C	G/G
NSCLC case-0004	1	60	0	0	0	T/T	C/C	A/A
NSCLC case-0005	1	61	0	0	0	C/T	C/C	A/A
NSCLC case-0006	2	63	0	0	0	C/T	G/G	A/A
NSCLC case-0007	2	52	0	0	0	T/T	G/C	G/A
NSCLC case-0008	2	57	0	0	1	T/T	G/C	A/A
NSCLC case-0009	1	71	1	0	0	C/C	G/G	A/A
NSCLC case-0010	2	45	0	0	0	C/C	G/C	A/A
NSCLC case-0011	1	75	1	0	1	C/T	C/C	A/A
NSCLC case-0012	2	53	0	0	1	C/T	G/C	A/A
NSCLC case-0013	2	46	0	0	0	T/T	C/C	A/A
NSCLC case-0014	1	48	1	0	0	C/C	C/C	A/A
NSCLC case-0015	1	53	0	0	0	C/T	G/G	A/A
NSCLC case-0016	2	56	0	0	1	T/T	C/C	A/A
NSCLC case-0017	2	43	0	0	0	T/T	C/C	G/A
NSCLC case-0018	2	53	0	0	0	T/T	C/C	A/A
NSCLC case-0019	2	62	0	0	1	C/T	G/C	G/A
NSCLC case-0020	1	67	1	0	0	C/T	C/C	A/A
NSCLC case-0021	1	53	0	0	1	C/T	G/C	A/A
NSCLC case-0022	2	47	0	0	0	C/T	G/C	A/A
NSCLC case-0023	2	49	0	0	1	C/T	C/C	A/A
NSCLC case-0024	2	64	0	0	1	C/C	G/C	G/A
NSCLC case-0025	2	57	0	0	0	T/T	C/C	A/A
NSCLC case-0026	2	62	0	0	0	C/T	C/C	A/A
NSCLC case-0027	2	53	0	0	0	T/T	G/C	A/A
NSCLC case-0028	2	64	0	0	0	T/T	G/G	A/A
NSCLC case-0029	2	59	0	0	0	C/T	C/C	A/A
NSCLC case-0030	2	52	0	0	1	C/T	C/C	A/A
NSCLC case-0031	2	55	0	0	1	T/T	C/C	A/A
NSCLC case-0032	2	63	0	0	0	C/C	C/C	A/A
NSCLC case-0033	1	42	1	0	0	C/T	G/C	A/A
NSCLC case-0034	1	56	1	1	1	T/T	G/C	A/A
NSCLC case-0035	1	61	1	0	0	T/T	C/C	G/A
NSCLC case-0036	2	39	0	0	0	C/C	G/C	A/A
NSCLC case-0037	1	65	1	0	1	C/C	G/G	G/A
NSCLC case-0038	1	48	1	1	0	C/T	G/C	A/A
NSCLC case-0039	2	54	0	0	0	C/T	C/C	G/A
NSCLC case-0040	1	59	1	0	1	T/T	C/C	G/A
NSCLC case-0041	1	52	1	0	0	T/T	C/C	A/A
NSCLC case-0042	2	61	0	0	0	C/T	C/C	A/A
NSCLC case-0043	2	59	0	0	1	C/C	C/C	A/A
NSCLC case-0044	2	64	0	0	0	C/T	C/C	A/A
NSCLC case-0045	1	51	1	0	0	C/C	C/C	G/A
NSCLC case-0046	1	73	0	0	1	C/T	G/G	G/A
NSCLC case-0047	1	76	0	0	0	C/T	G/C	A/A
NSCLC case-0048	2	70	0	0	0	C/T	C/C	G/A

NSCLC	case-0049	1	67	0	0	0	T/T	G/C	A/A
NSCLC	case-0050	2	65	0	0	0	C/T	G/G	G/A
NSCLC	case-0051	1	44	1	1	0	C/T	C/C	A/A
NSCLC	case-0052	2	72	0	0	1	T/T	C/C	A/A
NSCLC	case-0053	1	65	1	0	0	C/T	G/C	A/A
NSCLC	case-0054	2	48	0	0	1	T/T	G/C	A/A
NSCLC	case-0055	2	65	0	0	0	C/C	G/C	A/A
NSCLC	case-0056	2	53	0	0	0	C/T	C/C	G/A
NSCLC	case-0057	1	68	1	0	0	C/T	C/C	G/A
NSCLC	case-0058	1	53	1	0	0	T/T	G/C	G/A
NSCLC	case-0059	1	53	1	0	0	C/C	G/G	G/A
NSCLC	case-0060	1	59	1	0	0	C/T	G/G	G/A
NSCLC	case-0061	1	59	1	0	1	C/T	C/C	G/A
NSCLC	case-0062	2	59	0	0	1	C/T	G/G	A/A
NSCLC	case-0063	1	77	0	0	0	C/T	G/C	G/A
NSCLC	case-0064	1	78	1	0	1	C/T	G/C	A/A
NSCLC	case-0065	2	43	0	0	0	C/T	G/C	A/A
NSCLC	case-0066	1	71	1	1	0	C/T	C/C	A/A
NSCLC	case-0067	1	61	0	0	0	C/T	G/G	A/A
NSCLC	case-0068	1	53	0	0	0	C/C	G/C	G/A
NSCLC	case-0069	2	74	0	0	1	T/T	G/C	A/A
NSCLC	case-0070	2	73	0	0	0	C/T	C/C	G/A
NSCLC	case-0071	1	59	1	1	0	C/T	G/C	A/A
NSCLC	case-0072	2	61	0	0	0	T/T	G/C	A/A
NSCLC	case-0073	1	60	1	0	0	C/T	C/C	A/A
NSCLC	case-0074	1	70	1	1	1	T/T	C/C	A/A
NSCLC	case-0075	1	80	0	0	1	C/T	G/C	G/A
NSCLC	case-0076	1	62	1	1	0	C/C	C/C	A/A
NSCLC	case-0077	2	64	0	0	1	C/T	C/C	A/A
NSCLC	case-0078	2	45	0	0	1	C/T	G/C	A/A
NSCLC	case-0079	2	67	0	0	0	T/T	C/C	G/A
NSCLC	case-0080	2	53	0	0	0	T/T	G/C	G/A
NSCLC	case-0081	2	64	1	1	0	T/T	C/C	A/A
NSCLC	case-0082	1	71	1	0	0	C/T	G/C	A/A
NSCLC	case-0083	2	57	0	0	0	C/T	C/C	A/A
NSCLC	case-0084	2	62	0	0	0	C/C	G/C	A/A
NSCLC	case-0085	2	66	0	0	1	C/C	C/C	A/A
NSCLC	case-0086	1	49	0	0	1	T/T	C/C	A/A
NSCLC	case-0087	1	70	1	0	0	C/T	C/C	A/A
NSCLC	case-0088	1	71	1	0	0	T/T	G/C	A/A
NSCLC	case-0089	1	53	1	1	0	C/T	G/C	A/A
NSCLC	case-0090	1	47	1	0	0	T/T	G/C	A/A
NSCLC	case-0091	1	64	1	0	0	C/C	G/C	G/A
NSCLC	case-0092	2	55	0	0	1	T/T	C/C	G/A
NSCLC	case-0093	2	63	0	0	0	T/T	G/G	A/A
NSCLC	case-0094	1	50	1	1	0	C/T	C/C	A/A
NSCLC	case-0095	2	64	0	0	0	C/T	G/C	A/A
NSCLC	case-0096	1	70	1	0	1	T/T	G/C	G/A
NSCLC	case-0097	1	64	0	0	0	C/T	C/C	G/A
NSCLC	case-0098	1	62	1	0	1	T/T	G/C	A/A
NSCLC	case-0099	2	72	0	0	0	C/T	G/C	G/G
NSCLC	case-0100	1	59	0	0	1	T/T	G/C	A/A
NSCLC	case-0101	1	73	1	0	0	C/T	C/C	A/A
NSCLC	case-0102	2	68	0	0	1	C/T	G/C	A/A

NSCLC	case-0103	1	44	1	0	0	C/T	C/C	A/A
NSCLC	case-0104	2	48	0	0	1	C/T	G/C	A/A
NSCLC	case-0105	1	87	0	0	1	C/T	G/G	A/A
NSCLC	case-0106	2	60	0	0	1	T/T	G/G	G/A
NSCLC	case-0107	2	58	0	0	0	C/T	G/C	A/A
NSCLC	case-0108	2	61	0	0	0	T/T	C/C	A/A
NSCLC	case-0109	2	57	0	0	0	T/T	G/C	A/A
NSCLC	case-0110	1	58	1	0	1	T/T	G/C	A/A
NSCLC	case-0111	1	50	0	0	1	C/T	G/C	A/A
NSCLC	case-0112	2	69	0	0	0	T/T	C/C	A/A
NSCLC	case-0113	1	58	1	0	0	C/T	C/C	A/A
NSCLC	case-0114	2	52	0	0	0	C/T	G/C	A/A
NSCLC	case-0115	2	56	0	0	1	C/C	G/C	A/A
NSCLC	case-0116	1	56	1	1	1	T/T	C/C	A/A
NSCLC	case-0117	1	34	1	0	1	C/C	C/C	A/A
NSCLC	case-0118	2	70	0	0	0	C/T	G/C	A/A
NSCLC	case-0119	2	36	0	0	0	C/T	G/G	G/A
NSCLC	case-0120	2	65	0	0	1	C/T	G/C	A/A
NSCLC	case-0121	2	40	0	0	1	T/T	C/C	G/A
NSCLC	case-0122	2	42	0	0	0	C/T	G/C	A/A
NSCLC	case-0123	1	62	0	0	1	C/T	G/C	A/A
NSCLC	case-0124	2	58	0	0	0	C/C	G/G	A/A
NSCLC	case-0125	1	53	1	0	0	T/T	G/G	A/A
NSCLC	case-0126	1	66	1	0	1	T/T	G/C	A/A
NSCLC	case-0127	1	58	1	1	0	T/T	G/C	G/A
NSCLC	case-0128	1	75	1	0	1	C/T	G/C	G/A
NSCLC	case-0129	2	57	0	0	1	C/T	G/C	A/A
NSCLC	case-0130	2	73	0	0	1	T/T	C/C	A/A
NSCLC	case-0131	2	61	0	0	0	T/T	C/C	A/A
NSCLC	case-0132	1	65	1	0	0	T/T	G/C	A/A
NSCLC	case-0133	2	63	0	0	1	C/C	G/C	A/A
NSCLC	case-0134	1	61	1	0	0	T/T	G/C	A/A
NSCLC	case-0135	2	60	0	0	1	C/T	G/G	A/A
NSCLC	case-0136	2	67	0	0	0	C/T	G/C	A/A
NSCLC	case-0137	2	64	0	0	0	T/T	C/C	A/A
NSCLC	case-0138	1	67	1	0	0	T/T	C/C	A/A
NSCLC	case-0139	1	65	1	0	0	T/T	C/C	A/A
NSCLC	case-0140	2	70	0	0	1	T/T	G/C	A/A
NSCLC	case-0141	1	87	0	0	1	C/T	G/G	A/A
NSCLC	case-0142	1	66	1	0	1	C/T	G/C	G/A
NSCLC	case-0143	1	46	1	0	0	T/T	G/C	A/A
NSCLC	case-0144	2	59	0	0	1	C/C	G/C	G/A
NSCLC	case-0145	2	58	0	0	0	T/T	C/C	G/A
NSCLC	case-0146	1	46	0	0	0	C/T	G/C	G/A
NSCLC	case-0147	1	62	1	0	0	C/T	C/C	A/A
NSCLC	case-0148	1	68	1	0	0	T/T	C/C	A/A
NSCLC	case-0149	1	76	0	0	1	C/T	C/C	G/G
NSCLC	case-0150	1	56	1	0	0	C/C	C/C	G/A
NSCLC	case-0151	2	46	0	0	1	T/T	C/C	A/A
NSCLC	case-0152	2	78	0	0	0	T/T	G/C	G/A
NSCLC	case-0153	2	51	0	0	0	T/T	G/C	A/A
NSCLC	case-0154	1	57	1	0	1	C/C	C/C	A/A
NSCLC	case-0155	1	60	1	0	0	T/T	G/C	A/A
NSCLC	case-0156	1	43	0	0	0	C/T	G/C	G/A

NSCLC	case-0157	1	64	1	0	1	C/C	C/C	G/A
NSCLC	case-0158	1	53	1	0	1	C/T	G/G	A/A
NSCLC	case-0159	1	71	1	0	0	C/T	G/C	G/A
NSCLC	case-0160	1	52	1	0	0	C/T	G/C	G/A
NSCLC	case-0161	1	58	0	1	0	C/C	G/G	A/A
NSCLC	case-0162	1	60	0	0	1	T/T	C/C	A/A
NSCLC	case-0163	2	53	0	0	1	C/C	G/C	A/A
NSCLC	case-0164	2	81	0	0	0	C/T	G/C	G/A
NSCLC	case-0165	2	66	0	0	0	T/T	G/G	G/A
NSCLC	case-0166	2	62	0	0	0	C/T	C/C	A/A
NSCLC	case-0167	2	48	0	0	0	T/T	G/C	A/A
NSCLC	case-0168	2	39	0	0	0	T/T	C/C	A/A
NSCLC	case-0169	2	39	0	0	0	C/T	G/C	A/A
NSCLC	case-0170	1	78	1	0	0	C/T	G/G	G/A
NSCLC	case-0171	1	66	1	0	0	C/T	G/C	G/A
NSCLC	case-0172	1	37	0	0	1	C/C	C/C	A/A
NSCLC	case-0173	1	61	1	0	0	T/T	G/C	G/A
NSCLC	case-0174	1	43	1	1	1	C/T	G/C	G/A
NSCLC	case-0175	1	73	1	0	0	C/T	C/C	G/A
NSCLC	case-0176	1	63	1	0	0	C/T	C/C	A/A
NSCLC	case-0177	1	61	1	0	0	T/T	G/C	G/A
NSCLC	case-0178	1	76	0	0	1	C/T	G/C	A/A
NSCLC	case-0179	1	57	1	0	0	T/T	G/C	A/A
NSCLC	case-0180	2	68	0	0	0	C/C	G/C	A/A
NSCLC	case-0181	2	63	0	0	0	C/T	G/G	A/A
NSCLC	case-0182	1	52	1	0	0	C/T	C/C	A/A
NSCLC	case-0183	1	46	1	0	0	C/T	G/C	G/A
NSCLC	case-0184	1	74	1	0	0	C/C	C/C	A/A
NSCLC	case-0185	1	43	1	0	1	C/T	G/C	A/A
NSCLC	case-0186	1	61	1	0	1	T/T	C/C	G/A
NSCLC	case-0187	2	75	0	0	1	T/T	G/G	A/A
NSCLC	case-0188	2	53	0	0	0	C/C	G/G	A/A
NSCLC	case-0189	2	38	0	0	0	C/T	G/C	A/A
NSCLC	case-0190	2	68	0	0	0	C/T	G/C	A/A
NSCLC	case-0191	2	28	0	0	0	C/T	C/C	A/A
NSCLC	case-0192	1	56	1	0	0	C/C	G/C	G/A
NSCLC	case-0193	2	68	0	0	1	T/T	G/C	A/A
NSCLC	case-0194	1	63	1	0	1	T/T	G/C	A/A
NSCLC	case-0195	2	50	0	0	1	C/T	C/C	G/A
NSCLC	case-0196	2	50	0	0	1	C/T	G/G	A/A
NSCLC	case-0197	1	67	1	0	0	C/T	C/C	A/A
NSCLC	case-0198	2	70	0	0	0	C/T	G/C	A/A
NSCLC	case-0199	2	46	0	0	0	T/T	C/C	G/A
NSCLC	case-0200	1	52	1	0	1	C/T	G/G	A/A
NSCLC	case-0201	1	62	1	0	0	T/T	G/C	A/A
NSCLC	case-0202	1	44	1	0	1	T/T	C/C	A/A
NSCLC	case-0203	2	55	0	0	0	C/C	C/C	A/A
NSCLC	case-0204	1	61	1	0	0	C/T	G/C	G/A
NSCLC	case-0205	1	50	1	0	0	C/C	G/C	A/A
NSCLC	case-0206	1	63	1	0	0	T/T	C/C	A/A
NSCLC	case-0207	2	61	0	0	0	C/C	C/C	A/A
NSCLC	case-0208	1	58	1	0	1	T/T	G/G	A/A
NSCLC	case-0209	2	76	0	0	1	T/T	G/G	G/A
NSCLC	case-0210	1	54	1	0	0	T/T	C/C	A/A

NSCLC	case-0211	2	63	0	0	0	C/T	G/C	G/A
NSCLC	case-0212	1	59	0	0	0	C/T	G/C	A/A
NSCLC	case-0213	1	70	1	0	1	C/T	C/C	A/A
NSCLC	case-0214	2	57	0	0	1	C/T	G/C	A/A
NSCLC	case-0215	1	56	1	0	0	C/T	C/C	A/A
NSCLC	case-0216	1	52	1	0	1	T/T	C/C	G/A
NSCLC	case-0217	2	50	0	0	0	T/T	G/C	G/A
NSCLC	case-0218	2	62	0	0	0	C/T	G/C	G/G
NSCLC	case-0219	1	51	1	0	1	C/T	C/C	A/A
NSCLC	case-0220	2	45	0	0	0	C/T	C/C	G/A
NSCLC	case-0221	1	60	1	0	1	T/T	G/C	A/A
NSCLC	case-0222	1	53	0	0	1	T/T	G/G	A/A
NSCLC	case-0223	1	59	0	0	1	C/C	C/C	G/G
NSCLC	case-0224	2	52	0	0	0	C/C	C/C	A/A
NSCLC	case-0225	2	52	0	0	1	C/T	C/C	A/A
NSCLC	case-0226	1	51	1	0	0	C/C	G/C	A/A
NSCLC	case-0227	1	61	1	0	0	T/T	G/G	A/A
NSCLC	case-0228	1	44	1	0	0	C/C	C/C	G/A
NSCLC	case-0229	2	60	0	0	1	T/T	C/C	A/A
NSCLC	case-0230	1	59	0	0	0	T/T	C/C	A/A
NSCLC	case-0231	1	52	1	0	1	C/T	G/C	G/A
NSCLC	case-0232	1	67	1	1	1	T/T	G/C	A/A
NSCLC	case-0233	1	75	0	0	0	T/T	G/C	G/A
NSCLC	case-0234	1	63	1	0	0	C/T	G/G	A/A
NSCLC	case-0235	1	58	1	0	1	T/T	G/G	A/A
NSCLC	case-0236	2	63	0	0	1	T/T	C/C	G/G
NSCLC	case-0237	1	67	1	1	0	C/T	C/C	A/A
NSCLC	case-0238	2	49	0	0	1	T/T	G/C	G/A
NSCLC	case-0239	2	36	0	0	0	T/T	G/C	G/A
NSCLC	case-0240	2	56	0	0	0	T/T	G/C	A/A
NSCLC	case-0241	1	62	1	0	0	C/T	C/C	G/A
NSCLC	case-0242	2	65	0	0	0	T/T	C/C	G/A
NSCLC	case-0243	1	48	1	0	1	C/C	G/G	A/A
NSCLC	case-0244	2	49	0	0	0	T/T	C/C	A/A
NSCLC	case-0245	1	66	1	0	0	C/T	C/C	G/A
NSCLC	case-0246	2	60	0	0	0	C/T	C/C	A/A
NSCLC	case-0247	1	69	1	0	0	C/T	G/C	A/A
NSCLC	case-0248	2	59	0	0	0	C/T	G/C	G/A
NSCLC	case-0249	1	76	1	0	0	C/T	C/C	A/A
NSCLC	case-0250	1	63	1	1	0	C/T	C/C	A/A
NSCLC	case-0251	2	60	0	0	0	T/T	G/C	G/A
NSCLC	case-0252	2	59	0	0	0	C/T	G/C	G/A
NSCLC	case-0253	2	35	0	0	0	C/T	G/C	A/A
NSCLC	case-0254	1	53	1	0	1	T/T	C/C	A/A
NSCLC	case-0255	2	64	0	0	1	T/T	G/C	A/A
NSCLC	case-0256	2	53	0	0	1	C/T	C/C	A/A
NSCLC	case-0257	2	62	0	0	0	C/T	G/C	A/A
NSCLC	case-0258	1	53	1	0	1	T/T	G/G	G/G
NSCLC	case-0259	1	69	0	0	0	T/T	C/C	A/A
NSCLC	case-0260	1	40	1	0	1	C/T	G/C	A/A
NSCLC	case-0261	2	72	0	0	0	C/T	C/C	A/A
NSCLC	case-0262	1	45	0	0	1	C/C	G/C	A/A
NSCLC	case-0263	2	52	0	0	1	C/T	C/C	A/A
NSCLC	case-0264	1	46	1	0	1	C/T	C/C	G/A

NSCLC	case-0265	2	44	0	0	1	C/T	G/C	A/A
NSCLC	case-0266	1	49	1	1	0	C/T	G/C	A/A
NSCLC	case-0267	1	53	1	0	0	C/C	G/C	A/A
NSCLC	case-0268	1	58	0	0	0	T/T	C/C	G/A
NSCLC	case-0269	2	52	0	0	1	C/T	G/C	G/A
NSCLC	case-0270	2	67	0	0	0	C/T	C/C	A/A
NSCLC	case-0271	1	52	1	1	0	C/T	C/C	A/A
NSCLC	case-0272	2	67	0	0	0	T/T	G/G	G/A
NSCLC	case-0273	2	67	0	0	0	C/C	C/C	A/A
NSCLC	case-0274	1	59	1	0	0	C/C	C/C	A/A
NSCLC	case-0275	2	63	0	0	0	C/T	C/C	G/A
NSCLC	case-0276	1	54	1	0	1	T/T	C/C	A/A
NSCLC	case-0277	2	61	0	0	0	T/T	G/C	A/A
NSCLC	case-0278	1	57	0	0	1	C/T	C/C	A/A
NSCLC	case-0279	2	69	0	0	0	C/C	G/G	A/A
NSCLC	case-0280	1	67	0	0	0	C/C	C/C	G/A
NSCLC	case-0281	2	60	0	0	0	C/T	C/C	A/A
NSCLC	case-0282	1	64	1	0	0	C/T	C/C	G/G
NSCLC	case-0283	2	72	0	0	0	T/T	G/G	A/A
NSCLC	case-0284	1	70	0	0	1	T/T	G/C	A/A
NSCLC	case-0285	2	46	0	0	0	T/T	G/C	G/A
NSCLC	case-0286	1	61	0	0	1	C/T	G/C	A/A
NSCLC	case-0287	1	62	1	1	0	C/T	C/C	A/A
NSCLC	case-0288	1	65	1	0	1	C/C	C/C	A/A
NSCLC	case-0289	1	77	1	1	0	C/C	C/C	A/A
NSCLC	case-0290	2	38	0	0	0	C/C	G/C	A/A
NSCLC	case-0291	2	44	0	0	0	C/T	C/C	A/A
NSCLC	case-0292	1	60	0	1	1	C/C	G/C	A/A
NSCLC	case-0293	1	76	0	0	0	C/T	G/C	A/A
NSCLC	case-0294	1	46	1	0	0	T/T	C/C	G/A
NSCLC	case-0295	2	43	0	0	1	C/T	G/G	A/A
NSCLC	case-0296	2	57	0	0	0	T/T	C/C	G/A
NSCLC	case-0297	2	69	0	0	0	T/T	C/C	A/A
NSCLC	case-0298	1	64	0	1	0	C/C	C/C	A/A
NSCLC	case-0299	1	77	1	0	0	C/C	C/C	G/A
NSCLC	case-0300	1	62	1	1	1	C/T	G/C	G/A
NSCLC	case-0301	2	65	0	0	1	C/C	C/C	G/A
NSCLC	case-0302	2	62	0	0	1	C/T	G/C	A/A
NSCLC	case-0303	1	58	1	0	0	C/T	C/C	A/A
NSCLC	case-0304	1	74	1	0	1	T/T	G/C	A/A
NSCLC	case-0305	1	56	1	1	1	C/T	C/C	A/A
NSCLC	case-0306	2	60	0	0	0	C/C	G/C	A/A
NSCLC	case-0307	1	27	0	1	0	C/T	G/C	A/A
NSCLC	case-0308	1	87	1	1	1	T/T	G/C	A/A
NSCLC	case-0309	1	48	1	0	0	T/T	G/C	G/A
NSCLC	case-0310	2	73	0	0	0	C/C	G/C	A/A
NSCLC	case-0311	1	65	1	0	0	T/T	C/C	A/A
NSCLC	case-0312	2	59	0	0	1	C/T	G/C	G/G
NSCLC	case-0313	1	64	0	0	0	C/T	C/C	G/A
NSCLC	case-0314	2	59	0	0	0	C/C	G/G	G/A
NSCLC	case-0315	1	54	1	0	1	C/T	C/C	G/A
NSCLC	case-0316	1	63	1	1	0	C/C	C/C	A/A
NSCLC	case-0317	2	59	0	0	0	C/C	G/C	G/A
NSCLC	case-0318	2	60	0	0	0	T/T	G/G	A/A

NSCLC	case-0319	1	67	1	1	0	C/T	C/C	G/A
NSCLC	case-0320	1	62	1	1	0	T/T	G/C	A/A
NSCLC	case-0321	2	66	0	0	1	C/T	C/C	G/A
NSCLC	case-0322	2	74	0	0	0	C/T	C/C	A/A
NSCLC	case-0323	1	65	1	1	0	T/T	G/C	A/A
NSCLC	case-0324	1	63	0	0	1	T/T	G/C	A/A
NSCLC	case-0325	2	67	0	0	1	C/T	C/C	G/G
NSCLC	case-0326	1	52	1	0	0	C/C	C/C	A/A
NSCLC	case-0327	2	61	0	0	0	C/T	G/G	A/A
NSCLC	case-0328	1	67	0	0	0	C/T	G/C	G/A
NSCLC	case-0329	1	72	0	0	0	C/T	G/C	G/A
NSCLC	case-0330	1	70	1	1	1	C/C	G/C	A/A
NSCLC	case-0331	2	59	0	0	0	C/T	G/C	G/A
NSCLC	case-0332	1	56	0	1	1	C/T	C/C	G/A
NSCLC	case-0333	1	76	1	1	0	T/T	C/C	G/A
NSCLC	case-0334	1	74	0	0	0	C/T	C/C	A/A
NSCLC	case-0335	2	26	0	0	1	T/T	C/C	A/A
NSCLC	case-0336	2	56	0	0	1	T/T	C/C	G/G
NSCLC	case-0337	2	69	0	0	1	T/T	G/C	A/A
NSCLC	case-0338	1	48	0	0	1	C/T	G/C	A/A
NSCLC	case-0339	1	55	1	1	0	C/C	G/C	A/A
NSCLC	case-0340	2	65	0	0	0	C/T	G/C	G/A
NSCLC	case-0341	2	51	0	0	0	C/T	G/C	A/A
NSCLC	case-0342	1	58	1	1	1	C/T	G/C	A/A
NSCLC	case-0343	2	44	0	0	1	C/T	G/C	A/A
NSCLC	case-0344	1	73	0	0	1	C/T	G/C	G/A
NSCLC	case-0345	2	70	0	0	1	C/T	G/C	G/A
NSCLC	case-0346	1	64	1	0	0	T/T	C/C	A/A
NSCLC	case-0347	2	49	0	0	0	C/T	G/C	A/A
NSCLC	case-0348	1	42	0	1	1	C/C	G/C	A/A
NSCLC	case-0349	1	79	0	0	1	C/T	C/C	A/A
NSCLC	case-0350	1	52	1	0	0	C/T	G/C	G/A
NSCLC	case-0351	2	41	0	0	0	C/T	G/G	G/A
NSCLC	case-0352	1	40	0	1	0	C/C	G/G	G/A
NSCLC	case-0353	2	61	0	0	1	T/T	G/C	A/A
NSCLC	case-0354	1	63	1	1	1	C/T	G/G	G/A
NSCLC	case-0355	1	62	1	1	0	T/T	G/C	A/A
NSCLC	case-0356	2	40	0	0	1	C/C	G/C	G/A
NSCLC	case-0357	2	49	0	0	0	T/T	G/C	A/A
NSCLC	case-0358	2	44	0	0	0	C/T	G/C	A/A
NSCLC	case-0359	1	68	1	1	1	T/T	G/C	A/A
NSCLC	case-0360	2	54	0	0	1	C/T	G/C	A/A
NSCLC	case-0361	2	61	0	0	1	C/T	G/C	A/A
NSCLC	case-0362	2	74	0	0	0	C/T	G/C	A/A
NSCLC	case-0363	1	59	1	1	1	T/T	C/C	A/A
NSCLC	case-0364	1	68	0	0	0	C/C	G/C	A/A
NSCLC	case-0365	1	55	0	0	0	C/T	G/C	A/A
NSCLC	case-0366	1	72	1	0	1	C/C	G/C	G/A
NSCLC	case-0367	1	52	1	1	1	T/T	C/C	A/A
NSCLC	case-0368	1	59	1	1	1	C/T	G/C	A/A
NSCLC	case-0369	1	62	1	0	0	C/T	G/C	G/A
NSCLC	case-0370	2	42	0	0	0	C/C	C/C	A/A
NSCLC	case-0371	2	62	0	0	0	C/C	G/C	G/A
NSCLC	case-0372	2	28	0	0	0	C/T	G/C	A/A

NSCLC	case-0373	2	66	0	0	1	T/T	C/C	A/A
NSCLC	case-0374	2	45	0	0	0	T/T	G/C	A/A
NSCLC	case-0375	1	41	0	0	0	T/T	C/C	A/A
NSCLC	case-0376	1	66	1	1	0	C/T	G/C	A/A
NSCLC	case-0377	2	57	0	0	0	C/T	C/C	A/A
NSCLC	case-0378	1	72	1	0	1	C/T	G/C	A/A
NSCLC	case-0379	2	67	0	0	1	C/T	G/C	A/A
NSCLC	case-0380	1	66	0	0	0	C/T	G/C	A/A
NSCLC	case-0381	1	69	1	1	0	T/T	C/C	G/A
NSCLC	case-0382	2	46	0	0	1	T/T	G/C	A/A
NSCLC	case-0383	1	66	0	0	1	C/C	G/C	A/A
NSCLC	case-0384	1	70	0	0	1	C/T	G/G	A/A
NSCLC	case-0385	1	51	1	0	0	T/T	G/G	A/A
NSCLC	case-0386	1	48	1	0	0	C/T	G/C	A/A
NSCLC	case-0387	2	57	0	0	0	C/T	G/G	G/A
NSCLC	case-0388	1	62	1	0	1	C/T	C/C	G/A
NSCLC	case-0389	1	55	1	1	1	C/T	G/C	A/A
NSCLC	case-0390	1	64	1	0	0	C/T	G/G	A/A
NSCLC	case-0391	2	53	0	0	0	T/T	G/G	A/A
NSCLC	case-0392	1	67	1	1	1	C/T	G/C	A/A
NSCLC	case-0393	2	55	0	0	1	C/T	G/C	G/A
NSCLC	case-0394	2	69	0	0	0	C/C	G/G	A/A
NSCLC	case-0395	1	43	1	1	0	C/T	C/C	G/A
NSCLC	case-0396	2	48	0	0	1	C/T	G/C	G/A
NSCLC	case-0397	2	81	0	0	0	C/T	G/C	A/A
NSCLC	case-0398	1	62	1	0	0	C/T	G/G	A/A
NSCLC	case-0399	2	43	0	0	0	T/T	G/C	A/A
NSCLC	case-0400	1	57	1	0	1	C/C	G/C	A/A
NSCLC	case-0401	2	46	0	0	0	T/T	C/C	A/A
NSCLC	case-0402	1	61	1	0	0	C/T	C/C	A/A
NSCLC	case-0403	2	61	0	0	0	C/T	C/C	A/A
NSCLC	case-0404	2	36	0	0	0	C/C	C/C	A/A
NSCLC	case-0405	2	70	0	0	0	T/T	G/C	G/A
NSCLC	case-0406	1	67	1	1	0	T/T	G/C	A/A
NSCLC	case-0407	1	56	1	1	1	T/T	C/C	G/A
NSCLC	case-0408	2	60	1	1	1	T/T	C/C	A/A
NSCLC	case-0409	2	66	0	0	0	C/T	G/C	A/A
NSCLC	case-0410	1	69	1	1	0	C/T	C/C	G/A
NSCLC	case-0411	1	55	1	1	0	T/T	G/G	A/A
NSCLC	case-0412	1	61	1	1	0	C/C	G/C	G/A
NSCLC	case-0413	2	58	0	0	0	T/T	C/C	A/A
NSCLC	case-0414	2	54	0	0	1	C/T	G/C	A/A
NSCLC	case-0415	1	54	1	1	0	T/T	G/C	G/A
NSCLC	case-0416	1	59	0	0	0	C/T	C/C	A/A
NSCLC	case-0417	2	53	0	0	0	C/C	C/C	A/A
NSCLC	case-0418	1	71	1	1	0	C/T	G/C	G/A
NSCLC	case-0419	1	63	1	1	1	T/T	G/C	A/A
NSCLC	case-0420	2	80	0	0	0	C/T	C/C	G/A
NSCLC	case-0421	1	80	1	1	0	C/T	G/C	A/A
NSCLC	case-0422	1	40	0	0	0	C/C	G/C	A/A
NSCLC	case-0423	2	51	0	0	1	T/T	G/G	A/A
NSCLC	case-0424	2	49	0	0	1	C/C	G/C	G/A
NSCLC	case-0425	2	62	0	0	0	T/T	C/C	G/A
NSCLC	case-0426	1	59	0	1	0	T/T	C/C	A/A

NSCLC	case-0427	2	68	0	0	1	T/T	G/C	A/A
NSCLC	case-0428	2	63	0	0	1	C/T	C/C	A/A
NSCLC	case-0429	2	64	0	0	1	T/T	G/G	G/A
NSCLC	case-0430	1	58	0	0	0	C/C	G/C	A/A
NSCLC	case-0431	2	32	0	0	0	C/C	C/C	A/A
NSCLC	case-0432	1	79	0	0	0	T/T	G/G	G/A
NSCLC	case-0433	1	76	0	0	0	C/C	G/G	A/A
NSCLC	case-0434	2	74	0	0	1	C/C	G/C	A/A
NSCLC	case-0435	1	71	1	1	0	C/T	G/C	A/A
NSCLC	case-0436	1	74	1	0	0	C/C	G/C	A/A
NSCLC	case-0437	1	60	1	0	1	C/T	C/C	A/A
NSCLC	case-0438	2	55	0	0	0	T/T	G/C	G/A
NSCLC	case-0439	1	64	1	1	0	C/T	C/C	A/A
NSCLC	case-0440	1	70	1	1	0	T/T	G/C	G/A
NSCLC	case-0441	1	76	0	0	0	C/C	C/C	G/G
NSCLC	case-0442	1	62	0	1	0	T/T	G/C	G/A
NSCLC	case-0443	1	80	1	0	0	T/T	C/C	G/A
NSCLC	case-0444	1	78	0	0	1	T/T	G/C	A/A
NSCLC	case-0445	1	69	0	0	0	C/T	G/C	G/A
NSCLC	case-0446	2	74	0	0	0	C/T	G/C	G/A
NSCLC	case-0447	1	57	0	0	0	C/T	G/C	A/A
NSCLC	case-0448	1	67	1	0	0	T/T	G/G	A/A
NSCLC	case-0449	2	57	0	0	0	T/T	G/C	G/A
NSCLC	case-0450	1	64	1	1	1	C/T	G/G	A/A
NSCLC	case-0451	2	26	0	0	1	C/T	G/C	A/A
NSCLC	case-0452	1	61	1	1	0	T/T	G/C	A/A
NSCLC	case-0453	1	65	1	1	0	T/T	G/C	A/A
NSCLC	case-0454	1	64	1	0	0	C/C	G/C	A/A
NSCLC	case-0455	2	79	0	0	0	C/T	G/C	A/A
NSCLC	case-0456	1	49	1	1	1	T/T	G/C	A/A
NSCLC	case-0457	1	61	0	1	0	C/T	G/G	A/A
NSCLC	case-0458	1	58	1	1	0	C/T	C/C	A/A
NSCLC	case-0459	1	66	1	0	0	C/T	C/C	G/A
NSCLC	case-0460	1	77	1	0	0	C/T	G/C	G/A
NSCLC	case-0461	2	46	0	0	0	C/C	C/C	A/A
NSCLC	case-0462	1	60	1	0	0	C/T	G/C	A/A
NSCLC	case-0463	2	75	0	0	0	T/T	G/C	A/A
NSCLC	case-0464	1	61	0	0	0	C/T	C/C	A/A
NSCLC	case-0465	1	64	0	0	0	C/T	C/C	A/A
NSCLC	case-0466	2	46	0	0	0	C/T	G/C	G/A
NSCLC	case-0467	1	82	0	0	1	T/T	C/C	G/A
NSCLC	case-0468	1	71	1	0	0	C/T	G/C	A/A
NSCLC	case-0469	1	61	0	0	1	C/T	G/C	G/A
NSCLC	case-0470	2	71	0	0	0	T/T	G/G	A/A
NSCLC	case-0471	2	67	0	1	1	C/C	C/C	A/A
NSCLC	case-0472	2	55	0	0	0	T/T	G/C	A/A
NSCLC	case-0473	1	77	0	0	0	T/T	G/C	A/A
NSCLC	case-0474	1	52	1	1	0	C/T	G/C	A/A
NSCLC	case-0475	2	64	0	0	0	C/T	G/C	G/A
NSCLC	case-0476	2	46	0	0	0	C/C	G/C	G/A
NSCLC	case-0477	2	61	0	0	1	T/T	G/C	G/A
NSCLC	case-0478	2	60	0	0	0	C/T	G/C	G/A
NSCLC	case-0479	1	53	1	1	1	C/T	C/C	A/A
NSCLC	case-0480	1	75	0	0	0	T/T	G/C	A/A

NSCLC	case-0481	1	79	0	0	1	C/T	G/C	A/A
NSCLC	case-0482	1	70	1	1	0	C/T	G/C	G/A
NSCLC	case-0483	1	78	1	0	0	C/C	G/C	G/A
NSCLC	case-0484	1	70	1	0	0	C/T	G/C	A/A
NSCLC	case-0485	1	81	0	0	0	C/T	G/G	A/A
NSCLC	case-0486	1	58	1	0	0	C/T	G/C	A/A
NSCLC	case-0487	1	61	1	0	0	C/T	C/C	A/A
NSCLC	case-0488	1	68	0	0	0	T/T	C/C	A/A
NSCLC	case-0489	1	70	1	1	0	T/T	G/C	A/A
NSCLC	case-0490	1	68	1	1	0	C/T	G/C	A/A
NSCLC	case-0491	2	59	0	0	0	C/T	G/C	A/A
NSCLC	case-0492	2	55	0	0	1	C/T	C/C	A/A
NSCLC	case-0493	2	59	0	0	1	C/T	C/C	A/A
NSCLC	case-0494	1	70	0	0	1	T/T	G/C	A/A
NSCLC	case-0495	1	52	1	0	0	C/T	G/C	A/A
NSCLC	case-0496	1	65	1	0	0	T/T	G/C	G/A
NSCLC	case-0497	2	58	0	0	0	T/T	G/C	A/A
NSCLC	case-0498	1	74	1	1	0	C/C	G/C	A/A
NSCLC	case-0499	2	56	0	0	0	C/T	G/C	A/A
NSCLC	case-0500	1	51	1	0	1	C/C	C/C	A/A
NSCLC	case-0501	2	66	0	0	0	C/T	C/C	A/A
NSCLC	case-0502	1	48	1	0	0	C/T	G/C	A/A
NSCLC	case-0503	2	56	0	0	0	C/C	C/C	A/A
NSCLC	case-0504	1	57	1	0	0	C/T	G/C	A/A
NSCLC	case-0505	2	67	0	0	0	C/T	G/C	A/A
NSCLC	case-0506	1	54	1	0	0	T/T	C/C	A/A
NSCLC	case-0507	2	59	0	0	1	C/C	C/C	A/A
NSCLC	case-0508	1	66	0	0	0	C/C	G/G	G/A
NSCLC	case-0509	1	63	0	0	0	C/T	G/C	G/A
NSCLC	case-0510	2	65	1	0	1	T/T	G/G	A/A
NSCLC	case-0511	1	67	0	0	1	C/T	G/C	A/A
NSCLC	case-0512	1	63	0	0	1	C/T	G/C	A/A
NSCLC	case-0513	1	62	1	0	1	C/T	G/G	G/A
NSCLC	case-0514	1	81	0	0	1	T/T	C/C	A/A
NSCLC	case-0515	2	51	0	0	1	T/T	G/G	A/A
NSCLC	case-0516	1	62	0	0	0	T/T	G/C	G/A
NSCLC	case-0517	2	52	0	0	0	T/T	G/C	A/A
NSCLC	case-0518	2	48	0	0	1	T/T	G/G	G/A
NSCLC	case-0519	1	67	1	1	0	C/T	G/C	G/A
NSCLC	case-0520	1	62	1	1	0	T/T	G/C	A/A
NSCLC	case-0521	1	44	1	1	0	C/T	C/C	A/A
NSCLC	case-0522	1	66	1	1	0	C/T	G/C	A/A
NSCLC	case-0523	2	71	0	0	0	T/T	C/C	A/A
NSCLC	case-0524	2	61	0	0	1	C/C	G/C	A/A
NSCLC	case-0525	1	43	0	1	0	C/T	C/C	A/A
NSCLC	case-0526	1	66	1	1	0	T/T	C/C	A/A
NSCLC	case-0527	1	71	1	0	1	C/C	G/G	A/A
NSCLC	case-0528	2	72	0	0	0	C/T	C/C	A/A
NSCLC	case-0529	1	69	1	0	0	T/T	C/C	A/A
NSCLC	case-0530	1	85	0	1	1	C/C	C/C	A/A
NSCLC	case-0531	2	65	0	0	1	C/T	G/C	A/A
NSCLC	case-0532	2	64	0	0	1	T/T	G/C	A/A
NSCLC	case-0533	1	35	1	0	0	T/T	C/C	G/A
NSCLC	case-0534	1	55	1	0	0	C/C	C/C	A/A

NSCLC	case-0535	2	48	0	0	1	?	?	?
NSCLC	case-0536	2	55	0	0	1	C/T	C/C	A/A
NSCLC	case-0537	2	40	0	0	0	?	?	?
NSCLC	case-0538	2	46	0	0	0	C/T	G/C	G/A
NSCLC	case-0539	1	68	0	1	0	C/T	G/G	A/A
NSCLC	case-0540	2	71	0	0	1	C/T	C/C	A/A
NSCLC	case-0541	2	37	0	0	1	C/T	G/G	G/G
NSCLC	case-0542	2	64	0	0	0	T/T	G/G	A/A
NSCLC	case-0543	1	44	1	1	1	T/T	C/C	A/A
NSCLC	case-0544	1	46	0	0	1	T/T	C/C	A/A
NSCLC	case-0545	1	64	1	1	0	T/T	G/C	A/A
NSCLC	case-0546	1	54	1	0	1	C/T	G/C	A/A
NSCLC	case-0547	2	63	0	0	0	C/T	G/C	A/A
NSCLC	case-0548	1	64	1	1	0	T/T	C/C	A/A
NSCLC	case-0549	2	71	0	0	0	C/T	C/C	A/A
NSCLC	case-0550	2	53	0	0	0	C/T	G/G	G/A
NSCLC	case-0551	2	49	0	0	0	C/T	G/C	G/A
NSCLC	case-0552	2	35	0	0	0	C/C	G/C	G/A
NSCLC	case-0553	2	48	0	0	0	C/T	C/C	G/A
NSCLC	case-0554	2	65	0	0	0	C/T	G/C	A/A
NSCLC	case-0555	1	62	0	0	0	C/T	G/C	A/A
NSCLC	case-0556	1	68	0	1	0	T/T	G/C	A/A
NSCLC	case-0557	2	80	0	0	1	C/T	G/G	A/A
NSCLC	case-0558	2	53	0	0	0	C/T	G/C	A/A
NSCLC	case-0559	1	55	0	0	1	T/T	C/C	A/A
NSCLC	case-0560	1	33	0	0	0	C/C	C/C	A/A
NSCLC	case-0561	2	64	0	0	1	C/T	G/G	A/A
NSCLC	case-0562	2	63	0	0	1	C/T	G/G	A/A
NSCLC	case-0563	1	81	0	0	0	C/T	C/C	G/A
NSCLC	case-0564	2	68	0	0	0	C/T	G/C	A/A
NSCLC	case-0565	1	63	0	0	1	C/T	C/C	G/A
NSCLC	case-0566	2	62	0	0	0	C/T	G/C	A/A
NSCLC	case-0567	2	53	0	0	0	C/C	C/C	G/A
NSCLC	case-0568	2	56	0	0	0	T/T	C/C	A/A
NSCLC	case-0569	1	61	1	1	0	C/C	G/C	A/A
NSCLC	case-0570	1	70	1	1	0	C/T	G/G	A/A
NSCLC	case-0571	1	55	0	0	0	T/T	C/C	G/G
NSCLC	case-0572	1	43	0	0	0	T/T	C/C	A/A
NSCLC	case-0573	1	67	1	1	1	C/T	C/C	A/A
NSCLC	case-0574	2	49	0	0	0	T/T	G/G	A/A
NSCLC	case-0575	2	63	0	0	0	C/T	C/C	A/A
NSCLC	case-0576	1	64	1	1	0	C/T	C/C	A/A
NSCLC	case-0577	2	50	0	0	0	T/T	G/C	A/A
NSCLC	case-0578	1	72	0	0	1	T/T	G/C	G/A
NSCLC	case-0579	2	66	0	0	1	T/T	C/C	A/A
NSCLC	case-0580	2	57	0	0	0	C/T	G/C	G/A
NSCLC	case-0581	1	62	1	0	0	T/T	G/C	A/A
NSCLC	case-0582	1	43	1	0	0	T/T	G/G	G/A
NSCLC	case-0583	2	62	0	0	1	T/T	C/C	A/A
NSCLC	case-0584	1	52	0	0	0	T/T	G/C	A/A
NSCLC	case-0585	2	54	0	0	0	T/T	G/C	A/A
NSCLC	case-0586	1	42	0	0	1	C/T	G/C	A/A
NSCLC	case-0587	1	62	1	1	0	C/T	G/C	A/A
NSCLC	case-0588	1	54	0	0	0	C/T	G/C	G/A

NSCLC	case-0589	1	61	0	1	0	T/T	G/C	A/A
NSCLC	case-0590	1	75	1	1	0	T/T	G/C	A/A
NSCLC	case-0591	2	48	0	0	0	C/T	G/C	A/A
NSCLC	case-0592	1	66	1	1	1	T/T	C/C	A/A
NSCLC	case-0593	2	52	1	1	0	C/T	G/C	G/A
NSCLC	case-0594	1	50	0	0	0	C/T	C/C	G/A
NSCLC	case-0595	2	55	0	0	0	T/T	G/C	A/A
NSCLC	case-0596	1	61	1	1	0	C/T	C/C	A/A
NSCLC	case-0597	2	53	0	0	0	C/C	G/G	G/A
NSCLC	case-0598	2	53	0	0	1	C/T	G/C	G/A
NSCLC	case-0599	1	66	0	0	0	C/C	G/C	A/A
NSCLC	case-0600	1	68	0	0	0	C/C	G/C	A/A
NSCLC	case-0601	2	62	0	0	0	T/T	G/C	A/A
NSCLC	case-0602	2	48	0	0	0	T/T	G/C	G/A
NSCLC	case-0603	1	57	1	1	0	C/T	G/C	A/A
NSCLC	case-0604	2	44	0	0	1	C/T	C/C	A/A
NSCLC	case-0605	2	64	0	0	1	C/T	G/C	G/A
NSCLC	case-0606	1	55	1	1	1	C/T	C/C	A/A
NSCLC	case-0607	2	63	0	0	0	C/C	G/C	G/G
NSCLC	case-0608	2	66	0	0	1	T/T	G/C	A/A
NSCLC	case-0609	1	53	1	0	0	T/T	G/G	A/A
NSCLC	case-0610	1	33	1	1	1	C/T	C/C	G/A
NSCLC	case-0611	2	53	0	0	1	T/T	C/C	A/A
NSCLC	case-0612	2	76	0	0	0	C/T	C/C	A/A
NSCLC	case-0613	2	54	0	0	0	C/T	C/C	A/A
NSCLC	case-0614	2	53	0	0	1	T/T	G/C	G/A
NSCLC	case-0615	2	57	0	0	0	C/T	G/G	A/A
NSCLC	case-0616	1	62	0	0	0	C/T	C/C	A/A
NSCLC	case-0617	1	78	1	1	0	C/C	C/C	A/A
NSCLC	case-0618	1	51	0	0	1	C/T	C/C	A/A
NSCLC	case-0619	2	48	0	0	0	C/T	C/C	A/A
NSCLC	case-0620	2	78	0	0	1	C/C	C/C	A/A
NSCLC	case-0621	2	63	0	0	1	C/T	C/C	G/A
NSCLC	case-0622	2	75	0	0	1	C/T	G/G	A/A
NSCLC	case-0623	2	46	0	0	0	T/T	G/G	A/A
NSCLC	case-0624	1	47	1	0	0	C/T	C/C	A/A
NSCLC	case-0625	2	65	0	0	0	C/T	G/C	G/A
NSCLC	case-0626	2	81	0	0	1	C/T	G/C	G/A
NSCLC	case-0627	2	54	0	0	0	C/T	G/C	G/A
NSCLC	case-0628	1	75	1	1	1	T/T	C/C	A/A
NSCLC	case-0629	1	54	0	1	0	C/T	C/C	A/A
NSCLC	case-0630	1	59	1	1	1	T/T	G/C	A/A
NSCLC	case-0631	1	62	1	0	0	C/C	C/C	A/A
NSCLC	case-0632	2	39	0	0	0	C/C	G/C	G/G
NSCLC	case-0633	1	54	1	1	1	C/C	G/C	A/A
NSCLC	case-0634	1	54	1	1	0	C/T	G/C	A/A
NSCLC	case-0635	2	48	0	0	1	C/T	G/C	G/A
NSCLC	case-0636	1	59	1	1	0	C/T	G/C	A/A
NSCLC	case-0637	1	77	0	0	0	C/C	G/G	A/A
NSCLC	case-0638	2	51	0	0	0	C/T	G/G	A/A
NSCLC	case-0639	2	45	0	0	0	T/T	G/C	A/A
NSCLC	case-0640	1	53	1	1	0	C/T	G/C	A/A
NSCLC	case-0641	1	80	1	0	0	C/T	G/C	A/A
NSCLC	case-0642	2	57	0	0	0	T/T	C/C	G/A

NSCLC	case-0643	1	37	1	0	0	T/T	G/C	A/A
NSCLC	case-0644	1	70	0	0	0	C/T	C/C	G/A
NSCLC	case-0645	1	54	0	1	0	T/T	G/C	A/A
NSCLC	case-0646	2	64	0	0	0	C/T	C/C	A/A
NSCLC	case-0647	1	53	1	1	1	T/T	C/C	G/A
NSCLC	case-0648	2	55	0	0	0	?	?	?
NSCLC	case-0649	2	59	0	0	1	T/T	G/C	A/A
NSCLC	case-0650	2	71	0	0	0	C/C	G/C	G/A
NSCLC	case-0651	1	62	0	0	0	T/T	G/C	G/G
NSCLC	case-0652	1	44	0	0	1	T/T	G/C	A/A
NSCLC	case-0653	1	57	0	0	1	?	?	?
NSCLC	case-0654	1	61	0	0	1	C/C	C/C	A/A
NSCLC	case-0655	1	61	0	0	0	C/T	G/C	A/A
NSCLC	case-0656	2	54	0	0	0	C/T	G/G	A/A
NSCLC	case-0657	1	71	1	1	0	C/C	G/C	A/A
NSCLC	case-0658	2	62	0	0	0	T/T	C/C	A/A
NSCLC	case-0659	1	30	0	0	1	T/T	G/C	A/A
NSCLC	case-0660	2	61	0	0	0	T/T	C/C	A/A
NSCLC	case-0661	2	51	0	0	0	T/T	C/C	A/A
NSCLC	case-0662	2	65	0	0	0	C/C	G/C	A/A
NSCLC	case-0663	2	72	0	0	0	C/T	G/C	A/A
NSCLC	case-0664	2	71	0	0	1	C/T	C/C	A/A
NSCLC	case-0665	2	66	0	0	0	T/T	G/C	A/A
NSCLC	case-0666	1	60	1	1	1	C/C	C/C	G/A
NSCLC	case-0667	1	68	1	1	0	C/T	C/C	G/A
NSCLC	case-0668	1	65	1	0	0	C/T	C/C	G/A
NSCLC	case-0669	1	51	0	1	1	C/C	C/C	A/A
NSCLC	case-0670	2	45	0	0	0	T/T	C/C	A/A
NSCLC	case-0671	1	66	0	1	0	T/T	G/C	G/A
NSCLC	case-0672	1	50	1	1	1	T/T	C/C	A/A
NSCLC	case-0673	2	49	0	0	0	T/T	G/C	A/A
NSCLC	case-0674	2	59	0	0	0	C/T	C/C	A/A
NSCLC	case-0675	2	59	0	0	0	T/T	G/C	G/A
NSCLC	case-0676	1	71	1	0	1	C/C	G/G	A/A
NSCLC	case-0677	1	71	1	1	0	C/T	C/C	A/A
NSCLC	case-0678	2	79	0	0	0	C/C	G/C	A/A
NSCLC	case-0679	1	80	0	0	0	C/T	C/C	A/A
NSCLC	case-0680	1	64	1	1	1	C/C	G/C	G/A
NSCLC	case-0681	1	67	1	0	0	C/C	C/C	G/A
NSCLC	case-0682	2	69	0	0	1	C/C	C/C	G/A
NSCLC	case-0683	1	64	1	1	1	C/C	G/C	A/A
NSCLC	case-0684	2	60	0	0	0	T/T	G/C	G/A
NSCLC	case-0685	2	46	0	0	0	C/T	G/C	A/A
NSCLC	case-0686	2	59	0	0	0	C/T	C/C	A/A
NSCLC	case-0687	1	59	1	0	0	C/C	C/C	A/A
NSCLC	case-0688	2	63	0	0	0	T/T	G/C	G/A
NSCLC	case-0689	1	42	0	0	0	C/T	G/C	G/A
NSCLC	case-0690	2	52	0	0	1	C/T	G/C	A/A
NSCLC	case-0691	1	47	0	0	1	T/T	C/C	A/A
NSCLC	case-0692	2	70	0	0	1	C/T	C/C	A/A
NSCLC	case-0693	1	62	1	1	1	C/T	C/C	G/A
NSCLC	case-0694	2	45	0	0	0	T/T	C/C	A/A
NSCLC	case-0695	2	52	0	0	0	T/T	G/C	G/A
NSCLC	case-0696	2	72	0	0	1	C/C	C/C	A/A

NSCLC	case-0697	2	57	0	0	0	T/T	C/C	A/A
NSCLC	case-0698	2	58	0	0	1	T/T	G/C	G/A
NSCLC	case-0699	1	58	1	1	0	C/T	C/C	A/A
NSCLC	case-0700	1	59	1	0	1	C/T	G/G	G/A
NSCLC	case-0701	2	61	0	0	1	C/C	C/C	A/A
NSCLC	case-0702	2	67	0	0	0	C/T	C/C	A/A
NSCLC	case-0703	2	35	0	0	0	C/T	C/C	G/A
NSCLC	case-0704	2	61	0	0	1	T/T	C/C	G/A
NSCLC	case-0705	2	48	0	0	0	T/T	C/C	G/A
NSCLC	case-0706	1	60	1	1	1	T/T	C/C	G/A
NSCLC	case-0707	2	53	0	0	1	C/C	G/C	G/A
NSCLC	case-0708	2	65	0	0	0	T/T	C/C	A/A
NSCLC	case-0709	2	45	0	0	0	C/C	G/C	A/A
NSCLC	case-0710	2	43	0	0	0	C/T	G/C	A/A
NSCLC	case-0711	2	66	0	0	0	T/T	G/C	A/A
NSCLC	case-0712	1	55	1	1	1	T/T	G/G	A/A
NSCLC	case-0713	2	45	0	0	0	T/T	G/C	A/A
NSCLC	case-0714	1	27	0	0	0	C/T	G/C	A/A
NSCLC	case-0715	2	32	0	0	0	T/T	C/C	A/A
NSCLC	case-0716	1	68	1	1	1	T/T	G/G	A/A
NSCLC	case-0717	2	61	0	0	0	C/C	G/C	A/A
NSCLC	case-0718	1	48	1	1	0	C/T	C/C	G/A
NSCLC	case-0719	2	42	0	0	1	T/T	C/C	A/A
NSCLC	case-0720	1	67	1	1	1	T/T	G/G	A/A
NSCLC	case-0721	2	59	0	0	0	T/T	G/G	G/A
NSCLC	case-0722	2	57	0	0	0	T/T	C/C	A/A
NSCLC	case-0723	1	51	0	1	1	C/T	C/C	A/A
NSCLC	case-0724	2	46	0	0	1	C/C	C/C	A/A
NSCLC	case-0725	2	40	0	0	0	C/T	G/C	A/A
NSCLC	case-0726	1	59	1	1	1	C/T	G/C	A/A
NSCLC	case-0727	1	71	0	0	0	C/T	C/C	A/A
NSCLC	case-0728	2	50	0	0	0	T/T	C/C	A/A
NSCLC	case-0729	2	55	0	0	0	C/T	G/C	G/A
NSCLC	case-0730	2	40	0	0	0	T/T	G/C	A/A
NSCLC	case-0731	1	66	0	1	1	C/C	C/C	A/A
NSCLC	case-0732	2	37	0	0	0	C/T	G/C	A/A
NSCLC	case-0733	2	61	0	0	0	C/T	G/C	A/A
NSCLC	case-0734	2	44	0	0	0	C/T	C/C	G/A
NSCLC	case-0735	1	59	0	0	0	T/T	G/C	A/A
NSCLC	case-0736	1	75	1	1	0	?	?	?
NSCLC	case-0737	1	53	1	1	0	T/T	G/C	A/A
NSCLC	case-0738	2	64	0	0	0	T/T	G/C	G/A
NSCLC	case-0739	2	54	0	0	0	T/T	C/C	G/A
NSCLC	case-0740	2	53	0	0	0	C/T	C/C	G/A
NSCLC	case-0741	2	32	0	0	0	C/T	C/C	A/A
NSCLC	case-0742	2	53	0	0	0	C/T	G/G	G/A
NSCLC	case-0743	1	62	1	1	0	T/T	C/C	A/A
NSCLC	case-0744	1	70	0	0	0	C/T	C/C	A/A
NSCLC	case-0745	2	48	0	0	1	C/T	C/C	A/A
NSCLC	case-0746	2	59	0	0	0	T/T	G/C	G/A
NSCLC	case-0747	2	54	0	0	0	C/C	G/C	A/A
NSCLC	case-0748	1	59	1	1	0	T/T	C/C	A/A
NSCLC	case-0749	2	62	0	0	0	C/C	G/C	A/A
NSCLC	case-0750	2	65	0	0	0	C/T	G/G	A/A

NSCLC	case-0751	2	40	0	0	0	T/T	C/C	A/A
NSCLC	case-0752	2	45	0	0	0	C/C	G/C	A/A
NSCLC	case-0753	1	64	1	0	0	T/T	G/C	A/A
NSCLC	case-0754	1	70	1	0	0	C/T	G/C	G/A
NSCLC	case-0755	1	52	1	0	0	T/T	G/G	A/A
NSCLC	case-0756	1	48	1	0	1	T/T	G/C	A/A
NSCLC	case-0757	1	64	1	0	1	C/C	C/C	A/A
NSCLC	case-0758	1	43	0	1	0	C/T	C/C	A/A
NSCLC	case-0759	1	65	1	1	0	C/T	C/C	G/A
NSCLC	case-0760	1	61	1	0	0	T/T	G/G	A/A
NSCLC	case-0761	2	54	0	0	0	T/T	C/C	A/A
NSCLC	case-0762	1	69	1	1	1	T/T	G/C	G/A
NSCLC	case-0763	2	60	0	0	0	C/T	G/C	G/A
NSCLC	case-0764	2	54	0	0	0	C/T	G/C	A/A
NSCLC	case-0765	2	65	0	0	1	C/C	G/G	A/A
NSCLC	case-0766	1	55	1	0	0	T/T	G/C	G/A
NSCLC	case-0767	2	63	1	1	0	C/T	G/C	A/A
NSCLC	case-0768	2	50	0	0	0	C/T	G/C	?
NSCLC	case-0769	1	61	0	0	0	C/T	G/C	A/A
NSCLC	case-0770	2	49	0	0	0	C/T	G/C	A/A
NSCLC	case-0771	2	52	0	0	0	T/T	G/C	A/A
NSCLC	case-0772	2	44	0	0	0	C/T	C/C	A/A
NSCLC	case-0773	2	60	0	0	0	C/T	G/C	G/A
NSCLC	case-0774	1	60	1	0	0	C/T	C/C	G/A
NSCLC	case-0775	2	53	0	0	0	C/T	C/C	A/A
NSCLC	case-0776	1	72	1	1	0	C/C	G/C	A/A
NSCLC	case-0777	2	71	0	0	1	C/T	C/C	G/A
NSCLC	case-0778	1	54	0	0	0	C/T	G/C	G/A
NSCLC	case-0779	1	59	1	1	0	C/C	C/C	A/A
NSCLC	case-0780	2	54	0	0	0	C/T	C/C	A/A
NSCLC	case-0781	1	55	1	1	1	C/T	G/C	G/A
NSCLC	case-0782	1	72	1	1	0	C/T	C/C	A/A
NSCLC	case-0783	1	45	0	0	0	C/T	G/G	A/A
NSCLC	case-0784	1	60	0	0	1	T/T	C/C	G/G
NSCLC	case-0785	2	28	0	0	0	C/C	C/C	G/A
NSCLC	case-0786	1	62	1	1	0	C/C	G/C	A/A
NSCLC	case-0787	1	57	1	1	0	T/T	C/C	G/A
NSCLC	case-0788	2	73	0	0	0	C/C	G/G	G/A
NSCLC	case-0789	2	65	0	0	0	T/T	C/C	A/A
NSCLC	case-0790	1	58	1	1	0	C/T	G/C	A/A
NSCLC	case-0791	2	70	0	0	0	T/T	C/C	G/A
NSCLC	case-0792	1	51	1	1	0	C/T	C/C	G/A
NSCLC	case-0793	2	62	0	0	0	C/T	C/C	A/A
NSCLC	case-0794	1	51	1	0	0	C/T	C/C	A/A
NSCLC	case-0795	2	44	0	0	0	T/T	G/C	G/A
NSCLC	case-0796	1	58	1	0	1	C/T	C/C	A/A
NSCLC	case-0797	1	60	0	1	0	C/T	C/C	A/A
NSCLC	case-0798	2	66	0	0	1	C/C	G/C	A/A
NSCLC	case-0799	1	63	0	0	0	T/T	C/C	A/A
NSCLC	case-0800	2	61	0	0	0	C/T	G/C	A/A
NSCLC	case-0801	2	65	0	0	0	C/T	G/C	A/A
NSCLC	case-0802	2	72	0	0	0	T/T	G/C	G/G
NSCLC	case-0803	1	74	1	0	1	T/T	G/C	A/A
NSCLC	case-0804	1	60	0	0	0	C/C	G/C	A/A

NSCLC	case-0805	2	47	0	0	1	C/T	G/C	A/A
NSCLC	case-0806	1	73	0	1	0	C/T	G/G	A/A
NSCLC	case-0807	1	52	1	1	1	C/C	G/C	A/A
NSCLC	case-0808	1	70	1	0	0	C/T	C/C	G/A
NSCLC	case-0809	2	63	0	0	0	C/T	C/C	G/A
NSCLC	case-0810	1	40	0	1	1	T/T	G/C	G/A
NSCLC	case-0811	2	46	0	0	0	C/C	G/C	A/A
NSCLC	case-0812	1	56	1	1	0	T/T	C/C	A/A
NSCLC	case-0813	1	53	1	1	1	C/T	C/C	A/A
NSCLC	case-0814	1	76	1	1	0	T/T	C/C	A/A
NSCLC	case-0815	1	53	1	1	0	C/T	G/G	A/A
NSCLC	case-0816	1	29	0	0	1	T/T	C/C	A/A
NSCLC	case-0817	1	50	1	0	0	C/T	G/C	A/A
NSCLC	case-0818	2	54	0	0	0	C/T	G/C	A/A
NSCLC	case-0819	1	57	0	1	0	C/T	C/C	A/A
NSCLC	case-0820	1	38	1	1	1	C/T	G/C	A/A
NSCLC	case-0821	1	30	0	0	0	C/T	C/C	G/G
NSCLC	case-0822	2	51	0	0	1	C/T	G/C	A/A
NSCLC	case-0823	2	49	0	0	0	C/C	G/C	G/A
NSCLC	case-0824	1	66	0	1	1	C/T	C/C	G/A
NSCLC	case-0825	1	64	1	0	0	C/C	C/C	G/A
NSCLC	case-0826	2	45	0	0	0	C/T	G/C	A/A
NSCLC	case-0827	2	54	0	0	1	T/T	C/C	A/A
NSCLC	case-0828	2	50	0	0	0	T/T	C/C	G/A
NSCLC	case-0829	2	40	0	0	0	T/T	G/C	A/A
NSCLC	case-0830	1	71	1	0	0	C/T	C/C	A/A
NSCLC	case-0831	1	57	1	1	1	C/C	C/C	G/A
NSCLC	case-0832	2	65	0	0	0	C/T	G/C	A/A
NSCLC	case-0833	2	62	0	0	1	C/C	C/C	A/A
NSCLC	case-0834	2	68	0	0	0	T/T	G/G	G/A
NSCLC	case-0835	1	48	0	0	1	C/C	G/G	G/A
NSCLC	case-0836	1	60	1	0	1	C/T	G/C	G/A
NSCLC	case-0837	1	57	0	1	1	T/T	G/C	A/A
NSCLC	case-0838	1	59	0	1	1	C/T	G/G	A/A
NSCLC	case-0839	1	76	1	0	0	C/C	G/G	A/A
NSCLC	case-0840	1	73	1	0	0	T/T	G/C	G/A
NSCLC	case-0841	2	55	0	0	0	C/C	G/C	A/A
NSCLC	case-0842	2	46	0	0	1	C/T	G/C	G/A
NSCLC	case-0843	1	59	1	0	0	C/T	C/C	A/A
NSCLC	case-0844	1	59	1	1	0	C/C	G/C	A/A
NSCLC	case-0845	1	51	1	1	1	C/T	G/G	A/A
NSCLC	case-0846	1	51	1	1	0	C/C	G/G	G/A
NSCLC	case-0847	2	67	0	1	0	C/C	G/G	G/A
NSCLC	case-0848	2	56	0	0	0	T/T	G/C	A/A
NSCLC	case-0849	2	60	0	0	0	C/T	C/C	A/A
NSCLC	case-0850	1	62	1	0	1	C/T	G/C	G/A
NSCLC	case-0851	2	68	0	0	0	C/T	G/C	A/A
NSCLC	case-0852	1	49	1	1	0	T/T	G/C	A/A
NSCLC	case-0853	2	51	0	0	0	C/C	G/G	G/A
NSCLC	case-0854	2	58	0	0	0	T/T	C/C	A/A
NSCLC	case-0855	2	53	0	0	1	T/T	G/C	A/A
NSCLC	case-0856	2	53	0	0	1	T/T	G/C	A/A
NSCLC	case-0857	2	58	0	0	0	C/C	G/C	G/A
NSCLC	case-0858	1	66	1	1	0	T/T	G/C	A/A

NSCLC	case-0859	1	52	1	0	0	C/T	G/C	A/A
NSCLC	case-0860	1	78	0	0	0	C/T	C/C	A/A
NSCLC	case-0861	1	57	1	0	0	T/T	G/C	A/A
NSCLC	case-0862	1	54	1	1	1	T/T	G/C	A/A
NSCLC	case-0863	1	38	0	0	1	T/T	C/C	A/A
NSCLC	case-0864	2	67	0	0	1	C/T	C/C	G/A
NSCLC	case-0865	2	51	0	0	1	C/C	C/C	A/A
NSCLC	case-0866	2	46	0	0	1	C/T	G/C	G/A
NSCLC	case-0867	1	69	0	0	0	C/T	C/C	A/A
NSCLC	case-0868	1	27	0	0	1	C/T	G/C	A/A
NSCLC	case-0869	2	51	0	0	0	C/C	G/G	A/A
NSCLC	case-0870	1	28	1	1	0	T/T	G/C	A/A
NSCLC	case-0871	1	57	1	1	0	T/T	C/C	A/A
NSCLC	case-0872	1	62	1	1	1	C/T	G/G	A/A
NSCLC	case-0873	2	59	0	0	0	C/T	G/C	A/A
NSCLC	case-0874	1	40	0	1	0	C/T	G/G	A/A
NSCLC	case-0875	2	40	0	0	0	T/T	G/G	A/A
NSCLC	case-0876	1	56	0	0	0	C/C	G/G	A/A
NSCLC	case-0877	2	49	0	0	0	C/T	G/C	G/A
NSCLC	case-0878	1	69	1	1	0	T/T	G/C	A/A
NSCLC	case-0879	2	53	0	0	0	C/T	C/C	A/A
NSCLC	case-0880	2	51	0	0	0	C/T	G/C	A/A
NSCLC	case-0881	1	55	1	1	0	C/T	G/C	G/G
NSCLC	case-0882	2	48	0	0	1	C/T	G/C	A/A
NSCLC	case-0883	1	64	1	0	1	T/T	G/C	A/A
NSCLC	case-0884	1	62	1	1	0	T/T	C/C	G/A
NSCLC	case-0885	1	54	1	1	0	C/T	G/C	A/A
NSCLC	case-0886	1	61	0	0	0	C/C	G/C	A/A
NSCLC	case-0887	2	43	0	0	0	T/T	G/C	A/A
NSCLC	case-0888	1	43	1	1	1	C/T	G/C	A/A
NSCLC	case-0889	2	63	0	0	0	C/T	G/C	G/A
NSCLC	case-0890	1	65	1	1	0	C/T	C/C	G/G
NSCLC	case-0891	1	63	0	0	1	C/T	G/C	A/A
NSCLC	case-0892	2	62	0	0	0	C/T	C/C	A/A
NSCLC	case-0893	1	82	1	0	0	T/T	C/C	A/A
NSCLC	case-0894	2	50	0	0	0	C/T	G/C	G/A
NSCLC	case-0895	1	67	1	1	0	C/C	G/C	G/A
NSCLC	case-0896	2	68	0	0	0	T/T	C/C	A/A
NSCLC	case-0897	2	53	0	0	0	C/C	G/G	A/A
NSCLC	case-0898	1	66	1	0	0	T/T	G/C	A/A
NSCLC	case-0899	2	52	0	0	0	T/T	C/C	G/A
NSCLC	case-0900	2	60	0	0	0	C/T	C/C	A/A
NSCLC	case-0901	1	48	1	1	0	T/T	C/C	G/A
NSCLC	case-0902	1	72	1	1	0	C/T	G/C	A/A
NSCLC	case-0903	2	60	0	0	1	C/T	G/C	G/A
NSCLC	case-0904	1	47	0	0	0	T/T	C/C	A/A
NSCLC	case-0905	1	66	1	1	0	C/T	G/C	A/A
NSCLC	case-0906	1	57	1	1	0	C/T	G/C	A/A
NSCLC	case-0907	1	65	1	1	0	C/T	G/C	G/A
NSCLC	case-0908	1	52	1	1	1	C/T	G/G	A/A
NSCLC	case-0909	1	66	1	1	0	C/C	G/G	G/A
NSCLC	case-0910	1	79	1	1	1	C/T	G/G	A/A
NSCLC	case-0911	1	63	1	0	1	T/T	C/C	A/A
NSCLC	case-0912	1	58	1	0	0	C/T	G/C	A/A

NSCLC	case-0913	1	73	1	0	0	T/T	G/C	A/A
NSCLC	case-0914	2	66	0	0	0	T/T	G/C	A/A
NSCLC	case-0915	1	55	1	0	0	C/T	G/C	G/G
NSCLC	case-0916	2	71	0	0	0	T/T	G/C	A/A
NSCLC	case-0917	2	55	0	0	0	C/C	C/C	A/A
NSCLC	case-0918	2	57	0	0	0	C/C	G/C	A/A
NSCLC	case-0919	1	55	1	0	0	T/T	C/C	A/A
NSCLC	case-0920	2	62	0	0	0	C/C	G/C	A/A
NSCLC	case-0921	1	77	1	0	0	T/T	G/G	G/A
NSCLC	case-0922	1	59	1	1	0	C/T	G/C	G/G
NSCLC	case-0923	2	52	0	0	0	C/T	C/C	A/A
NSCLC	case-0924	2	59	0	0	0	C/T	G/G	A/A
NSCLC	case-0925	1	62	1	1	0	C/T	G/C	A/A
NSCLC	case-0926	1	59	1	1	0	C/T	C/C	A/A
NSCLC	case-0927	1	53	1	1	0	C/T	G/C	A/A
NSCLC	case-0928	1	66	1	0	1	C/T	G/G	A/A
NSCLC	case-0929	2	63	0	0	0	T/T	G/G	A/A
NSCLC	case-0930	2	56	0	0	0	T/T	G/C	A/A
NSCLC	case-0931	1	70	0	0	0	C/T	G/C	G/A
NSCLC	case-0932	1	64	0	0	0	C/T	G/C	G/A
NSCLC	case-0933	1	52	0	0	1	C/T	C/C	A/A
NSCLC	case-0934	1	74	1	1	1	T/T	C/C	A/A
NSCLC	case-0935	1	58	1	1	0	C/T	C/C	A/A
NSCLC	case-0936	2	64	1	1	0	C/T	G/G	A/A
NSCLC	case-0937	2	55	0	0	0	C/T	G/C	G/A
NSCLC	case-0938	1	53	1	1	1	T/T	G/C	A/A
NSCLC	case-0939	2	54	0	0	0	C/T	C/C	A/A
NSCLC	case-0940	1	51	1	1	0	C/C	C/C	G/A
NSCLC	case-0941	1	55	1	1	1	C/T	C/C	G/A
NSCLC	case-0942	1	64	1	1	1	C/T	G/C	A/A
NSCLC	case-0943	1	52	1	1	1	C/T	G/C	A/A
NSCLC	case-0944	1	52	0	0	0	T/T	G/G	A/A
NSCLC	case-0945	1	59	0	0	0	C/T	G/C	A/A
NSCLC	case-0946	1	65	0	1	0	T/T	C/C	A/A
NSCLC	case-0947	2	76	0	0	0	T/T	G/C	A/A
NSCLC	case-0948	1	70	0	0	1	C/T	G/C	G/G
NSCLC	case-0949	2	51	0	0	1	C/T	G/C	G/A
NSCLC	case-0950	2	59	0	0	0	C/T	C/C	A/A
NSCLC	case-0951	2	55	0	0	1	C/T	G/C	A/A
NSCLC	case-0952	1	56	0	0	1	T/T	C/C	G/A
NSCLC	case-0953	1	59	1	1	0	C/T	C/C	A/A
NSCLC	case-0954	1	52	1	1	0	C/T	C/C	A/A
NSCLC	case-0955	2	52	0	0	1	C/T	C/C	A/A
NSCLC	case-0956	2	54	0	0	0	T/T	G/C	G/A
NSCLC	case-0957	1	39	1	0	0	C/T	C/C	A/A
NSCLC	case-0958	2	55	0	0	0	C/T	G/G	G/A
NSCLC	case-0959	1	52	1	1	0	T/T	G/G	A/A
NSCLC	case-0960	1	66	1	1	0	C/T	G/C	A/A
NSCLC	case-0961	2	54	0	0	1	C/T	G/C	A/A
NSCLC	case-0962	2	75	0	0	1	T/T	C/C	A/A
NSCLC	case-0963	2	71	0	0	0	T/T	C/C	A/A
NSCLC	case-0964	2	56	0	0	0	C/C	G/C	G/A
NSCLC	case-0965	2	71	0	0	1	C/T	C/C	A/A
NSCLC	case-0966	1	69	1	0	0	C/C	G/C	A/A

NSCLC	case-0967	1	60	1	0	1	T/T	C/C	A/A
NSCLC	case-0968	2	71	0	0	0	C/C	G/C	A/A
NSCLC	case-0969	1	56	1	0	0	T/T	C/C	A/A
NSCLC	case-0970	2	60	0	0	0	C/T	C/C	A/A
NSCLC	case-0971	1	66	1	1	0	?	?	?
NSCLC	case-0972	1	59	1	1	0	T/T	C/C	A/A
NSCLC	case-0973	2	53	0	0	1	T/T	G/C	A/A
NSCLC	case-0974	2	57	0	0	0	C/C	G/C	G/A
NSCLC	case-0975	1	57	1	0	1	C/T	G/C	A/A
NSCLC	case-0976	1	68	0	0	0	T/T	G/C	A/A
NSCLC	case-0977	1	69	1	1	1	T/T	G/G	A/A
NSCLC	case-0978	2	67	0	0	0	T/T	G/G	A/A
NSCLC	case-0979	2	53	0	0	0	C/C	C/C	A/A
NSCLC	case-0980	2	52	0	0	0	C/T	G/G	A/A
NSCLC	case-0981	2	62	0	0	1	C/T	G/C	A/A
NSCLC	case-0982	1	64	1	1	0	T/T	G/G	A/A
NSCLC	case-0983	2	65	0	0	0	C/T	G/C	A/A
NSCLC	case-0984	1	49	1	1	0	T/T	C/C	A/A
NSCLC	case-0985	2	59	0	0	1	C/T	C/C	G/A
NSCLC	case-0986	2	46	0	0	0	C/T	G/C	A/A
NSCLC	case-0987	2	67	0	0	0	T/T	G/G	A/A
NSCLC	case-0988	1	55	0	0	0	C/T	C/C	A/A
NSCLC	case-0989	1	76	0	0	0	T/T	C/C	G/A
NSCLC	case-0990	1	48	0	0	0	C/T	G/C	A/A
NSCLC	case-0991	1	60	0	0	0	T/T	G/C	G/A
NSCLC	case-0992	1	67	1	1	0	C/C	G/G	A/A
NSCLC	case-0993	2	63	0	0	0	C/T	G/C	G/A
NSCLC	case-0994	1	60	0	1	1	C/C	C/C	G/A
NSCLC	case-0995	1	59	0	0	1	C/T	C/C	G/A
NSCLC	case-0996	2	57	0	0	0	C/T	G/C	A/A
NSCLC	case-0997	2	56	0	0	1	C/C	G/C	A/A
NSCLC	case-0998	1	60	0	0	0	C/T	C/C	A/A
NSCLC	case-0999	1	64	0	0	0	C/T	G/G	A/A
NSCLC	case-1000	2	72	0	0	0	T/T	C/C	A/A
NSCLC	case-1001	2	73	0	0	1	T/T	G/C	A/A
NSCLC	case-1002	2	59	0	0	1	C/T	G/C	G/A
NSCLC	case-1003	2	54	0	0	1	C/T	C/C	A/A
NSCLC	case-1004	1	62	1	1	0	C/C	G/C	G/A
NSCLC	case-1005	2	62	0	0	0	T/T	G/G	A/A
NSCLC	case-1006	1	75	0	0	1	T/T	C/C	A/A
NSCLC	case-1007	2	72	0	0	0	C/C	G/C	A/A
NSCLC	case-1008	1	46	1	1	1	C/C	G/G	G/A
NSCLC	case-1009	1	58	1	0	1	C/T	G/G	G/A
NSCLC	case-1010	2	66	0	0	1	C/T	C/C	A/A
NSCLC	case-1011	1	56	1	0	0	C/T	G/C	G/G
NSCLC	case-1012	1	62	1	1	0	C/C	G/C	A/A
NSCLC	case-1013	2	64	0	0	0	T/T	G/G	A/A
NSCLC	case-1014	2	53	0	0	1	C/C	G/C	A/A
NSCLC	case-1015	1	62	0	0	1	C/T	G/C	G/A
NSCLC	case-1016	1	63	0	0	1	C/T	G/C	G/G
NSCLC	case-1017	2	60	0	0	1	C/T	C/C	A/A
NSCLC	case-1018	1	57	0	0	0	C/T	G/G	G/G
NSCLC	case-1019	1	60	1	0	0	C/T	G/C	A/A
NSCLC	case-1020	2	67	0	0	1	T/T	G/C	A/A

NSCLC	case-1021	2	54	0	0	1	C/C	G/C	G/A
NSCLC	case-1022	2	79	0	0	0	C/T	G/G	G/A
NSCLC	case-1023	1	63	1	0	1	C/T	G/C	A/A
NSCLC	case-1024	2	69	0	0	1	C/T	G/G	A/A
NSCLC	case-1025	1	69	1	1	0	C/T	C/C	G/A
NSCLC	case-1026	1	57	1	1	0	C/C	G/C	A/A
NSCLC	case-1027	1	45	0	0	0	C/C	G/C	A/A
NSCLC	case-1028	2	69	0	0	1	T/T	C/C	A/A
NSCLC	case-1029	1	62	1	0	0	C/C	G/C	A/A
NSCLC	case-1030	1	61	0	0	1	C/C	G/C	G/A
NSCLC	case-1031	1	55	1	0	0	C/C	C/C	A/A
NSCLC	case-1032	2	55	0	0	1	C/T	C/C	G/A
NSCLC	case-1033	1	74	0	0	0	C/C	G/C	G/A
NSCLC	case-1034	2	50	0	0	1	T/T	C/C	A/A
NSCLC	case-1035	2	65	0	0	0	C/T	C/C	A/A
NSCLC	case-1036	2	62	0	0	0	C/T	C/C	A/A
NSCLC	case-1037	1	60	1	0	1	T/T	C/C	G/A
NSCLC	case-1038	1	67	1	0	1	T/T	C/C	G/A
NSCLC	case-1039	2	75	0	0	1	C/T	C/C	A/A
NSCLC	case-1040	1	52	1	0	0	T/T	C/C	G/A
NSCLC	case-1041	1	63	0	0	1	C/T	G/C	A/A
NSCLC	case-1042	2	66	0	0	0	C/C	C/C	A/A
NSCLC	case-1043	1	66	1	1	0	C/T	C/C	A/A
NSCLC	case-1044	2	54	0	0	0	C/C	G/C	A/A
NSCLC	case-1045	1	60	0	0	0	T/T	G/C	A/A
NSCLC	case-1046	1	75	1	1	0	C/C	G/G	A/A
NSCLC	case-1047	1	49	0	0	1	T/T	G/G	A/A
NSCLC	case-1048	2	60	0	0	1	C/T	G/C	A/A
NSCLC	case-1049	1	62	0	0	0	C/C	G/C	A/A
NSCLC	case-1050	1	71	1	1	1	T/T	C/C	A/A
NSCLC	case-1051	2	53	0	0	0	C/T	C/C	A/A
NSCLC	case-1052	2	58	0	0	0	C/T	C/C	G/A
NSCLC	case-1053	2	58	0	0	0	C/T	G/C	G/A
NSCLC	case-1054	2	64	0	0	0	C/T	G/G	G/G
NSCLC	case-1055	1	55	0	0	1	C/T	G/C	A/A
NSCLC	case-1056	2	54	0	0	1	C/C	G/C	G/A
NSCLC	case-1057	1	72	0	0	0	C/C	G/G	A/A
NSCLC	case-1058	1	62	0	0	0	C/T	G/G	A/A
NSCLC	case-1059	1	64	0	0	1	C/T	G/G	A/A
NSCLC	case-1060	2	64	0	0	0	C/T	G/G	G/A
NSCLC	case-1061	1	65	0	0	0	C/T	G/G	A/A
NSCLC	case-1062	1	68	1	1	0	C/T	C/C	A/A
NSCLC	case-1063	1	66	1	0	0	C/C	G/C	G/A
NSCLC	case-1064	2	74	0	0	0	C/T	G/C	G/A
NSCLC	case-1065	1	68	0	0	0	C/C	C/C	A/A
NSCLC	case-1066	1	49	0	0	0	T/T	G/C	G/G
NSCLC	case-1067	2	49	0	0	0	T/T	G/C	A/A
NSCLC	case-1068	1	69	1	0	0	C/C	G/G	A/A
NSCLC	case-1069	2	73	0	0	0	C/C	G/C	A/A
NSCLC	case-1070	1	50	1	0	1	C/T	G/G	G/A
NSCLC	case-1071	1	66	0	0	1	C/T	G/G	A/A
NSCLC	case-1072	1	53	1	0	0	T/T	G/C	A/A
NSCLC	case-1073	1	69	0	0	0	C/T	G/C	G/A
NSCLC	case-1074	1	70	1	0	0	C/T	G/C	A/A

NSCLC	case-1075	2	60	0	0	1	C/T	C/C	A/A
NSCLC	case-1076	1	63	1	1	1	C/T	C/C	A/A
NSCLC	case-1077	1	58	1	1	0	C/T	G/C	A/A
NSCLC	case-1078	1	69	0	0	1	C/T	G/G	A/A
NSCLC	case-1079	2	70	0	0	0	C/C	G/C	G/G
NSCLC	case-1080	2	46	0	0	1	T/T	C/C	G/A
NSCLC	case-1081	2	49	0	0	0	C/T	G/C	A/A
NSCLC	case-1082	2	59	0	0	0	C/T	C/C	G/A
NSCLC	case-1083	1	63	0	0	1	C/T	C/C	G/A
NSCLC	case-1084	1	67	1	1	0	C/C	C/C	G/A
NSCLC	case-1085	1	58	0	0	1	C/C	G/C	A/A
NSCLC	case-1086	1	62	1	0	1	C/T	G/C	G/A
NSCLC	case-1087	2	63	0	0	1	T/T	G/C	A/A
NSCLC	case-1088	1	37	1	1	0	T/T	C/C	G/A
NSCLC	case-1089	1	50	1	1	0	C/C	C/C	A/A
NSCLC	case-1090	2	44	0	0	0	C/C	G/C	A/A
NSCLC	case-1091	2	55	0	0	0	C/C	G/G	A/A
NSCLC	case-1092	1	57	1	1	0	C/T	G/C	A/A
NSCLC	case-1093	1	51	1	0	1	C/T	G/C	A/A
NSCLC	case-1094	1	53	0	0	1	C/T	G/C	A/A
NSCLC	case-1095	1	60	1	0	0	C/T	G/G	A/A
NSCLC	case-1096	2	37	0	0	0	T/T	G/G	G/G
NSCLC	case-1097	2	66	0	0	0	T/T	G/G	A/A
NSCLC	case-1098	1	66	0	0	0	C/T	C/C	G/A
NSCLC	case-1099	1	55	0	0	0	C/T	C/C	G/A
NSCLC	case-1100	1	51	1	1	0	C/C	G/C	A/A
NSCLC	case-1101	2	52	0	0	0	C/C	G/G	G/A
NSCLC	case-1102	1	52	1	1	0	T/T	C/C	A/A
NSCLC	case-1103	2	46	0	0	0	T/T	G/G	G/A
NSCLC	case-1104	2	77	0	0	1	C/T	G/G	A/A
NSCLC	case-1105	2	47	0	0	0	C/C	C/C	G/A
NSCLC	case-1106	2	51	0	0	0	C/T	G/C	A/A
NSCLC	case-1107	1	42	0	0	0	C/C	G/C	A/A
NSCLC	case-1108	2	55	0	0	1	T/T	C/C	A/A
NSCLC	case-1109	2	65	0	0	0	C/T	G/C	A/A
NSCLC	case-1110	1	49	0	0	0	T/T	G/C	A/A
NSCLC	case-1111	2	48	0	0	0	C/T	G/G	A/A
NSCLC	case-1112	1	55	1	1	0	C/T	G/C	A/A
NSCLC	case-1113	1	77	0	0	0	C/C	G/C	G/A
NSCLC	case-1114	1	76	1	1	1	C/T	C/C	A/A
NSCLC	case-1115	1	57	0	1	1	T/T	C/C	A/A
NSCLC	case-1116	1	63	1	1	1	C/T	G/G	A/A
NSCLC	case-1117	1	53	0	0	1	C/T	G/C	A/A
NSCLC	case-1118	1	51	1	0	1	C/T	G/C	A/A
NSCLC	case-1119	2	48	0	0	0	T/T	G/C	A/A
NSCLC	case-1120	1	70	1	0	0	C/T	G/C	A/A
NSCLC	case-1121	2	44	0	0	0	C/C	G/G	A/A
NSCLC	case-1122	1	70	0	0	0	C/T	C/C	A/A
NSCLC	case-1123	2	58	0	0	0	C/C	G/C	G/A
NSCLC	case-1124	1	55	0	0	0	C/T	C/C	G/A
NSCLC	case-1125	2	61	0	0	0	C/T	G/C	G/A
NSCLC	case-1126	1	48	1	1	1	C/C	C/C	G/A
NSCLC	case-1127	1	43	0	0	0	T/T	G/G	?
NSCLC	case-1128	1	59	1	0	0	T/T	G/C	A/A

NSCLC	case-1129	1	46	1	1	1	C/C	G/C	A/A
NSCLC	case-1130	2	61	0	0	0	C/C	G/C	G/A
NSCLC	case-1131	1	68	1	0	1	C/T	C/C	A/A
NSCLC	case-1132	1	60	1	1	1	T/T	G/C	A/A
NSCLC	case-1133	2	68	0	0	1	T/T	G/G	A/A
NSCLC	case-1134	2	65	0	0	0	C/T	C/C	G/G
NSCLC	case-1135	2	62	0	0	1	C/T	G/G	G/A
NSCLC	case-1136	1	70	1	1	0	?	?	?
NSCLC	case-1137	1	70	1	0	0	C/T	G/C	A/A
NSCLC	case-1138	2	39	0	0	0	C/T	C/C	G/A
NSCLC	case-1139	1	55	1	1	0	C/C	G/C	A/A
NSCLC	case-1140	2	53	0	0	0	C/T	C/C	A/A
NSCLC	case-1141	1	57	0	0	1	T/T	C/C	A/A
NSCLC	case-1142	2	68	0	0	1	C/C	C/C	A/A
NSCLC	case-1143	2	57	0	0	0	C/T	C/C	A/A
NSCLC	case-1144	1	70	1	1	0	C/T	C/C	G/A
NSCLC	case-1145	1	64	1	1	0	T/T	G/C	A/A
NSCLC	case-1146	1	65	1	1	0	T/T	C/C	A/A
NSCLC	case-1147	1	57	1	0	0	C/T	C/C	?
NSCLC	case-1148	2	45	0	0	0	C/T	G/C	?
NSCLC	case-1149	2	33	0	0	0	C/T	C/C	G/A
NSCLC	case-1150	1	29	0	0	0	C/T	C/C	A/A
NSCLC	case-1151	1	54	1	0	0	C/T	G/G	A/A
NSCLC	case-1152	2	66	0	0	0	C/T	C/C	A/A
NSCLC	case-1153	2	82	0	0	0	?	?	?
NSCLC	case-1154	1	62	0	0	1	C/T	C/C	A/A
NSCLC	case-1155	2	50	0	0	0	?	?	?
NSCLC	case-1156	1	51	1	0	0	C/C	G/C	G/G
NSCLC	case-1157	1	72	1	1	0	C/T	G/C	G/A
NSCLC	case-1158	2	55	0	0	0	C/T	C/C	G/A
NSCLC	case-1159	1	71	1	1	0	C/T	C/C	A/A
NSCLC	case-1160	1	51	1	1	1	C/T	C/C	A/A
NSCLC	case-1161	2	66	0	0	1	C/T	G/C	A/A
NSCLC	case-1162	2	48	0	0	0	T/T	G/G	A/A
NSCLC	case-1163	2	71	0	0	1	T/T	C/C	A/A
NSCLC	case-1164	2	47	0	0	0	C/T	C/C	A/A
NSCLC	case-1165	1	51	1	0	1	C/T	C/C	A/A
NSCLC	case-1166	2	45	0	0	1	C/T	C/C	A/A
NSCLC	case-1167	1	57	0	0	0	C/T	G/C	A/A
NSCLC	case-1168	2	54	0	0	0	C/T	C/C	A/A
NSCLC	case-1169	2	61	0	0	0	T/T	G/C	G/A
NSCLC	case-1170	1	73	1	1	0	C/T	C/C	G/A
NSCLC	case-1171	1	29	0	0	0	T/T	G/C	A/A
NSCLC	case-1172	2	62	0	0	0	C/T	C/C	A/A
NSCLC	case-1173	2	29	0	0	0	T/T	G/C	A/A
NSCLC	case-1174	2	54	0	0	1	C/C	G/C	G/A
NSCLC	case-1175	2	73	0	0	0	T/T	G/C	A/A
NSCLC	case-1176	2	54	0	0	0	T/T	G/C	G/G
NSCLC	case-1177	1	53	1	0	0	C/T	G/G	G/A
NSCLC	case-1178	1	62	1	0	0	T/T	C/C	A/A
NSCLC	case-1179	2	67	0	0	0	C/C	G/C	A/A
NSCLC	case-1180	2	45	0	0	0	T/T	C/C	A/A
NSCLC	case-1181	1	65	1	1	0	C/C	C/C	A/A
NSCLC	case-1182	1	54	1	0	0	C/T	G/C	G/A

NSCLC case-1183	1	72	1	0	0	T/T	G/C	G/A
NSCLC case-1184	1	51	1	1	0	T/T	G/C	A/A
NSCLC case-1185	1	60	0	0	0	C/T	C/C	G/G
NSCLC case-1186	1	52	0	0	0	C/T	C/C	G/A
NSCLC case-1187	2	60	1	0	0	C/T	G/C	G/A
NSCLC case-1188	1	71	1	0	0	C/T	C/C	G/A
NSCLC case-1189	1	64	1	1	1	C/T	C/C	A/A
NSCLC case-1190	2	67	0	0	1	C/T	G/C	A/A
NSCLC case-1191	2	63	0	0	0	T/T	C/C	G/A
NSCLC case-1192	2	29	0	0	0	T/T	G/C	A/A
NSCLC case-1193	2	56	0	0	0	C/C	G/C	G/A
Control-0001	1	55	0	0	1	C/T	G/G	A/A
Control-0002	2	53	0	0	1	T/T	G/C	A/A
Control-0003	1	59	0	0	1	T/T	G/C	A/A
Control-0004	2	54	0	0	0	C/T	G/C	G/A
Control-0005	2	70	0	0	1	C/T	C/C	G/A
Control-0006	2	56	0	0	0	C/C	G/G	A/A
Control-0007	1	51	0	0	1	T/T	C/C	G/A
Control-0008	2	61	0	0	0	C/T	G/C	A/A
Control-0009	1	57	1	0	1	T/T	G/C	A/A
Control-0010	1	53	1	0	0	C/C	G/C	G/A
Control-0011	1	56	0	1	1	C/T	G/C	A/A
Control-0012	1	64	1	1	1	T/T	G/G	A/A
Control-0013	1	62	0	0	1	C/T	C/C	A/A
Control-0014	1	66	0	0	1	C/T	G/G	A/A
Control-0015	1	29	1	0	1	C/T	G/G	G/A
Control-0016	1	38	0	0	1	C/T	C/C	A/A
Control-0017	1	60	1	0	1	T/T	C/C	A/A
Control-0018	2	53	0	0	1	T/T	C/C	A/A
Control-0019	1	58	0	0	0	T/T	G/C	A/A
Control-0020	2	52	0	0	0	T/T	G/C	A/A
Control-0021	2	38	0	0	0	T/T	G/C	A/A
Control-0022	1	28	0	0	0	T/T	G/C	G/A
Control-0023	2	65	0	0	1	C/T	C/C	A/A
Control-0024	2	52	0	0	0	T/T	C/C	A/A
Control-0025	2	46	0	0	0	C/T	G/G	G/A
Control-0026	1	56	0	0	1	C/T	G/C	A/A
Control-0027	2	68	0	0	1	T/T	C/C	A/A
Control-0028	1	59	0	0	0	C/T	G/C	A/A
Control-0029	1	62	0	0	1	C/T	C/C	A/A
Control-0030	1	71	0	0	0	C/C	G/C	A/A
Control-0031	1	60	1	1	0	C/T	G/C	A/A
Control-0032	1	56	1	0	0	C/T	C/C	G/A
Control-0033	1	60	0	0	0	C/T	G/C	A/A
Control-0034	2	72	0	0	0	C/T	G/C	A/A
Control-0035	2	61	0	0	0	C/C	G/C	A/A
Control-0036	2	66	0	0	0	C/T	G/C	A/A
Control-0037	1	61	1	0	0	C/C	C/C	A/A
Control-0038	2	54	0	0	0	C/T	G/C	G/A
Control-0039	2	56	0	0	0	C/T	G/G	A/A
Control-0040	2	71	0	0	1	T/T	G/C	A/A
Control-0041	1	72	0	0	0	C/T	G/C	A/A
Control-0042	1	46	0	0	0	C/T	G/G	A/A
Control-0043	1	62	1	0	0	C/T	C/C	A/A

Control-0044	2	59	0	0	0	C/T	C/C	A/A
Control-0045	2	60	0	0	1	C/T	G/G	A/A
Control-0046	2	52	0	0	1	C/C	C/C	A/A
Control-0047	1	56	1	0	0	T/T	G/C	A/A
Control-0048	1	59	1	0	0	T/T	G/C	G/A
Control-0049	1	61	0	0	1	C/T	C/C	A/A
Control-0050	1	63	0	0	1	T/T	C/C	A/A
Control-0051	1	72	0	0	0	C/T	G/C	G/A
Control-0052	1	64	0	1	0	C/T	C/C	A/A
Control-0053	2	67	0	0	1	C/C	C/C	A/A
Control-0054	1	58	1	0	0	C/T	C/C	A/A
Control-0055	2	61	0	0	0	C/T	G/C	A/A
Control-0056	1	41	1	0	1	C/T	G/C	A/A
Control-0057	2	69	0	0	0	C/C	G/G	A/A
Control-0058	1	62	0	0	0	C/C	G/C	A/A
Control-0059	1	81	0	0	0	C/T	C/C	A/A
Control-0060	1	57	0	0	0	T/T	C/C	G/A
Control-0061	2	51	0	0	1	C/T	G/C	A/A
Control-0062	1	55	1	0	0	C/C	G/C	A/A
Control-0063	2	37	0	0	0	T/T	C/C	A/A
Control-0064	1	65	1	1	0	C/T	G/C	A/A
Control-0065	1	57	0	0	1	C/T	G/C	G/A
Control-0066	1	80	0	0	0	C/C	G/C	A/A
Control-0067	2	49	0	0	0	C/C	G/C	A/A
Control-0068	2	46	0	0	0	C/T	G/G	A/A
Control-0069	2	64	0	0	1	C/C	G/C	G/A
Control-0070	2	65	0	0	0	C/C	G/C	A/A
Control-0071	1	78	0	0	1	C/T	C/C	A/A
Control-0072	1	43	0	0	0	C/C	G/G	A/A
Control-0073	1	64	0	0	0	C/C	C/C	G/A
Control-0074	2	56	0	0	1	C/T	G/C	G/A
Control-0075	2	52	0	0	1	T/T	C/C	A/A
Control-0076	2	57	0	0	0	C/T	C/C	A/A
Control-0077	2	67	0	0	0	C/T	G/G	G/A
Control-0078	2	53	0	0	0	C/T	G/C	A/A
Control-0079	1	48	1	0	1	C/T	C/C	A/A
Control-0080	1	57	1	0	0	C/C	C/C	A/A
Control-0081	1	62	1	0	1	C/T	G/C	A/A
Control-0082	2	71	0	0	0	C/C	G/C	A/A
Control-0083	2	53	0	0	0	C/T	G/C	A/A
Control-0084	2	49	0	0	1	C/T	G/C	G/A
Control-0085	2	61	0	0	0	C/T	G/C	A/A
Control-0086	2	62	0	0	1	T/T	C/C	A/A
Control-0087	1	53	1	0	1	T/T	G/C	A/A
Control-0088	1	62	0	0	1	C/C	G/C	G/A
Control-0089	1	63	0	0	0	C/T	G/C	A/A
Control-0090	1	33	0	0	0	C/T	G/G	A/A
Control-0091	1	81	0	0	1	C/T	G/G	A/A
Control-0092	2	59	0	0	1	C/T	C/C	A/A
Control-0093	2	70	0	0	0	C/T	G/C	G/A
Control-0094	2	56	0	0	0	C/T	C/C	A/A
Control-0095	1	58	0	0	0	C/C	G/C	A/A
Control-0096	1	60	1	0	0	C/T	C/C	A/A
Control-0097	1	59	0	0	1	C/C	C/C	A/A

Control-0098	1	76	1	0	0	C/C	C/C	G/G
Control-0099	1	52	0	0	0	C/T	G/G	A/A
Control-0100	2	34	0	0	0	C/C	C/C	A/A
Control-0101	1	60	1	0	1	C/C	C/C	A/A
Control-0102	1	76	1	0	0	C/T	G/C	A/A
Control-0103	2	50	0	0	1	T/T	G/C	G/A
Control-0104	1	57	0	0	0	C/C	G/G	A/A
Control-0105	1	51	0	0	0	C/C	C/C	G/G
Control-0106	1	60	0	0	0	T/T	C/C	A/A
Control-0107	2	65	0	0	1	C/C	G/G	A/A
Control-0108	1	55	0	0	1	T/T	G/C	G/A
Control-0109	1	38	1	0	0	C/T	C/C	A/A
Control-0110	1	64	0	0	1	T/T	G/G	A/A
Control-0111	1	62	0	0	0	C/T	G/G	A/A
Control-0112	1	54	1	0	0	T/T	C/C	G/A
Control-0113	1	64	0	0	0	C/T	C/C	A/A
Control-0114	1	56	0	0	0	T/T	G/G	A/A
Control-0115	2	60	0	0	1	C/T	C/C	A/A
Control-0116	1	64	0	0	0	C/C	G/C	A/A
Control-0117	1	58	0	0	0	C/T	C/C	A/A
Control-0118	1	46	0	0	0	T/T	G/G	A/A
Control-0119	2	72	0	0	0	C/T	G/C	G/A
Control-0120	1	44	1	0	1	C/T	C/C	A/A
Control-0121	2	41	0	0	0	C/C	G/G	A/A
Control-0122	2	67	0	0	0	T/T	C/C	G/A
Control-0123	2	65	0	0	0	T/T	G/G	G/A
Control-0124	2	66	0	0	1	T/T	G/C	G/A
Control-0125	1	61	1	0	0	T/T	C/C	A/A
Control-0126	1	52	0	0	0	C/T	C/C	A/A
Control-0127	2	67	0	0	1	C/T	G/G	A/A
Control-0128	1	60	0	0	1	C/T	G/G	A/A
Control-0129	1	56	1	0	0	C/C	C/C	A/A
Control-0130	1	60	0	0	1	C/T	C/C	G/A
Control-0131	1	57	0	0	1	C/C	C/C	A/A
Control-0132	2	65	0	0	0	C/T	G/G	A/A
Control-0133	2	70	0	0	0	C/T	G/C	G/G
Control-0134	2	69	0	0	1	C/T	G/C	G/A
Control-0135	1	53	1	0	0	C/T	C/C	A/A
Control-0136	2	25	0	0	0	T/T	G/C	A/A
Control-0137	2	43	0	0	0	C/T	C/C	A/A
Control-0138	1	62	1	0	1	C/T	C/C	G/A
Control-0139	2	74	0	0	1	C/T	C/C	G/A
Control-0140	2	73	0	0	0	T/T	G/C	G/G
Control-0141	2	62	0	0	0	C/T	C/C	A/A
Control-0142	1	58	0	0	0	T/T	C/C	A/A
Control-0143	2	69	0	0	1	C/T	G/G	A/A
Control-0144	2	51	0	0	0	C/C	G/C	A/A
Control-0145	1	61	0	0	1	C/C	C/C	A/A
Control-0146	1	74	0	0	0	C/T	G/C	A/A
Control-0147	1	55	0	0	1	C/T	G/C	A/A
Control-0148	2	72	0	0	0	C/T	C/C	A/A
Control-0149	1	56	0	0	0	C/T	G/C	A/A
Control-0150	2	78	0	0	1	C/T	G/G	G/A
Control-0151	2	63	0	0	1	C/T	C/C	G/A

Control-0152	2	61	0	0	1	C/T	G/C	A/A
Control-0153	1	59	1	0	1	T/T	C/C	A/A
Control-0154	2	63	0	0	1	C/T	G/C	A/A
Control-0155	2	39	0	0	1	T/T	G/C	A/A
Control-0156	1	58	0	0	0	C/T	C/C	A/A
Control-0157	1	54	0	0	0	T/T	G/G	A/A
Control-0158	1	57	1	0	1	C/T	G/C	A/A
Control-0159	1	66	0	0	0	T/T	C/C	G/A
Control-0160	1	74	0	0	1	C/T	C/C	A/A
Control-0161	2	50	0	0	0	T/T	G/C	A/A
Control-0162	1	68	0	0	1	C/T	G/G	A/A
Control-0163	1	60	0	0	1	T/T	C/C	G/A
Control-0164	2	59	0	0	1	T/T	G/C	G/A
Control-0165	1	73	0	0	1	C/C	G/C	A/A
Control-0166	1	75	1	0	1	T/T	C/C	A/A
Control-0167	1	65	0	0	0	C/C	C/C	G/A
Control-0168	1	62	0	0	0	C/T	G/C	A/A
Control-0169	1	57	0	0	0	T/T	C/C	A/A
Control-0170	2	76	0	0	1	C/T	G/G	A/A
Control-0171	2	60	0	0	1	T/T	G/C	A/A
Control-0172	1	59	0	0	0	C/C	G/C	A/A
Control-0173	2	52	0	0	0	C/T	G/C	A/A
Control-0174	1	52	0	0	1	T/T	C/C	A/A
Control-0175	1	76	0	0	1	C/C	G/C	A/A
Control-0176	1	75	0	0	1	C/T	C/C	A/A
Control-0177	2	51	0	0	1	C/T	C/C	A/A
Control-0178	1	66	0	0	1	C/C	C/C	A/A
Control-0179	2	61	0	0	0	C/T	C/C	A/A
Control-0180	2	62	0	0	1	T/T	G/C	A/A
Control-0181	1	40	0	0	1	C/T	G/C	A/A
Control-0182	1	50	0	1	1	T/T	C/C	A/A
Control-0183	2	59	0	0	0	T/T	C/C	G/A
Control-0184	1	48	0	0	1	C/T	C/C	A/A
Control-0185	1	74	0	1	1	T/T	C/C	A/A
Control-0186	1	84	0	0	0	C/T	G/C	G/G
Control-0187	1	57	0	0	0	C/C	C/C	G/A
Control-0188	1	69	0	0	1	C/T	C/C	A/A
Control-0189	1	62	0	0	1	C/T	C/C	A/A
Control-0190	2	57	0	0	1	C/T	C/C	A/A
Control-0191	1	37	0	0	0	C/T	G/C	A/A
Control-0192	1	64	0	0	1	T/T	G/C	A/A
Control-0193	1	64	0	0	0	C/T	G/G	A/A
Control-0194	1	77	0	0	0	T/T	G/C	G/A
Control-0195	1	60	0	0	0	C/T	C/C	A/A
Control-0196	1	68	0	0	0	C/T	G/G	G/A
Control-0197	1	81	0	0	1	C/C	G/C	G/A
Control-0198	2	55	0	0	1	T/T	C/C	A/A
Control-0199	1	79	1	1	0	T/T	C/C	G/A
Control-0200	1	71	1	1	0	C/T	G/C	A/A
Control-0201	1	64	0	0	0	C/T	G/C	A/A
Control-0202	2	57	0	0	0	C/T	C/C	G/A
Control-0203	2	78	0	0	1	C/C	G/G	G/A
Control-0204	1	58	1	0	1	C/T	C/C	A/A
Control-0205	1	74	0	0	0	C/C	G/C	A/A

Control-0206	2	54	0	0	1	C/C	G/C	A/A
Control-0207	1	59	0	1	0	C/T	C/C	A/A
Control-0208	1	70	0	0	0	C/C	C/C	A/A
Control-0209	1	55	0	0	1	C/C	G/C	G/A
Control-0210	1	58	0	0	0	T/T	C/C	A/A
Control-0211	1	59	0	0	1	T/T	G/G	A/A
Control-0212	1	60	0	0	0	C/T	C/C	G/A
Control-0213	1	58	0	0	0	T/T	G/C	A/A
Control-0214	2	60	0	0	0	T/T	C/C	A/A
Control-0215	2	61	0	0	0	C/T	C/C	A/A
Control-0216	2	67	0	0	0	C/T	C/C	G/A
Control-0217	1	59	1	0	1	C/T	C/C	A/A
Control-0218	2	55	0	0	0	C/T	C/C	A/A
Control-0219	1	66	0	0	0	T/T	G/C	A/A
Control-0220	1	64	0	0	1	C/T	C/C	A/A
Control-0221	1	56	1	0	1	C/C	C/C	A/A
Control-0222	2	61	0	0	1	T/T	C/C	A/A
Control-0223	1	60	1	0	0	C/T	C/C	G/A
Control-0224	2	66	0	0	0	C/T	G/C	G/A
Control-0225	2	63	0	0	1	C/T	G/C	G/A
Control-0226	1	65	1	0	1	C/C	G/G	A/A
Control-0227	1	60	0	0	0	C/T	C/C	G/A
Control-0228	2	51	0	0	0	C/C	C/C	A/A
Control-0229	1	57	0	0	1	C/T	G/C	A/A
Control-0230	2	79	0	0	1	T/T	C/C	G/A
Control-0231	1	55	1	1	0	C/T	G/C	A/A
Control-0232	1	51	0	0	1	T/T	C/C	A/A
Control-0233	2	66	0	0	0	C/T	C/C	A/A
Control-0234	1	53	1	0	0	C/T	G/G	A/A
Control-0235	1	61	0	0	1	C/C	G/C	G/G
Control-0236	1	62	0	0	1	C/T	C/C	A/A
Control-0237	1	60	1	0	1	T/T	G/C	G/A
Control-0238	1	71	1	1	1	C/T	G/C	G/A
Control-0239	1	67	0	0	0	C/C	C/C	A/A
Control-0240	2	69	0	0	0	C/C	G/C	A/A
Control-0241	1	62	0	0	0	T/T	G/C	A/A
Control-0242	1	61	1	0	0	C/C	G/C	G/A
Control-0243	1	62	1	0	0	C/T	G/C	A/A
Control-0244	2	70	0	0	1	C/T	C/C	A/A
Control-0245	2	56	0	0	1	C/C	G/C	A/A
Control-0246	2	68	0	0	1	T/T	C/C	G/A
Control-0247	2	53	0	0	1	C/T	G/C	A/A
Control-0248	2	56	0	0	1	C/C	G/G	A/A
Control-0249	2	48	0	0	0	C/C	G/C	A/A
Control-0250	1	61	0	0	1	C/C	G/C	G/A
Control-0251	1	67	0	0	1	C/T	G/C	A/A
Control-0252	1	57	0	0	0	C/T	C/C	A/A
Control-0253	1	63	0	0	0	C/T	G/C	A/A
Control-0254	1	74	1	0	0	T/T	C/C	G/A
Control-0255	1	67	0	0	0	C/T	C/C	A/A
Control-0256	1	61	0	0	0	C/T	C/C	A/A
Control-0257	1	74	0	1	0	T/T	G/C	G/A
Control-0258	2	65	0	0	1	C/C	G/C	A/A
Control-0259	1	71	1	0	0	T/T	C/C	A/A

Control-0260	1	59	1	0	0	T/T	G/C	G/A
Control-0261	1	69	1	0	0	C/T	G/G	A/A
Control-0262	2	78	0	0	0	C/C	C/C	A/A
Control-0263	1	59	0	0	1	C/T	G/C	A/A
Control-0264	1	63	0	0	1	C/C	G/C	G/A
Control-0265	2	63	0	0	1	T/T	G/C	G/A
Control-0266	1	71	0	0	1	C/T	G/G	G/A
Control-0267	1	64	0	1	0	C/T	G/G	A/A
Control-0268	1	56	0	0	0	C/C	C/C	G/A
Control-0269	2	72	0	0	1	C/T	C/C	A/A
Control-0270	1	62	1	0	0	C/T	G/G	A/A
Control-0271	2	51	0	0	0	C/T	G/G	A/A
Control-0272	1	58	1	0	0	T/T	G/C	G/A
Control-0273	1	60	0	0	0	T/T	G/C	G/A
Control-0274	2	66	0	0	0	C/T	G/C	G/A
Control-0275	2	70	0	0	0	C/T	C/C	G/A
Control-0276	1	57	0	0	1	T/T	C/C	A/A
Control-0277	2	61	0	0	0	C/T	C/C	G/A
Control-0278	2	59	0	0	0	C/C	C/C	A/A
Control-0279	2	54	0	0	0	C/T	C/C	A/A
Control-0280	1	64	0	0	0	T/T	G/C	A/A
Control-0281	2	48	0	0	1	C/T	G/C	A/A
Control-0282	1	63	1	0	0	C/C	G/C	A/A
Control-0283	2	69	0	0	0	T/T	G/C	A/A
Control-0284	2	74	0	0	1	C/T	G/C	G/A
Control-0285	1	59	0	0	1	C/C	G/G	A/A
Control-0286	2	63	0	0	1	T/T	C/C	A/A
Control-0287	1	73	0	0	1	C/C	C/C	G/A
Control-0288	2	68	0	0	0	T/T	G/C	A/A
Control-0289	1	65	0	0	0	T/T	G/G	G/A
Control-0290	1	73	0	0	1	C/T	G/G	G/G
Control-0291	2	59	0	0	0	C/T	G/G	A/A
Control-0292	1	65	1	0	0	T/T	G/C	A/A
Control-0293	1	66	0	0	0	T/T	C/C	G/A
Control-0294	2	67	0	0	0	C/T	C/C	A/A
Control-0295	2	55	0	0	0	C/C	C/C	A/A
Control-0296	1	71	0	0	1	C/C	G/C	G/A
Control-0297	2	67	0	0	0	C/C	C/C	A/A
Control-0298	1	53	0	0	1	C/T	C/C	A/A
Control-0299	2	67	0	0	1	T/T	C/C	A/A
Control-0300	2	68	0	0	1	C/C	G/C	G/A
Control-0301	1	55	1	0	0	C/T	G/C	A/A
Control-0302	2	63	0	0	1	T/T	G/C	A/A
Control-0303	2	72	0	0	0	C/T	G/C	A/A
Control-0304	1	56	1	0	1	C/T	G/C	A/A
Control-0305	1	56	0	0	1	C/T	G/C	A/A
Control-0306	1	65	0	0	0	T/T	G/C	A/A
Control-0307	1	59	0	0	1	C/C	G/C	A/A
Control-0308	1	72	0	0	0	C/T	C/C	A/A
Control-0309	1	59	1	0	1	C/C	C/C	A/A
Control-0310	1	77	1	0	0	C/T	G/C	A/A
Control-0311	1	60	0	0	0	T/T	C/C	G/A
Control-0312	1	56	1	0	0	C/T	G/C	G/A
Control-0313	1	62	0	0	0	T/T	C/C	A/A

Control-0314	2	65	0	0	1	C/T	G/C	A/A
Control-0315	1	49	1	0	0	C/T	G/C	A/A
Control-0316	1	71	1	0	1	T/T	C/C	A/A
Control-0317	1	52	0	0	0	C/T	G/C	A/A
Control-0318	1	85	1	0	1	T/T	G/C	G/A
Control-0319	1	47	0	0	0	T/T	C/C	G/A
Control-0320	1	48	0	0	1	T/T	C/C	A/A
Control-0321	2	46	0	0	0	C/T	C/C	A/A
Control-0322	2	51	0	0	0	C/T	C/C	G/A
Control-0323	1	67	1	0	1	T/T	G/C	A/A
Control-0324	1	78	0	0	0	T/T	C/C	A/A
Control-0325	1	48	1	0	0	T/T	C/C	G/A
Control-0326	2	56	0	0	0	T/T	G/C	A/A
Control-0327	2	51	0	0	1	C/T	G/G	A/A
Control-0328	2	63	0	0	1	C/T	G/C	A/A
Control-0329	2	54	0	0	1	C/T	C/C	A/A
Control-0330	2	52	0	0	1	T/T	G/C	A/A
Control-0331	1	52	0	0	1	C/T	G/C	G/A
Control-0332	1	58	0	0	0	C/C	C/C	A/A
Control-0333	1	51	0	0	0	C/T	G/C	A/A
Control-0334	2	68	0	0	1	C/T	G/C	G/A
Control-0335	1	52	0	0	0	C/C	C/C	A/A
Control-0336	2	51	0	0	1	C/T	C/C	A/A
Control-0337	2	66	0	0	1	T/T	C/C	A/A
Control-0338	2	62	0	0	0	C/T	C/C	A/A
Control-0339	1	66	0	0	0	T/T	G/C	G/A
Control-0340	1	65	0	0	1	T/T	C/C	A/A
Control-0341	2	60	0	0	0	T/T	G/C	A/A
Control-0342	1	48	0	0	1	C/T	G/C	A/A
Control-0343	2	53	0	0	1	C/T	G/C	A/A
Control-0344	1	70	1	0	1	C/T	G/C	A/A
Control-0345	2	60	0	0	1	C/T	G/C	A/A
Control-0346	2	63	0	0	0	T/T	G/C	A/A
Control-0347	2	61	0	0	1	C/T	G/C	A/A
Control-0348	1	55	0	0	0	C/T	G/C	A/A
Control-0349	1	69	0	0	1	T/T	C/C	A/A
Control-0350	1	67	0	0	1	C/T	C/C	G/A
Control-0351	1	62	0	0	0	C/C	G/C	A/A
Control-0352	2	49	0	0	0	C/T	C/C	G/A
Control-0353	1	55	0	0	1	C/T	C/C	A/A
Control-0354	1	62	1	0	0	C/T	G/C	A/A
Control-0355	1	48	1	0	1	T/T	G/C	A/A
Control-0356	1	49	1	0	1	C/T	G/C	G/A
Control-0357	1	61	0	0	1	C/T	C/C	A/A
Control-0358	2	53	0	0	0	C/T	C/C	A/A
Control-0359	1	52	0	0	0	T/T	G/C	A/A
Control-0360	2	67	0	0	0	C/C	C/C	A/A
Control-0361	1	30	0	0	0	T/T	G/G	G/G
Control-0362	1	58	0	0	0	C/C	C/C	G/A
Control-0363	2	61	0	0	1	C/T	C/C	A/A
Control-0364	1	54	0	0	0	C/T	C/C	A/A
Control-0365	2	42	0	0	0	T/T	C/C	A/A
Control-0366	2	61	0	0	0	C/T	G/C	G/A
Control-0367	1	57	0	0	0	T/T	G/G	A/A

Control-0368	1	50	1	0	1	C/T	C/C	A/A
Control-0369	2	66	0	0	0	C/T	C/C	A/A
Control-0370	1	55	1	0	1	C/T	G/G	A/A
Control-0371	2	52	0	0	1	T/T	G/C	A/A
Control-0372	1	41	0	0	0	C/C	C/C	A/A
Control-0373	2	63	0	0	1	T/T	G/C	A/A
Control-0374	1	59	0	0	1	C/T	G/C	A/A
Control-0375	1	74	1	1	0	T/T	G/C	A/A
Control-0376	1	68	0	0	0	C/T	G/C	G/A
Control-0377	1	63	1	1	1	T/T	G/C	G/A
Control-0378	1	60	1	0	0	C/T	C/C	G/A
Control-0379	1	59	0	0	1	T/T	G/C	G/A
Control-0380	2	57	0	0	1	C/C	C/C	A/A
Control-0381	1	60	1	0	1	C/T	C/C	G/A
Control-0382	2	42	0	0	0	C/T	G/C	A/A
Control-0383	1	54	1	0	0	C/C	G/C	A/A
Control-0384	2	42	0	0	1	C/T	C/C	A/A
Control-0385	1	51	0	0	0	T/T	G/C	A/A
Control-0386	1	50	0	0	1	T/T	G/C	A/A
Control-0387	1	58	0	0	1	T/T	C/C	G/A
Control-0388	2	65	1	0	1	C/T	C/C	G/A
Control-0389	1	51	0	0	0	C/T	G/C	A/A
Control-0390	1	64	0	0	1	T/T	G/C	A/A
Control-0391	1	27	0	0	0	C/T	C/C	G/A
Control-0392	1	63	0	0	0	C/T	G/C	A/A
Control-0393	1	53	0	0	0	T/T	G/C	A/A
Control-0394	2	64	0	0	0	T/T	C/C	A/A
Control-0395	1	55	1	0	0	T/T	C/C	A/A
Control-0396	1	39	1	0	0	T/T	C/C	G/A
Control-0397	1	69	0	0	1	C/C	C/C	A/A
Control-0398	1	50	1	0	0	C/T	C/C	A/A
Control-0399	1	76	0	0	0	T/T	G/C	A/A
Control-0400	1	79	1	0	0	C/T	C/C	A/A
Control-0401	1	52	0	0	0	C/C	C/C	A/A
Control-0402	2	58	0	0	1	C/C	G/C	A/A
Control-0403	1	43	0	0	0	C/C	G/C	A/A
Control-0404	2	53	0	0	0	C/T	C/C	A/A
Control-0405	1	78	0	0	0	C/T	G/C	G/A
Control-0406	1	63	1	0	0	C/T	G/C	G/A
Control-0407	1	57	1	0	1	C/T	G/C	G/A
Control-0408	2	60	0	0	0	C/T	G/G	A/A
Control-0409	1	67	0	0	1	T/T	G/C	G/A
Control-0410	1	66	0	0	1	C/T	G/C	A/A
Control-0411	1	62	0	0	1	T/T	G/G	G/A
Control-0412	2	61	0	0	1	C/T	G/G	A/A
Control-0413	1	61	0	0	1	C/T	G/C	A/A
Control-0414	1	66	0	0	1	C/C	G/G	A/A
Control-0415	1	52	0	0	1	C/C	C/C	G/A
Control-0416	1	60	1	0	0	T/T	C/C	G/A
Control-0417	1	72	1	1	0	C/C	G/C	A/A
Control-0418	1	70	0	0	0	C/C	G/C	A/A
Control-0419	1	68	0	0	0	T/T	C/C	A/A
Control-0420	1	71	1	0	0	C/T	G/C	A/A
Control-0421	2	69	0	0	1	C/T	G/C	A/A

Control-0422	2	61	0	0	1	C/C	C/C	G/A
Control-0423	1	69	0	0	1	C/T	G/C	A/A
Control-0424	1	74	1	0	1	C/C	G/C	A/A
Control-0425	2	52	0	0	1	T/T	G/G	A/A
Control-0426	1	41	0	0	0	T/T	C/C	A/A
Control-0427	1	52	0	0	0	T/T	G/C	A/A
Control-0428	2	44	0	0	0	C/C	G/C	G/G
Control-0429	2	50	0	0	0	C/T	G/C	A/A
Control-0430	1	63	0	0	1	C/T	G/C	G/A
Control-0431	1	59	1	0	1	T/T	G/G	A/A
Control-0432	1	44	0	0	1	C/T	C/C	G/A
Control-0433	2	66	0	0	0	C/C	C/C	G/A
Control-0434	1	59	1	1	0	T/T	C/C	A/A
Control-0435	1	61	1	0	1	C/T	G/C	G/A
Control-0436	2	53	0	0	1	T/T	C/C	A/A
Control-0437	1	66	1	0	1	C/T	G/C	A/A
Control-0438	2	54	0	0	0	C/T	C/C	A/A
Control-0439	2	51	0	0	1	C/C	G/C	G/A
Control-0440	2	61	0	0	0	C/T	G/G	A/A
Control-0441	1	56	0	0	1	T/T	G/C	A/A
Control-0442	1	52	0	0	1	C/T	C/C	G/A
Control-0443	2	49	0	0	1	C/T	C/C	A/A
Control-0444	2	48	0	0	0	C/T	C/C	A/A
Control-0445	2	45	0	0	1	C/C	G/C	A/A
Control-0446	2	47	0	0	0	C/C	C/C	G/A
Control-0447	2	65	0	0	1	T/T	C/C	A/A
Control-0448	1	73	1	0	0	C/T	G/C	A/A
Control-0449	2	59	0	0	0	C/C	C/C	A/A
Control-0450	1	59	0	0	0	C/T	G/C	G/A
Control-0451	1	67	0	0	0	C/T	C/C	A/A
Control-0452	1	59	0	0	1	T/T	C/C	G/A
Control-0453	1	59	0	0	1	C/T	G/C	A/A
Control-0454	1	47	1	0	1	C/T	G/C	A/A
Control-0455	2	68	0	0	0	C/T	G/G	A/A
Control-0456	1	64	0	1	0	C/C	G/C	A/A
Control-0457	2	50	0	0	1	C/T	G/C	G/A
Control-0458	1	74	1	0	0	C/C	G/G	A/A
Control-0459	2	50	0	0	0	T/T	G/C	A/A
Control-0460	1	70	0	0	0	C/T	C/C	A/A
Control-0461	2	63	0	0	0	C/T	G/C	A/A
Control-0462	1	53	0	0	0	C/C	G/C	A/A
Control-0463	2	59	0	0	0	C/T	G/C	G/A
Control-0464	1	58	0	0	0	C/T	C/C	G/A
Control-0465	1	68	0	0	0	T/T	G/C	A/A
Control-0466	2	53	0	0	1	C/T	C/C	A/A
Control-0467	2	65	0	0	1	C/T	C/C	G/A
Control-0468	1	73	1	0	0	C/T	C/C	A/A
Control-0469	2	56	0	0	1	C/T	G/C	A/A
Control-0470	1	59	0	0	0	C/C	G/C	A/A
Control-0471	2	51	0	0	1	T/T	C/C	A/A
Control-0472	1	62	0	0	1	T/T	G/C	G/A
Control-0473	1	55	0	0	0	T/T	G/C	A/A
Control-0474	2	59	0	0	1	C/T	G/C	A/A
Control-0475	1	62	0	0	1	C/T	C/C	A/A

Control-0476	1	69	0	0	0	T/T	G/C	G/A
Control-0477	1	29	0	0	1	C/T	G/G	A/A
Control-0478	1	59	0	0	0	C/T	G/C	A/A
Control-0479	1	52	1	0	0	C/T	G/C	A/A
Control-0480	1	60	0	0	1	C/T	G/C	A/A
Control-0481	2	61	0	0	0	T/T	C/C	A/A
Control-0482	2	69	0	0	1	C/T	C/C	A/A
Control-0483	1	59	0	0	1	C/C	C/C	A/A
Control-0484	2	63	0	0	1	C/C	C/C	A/A
Control-0485	2	66	0	0	0	C/T	G/C	G/A
Control-0486	1	63	0	0	1	C/T	G/C	A/A
Control-0487	1	74	0	0	1	C/C	C/C	A/A
Control-0488	2	59	0	0	1	C/C	C/C	A/A
Control-0489	1	57	0	0	1	C/T	C/C	A/A
Control-0490	1	63	0	0	0	C/T	C/C	A/A
Control-0491	2	68	0	0	1	C/T	G/C	A/A
Control-0492	1	61	1	0	1	T/T	C/C	A/A
Control-0493	1	53	0	0	1	C/T	G/C	G/A
Control-0494	2	58	0	0	0	C/T	G/C	A/A
Control-0495	1	62	0	0	1	C/T	G/C	A/A
Control-0496	1	57	1	0	0	C/T	G/C	A/A
Control-0497	1	57	1	0	1	C/C	C/C	G/A
Control-0498	1	46	1	1	1	C/T	G/C	G/A
Control-0499	1	60	1	1	0	C/T	G/G	A/A
Control-0500	2	65	0	0	0	C/T	G/C	G/A
Control-0501	1	62	0	0	0	T/T	C/C	A/A
Control-0502	1	54	1	0	0	T/T	C/C	G/A
Control-0503	1	53	0	0	0	T/T	C/C	A/A
Control-0504	2	64	0	0	1	C/T	G/C	A/A
Control-0505	1	64	1	1	1	C/C	G/C	A/A
Control-0506	1	55	0	0	1	T/T	C/C	A/A
Control-0507	2	60	0	0	0	T/T	G/C	A/A
Control-0508	1	59	1	0	0	C/T	C/C	A/A
Control-0509	1	59	1	0	1	C/T	G/C	G/A
Control-0510	1	63	1	0	0	T/T	C/C	A/A
Control-0511	1	62	0	1	1	T/T	C/C	A/A
Control-0512	1	53	0	0	1	T/T	G/C	A/A
Control-0513	1	52	0	0	0	C/C	G/C	A/A
Control-0514	1	54	1	0	1	C/T	G/C	A/A
Control-0515	1	55	0	0	0	C/T	C/C	A/A
Control-0516	2	69	0	0	1	C/T	G/C	G/A
Control-0517	1	65	0	1	0	C/T	C/C	A/A
Control-0518	1	58	0	1	1	C/T	C/C	G/A
Control-0519	1	51	1	0	1	C/T	C/C	A/A
Control-0520	1	48	1	0	1	T/T	C/C	G/A
Control-0521	2	54	0	0	0	C/C	G/C	A/A
Control-0522	2	60	0	0	1	C/T	G/G	G/A
Control-0523	2	64	0	0	0	C/C	C/C	A/A
Control-0524	1	59	1	1	1	C/T	G/C	G/A
Control-0525	2	59	0	0	0	C/T	G/C	A/A
Control-0526	1	56	1	0	0	C/T	G/C	A/A
Control-0527	1	60	0	0	0	T/T	C/C	A/A
Control-0528	1	51	0	0	0	C/C	C/C	A/A
Control-0529	1	58	1	0	0	C/T	C/C	A/A

Control-0530	1	65	0	0	0	T/T	C/C	A/A
Control-0531	1	63	0	0	0	C/T	C/C	A/A
Control-0532	2	58	0	0	0	T/T	G/C	A/A
Control-0533	2	61	0	0	0	C/T	G/C	A/A
Control-0534	1	62	1	1	1	T/T	C/C	G/A
Control-0535	1	54	0	0	0	C/T	G/G	G/A
Control-0536	1	58	0	0	1	T/T	C/C	A/A
Control-0537	2	60	0	0	1	C/T	G/C	A/A
Control-0538	1	59	1	0	1	C/T	G/C	A/A
Control-0539	2	59	0	0	1	C/C	G/G	A/A
Control-0540	1	64	0	0	1	C/T	C/C	A/A
Control-0541	1	58	1	0	0	T/T	G/C	A/A
Control-0542	1	60	0	0	1	C/T	G/C	G/A
Control-0543	1	60	0	1	1	T/T	C/C	A/A
Control-0544	2	50	0	0	0	C/T	G/C	A/A
Control-0545	1	54	1	1	1	T/T	C/C	G/A
Control-0546	1	58	0	0	1	C/C	G/G	A/A
Control-0547	1	57	0	0	0	C/C	G/G	G/A
Control-0548	1	62	0	0	1	C/T	C/C	G/A
Control-0549	1	65	0	0	1	T/T	G/C	A/A
Control-0550	1	61	0	0	1	C/T	C/C	G/A
Control-0551	2	61	0	0	0	C/T	C/C	A/A
Control-0552	2	57	0	0	0	C/T	G/C	G/A
Control-0553	1	56	1	1	1	C/T	C/C	A/A
Control-0554	2	59	0	0	1	T/T	G/G	A/A
Control-0555	2	61	0	0	1	T/T	G/G	A/A
Control-0556	2	54	0	1	0	T/T	G/C	A/A
Control-0557	1	65	0	0	1	C/T	C/C	A/A
Control-0558	1	64	1	0	0	C/T	C/C	G/A
Control-0559	2	66	0	0	1	C/T	G/C	A/A
Control-0560	1	56	1	1	0	C/T	G/C	A/A
Control-0561	1	51	1	0	1	C/T	G/C	A/A
Control-0562	1	54	0	0	1	C/T	G/G	G/A
Control-0563	1	59	1	1	0	C/T	G/C	G/A
Control-0564	1	57	1	0	1	C/C	C/C	A/A
Control-0565	1	52	1	1	0	C/C	G/C	A/A
Control-0566	2	65	0	0	0	T/T	G/C	A/A
Control-0567	1	59	1	0	0	C/T	G/C	A/A
Control-0568	1	64	0	1	1	C/T	C/C	A/A
Control-0569	1	57	1	0	1	C/T	C/C	A/A
Control-0570	1	52	0	0	1	C/T	G/C	A/A
Control-0571	1	51	0	0	1	C/T	G/G	A/A
Control-0572	1	63	0	0	0	T/T	C/C	A/A
Control-0573	1	63	1	0	1	C/T	G/C	G/A
Control-0574	2	68	0	0	1	C/T	G/G	A/A
Control-0575	2	55	0	0	1	T/T	C/C	A/A
Control-0576	1	55	1	1	1	C/T	G/C	A/A
Control-0577	1	64	1	1	0	T/T	G/G	G/A
Control-0578	1	60	0	0	0	C/C	C/C	G/A
Control-0579	1	50	0	0	0	C/T	G/G	A/A
Control-0580	1	51	0	1	1	C/C	G/C	G/A
Control-0581	1	58	1	1	0	T/T	G/C	A/A
Control-0582	1	57	0	0	0	C/T	C/C	G/G
Control-0583	1	61	1	1	1	C/T	C/C	G/A

Control-0584	1	63	1	0	0	T/T	C/C	G/A
Control-0585	1	60	1	0	0	C/C	C/C	A/A
Control-0586	2	66	0	0	1	C/C	G/C	A/A
Control-0587	1	65	1	0	0	C/T	G/C	G/A
Control-0588	2	63	0	0	0	T/T	C/C	G/A
Control-0589	2	59	0	0	1	C/T	G/C	A/A
Control-0590	1	59	1	1	0	C/C	G/C	G/A
Control-0591	1	61	1	1	0	C/T	G/C	A/A
Control-0592	1	50	0	1	1	C/C	C/C	G/A
Control-0593	2	50	0	0	1	T/T	G/C	A/A
Control-0594	2	56	0	0	0	C/T	G/C	G/A
Control-0595	1	55	0	0	0	T/T	G/C	G/A
Control-0596	2	59	0	0	1	C/T	C/C	G/G
Control-0597	1	59	0	0	0	T/T	G/C	A/A
Control-0598	1	59	1	0	0	C/T	G/G	A/A
Control-0599	1	56	0	0	1	T/T	G/C	A/A
Control-0600	2	63	0	0	1	C/T	G/G	A/A
Control-0601	1	52	1	0	1	C/T	C/C	A/A
Control-0602	2	68	0	0	1	C/T	C/C	A/A
Control-0603	2	53	0	0	1	C/T	G/C	A/A
Control-0604	1	61	1	1	0	C/C	C/C	A/A
Control-0605	2	68	0	0	0	C/T	G/G	G/A
Control-0606	1	56	0	0	0	C/C	G/C	A/A
Control-0607	1	64	1	1	0	C/C	G/C	A/A
Control-0608	2	53	0	0	1	T/T	G/G	A/A
Control-0609	1	52	0	0	1	C/T	C/C	A/A
Control-0610	1	52	1	0	1	T/T	G/C	A/A
Control-0611	1	66	0	0	0	C/T	C/C	A/A
Control-0612	2	57	0	0	0	T/T	C/C	A/A
Control-0613	1	56	1	1	0	C/T	G/G	A/A
Control-0614	2	62	0	0	0	T/T	G/C	G/A
Control-0615	2	61	0	0	0	C/T	C/C	A/A
Control-0616	2	57	0	0	0	C/C	C/C	A/A
Control-0617	2	63	0	0	1	C/T	C/C	A/A
Control-0618	2	58	0	0	1	C/T	C/C	G/A
Control-0619	1	50	0	0	0	C/C	G/G	A/A
Control-0620	2	55	0	0	1	C/T	G/C	A/A
Control-0621	1	56	0	0	1	C/T	G/C	A/A
Control-0622	2	59	0	0	0	T/T	G/C	A/A
Control-0623	1	65	1	1	0	T/T	G/C	G/G
Control-0624	2	47	0	0	1	C/C	G/C	G/A
Control-0625	1	58	1	1	0	C/T	C/C	A/A
Control-0626	1	60	1	1	0	T/T	G/C	A/A
Control-0627	1	69	0	0	1	C/T	C/C	A/A
Control-0628	2	62	0	0	0	C/T	C/C	G/A
Control-0629	2	61	0	0	1	C/T	G/G	A/A
Control-0630	2	69	0	0	1	C/T	G/C	A/A
Control-0631	2	53	0	0	0	C/T	G/C	A/A
Control-0632	1	58	0	0	1	C/T	G/C	A/A
Control-0633	2	61	0	0	0	C/C	C/C	A/A
Control-0634	1	61	0	0	0	T/T	C/C	A/A
Control-0635	2	53	0	0	0	C/C	C/C	A/A
Control-0636	1	69	0	0	1	C/T	G/C	A/A
Control-0637	2	45	0	0	1	C/T	C/C	A/A

Control-0638	1	55	1	1	0	C/T	G/C	A/A
Control-0639	2	52	0	0	1	T/T	G/C	G/A
Control-0640	1	67	0	0	1	C/C	G/C	A/A
Control-0641	1	53	1	0	1	C/C	G/C	G/A
Control-0642	2	59	0	0	1	C/T	C/C	A/A
Control-0643	1	49	1	1	0	C/T	C/C	A/A
Control-0644	2	59	0	0	1	C/C	G/C	G/A
Control-0645	1	55	1	0	1	T/T	G/C	G/G
Control-0646	1	62	0	0	1	C/C	G/C	A/A
Control-0647	2	63	0	0	1	T/T	G/C	A/A
Control-0648	1	57	0	1	0	C/T	G/C	A/A
Control-0649	1	53	0	0	0	T/T	G/C	A/A
Control-0650	2	68	0	0	0	C/T	G/C	A/A
Control-0651	1	45	0	0	1	T/T	G/C	A/A
Control-0652	1	67	1	1	0	T/T	C/C	A/A
Control-0653	1	52	0	0	1	C/T	C/C	G/A
Control-0654	2	47	0	0	0	T/T	G/C	A/A
Control-0655	1	53	0	0	1	C/T	C/C	G/A
Control-0656	2	56	0	0	0	T/T	G/G	G/A
Control-0657	2	53	0	0	0	C/C	G/C	A/A
Control-0658	1	52	0	1	0	C/C	C/C	A/A
Control-0659	2	67	0	0	1	C/T	C/C	G/A
Control-0660	2	48	0	0	1	C/T	C/C	A/A
Control-0661	1	52	0	0	1	T/T	G/G	A/A
Control-0662	2	65	0	0	1	C/T	C/C	A/A
Control-0663	2	48	0	0	0	C/T	C/C	A/A
Control-0664	2	59	0	0	0	T/T	C/C	G/A
Control-0665	1	69	1	1	0	C/T	G/C	A/A
Control-0666	1	50	0	0	0	C/T	G/C	A/A
Control-0667	2	53	0	0	1	C/C	C/C	A/A
Control-0668	1	54	0	0	1	C/T	C/C	A/A
Control-0669	1	56	0	0	1	C/T	C/C	A/A
Control-0670	2	58	0	0	1	C/C	C/C	A/A
Control-0671	1	60	0	0	0	C/T	G/C	A/A
Control-0672	1	82	1	0	0	C/T	C/C	A/A
Control-0673	1	60	1	0	0	C/T	G/G	A/A
Control-0674	2	57	0	0	1	C/T	C/C	A/A
Control-0675	1	55	0	0	0	T/T	C/C	A/A
Control-0676	2	68	0	0	0	C/T	C/C	G/A
Control-0677	2	61	0	0	1	C/T	G/G	A/A
Control-0678	1	45	1	1	0	C/T	G/C	G/A
Control-0679	2	64	0	0	0	C/C	G/C	A/A
Control-0680	2	66	0	0	0	T/T	G/C	G/A
Control-0681	2	67	0	0	0	C/T	G/C	A/A
Control-0682	2	51	0	0	1	C/T	G/G	A/A
Control-0683	2	67	0	0	1	C/T	G/G	A/A
Control-0684	1	51	1	0	0	C/T	C/C	A/A
Control-0685	1	65	0	0	0	T/T	G/C	A/A
Control-0686	2	61	0	0	1	T/T	G/G	G/A
Control-0687	2	65	0	0	0	C/T	G/C	A/A
Control-0688	1	65	0	0	1	C/T	C/C	G/A
Control-0689	1	60	1	0	1	T/T	C/C	A/A
Control-0690	1	58	1	1	1	C/T	G/C	G/A
Control-0691	2	67	0	0	0	T/T	G/C	A/A

Control-0692	2	66	0	0	1	C/T	C/C	A/A
Control-0693	1	68	0	0	1	C/T	G/C	A/A
Control-0694	1	43	0	0	1	C/C	G/G	A/A
Control-0695	2	47	0	0	1	C/T	G/C	A/A
Control-0696	2	52	0	0	1	C/T	G/C	G/A
Control-0697	1	52	0	0	1	C/C	C/C	A/A
Control-0698	2	61	0	0	0	C/C	G/C	A/A
Control-0699	2	66	0	0	0	C/C	G/C	A/A
Control-0700	2	59	0	0	0	C/T	G/C	A/A
Control-0701	1	63	1	0	1	C/C	G/G	G/A
Control-0702	1	59	1	1	0	C/T	G/C	A/A
Control-0703	2	56	0	0	1	C/C	G/C	A/A
Control-0704	2	69	0	0	1	C/T	C/C	A/A
Control-0705	1	58	1	1	1	C/C	G/C	A/A
Control-0706	1	49	1	1	1	T/T	G/C	G/A
Control-0707	2	60	0	0	1	C/T	C/C	A/A
Control-0708	1	51	1	1	0	C/T	G/C	A/A
Control-0709	2	51	0	0	1	C/C	G/G	A/A
Control-0710	1	46	1	0	1	C/T	C/C	A/A
Control-0711	1	64	1	0	0	C/T	G/C	A/A
Control-0712	1	56	1	1	1	T/T	G/G	A/A
Control-0713	2	46	0	0	0	T/T	C/C	A/A
Control-0714	2	56	0	0	0	T/T	G/C	A/A
Control-0715	1	53	0	0	1	C/T	G/C	A/A
Control-0716	2	66	0	0	1	T/T	C/C	A/A
Control-0717	2	54	0	0	1	C/T	C/C	A/A
Control-0718	1	53	0	0	0	T/T	G/C	A/A
Control-0719	1	65	1	0	1	C/C	G/C	A/A
Control-0720	2	60	0	0	0	C/T	G/C	G/A
Control-0721	2	48	0	0	0	C/T	C/C	A/A
Control-0722	1	52	0	0	0	T/T	G/C	A/A
Control-0723	2	62	0	0	0	T/T	G/C	A/A
Control-0724	2	61	0	0	0	C/T	C/C	G/A
Control-0725	1	56	0	0	1	T/T	C/C	A/A
Control-0726	1	57	0	0	1	T/T	G/C	G/A
Control-0727	1	61	1	1	0	T/T	G/G	A/A
Control-0728	2	65	0	0	0	C/T	C/C	A/A
Control-0729	2	68	0	0	0	C/T	G/G	A/A
Control-0730	1	60	1	0	0	C/T	C/C	A/A
Control-0731	1	65	0	0	1	T/T	G/C	G/A
Control-0732	2	56	0	0	0	C/C	G/C	A/A
Control-0733	1	50	0	1	0	C/C	C/C	A/A
Control-0734	1	49	0	0	0	C/C	G/C	A/A
Control-0735	2	47	0	0	1	C/T	G/C	A/A
Control-0736	2	71	0	0	1	T/T	C/C	G/A
Control-0737	2	64	0	0	0	C/T	G/C	A/A
Control-0738	2	61	0	0	1	C/C	G/G	G/A
Control-0739	2	60	0	0	0	T/T	G/G	A/A
Control-0740	2	66	0	0	1	C/T	C/C	A/A
Control-0741	2	77	0	0	0	T/T	C/C	G/A
Control-0742	2	75	0	0	1	C/T	C/C	A/A
Control-0743	2	43	0	0	1	C/C	G/G	G/A
Control-0744	2	66	0	0	1	C/T	G/G	G/A
Control-0745	1	58	0	0	0	T/T	C/C	A/A

Control-0746	1	58	0	1	1	T/T	G/C	A/A
Control-0747	1	62	0	0	1	C/T	G/G	A/A
Control-0748	2	66	0	0	1	C/C	C/C	G/A
Control-0749	2	61	0	0	0	C/C	G/C	A/A
Control-0750	1	66	1	0	0	C/T	G/C	A/A
Control-0751	2	61	0	0	0	C/T	C/C	G/A
Control-0752	2	70	0	0	1	C/T	C/C	A/A
Control-0753	1	70	0	0	1	C/C	C/C	A/A
Control-0754	1	69	1	1	1	C/T	C/C	A/A
Control-0755	1	60	0	0	0	C/T	G/G	A/A
Control-0756	2	72	0	0	1	C/T	C/C	A/A
Control-0757	1	56	0	0	1	T/T	G/C	G/A
Control-0758	1	66	0	0	0	C/T	C/C	A/A
Control-0759	2	51	0	0	0	C/C	C/C	A/A
Control-0760	1	47	1	1	1	C/T	G/C	A/A
Control-0761	2	72	0	0	0	C/C	G/C	A/A
Control-0762	2	74	0	0	0	C/C	G/C	G/A
Control-0763	2	59	0	0	0	C/C	G/G	A/A
Control-0764	1	69	0	0	1	C/T	G/C	A/A
Control-0765	1	64	0	0	0	T/T	C/C	A/A
Control-0766	2	66	0	0	0	T/T	G/C	A/A
Control-0767	2	72	0	0	1	C/T	C/C	A/A
Control-0768	2	67	0	0	1	T/T	G/C	A/A
Control-0769	2	79	0	0	1	C/T	G/C	G/A
Control-0770	2	59	0	0	0	C/T	G/C	G/A
Control-0771	2	53	0	0	0	C/C	C/C	A/A
Control-0772	1	66	0	0	1	C/T	C/C	G/A
Control-0773	1	50	0	0	1	T/T	C/C	A/A
Control-0774	1	67	0	0	1	C/T	C/C	G/A
Control-0775	1	83	0	0	1	C/T	G/G	A/A
Control-0776	2	50	0	0	0	C/T	G/C	G/A
Control-0777	2	69	0	0	0	C/C	G/C	A/A
Control-0778	1	52	0	0	0	T/T	G/G	G/A
Control-0779	2	69	0	0	0	C/T	C/C	A/A
Control-0780	2	83	0	0	1	C/T	G/G	A/A
Control-0781	2	68	0	0	0	C/C	G/C	A/A
Control-0782	2	61	0	0	0	C/T	G/C	G/A
Control-0783	2	53	0	0	1	C/T	G/C	A/A
Control-0784	2	74	0	0	1	C/T	G/G	A/A
Control-0785	2	67	0	0	1	C/C	G/C	A/A
Control-0786	2	73	0	0	0	T/T	C/C	A/A
Control-0787	2	64	0	0	0	T/T	G/G	A/A
Control-0788	2	61	0	0	1	C/C	G/C	A/A
Control-0789	2	68	0	0	1	C/T	C/C	A/A
Control-0790	2	62	0	0	1	T/T	G/G	G/A
Control-0791	2	75	0	0	1	C/T	C/C	A/A
Control-0792	2	65	0	0	1	C/T	G/C	G/A
Control-0793	2	69	0	0	1	C/T	G/C	A/A
Control-0794	2	48	0	0	0	T/T	C/C	A/A
Control-0795	2	63	0	0	1	C/T	C/C	A/A
Control-0796	2	41	0	0	0	T/T	C/C	A/A
Control-0797	2	49	0	0	0	C/T	C/C	A/A
Control-0798	2	66	0	0	0	C/T	G/G	A/A
Control-0799	2	45	0	0	0	C/T	C/C	G/A

Control-0800	2	45	0	0	1	C/T	G/C	A/A
Control-0801	2	46	0	0	1	C/T	G/C	A/A
Control-0802	2	67	0	0	0	C/T	C/C	G/A
Control-0803	2	65	0	0	0	C/T	G/C	A/A
Control-0804	2	74	0	0	0	C/C	C/C	A/A
Control-0805	2	62	0	0	0	C/T	G/C	A/A
Control-0806	2	55	0	0	0	C/T	G/C	A/A
Control-0807	2	59	0	0	1	C/T	C/C	A/A
Control-0808	2	59	1	1	1	C/T	C/C	A/A
Control-0809	2	57	0	0	1	C/T	C/C	G/A
Control-0810	2	64	0	0	0	C/C	C/C	G/A
Control-0811	2	58	0	0	1	T/T	C/C	A/A
Control-0812	2	64	0	0	1	C/C	C/C	A/A
Control-0813	2	62	0	0	0	C/C	G/C	G/A
Control-0814	2	63	0	0	0	T/T	C/C	A/A
Control-0815	2	56	0	0	1	T/T	G/C	A/A
Control-0816	1	57	0	0	0	T/T	G/C	A/A
Control-0817	1	54	0	0	1	T/T	C/C	A/A
Control-0818	1	52	0	0	0	C/T	G/G	A/A
Control-0819	2	52	0	0	0	T/T	G/G	A/A
Control-0820	1	53	0	0	0	C/C	G/G	A/A
Control-0821	1	59	0	0	0	T/T	G/C	A/A
Control-0822	1	61	0	0	0	T/T	G/C	G/A
Control-0823	2	**66**	0	**0**	0	C/C	C/C	A/A
Control-0824	1	63	0	0	0	C/C	G/C	A/A
Control-0825	1	57	0	0	1	C/T	G/C	G/A
Control-0826	1	67	0	0	0	C/T	C/C	G/A
Control-0827	1	65	0	0	1	C/T	G/C	G/A
Control-0828	1	56	0	0	1	C/T	G/C	G/A
Control-0829	1	67	0	0	0	C/T	G/C	A/A
Control-0830	1	71	1	1	0	C/T	G/C	A/A
Control-0831	1	65	0	0	0	C/C	C/C	A/A
Control-0832	1	55	0	0	0	C/T	G/C	A/A
Control-0833	1	68	0	0	1	C/T	C/C	A/A
Control-0834	2	53	0	0	0	C/C	G/C	A/A
Control-0835	2	65	0	0	0	C/T	G/C	A/A
Control-0836	2	72	0	0	0	C/T	G/C	G/A
Control-0837	1	65	1	0	1	C/C	G/C	A/A
Control-0838	1	65	1	0	0	C/T	C/C	A/A
Control-0839	2	68	0	0	1	C/T	C/C	A/A
Control-0840	1	62	0	0	1	C/T	G/G	A/A
Control-0841	1	52	0	0	1	C/T	C/C	G/A
Control-0842	1	70	1	1	0	T/T	G/G	A/A
Control-0843	2	49	0	0	1	C/T	C/C	A/A
Control-0844	2	56	0	0	0	T/T	G/C	A/A
Control-0845	2	64	0	0	1	C/T	C/C	A/A
Control-0846	2	68	0	0	0	C/T	G/G	A/A
Control-0847	2	53	0	0	0	C/T	C/C	A/A
Control-0848	2	54	0	0	0	C/T	G/C	G/A
Control-0849	2	68	0	0	0	C/C	G/C	G/A
Control-0850	2	49	0	0	1	T/T	C/C	A/A
Control-0851	2	61	0	0	0	C/T	G/C	G/A
Control-0852	2	59	0	0	0	C/T	G/C	A/A
Control-0853	2	57	0	0	0	C/T	C/C	A/A

Control-0854	2	52	0	0	1	C/T	G/C	A/A
Control-0855	2	58	0	0	0	T/T	G/C	G/A
Control-0856	2	64	0	0	0	C/C	C/C	A/A
Control-0857	2	59	0	0	1	C/T	G/G	A/A
Control-0858	2	58	0	0	0	T/T	C/C	A/A
Control-0859	2	62	0	0	0	T/T	G/G	G/A
Control-0860	2	63	0	0	1	C/T	G/C	G/A
Control-0861	2	73	0	0	0	C/T	G/C	G/A
Control-0862	2	59	0	0	0	C/C	G/C	G/A
Control-0863	2	77	0	0	1	C/C	C/C	A/A
Control-0864	2	59	0	0	0	C/T	C/C	G/G
Control-0865	2	59	0	0	0	T/T	G/C	A/A
Control-0866	2	73	0	0	1	C/C	G/G	A/A
Control-0867	2	60	0	0	1	C/C	G/C	A/A
Control-0868	2	73	0	0	0	C/C	G/C	A/A
Control-0869	2	59	0	0	1	T/T	G/C	G/A
Control-0870	2	71	0	0	1	C/T	G/G	A/A
Control-0871	2	59	0	0	1	C/C	C/C	A/A
Control-0872	2	62	0	0	0	C/T	C/C	G/A
Control-0873	2	76	0	0	1	C/T	G/C	A/A
Control-0874	2	56	0	0	0	T/T	C/C	G/A
Control-0875	2	55	0	0	1	C/T	C/C	A/A
Control-0876	2	62	0	0	0	C/T	G/C	G/G
Control-0877	2	62	0	0	1	C/T	C/C	A/A
Control-0878	1	57	0	0	0	C/T	G/C	A/A
Control-0879	1	52	0	0	0	?	?	?
Control-0880	1	54	0	0	1	T/T	C/C	G/G
Control-0881	1	56	0	0	1	T/T	C/C	A/A
Control-0882	1	52	0	0	0	C/T	G/C	A/A
Control-0883	1	50	0	0	0	C/T	C/C	A/A
Control-0884	1	62	0	0	0	C/T	G/C	A/A
Control-0885	1	40	0	0	1	C/T	C/C	A/A
Control-0886	1	49	0	0	1	T/T	G/C	A/A
Control-0887	1	53	0	0	1	C/C	G/C	G/G
Control-0888	1	45	0	0	0	C/T	G/G	G/G
Control-0889	1	48	0	0	0	T/T	G/G	A/A
Control-0890	1	43	0	0	0	C/C	C/C	G/G
Control-0891	1	61	0	0	1	C/C	G/G	A/A
Control-0892	1	51	0	0	1	C/T	G/C	G/G
Control-0893	1	69	0	0	1	C/T	G/C	A/A
Control-0894	1	55	0	0	1	C/T	G/G	A/A
Control-0895	2	50	0	0	1	T/T	G/C	G/A
Control-0896	2	56	0	0	1	C/T	G/G	A/A
Control-0897	1	53	1	1	0	C/T	C/C	A/A
Control-0898	2	55	0	0	0	T/T	G/C	A/A
Control-0899	1	62	0	0	1	C/T	G/C	A/A
Control-0900	1	49	0	0	1	T/T	G/G	A/A
Control-0901	1	63	0	0	0	T/T	C/C	A/A
Control-0902	2	61	0	0	1	T/T	G/C	A/A
Control-0903	1	51	0	0	1	T/T	G/C	A/A
Control-0904	1	47	0	0	0	C/T	G/G	A/A
Control-0905	1	49	0	0	0	T/T	G/C	G/A
Control-0906	2	62	0	0	1	C/T	G/G	A/A
Control-0907	2	68	0	0	1	C/T	C/C	A/A

Control-0908	1	40	0	0	0	C/T	C/C	A/A
Control-0909	1	59	0	0	0	T/T	G/C	G/A
Control-0910	1	49	0	0	0	C/C	C/C	G/A
Control-0911	1	63	0	0	0	C/C	C/C	G/A
Control-0912	1	49	0	0	1	C/T	C/C	G/A
Control-0913	2	67	0	0	1	C/T	C/C	G/A
Control-0914	1	66	0	0	0	C/T	G/C	G/A
Control-0915	1	42	1	1	0	T/T	G/G	A/A
Control-0916	1	41	0	0	0	T/T	G/G	A/A
Control-0917	2	56	0	0	1	T/T	G/G	G/A
Control-0918	1	60	1	1	0	C/T	G/C	G/A
Control-0919	1	65	1	0	0	C/C	C/C	A/A
Control-0920	1	55	0	0	0	T/T	C/C	A/A
Control-0921	1	63	1	1	0	T/T	C/C	A/A
Control-0922	2	40	0	0	0	C/T	C/C	A/A
Control-0923	1	50	0	1	0	C/C	C/C	A/A
Control-0924	1	50	1	0	0	C/T	G/C	A/A
Control-0925	2	53	0	0	1	T/T	G/C	G/A
Control-0926	2	55	0	0	0	C/T	G/C	A/A
Control-0927	2	57	0	0	1	T/T	C/C	A/A
Control-0928	2	56	0	0	0	C/T	C/C	A/A
Control-0929	2	60	0	0	1	C/T	C/C	G/A
Control-0930	2	51	0	0	1	C/T	G/G	A/A
Control-0931	2	61	0	0	1	C/C	C/C	A/A
Control-0932	2	52	0	0	0	C/T	G/C	G/A
Control-0933	2	56	0	0	0	T/T	G/C	A/A
Control-0934	2	60	0	0	0	T/T	G/C	G/G
Control-0935	2	58	0	0	0	C/T	C/C	A/A
Control-0936	2	59	0	0	0	C/T	C/C	A/A
Control-0937	2	60	0	0	1	C/T	G/C	A/A
Control-0938	2	53	0	0	1	T/T	C/C	A/A
Control-0939	2	56	0	0	0	T/T	G/C	A/A
Control-0940	2	61	0	0	1	C/C	G/C	A/A
Control-0941	2	59	0	0	0	C/T	G/C	G/A
Control-0942	2	57	0	0	0	T/T	C/C	A/A
Control-0943	2	60	0	0	0	C/T	C/C	A/A
Control-0944	2	32	0	0	0	T/T	C/C	G/A
Control-0945	2	50	0	0	1	C/T	C/C	A/A
Control-0946	2	56	0	0	0	C/T	G/G	G/A
Control-0947	2	60	0	0	1	C/C	G/C	A/A
Control-0948	2	58	0	0	1	T/T	C/C	A/A
Control-0949	2	50	0	0	1	C/C	C/C	A/A
Control-0950	2	52	0	0	1	T/T	G/C	G/A
Control-0951	1	69	0	0	0	C/T	C/C	A/A
Control-0952	2	79	0	0	1	C/T	G/C	G/A
Control-0953	1	63	0	0	1	C/T	G/G	A/A
Control-0954	1	61	0	0	0	C/C	C/C	G/A
Control-0955	1	55	0	0	1	C/T	G/C	A/A
Control-0956	1	77	0	0	0	C/C	C/C	A/A
Control-0957	1	63	0	0	0	T/T	G/G	G/G
Control-0958	2	61	0	0	1	C/T	G/C	A/A
Control-0959	2	69	0	0	0	C/T	C/C	A/A
Control-0960	2	47	0	0	0	C/T	C/C	A/A
Control-0961	1	46	0	0	0	T/T	C/C	G/A

Control-0962	1	65	1	1	0	C/T	G/C	G/A
Control-0963	1	50	0	0	1	C/C	G/C	G/A
Control-0964	2	76	0	0	1	T/T	G/C	A/A
Control-0965	2	53	0	0	1	C/T	G/C	A/A
Control-0966	1	65	0	0	1	C/T	G/C	A/A
Control-0967	1	38	0	0	1	C/C	C/C	A/A
Control-0968	2	46	0	0	0	C/T	G/G	A/A
Control-0969	1	32	1	1	0	C/C	C/C	G/A
Control-0970	2	69	0	0	0	C/T	C/C	G/A
Control-0971	1	74	0	0	0	C/T	G/G	A/A
Control-0972	1	58	1	0	1	C/T	C/C	A/A
Control-0973	2	45	0	0	0	C/T	G/C	A/A
Control-0974	1	61	1	0	1	T/T	G/C	A/A
Control-0975	1	34	0	0	0	C/C	C/C	A/A
Control-0976	1	68	0	0	0	C/T	G/C	A/A
Control-0977	2	47	0	0	1	C/C	G/C	G/A
Control-0978	2	30	0	0	0	C/T	C/C	A/A
Control-0979	1	57	0	1	0	C/T	G/C	A/A
Control-0980	2	46	0	0	1	T/T	C/C	A/A
Control-0981	2	58	0	0	0	C/T	C/C	A/A
Control-0982	1	44	1	1	1	T/T	G/G	G/A
Control-0983	1	58	0	1	1	?	?	?
Control-0984	1	61	1	0	0	C/T	C/C	G/A
Control-0985	1	57	0	0	0	T/T	G/C	G/A
Control-0986	2	63	0	0	1	C/T	G/C	G/A
Control-0987	2	49	1	1	0	T/T	G/C	A/A
Control-0988	2	64	0	0	0	C/T	C/C	A/A
Control-0989	2	36	0	0	0	C/T	C/C	A/A
Control-0990	1	59	0	0	0	C/T	G/G	A/A
Control-0991	2	44	0	0	0	C/T	G/C	G/A
Control-0992	2	62	0	0	0	C/C	C/C	A/A
Control-0993	2	73	0	0	1	C/T	G/C	G/A
Control-0994	1	60	1	0	0	T/T	C/C	A/A
Control-0995	2	62	0	0	0	C/T	G/C	G/A
Control-0996	1	51	1	0	1	C/T	C/C	A/A
Control-0997	1	73	0	0	1	C/T	C/C	G/A
Control-0998	1	75	0	0	0	C/C	C/C	A/A
Control-0999	2	47	0	0	1	C/C	G/C	G/A
Control-1000	1	44	0	0	0	C/C	C/C	A/A
Control-1001	1	79	0	0	0	T/T	C/C	G/A
Control-1002	2	65	0	0	1	C/T	C/C	A/A
Control-1003	1	68	0	0	0	C/T	C/C	A/A
Control-1004	2	43	0	0	0	C/C	G/C	A/A
Control-1005	1	79	0	0	1	C/T	G/C	A/A
Control-1006	2	51	0	0	0	T/T	G/C	A/A
Control-1007	1	39	0	0	1	C/T	C/C	A/A
Control-1008	2	68	0	0	0	T/T	G/C	A/A
Control-1009	2	75	0	0	0	?	?	?
Control-1010	2	63	0	0	0	T/T	G/C	G/A
Control-1011	2	44	0	0	1	C/T	G/C	A/A
Control-1012	1	60	0	0	0	T/T	C/C	A/A
Control-1013	1	86	0	0	0	C/T	G/C	A/A
Control-1014	1	47	0	0	0	C/C	C/C	A/A
Control-1015	2	41	0	1	1	T/T	G/C	A/A

Control-1016	2	79	0	0	0	C/T	G/G	A/A
Control-1017	1	67	1	1	0	C/T	C/C	A/A
Control-1018	1	46	0	0	0	C/C	G/C	A/A
Control-1019	2	56	0	0	1	C/T	G/C	G/A
Control-1020	2	74	0	0	0	C/C	C/C	G/G
Control-1021	1	76	0	0	0	T/T	C/C	G/A
Control-1022	1	44	1	0	0	C/T	G/C	G/A
Control-1023	1	43	0	0	1	T/T	C/C	G/A
Control-1024	1	74	1	1	1	C/C	G/C	G/A
Control-1025	2	68	0	0	0	C/C	G/C	A/A
Control-1026	1	82	0	0	0	T/T	C/C	G/A
Control-1027	2	78	0	0	1	C/C	C/C	A/A
Control-1028	1	64	1	0	0	C/C	C/C	A/A
Control-1029	1	36	1	1	0	C/C	G/G	A/A
Control-1030	1	35	0	0	1	C/T	C/C	A/A
Control-1031	1	63	0	0	0	T/T	G/C	G/A
Control-1032	2	74	0	0	0	C/T	G/C	A/A
Control-1033	2	63	0	0	0	C/T	G/C	A/A
Control-1034	2	70	0	0	1	C/C	C/C	A/A
Control-1035	1	63	0	0	0	C/T	G/C	A/A
Control-1036	1	65	0	0	0	C/T	G/C	A/A
Control-1037	1	80	0	0	1	T/T	G/C	G/A
Control-1038	1	74	1	1	0	C/T	C/C	G/A
Control-1039	1	55	0	0	1	C/C	G/C	A/A
Control-1040	1	24	0	0	0	C/T	G/G	A/A
Control-1041	2	71	0	0	0	C/T	G/C	A/A
Control-1042	1	58	0	0	1	C/T	G/C	G/A
Control-1043	1	53	1	1	0	C/T	G/C	G/G
Control-1044	2	60	0	0	0	C/T	G/C	A/A
Control-1045	1	78	0	0	1	C/C	C/C	A/A
Control-1046	1	69	1	1	0	T/T	G/C	A/A
Control-1047	1	53	0	0	1	T/T	G/G	A/A
Control-1048	1	49	0	0	0	T/T	C/C	A/A
Control-1049	2	78	0	0	1	C/T	G/G	A/A
Control-1050	1	38	0	0	0	C/T	C/C	G/A
Control-1051	1	47	1	1	0	C/T	G/C	A/A
Control-1052	1	77	0	0	0	C/T	G/G	A/A
Control-1053	2	62	0	0	0	C/T	C/C	A/A
Control-1054	2	67	0	0	0	T/T	G/G	A/A
Control-1055	1	54	1	0	1	C/C	C/C	A/A
Control-1056	1	64	0	0	1	C/C	G/G	G/G

Table S2 Detailed information and genotypes for SCC cases and controls

SUBJECTS	SEX(1: male, 2: female)	AGE (Years)	SMOKING (1: Yes, 2: No)	DRINKING (1: Yes, 2: No)	BMI (1: ≥ $24kg/m^2$, 0: < $24kg/m^2$)	PATHOLOGY	rs11614913	rs2910164	rs3746444
SCC001	1	67	1	0	0	SCC	C/C	G/C	G/A
SCC002	2	82	0	0	0	SCC	T/T	C/C	A/A
SCC003	1	53	0	0	0	SCC	C/T	G/G	A/A
SCC004	1	65	1	0	0	SCC	C/T	G/C	A/A
SCC005	1	77	0	0	0	SCC	C/T	G/C	G/A
SCC006	2	43	0	0	0	SCC	C/T	G/C	A/A
SCC007	1	71	1	1	0	SCC	C/T	C/C	A/A
SCC008	1	59	1	1	0	SCC	C/T	G/C	A/A
SCC009	2	64	1	1	0	SCC	T/T	C/C	A/A
SCC010	1	71	1	0	0	SCC	C/T	G/C	A/A
SCC011	1	53	1	1	0	SCC	C/T	G/C	A/A
SCC012	1	50	1	1	0	SCC	C/T	C/C	A/A
SCC013	1	58	1	0	1	SCC	T/T	G/C	A/A
SCC014	1	56	1	1	1	SCC	T/T	C/C	A/A
SCC015	1	75	1	0	1	SCC	C/T	G/C	G/A
SCC016	1	65	1	0	0	SCC	T/T	G/C	A/A
SCC017	1	61	1	0	0	SCC	T/T	G/C	A/A
SCC018	1	67	1	0	0	SCC	T/T	C/C	A/A
SCC019	1	65	1	0	0	SCC	T/T	C/C	A/A
SCC020	1	68	1	0	0	SCC	T/T	C/C	A/A
SCC021	1	56	1	0	0	SCC	C/C	C/C	G/A
SCC022	1	57	1	0	1	SCC	C/C	C/C	A/A
SCC023	1	64	1	0	1	SCC	C/C	C/C	G/A
SCC024	1	71	1	0	0	SCC	C/T	G/C	G/A
SCC025	1	78	1	0	0	SCC	C/T	G/G	G/A
SCC026	1	63	1	0	0	SCC	C/T	C/C	A/A
SCC027	1	57	1	0	0	SCC	T/T	G/C	A/A
SCC028	1	46	1	0	0	SCC	C/T	G/C	G/A
SCC029	1	67	1	0	0	SCC	C/T	C/C	A/A
SCC030	1	52	1	0	1	SCC	C/T	G/G	A/A
SCC031	1	63	1	0	0	SCC	T/T	C/C	A/A
SCC032	1	54	1	0	0	SCC	T/T	C/C	A/A
SCC033	1	51	1	0	0	SCC	C/C	G/C	A/A
SCC034	1	63	1	0	0	SCC	C/T	G/G	A/A
SCC035	1	58	1	0	1	SCC	T/T	G/G	A/A
SCC036	1	67	1	1	0	SCC	C/T	C/C	A/A
SCC037	1	62	1	0	0	SCC	C/T	C/C	G/A
SCC038	1	66	1	0	0	SCC	C/T	C/C	G/A
SCC039	1	53	1	0	1	SCC	T/T	C/C	A/A
SCC040	1	53	1	0	1	SCC	T/T	G/G	G/G
SCC041	2	72	0	0	0	SCC	C/T	C/C	A/A
SCC042	1	53	1	0	0	SCC	C/C	G/C	A/A
SCC043	1	64	1	0	0	SCC	C/T	C/C	G/G
SCC044	1	76	0	0	0	SCC	C/T	G/C	A/A

SCC045	1	46	1	0	0	SCC	T/T	C/C	G/A
SCC046	1	64	0	1	0	SCC	C/C	C/C	A/A
SCC047	1	77	1	0	0	SCC	C/C	C/C	G/A
SCC048	1	62	1	1	1	SCC	C/T	G/C	G/A
SCC049	1	87	1	1	1	SCC	T/T	G/C	A/A
SCC050	1	65	1	0	0	SCC	T/T	C/C	A/A
SCC051	1	54	1	0	1	SCC	C/T	C/C	G/A
SCC052	1	67	1	1	0	SCC	C/T	C/C	G/A
SCC053	2	69	0	0	1	SCC	T/T	G/C	A/A
SCC054	1	55	1	1	0	SCC	C/C	G/C	A/A
SCC055	1	68	1	1	1	SCC	T/T	G/C	A/A
SCC056	1	55	0	0	0	SCC	C/T	G/C	A/A
SCC057	1	62	1	0	0	SCC	C/T	G/C	G/A
SCC058	2	67	0	0	1	SCC	C/T	G/C	A/A
SCC059	1	69	1	1	0	SCC	T/T	C/C	G/A
SCC060	1	51	1	0	0	SCC	T/T	G/G	A/A
SCC061	1	62	1	0	1	SCC	C/T	C/C	G/A
SCC062	1	55	1	1	1	SCC	C/T	G/C	A/A
SCC063	1	43	1	1	0	SCC	C/T	C/C	G/A
SCC064	1	62	1	0	0	SCC	C/T	G/G	A/A
SCC065	1	61	1	0	0	SCC	C/T	C/C	A/A
SCC066	1	61	1	1	0	SCC	C/C	G/C	G/A
SCC067	2	64	0	0	1	SCC	T/T	G/G	G/A
SCC068	1	76	0	0	0	SCC	C/C	G/G	A/A
SCC069	1	71	1	1	0	SCC	C/T	G/C	A/A
SCC070	1	80	1	0	0	SCC	T/T	C/C	G/A
SCC071	1	64	1	1	1	SCC	C/T	G/G	A/A
SCC072	1	65	1	1	0	SCC	T/T	G/C	A/A
SCC073	1	64	1	0	0	SCC	C/C	G/C	A/A
SCC074	1	77	1	0	0	SCC	C/T	G/C	G/A
SCC075	1	60	1	0	0	SCC	C/T	G/C	A/A
SCC076	2	55	0	0	0	SCC	T/T	G/C	A/A
SCC077	1	77	0	0	0	SCC	T/T	G/C	A/A
SCC078	1	78	1	0	0	SCC	C/C	G/C	G/A
SCC079	1	70	1	0	0	SCC	C/T	G/C	A/A
SCC080	1	81	0	0	0	SCC	C/T	G/G	A/A
SCC081	1	58	1	0	0	SCC	C/T	G/C	A/A
SCC082	1	68	0	0	0	SCC	T/T	C/C	A/A
SCC083	1	70	1	1	0	SCC	T/T	G/C	A/A
SCC084	2	56	0	0	0	SCC	C/T	G/C	A/A
SCC085	1	51	1	0	1	SCC	C/C	C/C	A/A
SCC086	1	48	1	0	0	SCC	C/T	G/C	A/A
SCC087	1	57	1	0	0	SCC	C/T	G/C	A/A
SCC088	1	66	0	0	0	SCC	C/C	G/G	G/A
SCC089	1	62	0	0	0	SCC	T/T	G/C	G/A
SCC090	1	44	1	1	0	SCC	C/T	C/C	A/A
SCC091	1	55	1	0	0	SCC	C/C	C/C	A/A
SCC092	1	64	1	1	0	SCC	T/T	C/C	A/A

SCC093	2	65	0	0	0	SCC	C/T	G/C	A/A
SCC094	1	62	0	0	0	SCC	C/T	G/C	A/A
SCC095	1	70	1	1	0	SCC	C/T	G/G	A/A
SCC096	1	62	1	0	0	SCC	T/T	G/C	A/A
SCC097	1	53	1	0	0	SCC	T/T	G/G	A/A
SCC098	1	75	1	1	1	SCC	T/T	C/C	A/A
SCC099	1	59	1	1	1	SCC	T/T	G/C	A/A
SCC100	1	80	1	0	0	SCC	C/T	G/C	A/A
SCC101	1	50	1	1	1	SCC	T/T	C/C	A/A
SCC102	1	71	1	1	0	SCC	C/T	C/C	A/A
SCC103	1	64	1	1	1	SCC	C/C	G/C	A/A
SCC104	1	48	1	1	0	SCC	C/T	C/C	G/A
SCC105	2	46	0	0	1	SCC	C/C	C/C	A/A
SCC106	1	59	1	1	1	SCC	C/T	G/C	A/A
SCC107	1	65	1	1	0	SCC	C/T	C/C	G/A
SCC108	1	55	1	0	0	SCC	T/T	G/C	G/A
SCC109	1	60	1	0	0	SCC	C/T	C/C	G/A
SCC110	2	53	0	0	0	SCC	C/T	C/C	A/A
SCC111	1	72	1	1	0	SCC	C/C	G/C	A/A
SCC112	1	59	1	1	0	SCC	C/C	C/C	A/A
SCC113	1	62	1	1	0	SCC	C/C	G/C	A/A
SCC114	1	57	1	1	0	SCC	T/T	C/C	G/A
SCC115	1	51	1	0	0	SCC	C/T	C/C	A/A
SCC116	2	61	0	0	0	SCC	C/T	G/C	A/A
SCC117	1	74	1	0	1	SCC	T/T	G/C	A/A
SCC118	1	52	1	1	1	SCC	C/C	G/C	A/A
SCC119	1	53	1	1	0	SCC	C/T	G/G	A/A
SCC120	1	76	1	0	0	SCC	C/C	G/G	A/A
SCC121	1	73	1	0	0	SCC	T/T	G/C	G/A
SCC122	1	59	1	0	0	SCC	C/T	C/C	A/A
SCC123	1	62	1	0	1	SCC	C/T	G/C	G/A
SCC124	1	49	1	1	0	SCC	T/T	G/C	A/A
SCC125	1	52	1	0	0	SCC	C/T	G/C	A/A
SCC126	1	57	1	0	0	SCC	T/T	G/C	A/A
SCC127	1	69	0	0	0	SCC	C/T	C/C	A/A
SCC128	1	57	1	1	0	SCC	T/T	C/C	A/A
SCC129	1	56	0	0	0	SCC	C/C	G/G	A/A
SCC130	1	55	1	1	0	SCC	C/T	G/C	G/G
SCC131	1	62	1	1	0	SCC	T/T	C/C	G/A
SCC132	1	63	0	0	1	SCC	C/T	G/C	A/A
SCC133	1	67	1	1	0	SCC	C/C	G/C	G/A
SCC134	1	66	1	0	0	SCC	T/T	G/C	A/A
SCC135	1	72	1	1	0	SCC	C/T	G/C	A/A
SCC136	1	66	1	1	0	SCC	C/T	G/C	A/A
SCC137	1	57	1	1	0	SCC	C/T	G/C	A/A
SCC138	1	65	1	1	0	SCC	C/T	G/C	G/A
SCC139	1	73	1	0	0	SCC	T/T	G/C	A/A
SCC140	1	77	1	0	0	SCC	T/T	G/G	G/A

SCC141	2	52	0	0	0	SCC	C/T	C/C	A/A
SCC142	1	59	1	1	0	SCC	C/T	C/C	A/A
SCC143	1	64	0	0	0	SCC	C/T	G/C	G/A
SCC144	1	74	1	1	1	SCC	T/T	C/C	A/A
SCC145	1	58	1	1	0	SCC	C/T	C/C	A/A
SCC146	1	65	0	1	0	SCC	T/T	C/C	A/A
SCC147	1	66	1	1	0	SCC	C/T	G/C	A/A
SCC148	1	60	1	0	1	SCC	T/T	C/C	A/A
SCC149	1	56	1	0	0	SCC	T/T	C/C	A/A
SCC150	1	66	1	1	0	SCC	?	?	?
SCC151	1	59	1	1	0	SCC	T/T	C/C	A/A
SCC152	1	57	1	0	1	SCC	C/T	G/C	A/A
SCC153	1	68	0	0	0	SCC	T/T	G/C	A/A
SCC154	1	64	1	1	0	SCC	T/T	G/G	A/A
SCC155	1	48	0	0	0	SCC	C/T	G/C	A/A
SCC156	1	64	0	0	0	SCC	C/T	G/G	A/A
SCC157	1	56	1	0	0	SCC	C/T	G/C	G/G
SCC158	1	62	1	1	0	SCC	C/C	G/C	A/A
SCC159	1	60	1	0	0	SCC	C/T	G/C	A/A
SCC160	1	57	1	1	0	SCC	C/C	G/C	A/A
SCC161	1	61	0	0	1	SCC	C/C	G/C	G/A
SCC162	1	74	0	0	0	SCC	C/C	G/C	G/A
SCC163	1	60	1	0	1	SCC	T/T	C/C	G/A
SCC164	1	52	1	0	0	SCC	T/T	C/C	G/A
SCC165	1	71	1	1	1	SCC	T/T	C/C	A/A
SCC166	1	55	0	0	1	SCC	C/T	G/C	A/A
SCC167	1	68	1	1	0	SCC	C/T	C/C	A/A
SCC168	1	49	0	0	0	SCC	T/T	G/C	G/G
SCC169	2	73	0	0	0	SCC	C/C	G/C	A/A
SCC170	1	70	1	0	0	SCC	C/T	G/C	A/A
SCC171	1	62	1	0	1	SCC	C/T	G/C	G/A
SCC172	1	51	1	1	0	SCC	C/C	G/C	A/A
SCC173	1	55	1	1	0	SCC	C/T	G/C	A/A
SCC174	1	55	1	1	0	SCC	C/C	G/C	A/A
SCC175	2	53	0	0	0	SCC	C/T	C/C	A/A
SCC176	1	70	1	1	0	SCC	C/T	C/C	G/A
SCC177	1	57	1	0	0	SCC	C/T	C/C	?
SCC178	1	54	1	0	0	SCC	C/T	G/G	A/A
SCC179	1	72	1	1	0	SCC	C/T	G/C	G/A
SCC180	1	71	1	1	0	SCC	C/T	C/C	A/A
SCC181	1	72	1	0	0	SCC	T/T	G/C	G/A
SCC182	2	60	1	0	0	SCC	C/T	G/C	G/A
control-0001	1	55	0	0	1		C/T	G/G	A/A
control-0002	2	53	0	0	1		T/T	G/C	A/A
control-0003	1	59	0	0	1		T/T	G/C	A/A
control-0004	2	54	0	0	0		C/T	G/C	G/A
control-0005	2	70	0	0	1		C/T	C/C	G/A
control-0006	2	56	0	0	0		C/C	G/G	A/A

control-0007	1	51	0	0	1	T/T	C/C	G/A
control-0008	2	61	0	0	0	C/T	G/C	A/A
control-0009	1	57	1	0	1	T/T	G/C	A/A
control-0010	1	53	1	0	0	C/C	G/C	G/A
control-0011	1	56	0	1	1	C/T	G/C	A/A
control-0012	1	64	1	1	1	T/T	G/G	A/A
control-0013	1	62	0	0	1	C/T	C/C	A/A
control-0014	1	66	0	0	1	C/T	G/G	A/A
control-0015	1	29	1	0	1	C/T	G/G	G/A
control-0016	1	38	0	0	1	C/T	C/C	A/A
control-0017	1	60	1	0	1	T/T	C/C	A/A
control-0018	2	53	0	0	1	T/T	C/C	A/A
control-0019	1	58	0	0	0	T/T	G/C	A/A
control-0020	2	52	0	0	0	T/T	G/C	A/A
control-0021	2	38	0	0	0	T/T	G/C	A/A
control-0022	1	28	0	0	0	T/T	G/C	G/A
control-0023	2	65	0	0	1	C/T	C/C	A/A
control-0024	2	52	0	0	0	T/T	C/C	A/A
control-0025	2	46	0	0	0	C/T	G/G	G/A
control-0026	1	56	0	0	1	C/T	G/C	A/A
control-0027	2	68	0	0	1	T/T	C/C	A/A
control-0028	1	59	0	0	0	C/T	G/C	A/A
control-0029	1	62	0	0	1	C/T	C/C	A/A
control-0030	1	71	0	0	0	C/C	G/C	A/A
control-0031	1	60	1	1	0	C/T	G/C	A/A
control-0032	1	56	1	0	0	C/T	C/C	G/A
control-0033	1	60	0	0	0	C/T	G/C	A/A
control-0034	2	72	0	0	0	C/T	G/C	A/A
control-0035	2	61	0	0	0	C/C	G/C	A/A
control-0036	2	66	0	0	0	C/T	G/C	A/A
control-0037	1	61	1	0	0	C/C	C/C	A/A
control-0038	2	54	0	0	0	C/T	G/C	G/A
control-0039	2	56	0	0	0	C/T	G/G	A/A
control-0040	2	71	0	0	1	T/T	G/C	A/A
control-0041	1	72	0	0	0	C/T	G/C	A/A
control-0042	1	46	0	0	0	C/T	G/G	A/A
control-0043	1	62	1	0	0	C/T	C/C	A/A
control-0044	2	59	0	0	0	C/T	C/C	A/A
control-0045	2	60	0	0	1	C/T	G/G	A/A
control-0046	2	52	0	0	1	C/C	C/C	A/A
control-0047	1	56	1	0	0	T/T	G/C	A/A
control-0048	1	59	1	0	0	T/T	G/C	G/A
control-0049	1	61	0	0	1	C/T	C/C	A/A
control-0050	1	63	0	0	1	T/T	C/C	A/A
control-0051	1	72	0	0	0	C/T	G/C	G/A
control-0052	1	64	0	1	0	C/T	C/C	A/A
control-0053	2	67	0	0	1	C/C	C/C	A/A
control-0054	1	58	1	0	0	C/T	C/C	A/A

control-0055	2	61	0	0	0	C/T	G/C	A/A
control-0056	1	41	1	0	1	C/T	G/C	A/A
control-0057	2	69	0	0	0	C/C	G/G	A/A
control-0058	1	62	0	0	0	C/C	G/C	A/A
control-0059	1	81	0	0	0	C/T	C/C	A/A
control-0060	1	57	0	0	0	T/T	C/C	G/A
control-0061	2	51	0	0	1	C/T	G/C	A/A
control-0062	1	55	1	0	0	C/C	G/C	A/A
control-0063	2	37	0	0	0	T/T	C/C	A/A
control-0064	1	65	1	1	0	C/T	G/C	A/A
control-0065	1	57	0	0	1	C/T	G/C	G/A
control-0066	1	80	0	0	0	C/C	G/C	A/A
control-0067	2	49	0	0	0	C/C	G/C	A/A
control-0068	2	46	0	0	0	C/T	G/G	A/A
control-0069	2	64	0	0	1	C/C	G/C	G/A
control-0070	2	65	0	0	0	C/C	G/C	A/A
control-0071	1	78	0	0	1	C/T	C/C	A/A
control-0072	1	43	0	0	0	C/C	G/G	A/A
control-0073	1	64	0	0	0	C/C	C/C	G/A
control-0074	2	56	0	0	1	C/T	G/C	G/A
control-0075	2	52	0	0	1	T/T	C/C	A/A
control-0076	2	57	0	0	0	C/T	C/C	A/A
control-0077	2	67	0	0	0	C/T	G/G	G/A
control-0078	2	53	0	0	0	C/T	G/C	A/A
control-0079	1	48	1	0	1	C/T	C/C	A/A
control-0080	1	57	1	0	0	C/C	C/C	A/A
control-0081	1	62	1	0	1	C/T	G/C	A/A
control-0082	2	71	0	0	0	C/C	G/C	A/A
control-0083	2	53	0	0	0	C/T	G/C	A/A
control-0084	2	49	0	0	1	C/T	G/C	G/A
control-0085	2	61	0	0	0	C/T	G/C	A/A
control-0086	2	62	0	0	1	T/T	C/C	A/A
control-0087	1	53	1	0	1	T/T	G/C	A/A
control-0088	1	62	0	0	1	C/C	G/C	G/A
control-0089	1	63	0	0	0	C/T	G/C	A/A
control-0090	1	33	0	0	0	C/T	G/G	A/A
control-0091	1	81	0	0	1	C/T	G/G	A/A
control-0092	2	59	0	0	1	C/T	C/C	A/A
control-0093	2	70	0	0	0	C/T	G/C	G/A
control-0094	2	56	0	0	0	C/T	C/C	A/A
control-0095	1	58	0	0	0	C/C	G/C	A/A
control-0096	1	60	1	0	0	C/T	C/C	A/A
control-0097	1	59	0	0	1	C/C	C/C	A/A
control-0098	1	76	1	0	0	C/C	C/C	G/G
control-0099	1	52	0	0	0	C/T	G/G	A/A
control-0100	2	34	0	0	0	C/C	C/C	A/A
control-0101	1	60	1	0	1	C/C	C/C	A/A
control-0102	1	76	1	0	0	C/T	G/C	A/A

control-0103	2	50	0	0	1	T/T	G/C	G/A
control-0104	1	57	0	0	0	C/C	G/G	A/A
control-0105	1	51	0	0	0	C/C	C/C	G/G
control-0106	1	60	0	0	0	T/T	C/C	A/A
control-0107	2	65	0	0	1	C/C	G/G	A/A
control-0108	1	55	0	0	1	T/T	G/C	G/A
control-0109	1	38	1	0	0	C/T	C/C	A/A
control-0110	1	64	0	0	1	T/T	G/G	A/A
control-0111	1	62	0	0	0	C/T	G/G	A/A
control-0112	1	54	1	0	0	T/T	C/C	G/A
control-0113	1	64	0	0	0	C/T	C/C	A/A
control-0114	1	56	0	0	0	T/T	G/G	A/A
control-0115	2	60	0	0	1	C/T	C/C	A/A
control-0116	1	64	0	0	0	C/C	G/C	A/A
control-0117	1	58	0	0	0	C/T	C/C	A/A
control-0118	1	46	0	0	0	T/T	G/G	A/A
control-0119	2	72	0	0	0	C/T	G/C	G/A
control-0120	1	44	1	0	1	C/T	C/C	A/A
control-0121	2	41	0	0	0	C/C	G/G	A/A
control-0122	2	67	0	0	0	T/T	C/C	G/A
control-0123	2	65	0	0	0	T/T	G/G	G/A
control-0124	2	66	0	0	1	T/T	G/C	G/A
control-0125	1	61	1	0	0	T/T	C/C	A/A
control-0126	1	52	0	0	0	C/T	C/C	A/A
control-0127	2	67	0	0	1	C/T	G/G	A/A
control-0128	1	60	0	0	1	C/T	G/G	A/A
control-0129	1	56	1	0	0	C/C	C/C	A/A
control-0130	1	60	0	0	1	C/T	C/C	G/A
control-0131	1	57	0	0	1	C/C	C/C	A/A
control-0132	2	65	0	0	0	C/T	G/G	A/A
control-0133	2	70	0	0	0	C/T	G/C	G/G
control-0134	2	69	0	0	1	C/T	G/C	G/A
control-0135	1	53	1	0	0	C/T	C/C	A/A
control-0136	2	25	0	0	0	T/T	G/C	A/A
control-0137	2	43	0	0	0	C/T	C/C	A/A
control-0138	1	62	1	0	1	C/T	C/C	G/A
control-0139	2	74	0	0	1	C/T	C/C	G/A
control-0140	2	73	0	0	0	T/T	G/C	G/G
control-0141	2	62	0	0	0	C/T	C/C	A/A
control-0142	1	58	0	0	0	T/T	C/C	A/A
control-0143	2	69	0	0	1	C/T	G/G	A/A
control-0144	2	51	0	0	0	C/C	G/C	A/A
control-0145	1	61	0	0	1	C/C	C/C	A/A
control-0146	1	74	0	0	0	C/T	G/C	A/A
control-0147	1	55	0	0	1	C/T	G/C	A/A
control-0148	2	72	0	0	0	C/T	C/C	A/A
control-0149	1	56	0	0	0	C/T	G/C	A/A
control-0150	2	78	0	0	1	C/T	G/G	G/A

control-0151	2	63	0	0	1	C/T	C/C	G/A
control-0152	2	61	0	0	1	C/T	G/C	A/A
control-0153	1	59	1	0	1	T/T	C/C	A/A
control-0154	2	63	0	0	1	C/T	G/C	A/A
control-0155	2	39	0	0	1	T/T	G/C	A/A
control-0156	1	58	0	0	0	C/T	C/C	A/A
control-0157	1	54	0	0	0	T/T	G/G	A/A
control-0158	1	57	1	0	1	C/T	G/C	A/A
control-0159	1	66	0	0	0	T/T	C/C	G/A
control-0160	1	74	0	0	1	C/T	C/C	A/A
control-0161	2	50	0	0	0	T/T	G/C	A/A
control-0162	1	68	0	0	1	C/T	G/G	A/A
control-0163	1	60	0	0	1	T/T	C/C	G/A
control-0164	2	59	0	0	1	T/T	G/C	G/A
control-0165	1	73	0	0	1	C/C	G/C	A/A
control-0166	1	75	1	0	1	T/T	C/C	A/A
control-0167	1	65	0	0	0	C/C	C/C	G/A
control-0168	1	62	0	0	0	C/T	G/C	A/A
control-0169	1	57	0	0	0	T/T	C/C	A/A
control-0170	2	76	0	0	1	C/T	G/G	A/A
control-0171	2	60	0	0	1	T/T	G/C	A/A
control-0172	1	59	0	0	0	C/C	G/C	A/A
control-0173	2	52	0	0	0	C/T	G/C	A/A
control-0174	1	52	0	0	1	T/T	C/C	A/A
control-0175	1	76	0	0	1	C/C	G/C	A/A
control-0176	1	75	0	0	1	C/T	C/C	A/A
control-0177	2	51	0	0	1	C/T	C/C	A/A
control-0178	1	66	0	0	1	C/C	C/C	A/A
control-0179	2	61	0	0	0	C/T	C/C	A/A
control-0180	2	62	0	0	1	T/T	G/C	A/A
control-0181	1	40	0	0	1	C/T	G/C	A/A
control-0182	1	50	0	1	1	T/T	C/C	A/A
control-0183	2	59	0	0	0	T/T	C/C	G/A
control-0184	1	48	0	0	1	C/T	C/C	A/A
control-0185	1	74	0	1	1	T/T	C/C	A/A
control-0186	1	84	0	0	0	C/T	G/C	G/G
control-0187	1	57	0	0	0	C/C	C/C	G/A
control-0188	1	69	0	0	1	C/T	C/C	A/A
control-0189	1	62	0	0	1	C/T	C/C	A/A
control-0190	2	57	0	0	1	C/T	C/C	A/A
control-0191	1	37	0	0	0	C/T	G/C	A/A
control-0192	1	64	0	0	1	T/T	G/C	A/A
control-0193	1	64	0	0	0	C/T	G/G	A/A
control-0194	1	77	0	0	0	T/T	G/C	G/A
control-0195	1	60	0	0	0	C/T	C/C	A/A
control-0196	1	68	0	0	0	C/T	G/G	G/A
control-0197	1	81	0	0	1	C/C	G/C	G/A
control-0198	2	55	0	0	1	T/T	C/C	A/A

control-0199	1	79	1	1	0	T/T	C/C	G/A
control-0200	1	71	1	1	0	C/T	G/C	A/A
control-0201	1	64	0	0	0	C/T	G/C	A/A
control-0202	2	57	0	0	0	C/T	C/C	G/A
control-0203	2	78	0	0	1	C/C	G/G	G/A
control-0204	1	58	1	0	1	C/T	C/C	A/A
control-0205	1	74	0	0	0	C/C	G/C	A/A
control-0206	2	54	0	0	1	C/C	G/C	A/A
control-0207	1	59	0	1	0	C/T	C/C	A/A
control-0208	1	70	0	0	0	C/C	C/C	A/A
control-0209	1	55	0	0	1	C/C	G/C	G/A
control-0210	1	58	0	0	0	T/T	C/C	A/A
control-0211	1	59	0	0	1	T/T	G/G	A/A
control-0212	1	60	0	0	0	C/T	C/C	G/A
control-0213	1	58	0	0	0	T/T	G/C	A/A
control-0214	2	60	0	0	0	T/T	C/C	A/A
control-0215	2	61	0	0	0	C/T	C/C	A/A
control-0216	2	67	0	0	0	C/T	C/C	G/A
control-0217	1	59	1	0	1	C/T	C/C	A/A
control-0218	2	55	0	0	0	C/T	C/C	A/A
control-0219	1	66	0	0	0	T/T	G/C	A/A
control-0220	1	64	0	0	1	C/T	C/C	A/A
control-0221	1	56	1	0	1	C/C	C/C	A/A
control-0222	2	61	0	0	1	T/T	C/C	A/A
control-0223	1	60	1	0	0	C/T	C/C	G/A
control-0224	2	66	0	0	0	C/T	G/C	G/A
control-0225	2	63	0	0	1	C/T	G/C	G/A
control-0226	1	65	1	0	1	C/C	G/G	A/A
control-0227	1	60	0	0	0	C/T	C/C	G/A
control-0228	2	51	0	0	0	C/C	C/C	A/A
control-0229	1	57	0	0	1	C/T	G/C	A/A
control-0230	2	79	0	0	1	T/T	C/C	G/A
control-0231	1	55	1	1	0	C/T	G/C	A/A
control-0232	1	51	0	0	1	T/T	C/C	A/A
control-0233	2	66	0	0	0	C/T	C/C	A/A
control-0234	1	53	1	0	0	C/T	G/G	A/A
control-0235	1	61	0	0	1	C/C	G/C	G/G
control-0236	1	62	0	0	1	C/T	C/C	A/A
control-0237	1	60	1	0	1	T/T	G/C	G/A
control-0238	1	71	1	1	1	C/T	G/C	G/A
control-0239	1	67	0	0	0	C/C	C/C	A/A
control-0240	2	69	0	0	0	C/C	G/C	A/A
control-0241	1	62	0	0	0	T/T	G/C	A/A
control-0242	1	61	1	0	0	C/C	G/C	G/A
control-0243	1	62	1	0	0	C/T	G/C	A/A
control-0244	2	70	0	0	1	C/T	C/C	A/A
control-0245	2	56	0	0	1	C/C	G/C	A/A
control-0246	2	68	0	0	1	T/T	C/C	G/A

control-0247	2	53	0	0	1	C/T	G/C	A/A
control-0248	2	56	0	0	1	C/C	G/G	A/A
control-0249	2	48	0	0	0	C/C	G/C	A/A
control-0250	1	61	0	0	1	C/C	G/C	G/A
control-0251	1	67	0	0	1	C/T	G/C	A/A
control-0252	1	57	0	0	0	C/T	C/C	A/A
control-0253	1	63	0	0	0	C/T	G/C	A/A
control-0254	1	74	1	0	0	T/T	C/C	G/A
control-0255	1	67	0	0	0	C/T	C/C	A/A
control-0256	1	61	0	0	0	C/T	C/C	A/A
control-0257	1	74	0	1	0	T/T	G/C	G/A
control-0258	2	65	0	0	1	C/C	G/C	A/A
control-0259	1	71	1	0	0	T/T	C/C	A/A
control-0260	1	59	1	0	0	T/T	G/C	G/A
control-0261	1	69	1	0	0	C/T	G/G	A/A
control-0262	2	78	0	0	0	C/C	C/C	A/A
control-0263	1	59	0	0	1	C/T	G/C	A/A
control-0264	1	63	0	0	1	C/C	G/C	G/A
control-0265	2	63	0	0	1	T/T	G/C	G/A
control-0266	1	71	0	0	1	C/T	G/G	G/A
control-0267	1	64	0	1	0	C/T	G/G	A/A
control-0268	1	56	0	0	0	C/C	C/C	G/A
control-0269	2	72	0	0	1	C/T	C/C	A/A
control-0270	1	62	1	0	0	C/T	G/G	A/A
control-0271	2	51	0	0	0	C/T	G/G	A/A
control-0272	1	58	1	0	0	T/T	G/C	G/A
control-0273	1	60	0	0	0	T/T	G/C	G/A
control-0274	2	66	0	0	0	C/T	G/C	G/A
control-0275	2	70	0	0	0	C/T	C/C	G/A
control-0276	1	57	0	0	1	T/T	C/C	A/A
control-0277	2	61	0	0	0	C/T	C/C	G/A
control-0278	2	59	0	0	0	C/C	C/C	A/A
control-0279	2	54	0	0	0	C/T	C/C	A/A
control-0280	1	64	0	0	0	T/T	G/C	A/A
control-0281	2	48	0	0	1	C/T	G/C	A/A
control-0282	1	63	1	0	0	C/C	G/C	A/A
control-0283	2	69	0	0	0	T/T	G/C	A/A
control-0284	2	74	0	0	1	C/T	G/C	G/A
control-0285	1	59	0	0	1	C/C	G/G	A/A
control-0286	2	63	0	0	1	T/T	C/C	A/A
control-0287	1	73	0	0	1	C/C	C/C	G/A
control-0288	2	68	0	0	0	T/T	G/C	A/A
control-0289	1	65	0	0	0	T/T	G/G	G/A
control-0290	1	73	0	0	1	C/T	G/G	G/G
control-0291	2	59	0	0	0	C/T	G/G	A/A
control-0292	1	65	1	0	0	T/T	G/C	A/A
control-0293	1	66	0	0	0	T/T	C/C	G/A
control-0294	2	67	0	0	0	C/T	C/C	A/A

control-0295	2	55	0	0	0	C/C	C/C	A/A
control-0296	1	71	0	0	1	C/C	G/C	G/A
control-0297	2	67	0	0	0	C/C	C/C	A/A
control-0298	1	53	0	0	1	C/T	C/C	A/A
control-0299	2	67	0	0	1	T/T	C/C	A/A
control-0300	2	68	0	0	1	C/C	G/C	G/A
control-0301	1	55	1	0	0	C/T	G/C	A/A
control-0302	2	63	0	0	1	T/T	G/C	A/A
control-0303	2	72	0	0	0	C/T	G/C	A/A
control-0304	1	56	1	0	1	C/T	G/C	A/A
control-0305	1	56	0	0	1	C/T	G/C	A/A
control-0306	1	65	0	0	0	T/T	G/C	A/A
control-0307	1	59	0	0	1	C/C	G/C	A/A
control-0308	1	72	0	0	0	C/T	C/C	A/A
control-0309	1	59	1	0	1	C/C	C/C	A/A
control-0310	1	77	1	0	0	C/T	G/C	A/A
control-0311	1	60	0	0	0	T/T	C/C	G/A
control-0312	1	56	1	0	0	C/T	G/C	G/A
control-0313	1	62	0	0	0	T/T	C/C	A/A
control-0314	2	65	0	0	1	C/T	G/C	A/A
control-0315	1	49	1	0	0	C/T	G/C	A/A
control-0316	1	71	1	0	1	T/T	C/C	A/A
control-0317	1	52	0	0	0	C/T	G/C	A/A
control-0318	1	85	1	0	1	T/T	G/C	G/A
control-0319	1	47	0	0	0	T/T	C/C	G/A
control-0320	1	48	0	0	1	T/T	C/C	A/A
control-0321	2	46	0	0	0	C/T	C/C	A/A
control-0322	2	51	0	0	0	C/T	C/C	G/A
control-0323	1	67	1	0	1	T/T	G/C	A/A
control-0324	1	78	0	0	0	T/T	C/C	A/A
control-0325	1	48	1	0	0	T/T	C/C	G/A
control-0326	2	56	0	0	0	T/T	G/C	A/A
control-0327	2	51	0	0	1	C/T	G/G	A/A
control-0328	2	63	0	0	1	C/T	G/C	A/A
control-0329	2	54	0	0	1	C/T	C/C	A/A
control-0330	2	52	0	0	1	T/T	G/C	A/A
control-0331	1	52	0	0	1	C/T	G/C	G/A
control-0332	1	58	0	0	0	C/C	C/C	A/A
control-0333	1	51	0	0	0	C/T	G/C	A/A
control-0334	2	68	0	0	1	C/T	G/C	G/A
control-0335	1	52	0	0	0	C/C	C/C	A/A
control-0336	2	51	0	0	1	C/T	C/C	A/A
control-0337	2	66	0	0	1	T/T	C/C	A/A
control-0338	2	62	0	0	0	C/T	C/C	A/A
control-0339	1	66	0	0	0	T/T	G/C	G/A
control-0340	1	65	0	0	1	T/T	C/C	A/A
control-0341	2	60	0	0	0	T/T	G/C	A/A
control-0342	1	48	0	0	1	C/T	G/C	A/A

control-0343	2	53	0	0	1	C/T	G/C	A/A
control-0344	1	70	1	0	1	C/T	G/C	A/A
control-0345	2	60	0	0	1	C/T	G/C	A/A
control-0346	2	63	0	0	0	T/T	G/C	A/A
control-0347	2	61	0	0	1	C/T	G/C	A/A
control-0348	1	55	0	0	0	C/T	G/C	A/A
control-0349	1	69	0	0	1	T/T	C/C	A/A
control-0350	1	67	0	0	1	C/T	C/C	G/A
control-0351	1	62	0	0	0	C/C	G/C	A/A
control-0352	2	49	0	0	0	C/T	C/C	G/A
control-0353	1	55	0	0	1	C/T	C/C	A/A
control-0354	1	62	1	0	0	C/T	G/C	A/A
control-0355	1	48	1	0	1	T/T	G/C	A/A
control-0356	1	49	1	0	1	C/T	G/C	G/A
control-0357	1	61	0	0	1	C/T	C/C	A/A
control-0358	2	53	0	0	0	C/T	C/C	A/A
control-0359	1	52	0	0	0	T/T	G/C	A/A
control-0360	2	67	0	0	0	C/C	C/C	A/A
control-0361	1	30	0	0	0	T/T	G/G	G/G
control-0362	1	58	0	0	0	C/C	C/C	G/A
control-0363	2	61	0	0	1	C/T	C/C	A/A
control-0364	1	54	0	0	0	C/T	C/C	A/A
control-0365	2	42	0	0	0	T/T	C/C	A/A
control-0366	2	61	0	0	0	C/T	G/C	G/A
control-0367	1	57	0	0	0	T/T	G/G	A/A
control-0368	1	50	1	0	1	C/T	C/C	A/A
control-0369	2	66	0	0	0	C/T	C/C	A/A
control-0370	1	55	1	0	1	C/T	G/G	A/A
control-0371	2	52	0	0	1	T/T	G/C	A/A
control-0372	1	41	0	0	0	C/C	C/C	A/A
control-0373	2	63	0	0	1	T/T	G/C	A/A
control-0374	1	59	0	0	1	C/T	G/C	A/A
control-0375	1	74	1	1	0	T/T	G/C	A/A
control-0376	1	68	0	0	0	C/T	G/C	G/A
control-0377	1	63	1	1	1	T/T	G/C	G/A
control-0378	1	60	1	0	0	C/T	C/C	G/A
control-0379	1	59	0	0	1	T/T	G/C	G/A
control-0380	2	57	0	0	1	C/C	C/C	A/A
control-0381	1	60	1	0	1	C/T	C/C	G/A
control-0382	2	42	0	0	0	C/T	G/C	A/A
control-0383	1	54	1	0	0	C/C	G/C	A/A
control-0384	2	42	0	0	1	C/T	C/C	A/A
control-0385	1	51	0	0	0	T/T	G/C	A/A
control-0386	1	50	0	0	1	T/T	G/C	A/A
control-0387	1	58	0	0	1	T/T	C/C	G/A
control-0388	2	65	1	0	1	C/T	C/C	G/A
control-0389	1	51	0	0	0	C/T	G/C	A/A
control-0390	1	64	0	0	1	T/T	G/C	A/A

control-0391	1	27	0	0	0	C/T	C/C	G/A
control-0392	1	63	0	0	0	C/T	G/C	A/A
control-0393	1	53	0	0	0	T/T	G/C	A/A
control-0394	2	64	0	0	0	T/T	C/C	A/A
control-0395	1	55	1	0	0	T/T	C/C	A/A
control-0396	1	39	1	0	0	T/T	C/C	G/A
control-0397	1	69	0	0	1	C/C	C/C	A/A
control-0398	1	50	1	0	0	C/T	C/C	A/A
control-0399	1	76	0	0	0	T/T	G/C	A/A
control-0400	1	79	1	0	0	C/T	C/C	A/A
control-0401	1	52	0	0	0	C/C	C/C	A/A
control-0402	2	58	0	0	1	C/C	G/C	A/A
control-0403	1	43	0	0	0	C/C	G/C	A/A
control-0404	2	53	0	0	0	C/T	C/C	A/A
control-0405	1	78	0	0	0	C/T	G/C	G/A
control-0406	1	63	1	0	0	C/T	G/C	G/A
control-0407	1	57	1	0	1	C/T	G/C	G/A
control-0408	2	60	0	0	0	C/T	G/G	A/A
control-0409	1	67	0	0	1	T/T	G/C	G/A
control-0410	1	66	0	0	1	C/T	G/C	A/A
control-0411	1	62	0	0	1	T/T	G/G	G/A
control-0412	2	61	0	0	1	C/T	G/G	A/A
control-0413	1	61	0	0	1	C/T	G/C	A/A
control-0414	1	66	0	0	1	C/C	G/G	A/A
control-0415	1	52	0	0	1	C/C	C/C	G/A
control-0416	1	60	1	0	0	T/T	C/C	G/A
control-0417	1	72	1	1	0	C/C	G/C	A/A
control-0418	1	70	0	0	0	C/C	G/C	A/A
control-0419	1	68	0	0	0	T/T	C/C	A/A
control-0420	1	71	1	0	0	C/T	G/C	A/A
control-0421	2	69	0	0	1	C/T	G/C	A/A
control-0422	2	61	0	0	1	C/C	C/C	G/A
control-0423	1	69	0	0	1	C/T	G/C	A/A
control-0424	1	74	1	0	1	C/C	G/C	A/A
control-0425	2	52	0	0	1	T/T	G/G	A/A
control-0426	1	41	0	0	0	T/T	C/C	A/A
control-0427	1	52	0	0	0	T/T	G/C	A/A
control-0428	2	44	0	0	0	C/C	G/C	G/G
control-0429	2	50	0	0	0	C/T	G/C	A/A
control-0430	1	63	0	0	1	C/T	G/C	G/A
control-0431	1	59	1	0	1	T/T	G/G	A/A
control-0432	1	44	0	0	1	C/T	C/C	G/A
control-0433	2	66	0	0	0	C/C	C/C	G/A
control-0434	1	59	1	1	0	T/T	C/C	A/A
control-0435	1	61	1	0	1	C/T	G/C	G/A
control-0436	2	53	0	0	1	T/T	C/C	A/A
control-0437	1	66	1	0	1	C/T	G/C	A/A
control-0438	2	54	0	0	0	C/T	C/C	A/A

control-0439	2	51	0	0	1	C/C	G/C	G/A
control-0440	2	61	0	0	0	C/T	G/G	A/A
control-0441	1	56	0	0	1	T/T	G/C	A/A
control-0442	1	52	0	0	1	C/T	C/C	G/A
control-0443	2	49	0	0	1	C/T	C/C	A/A
control-0444	2	48	0	0	0	C/T	C/C	A/A
control-0445	2	45	0	0	1	C/C	G/C	A/A
control-0446	2	47	0	0	0	C/C	C/C	G/A
control-0447	2	65	0	0	1	T/T	C/C	A/A
control-0448	1	73	1	0	0	C/T	G/C	A/A
control-0449	2	59	0	0	0	C/C	C/C	A/A
control-0450	1	59	0	0	0	C/T	G/C	G/A
control-0451	1	67	0	0	0	C/T	C/C	A/A
control-0452	1	59	0	0	1	T/T	C/C	G/A
control-0453	1	59	0	0	1	C/T	G/C	A/A
control-0454	1	47	1	0	1	C/T	G/C	A/A
control-0455	2	68	0	0	0	C/T	G/G	A/A
control-0456	1	64	0	1	0	C/C	G/C	A/A
control-0457	2	50	0	0	1	C/T	G/C	G/A
control-0458	1	74	1	0	0	C/C	G/G	A/A
control-0459	2	50	0	0	0	T/T	G/C	A/A
control-0460	1	70	0	0	0	C/T	C/C	A/A
control-0461	2	63	0	0	0	C/T	G/C	A/A
control-0462	1	53	0	0	0	C/C	G/C	A/A
control-0463	2	59	0	0	0	C/T	G/C	G/A
control-0464	1	58	0	0	0	C/T	C/C	G/A
control-0465	1	68	0	0	0	T/T	G/C	A/A
control-0466	2	53	0	0	1	C/T	C/C	A/A
control-0467	2	65	0	0	1	C/T	C/C	G/A
control-0468	1	73	1	0	0	C/T	C/C	A/A
control-0469	2	56	0	0	1	C/T	G/C	A/A
control-0470	1	59	0	0	0	C/C	G/C	A/A
control-0471	2	51	0	0	1	T/T	C/C	A/A
control-0472	1	62	0	0	1	T/T	G/C	G/A
control-0473	1	55	0	0	0	T/T	G/C	A/A
control-0474	2	59	0	0	1	C/T	G/C	A/A
control-0475	1	62	0	0	1	C/T	C/C	A/A
control-0476	1	69	0	0	0	T/T	G/C	G/A
control-0477	1	29	0	0	1	C/T	G/G	A/A
control-0478	1	59	0	0	0	C/T	G/C	A/A
control-0479	1	52	1	0	0	C/T	G/C	A/A
control-0480	1	60	0	0	1	C/T	G/C	A/A
control-0481	2	61	0	0	0	T/T	C/C	A/A
control-0482	2	69	0	0	1	C/T	C/C	A/A
control-0483	1	59	0	0	1	C/C	C/C	A/A
control-0484	2	63	0	0	1	C/C	C/C	A/A
control-0485	2	66	0	0	0	C/T	G/C	G/A
control-0486	1	63	0	0	1	C/T	G/C	A/A

control-0487	1	74	0	0	1	C/C	C/C	A/A
control-0488	2	59	0	0	1	C/C	C/C	A/A
control-0489	1	57	0	0	1	C/T	C/C	A/A
control-0490	1	63	0	0	0	C/T	C/C	A/A
control-0491	2	68	0	0	1	C/T	G/C	A/A
control-0492	1	61	1	0	1	T/T	C/C	A/A
control-0493	1	53	0	0	1	C/T	G/C	G/A
control-0494	2	58	0	0	0	C/T	G/C	A/A
control-0495	1	62	0	0	1	C/T	G/C	A/A
control-0496	1	57	1	0	0	C/T	G/C	A/A
control-0497	1	57	1	0	1	C/C	C/C	G/A
control-0498	1	46	1	1	1	C/T	G/C	G/A
control-0499	1	60	1	1	0	C/T	G/G	A/A
control-0500	2	65	0	0	0	C/T	G/C	G/A
control-0501	1	62	0	0	0	T/T	C/C	A/A
control-0502	1	54	1	0	0	T/T	C/C	G/A
control-0503	1	53	0	0	0	T/T	C/C	A/A
control-0504	2	64	0	0	1	C/T	G/C	A/A
control-0505	1	64	1	1	1	C/C	G/C	A/A
control-0506	1	55	0	0	1	T/T	C/C	A/A
control-0507	2	60	0	0	0	T/T	G/C	A/A
control-0508	1	59	1	0	0	C/T	C/C	A/A
control-0509	1	59	1	0	1	C/T	G/C	G/A
control-0510	1	63	1	0	0	T/T	C/C	A/A
control-0511	1	62	0	1	1	T/T	C/C	A/A
control-0512	1	53	0	0	1	T/T	G/C	A/A
control-0513	1	52	0	0	0	C/C	G/C	A/A
control-0514	1	54	1	0	1	C/T	G/C	A/A
control-0515	1	55	0	0	0	C/T	C/C	A/A
control-0516	2	69	0	0	1	C/T	G/C	G/A
control-0517	1	65	0	1	0	C/T	C/C	A/A
control-0518	1	58	0	1	1	C/T	C/C	G/A
control-0519	1	51	1	0	1	C/T	C/C	A/A
control-0520	1	48	1	0	1	T/T	C/C	G/A
control-0521	2	54	0	0	0	C/C	G/C	A/A
control-0522	2	60	0	0	1	C/T	G/G	G/A
control-0523	2	64	0	0	0	C/C	C/C	A/A
control-0524	1	59	1	1	1	C/T	G/C	G/A
control-0525	2	59	0	0	0	C/T	G/C	A/A
control-0526	1	56	1	0	0	C/T	G/C	A/A
control-0527	1	60	0	0	0	T/T	C/C	A/A
control-0528	1	51	0	0	0	C/C	C/C	A/A
control-0529	1	58	1	0	0	C/T	C/C	A/A
control-0530	1	65	0	0	0	T/T	C/C	A/A
control-0531	1	63	0	0	0	C/T	C/C	A/A
control-0532	2	58	0	0	0	T/T	G/C	A/A
control-0533	2	61	0	0	0	C/T	G/C	A/A
control-0534	1	62	1	1	1	T/T	C/C	G/A

control-0535	1	54	0	0	0	C/T	G/G	G/A
control-0536	1	58	0	0	1	T/T	C/C	A/A
control-0537	2	60	0	0	1	C/T	G/C	A/A
control-0538	1	59	1	0	1	C/T	G/C	A/A
control-0539	2	59	0	0	1	C/C	G/G	A/A
control-0540	1	64	0	0	1	C/T	C/C	A/A
control-0541	1	58	1	0	0	T/T	G/C	A/A
control-0542	1	60	0	0	1	C/T	G/C	G/A
control-0543	1	60	0	1	1	T/T	C/C	A/A
control-0544	2	50	0	0	0	C/T	G/C	A/A
control-0545	1	54	1	1	1	T/T	C/C	G/A
control-0546	1	58	0	0	1	C/C	G/G	A/A
control-0547	1	57	0	0	0	C/C	G/G	G/A
control-0548	1	62	0	0	1	C/T	C/C	G/A
control-0549	1	65	0	0	1	T/T	G/C	A/A
control-0550	1	61	0	0	1	C/T	C/C	G/A
control-0551	2	61	0	0	0	C/T	C/C	A/A
control-0552	2	57	0	0	0	C/T	G/C	G/A
control-0553	1	56	1	1	1	C/T	C/C	A/A
control-0554	2	59	0	0	1	T/T	G/G	A/A
control-0555	2	61	0	0	1	T/T	G/G	A/A
control-0556	2	54	0	1	0	T/T	G/C	A/A
control-0557	1	65	0	0	1	C/T	C/C	A/A
control-0558	1	64	1	0	0	C/T	C/C	G/A
control-0559	2	66	0	0	1	C/T	G/C	A/A
control-0560	1	56	1	1	0	C/T	G/C	A/A
control-0561	1	51	1	0	1	C/T	G/C	A/A
control-0562	1	54	0	0	1	C/T	G/G	G/A
control-0563	1	59	1	1	0	C/T	G/C	G/A
control-0564	1	57	1	0	1	C/C	C/C	A/A
control-0565	1	52	1	1	0	C/C	G/C	A/A
control-0566	2	65	0	0	0	T/T	G/C	A/A
control-0567	1	59	1	0	0	C/T	G/C	A/A
control-0568	1	64	0	1	1	C/T	C/C	A/A
control-0569	1	57	1	0	1	C/T	C/C	A/A
control-0570	1	52	0	0	1	C/T	G/C	A/A
control-0571	1	51	0	0	1	C/T	G/G	A/A
control-0572	1	63	0	0	0	T/T	C/C	A/A
control-0573	1	63	1	0	1	C/T	G/C	G/A
control-0574	2	68	0	0	1	C/T	G/G	A/A
control-0575	2	55	0	0	1	T/T	C/C	A/A
control-0576	1	55	1	1	1	C/T	G/C	A/A
control-0577	1	64	1	1	0	T/T	G/G	G/A
control-0578	1	60	0	0	0	C/C	C/C	G/A
control-0579	1	50	0	0	0	C/T	G/G	A/A
control-0580	1	51	0	1	1	C/C	G/C	G/A
control-0581	1	58	1	1	0	T/T	G/C	A/A
control-0582	1	57	0	0	0	C/T	C/C	G/G

control-0583	1	61	1	1	1	C/T	C/C	G/A
control-0584	1	63	1	0	0	T/T	C/C	G/A
control-0585	1	60	1	0	0	C/C	C/C	A/A
control-0586	2	66	0	0	1	C/C	G/C	A/A
control-0587	1	65	1	0	0	C/T	G/C	G/A
control-0588	2	63	0	0	0	T/T	C/C	G/A
control-0589	2	59	0	0	1	C/T	G/C	A/A
control-0590	1	59	1	1	0	C/C	G/C	G/A
control-0591	1	61	1	1	0	C/T	G/C	A/A
control-0592	1	50	0	1	1	C/C	C/C	G/A
control-0593	2	50	0	0	1	T/T	G/C	A/A
control-0594	2	56	0	0	0	C/T	G/C	G/A
control-0595	1	55	0	0	0	T/T	G/C	G/A
control-0596	2	59	0	0	1	C/T	C/C	G/G
control-0597	1	59	0	0	0	T/T	G/C	A/A
control-0598	1	59	1	0	0	C/T	G/G	A/A
control-0599	1	56	0	0	1	T/T	G/C	A/A
control-0600	2	63	0	0	1	C/T	G/G	A/A
control-0601	1	52	1	0	1	C/T	C/C	A/A
control-0602	2	68	0	0	1	C/T	C/C	A/A
control-0603	2	53	0	0	1	C/T	G/C	A/A
control-0604	1	61	1	1	0	C/C	C/C	A/A
control-0605	2	68	0	0	0	C/T	G/G	G/A
control-0606	1	56	0	0	0	C/C	G/C	A/A
control-0607	1	64	1	1	0	C/C	G/C	A/A
control-0608	2	53	0	0	1	T/T	G/G	A/A
control-0609	1	52	0	0	1	C/T	C/C	A/A
control-0610	1	52	1	0	1	T/T	G/C	A/A
control-0611	1	66	0	0	0	C/T	C/C	A/A
control-0612	2	57	0	0	0	T/T	C/C	A/A
control-0613	1	56	1	1	0	C/T	G/G	A/A
control-0614	2	62	0	0	0	T/T	G/C	G/A
control-0615	2	61	0	0	0	C/T	C/C	A/A
control-0616	2	57	0	0	0	C/C	C/C	A/A
control-0617	2	63	0	0	1	C/T	C/C	A/A
control-0618	2	58	0	0	1	C/T	C/C	G/A
control-0619	1	50	0	0	0	C/C	G/G	A/A
control-0620	2	55	0	0	1	C/T	G/C	A/A
control-0621	1	56	0	0	1	C/T	G/C	A/A
control-0622	2	59	0	0	0	T/T	G/C	A/A
control-0623	1	65	1	1	0	T/T	G/C	G/G
control-0624	2	47	0	0	1	C/C	G/C	G/A
control-0625	1	58	1	1	0	C/T	C/C	A/A
control-0626	1	60	1	1	0	T/T	G/C	A/A
control-0627	1	69	0	0	1	C/T	C/C	A/A
control-0628	2	62	0	0	0	C/T	C/C	G/A
control-0629	2	61	0	0	1	C/T	G/G	A/A
control-0630	2	69	0	0	1	C/T	G/C	A/A

control-0631	2	53	0	0	0	C/T	G/C	A/A
control-0632	1	58	0	0	1	C/T	G/C	A/A
control-0633	2	61	0	0	0	C/C	C/C	A/A
control-0634	1	61	0	0	0	T/T	C/C	A/A
control-0635	2	53	0	0	0	C/C	C/C	A/A
control-0636	1	69	0	0	1	C/T	G/C	A/A
control-0637	2	45	0	0	1	C/T	C/C	A/A
control-0638	1	55	1	1	0	C/T	G/C	A/A
control-0639	2	52	0	0	1	T/T	G/C	G/A
control-0640	1	67	0	0	1	C/C	G/C	A/A
control-0641	1	53	1	0	1	C/C	G/C	G/A
control-0642	2	59	0	0	1	C/T	C/C	A/A
control-0643	1	49	1	1	0	C/T	C/C	A/A
control-0644	2	59	0	0	1	C/C	G/C	G/A
control-0645	1	55	1	0	1	T/T	G/C	G/G
control-0646	1	62	0	0	1	C/C	G/C	A/A
control-0647	2	63	0	0	1	T/T	G/C	A/A
control-0648	1	57	0	1	0	C/T	G/C	A/A
control-0649	1	53	0	0	0	T/T	G/C	A/A
control-0650	2	68	0	0	0	C/T	G/C	A/A
control-0651	1	45	0	0	1	T/T	G/C	A/A
control-0652	1	67	1	1	0	T/T	C/C	A/A
control-0653	1	52	0	0	1	C/T	C/C	G/A
control-0654	2	47	0	0	0	T/T	G/C	A/A
control-0655	1	53	0	0	1	C/T	C/C	G/A
control-0656	2	56	0	0	0	T/T	G/G	G/A
control-0657	2	53	0	0	0	C/C	G/C	A/A
control-0658	1	52	0	1	0	C/C	C/C	A/A
control-0659	2	67	0	0	1	C/T	C/C	G/A
control-0660	2	48	0	0	1	C/T	C/C	A/A
control-0661	1	52	0	0	1	T/T	G/G	A/A
control-0662	2	65	0	0	1	C/T	C/C	A/A
control-0663	2	48	0	0	0	C/T	C/C	A/A
control-0664	2	59	0	0	0	T/T	C/C	G/A
control-0665	1	69	1	1	0	C/T	G/C	A/A
control-0666	1	50	0	0	0	C/T	G/C	A/A
control-0667	2	53	0	0	1	C/C	C/C	A/A
control-0668	1	54	0	0	1	C/T	C/C	A/A
control-0669	1	56	0	0	1	C/T	C/C	A/A
control-0670	2	58	0	0	1	C/C	C/C	A/A
control-0671	1	60	0	0	0	C/T	G/C	A/A
control-0672	1	82	1	0	0	C/T	C/C	A/A
control-0673	1	60	1	0	0	C/T	G/G	A/A
control-0674	2	57	0	0	1	C/T	C/C	A/A
control-0675	1	55	0	0	0	T/T	C/C	A/A
control-0676	2	68	0	0	0	C/T	C/C	G/A
control-0677	2	61	0	0	1	C/T	G/G	A/A
control-0678	1	45	1	1	0	C/T	G/C	G/A

control-0679	2	64	0	0	0	C/C	G/C	A/A
control-0680	2	66	0	0	0	T/T	G/C	G/A
control-0681	2	67	0	0	0	C/T	G/C	A/A
control-0682	2	51	0	0	1	C/T	G/G	A/A
control-0683	2	67	0	0	1	C/T	G/G	A/A
control-0684	1	51	1	0	0	C/T	C/C	A/A
control-0685	1	65	0	0	0	T/T	G/C	A/A
control-0686	2	61	0	0	1	T/T	G/G	G/A
control-0687	2	65	0	0	0	C/T	G/C	A/A
control-0688	1	65	0	0	1	C/T	C/C	G/A
control-0689	1	60	1	0	1	T/T	C/C	A/A
control-0690	1	58	1	1	1	C/T	G/C	G/A
control-0691	2	67	0	0	0	T/T	G/C	A/A
control-0692	2	66	0	0	1	C/T	C/C	A/A
control-0693	1	68	0	0	1	C/T	G/C	A/A
control-0694	1	43	0	0	1	C/C	G/G	A/A
control-0695	2	47	0	0	1	C/T	G/C	A/A
control-0696	2	52	0	0	1	C/T	G/C	G/A
control-0697	1	52	0	0	1	C/C	C/C	A/A
control-0698	2	61	0	0	0	C/C	G/C	A/A
control-0699	2	66	0	0	0	C/C	G/C	A/A
control-0700	2	59	0	0	0	C/T	G/C	A/A
control-0701	1	63	1	0	1	C/C	G/G	G/A
control-0702	1	59	1	1	0	C/T	G/C	A/A
control-0703	2	56	0	0	1	C/C	G/C	A/A
control-0704	2	69	0	0	1	C/T	C/C	A/A
control-0705	1	58	1	1	1	C/C	G/C	A/A
control-0706	1	49	1	1	1	T/T	G/C	G/A
control-0707	2	60	0	0	1	C/T	C/C	A/A
control-0708	1	51	1	1	0	C/T	G/C	A/A
control-0709	2	51	0	0	1	C/C	G/G	A/A
control-0710	1	46	1	0	1	C/T	C/C	A/A
control-0711	1	64	1	0	0	C/T	G/C	A/A
control-0712	1	56	1	1	1	T/T	G/G	A/A
control-0713	2	46	0	0	0	T/T	C/C	A/A
control-0714	2	56	0	0	0	T/T	G/C	A/A
control-0715	1	53	0	0	1	C/T	G/C	A/A
control-0716	2	66	0	0	1	T/T	C/C	A/A
control-0717	2	54	0	0	1	C/T	C/C	A/A
control-0718	1	53	0	0	0	T/T	G/C	A/A
control-0719	1	65	1	0	1	C/C	G/C	A/A
control-0720	2	60	0	0	0	C/T	G/C	G/A
control-0721	2	48	0	0	0	C/T	C/C	A/A
control-0722	1	52	0	0	0	T/T	G/C	A/A
control-0723	2	62	0	0	0	T/T	G/C	A/A
control-0724	2	61	0	0	0	C/T	C/C	G/A
control-0725	1	56	0	0	1	T/T	C/C	A/A
control-0726	1	57	0	0	1	T/T	G/C	G/A

control-0727	1	61	1	1	0	T/T	G/G	A/A
control-0728	2	65	0	0	0	C/T	C/C	A/A
control-0729	2	68	0	0	0	C/T	G/G	A/A
control-0730	1	60	1	0	0	C/T	C/C	A/A
control-0731	1	65	0	0	1	T/T	G/C	G/A
control-0732	2	56	0	0	0	C/C	G/C	A/A
control-0733	1	50	0	1	0	C/C	C/C	A/A
control-0734	1	49	0	0	0	C/C	G/C	A/A
control-0735	2	47	0	0	1	C/T	G/C	A/A
control-0736	2	71	0	0	1	T/T	C/C	G/A
control-0737	2	64	0	0	0	C/T	G/C	A/A
control-0738	2	61	0	0	1	C/C	G/G	G/A
control-0739	2	60	0	0	0	T/T	G/G	A/A
control-0740	2	66	0	0	1	C/T	C/C	A/A
control-0741	2	77	0	0	0	T/T	C/C	G/A
control-0742	2	75	0	0	1	C/T	C/C	A/A
control-0743	2	43	0	0	1	C/C	G/G	G/A
control-0744	2	66	0	0	1	C/T	G/G	G/A
control-0745	1	58	0	0	0	T/T	C/C	A/A
control-0746	1	58	0	1	1	T/T	G/C	A/A
control-0747	1	62	0	0	1	C/T	G/G	A/A
control-0748	2	66	0	0	1	C/C	C/C	G/A
control-0749	2	61	0	0	0	C/C	G/C	A/A
control-0750	1	66	1	0	0	C/T	G/C	A/A
control-0751	2	61	0	0	0	C/T	C/C	G/A
control-0752	2	70	0	0	1	C/T	C/C	A/A
control-0753	1	70	0	0	1	C/C	C/C	A/A
control-0754	1	69	1	1	1	C/T	C/C	A/A
control-0755	1	60	0	0	0	C/T	G/G	A/A
control-0756	2	72	0	0	1	C/T	C/C	A/A
control-0757	1	56	0	0	1	T/T	G/C	G/A
control-0758	1	66	0	0	0	C/T	C/C	A/A
control-0759	2	51	0	0	0	C/C	C/C	A/A
control-0760	1	47	1	1	1	C/T	G/C	A/A
control-0761	2	72	0	0	0	C/C	G/C	A/A
control-0762	2	74	0	0	0	C/C	G/C	G/A
control-0763	2	59	0	0	0	C/C	G/G	A/A
control-0764	1	69	0	0	1	C/T	G/C	A/A
control-0765	1	64	0	0	0	T/T	C/C	A/A
control-0766	2	66	0	0	0	T/T	G/C	A/A
control-0767	2	72	0	0	1	C/T	C/C	A/A
control-0768	2	67	0	0	1	T/T	G/C	A/A
control-0769	2	79	0	0	1	C/T	G/C	G/A
control-0770	2	59	0	0	0	C/T	G/C	G/A
control-0771	2	53	0	0	0	C/C	C/C	A/A
control-0772	1	66	0	0	1	C/T	C/C	G/A
control-0773	1	50	0	0	1	T/T	C/C	A/A
control-0774	1	67	0	0	1	C/T	C/C	G/A

control-0775	1	83	0	0	1	C/T	G/G	A/A
control-0776	2	50	0	0	0	C/T	G/C	G/A
control-0777	2	69	0	0	0	C/C	G/C	A/A
control-0778	1	52	0	0	0	T/T	G/G	G/A
control-0779	2	69	0	0	0	C/T	C/C	A/A
control-0780	2	83	0	0	1	C/T	G/G	A/A
control-0781	2	68	0	0	0	C/C	G/C	A/A
control-0782	2	61	0	0	0	C/T	G/C	G/A
control-0783	2	53	0	0	1	C/T	G/C	A/A
control-0784	2	74	0	0	1	C/T	G/G	A/A
control-0785	2	67	0	0	1	C/C	G/C	A/A
control-0786	2	73	0	0	0	T/T	C/C	A/A
control-0787	2	64	0	0	0	T/T	G/G	A/A
control-0788	2	61	0	0	1	C/C	G/C	A/A
control-0789	2	68	0	0	1	C/T	C/C	A/A
control-0790	2	62	0	0	1	T/T	G/G	G/A
control-0791	2	75	0	0	1	C/T	C/C	A/A
control-0792	2	65	0	0	1	C/T	G/C	G/A
control-0793	2	69	0	0	1	C/T	G/C	A/A
control-0794	2	48	0	0	0	T/T	C/C	A/A
control-0795	2	63	0	0	1	C/T	C/C	A/A
control-0796	2	41	0	0	0	T/T	C/C	A/A
control-0797	2	49	0	0	0	C/T	C/C	A/A
control-0798	2	66	0	0	0	C/T	G/G	A/A
control-0799	2	45	0	0	0	C/T	C/C	G/A
control-0800	2	45	0	0	1	C/T	G/C	A/A
control-0801	2	46	0	0	1	C/T	G/C	A/A
control-0802	2	67	0	0	0	C/T	C/C	G/A
control-0803	2	65	0	0	0	C/T	G/C	A/A
control-0804	2	74	0	0	0	C/C	C/C	A/A
control-0805	2	62	0	0	0	C/T	G/C	A/A
control-0806	2	55	0	0	0	C/T	G/C	A/A
control-0807	2	59	0	0	1	C/T	C/C	A/A
control-0808	2	59	1	1	1	C/T	C/C	A/A
control-0809	2	57	0	0	1	C/T	C/C	G/A
control-0810	2	64	0	0	0	C/C	C/C	G/A
control-0811	2	58	0	0	1	T/T	C/C	A/A
control-0812	2	64	0	0	1	C/C	C/C	A/A
control-0813	2	62	0	0	0	C/C	G/C	G/A
control-0814	2	63	0	0	0	T/T	C/C	A/A
control-0815	2	56	0	0	1	T/T	G/C	A/A
control-0816	1	57	0	0	0	T/T	G/C	A/A
control-0817	1	54	0	0	1	T/T	C/C	A/A
control-0818	1	52	0	0	0	C/T	G/G	A/A
control-0819	2	52	0	0	0	T/T	G/G	A/A
control-0820	1	53	0	0	0	C/C	G/G	A/A
control-0821	1	59	0	0	0	T/T	G/C	A/A
control-0822	1	61	0	0	0	T/T	G/C	G/A

control-0823	2	**66**	0	**0**	0	C/C	C/C	A/A
control-0824	1	63	0	0	0	C/C	G/C	A/A
control-0825	1	57	0	0	1	C/T	G/C	G/A
control-0826	1	67	0	0	0	C/T	C/C	G/A
control-0827	1	65	0	0	1	C/T	G/C	G/A
control-0828	1	56	0	0	1	C/T	G/C	G/A
control-0829	1	67	0	0	0	C/T	G/C	A/A
control-0830	1	71	1	1	0	C/T	G/C	A/A
control-0831	1	65	0	0	0	C/C	C/C	A/A
control-0832	1	55	0	0	0	C/T	G/C	A/A
control-0833	1	68	0	0	1	C/T	C/C	A/A
control-0834	2	53	0	0	0	C/C	G/C	A/A
control-0835	2	65	0	0	0	C/T	G/C	A/A
control-0836	2	72	0	0	0	C/T	G/C	G/A
control-0837	1	65	1	0	1	C/C	G/C	A/A
control-0838	1	65	1	0	0	C/T	C/C	A/A
control-0839	2	68	0	0	1	C/T	C/C	A/A
control-0840	1	62	0	0	1	C/T	G/G	A/A
control-0841	1	52	0	0	1	C/T	C/C	G/A
control-0842	1	70	1	1	0	T/T	G/G	A/A
control-0843	2	49	0	0	1	C/T	C/C	A/A
control-0844	2	56	0	0	0	T/T	G/C	A/A
control-0845	2	64	0	0	1	C/T	C/C	A/A
control-0846	2	68	0	0	0	C/T	G/G	A/A
control-0847	2	53	0	0	0	C/T	C/C	A/A
control-0848	2	54	0	0	0	C/T	G/C	G/A
control-0849	2	68	0	0	0	C/C	G/C	G/A
control-0850	2	49	0	0	1	T/T	C/C	A/A
control-0851	2	61	0	0	0	C/T	G/C	G/A
control-0852	2	59	0	0	0	C/T	G/C	A/A
control-0853	2	57	0	0	0	C/T	C/C	A/A
control-0854	2	52	0	0	1	C/T	G/C	A/A
control-0855	2	58	0	0	0	T/T	G/C	G/A
control-0856	2	64	0	0	0	C/C	C/C	A/A
control-0857	2	59	0	0	1	C/T	G/G	A/A
control-0858	2	58	0	0	0	T/T	C/C	A/A
control-0859	2	62	0	0	0	T/T	G/G	G/A
control-0860	2	63	0	0	1	C/T	G/C	G/A
control-0861	2	73	0	0	0	C/T	G/C	G/A
control-0862	2	59	0	0	0	C/C	G/C	G/A
control-0863	2	77	0	0	1	C/C	C/C	A/A
control-0864	2	59	0	0	0	C/T	C/C	G/G
control-0865	2	59	0	0	0	T/T	G/C	A/A
control-0866	2	73	0	0	1	C/C	G/G	A/A
control-0867	2	60	0	0	1	C/C	G/C	A/A
control-0868	2	73	0	0	0	C/C	G/C	A/A
control-0869	2	59	0	0	1	T/T	G/C	G/A
control-0870	2	71	0	0	1	C/T	G/G	A/A

control-0871	2	59	0	0	1	C/C	C/C	A/A
control-0872	2	62	0	0	0	C/T	C/C	G/A
control-0873	2	76	0	0	1	C/T	G/C	A/A
control-0874	2	56	0	0	0	T/T	C/C	G/A
control-0875	2	55	0	0	1	C/T	C/C	A/A
control-0876	2	62	0	0	0	C/T	G/C	G/G
control-0877	2	62	0	0	1	C/T	C/C	A/A
control-0878	1	57	0	0	0	C/T	G/C	A/A
control-0879	1	52	0	0	0	?	?	?
control-0880	1	54	0	0	1	T/T	C/C	G/G
control-0881	1	56	0	0	1	T/T	C/C	A/A
control-0882	1	52	0	0	0	C/T	G/C	A/A
control-0883	1	50	0	0	0	C/T	C/C	A/A
control-0884	1	62	0	0	0	C/T	G/C	A/A
control-0885	1	40	0	0	1	C/T	C/C	A/A
control-0886	1	49	0	0	1	T/T	G/C	A/A
control-0887	1	53	0	0	1	C/C	G/C	G/G
control-0888	1	45	0	0	0	C/T	G/G	G/G
control-0889	1	48	0	0	0	T/T	G/G	A/A
control-0890	1	43	0	0	0	C/C	C/C	G/G
control-0891	1	61	0	0	1	C/C	G/G	A/A
control-0892	1	51	0	0	1	C/T	G/C	G/G
control-0893	1	69	0	0	1	C/T	G/C	A/A
control-0894	1	55	0	0	1	C/T	G/G	A/A
control-0895	2	50	0	0	1	T/T	G/C	G/A
control-0896	2	56	0	0	1	C/T	G/G	A/A
control-0897	1	53	1	1	0	C/T	C/C	A/A
control-0898	2	55	0	0	0	T/T	G/C	A/A
control-0899	1	62	0	0	1	C/T	G/C	A/A
control-0900	1	49	0	0	1	T/T	G/G	A/A
control-0901	1	63	0	0	0	T/T	C/C	A/A
control-0902	2	61	0	0	1	T/T	G/C	A/A
control-0903	1	51	0	0	1	T/T	G/C	A/A
control-0904	1	47	0	0	0	C/T	G/G	A/A
control-0905	1	49	0	0	0	T/T	G/C	G/A
control-0906	2	62	0	0	1	C/T	G/G	A/A
control-0907	2	68	0	0	1	C/T	C/C	A/A
control-0908	1	40	0	0	0	C/T	C/C	A/A
control-0909	1	59	0	0	0	T/T	G/C	G/A
control-0910	1	49	0	0	0	C/C	C/C	G/A
control-0911	1	63	0	0	0	C/C	C/C	G/A
control-0912	1	49	0	0	1	C/T	C/C	G/A
control-0913	2	67	0	0	1	C/T	C/C	G/A
control-0914	1	66	0	0	0	C/T	G/C	G/A
control-0915	1	42	1	1	0	T/T	G/G	A/A
control-0916	1	41	0	0	0	T/T	G/G	A/A
control-0917	2	56	0	0	1	T/T	G/G	G/A
control-0918	1	60	1	1	0	C/T	G/C	G/A

control-0919	1	65	1	0	0	C/C	C/C	A/A
control-0920	1	55	0	0	0	T/T	C/C	A/A
control-0921	1	63	1	1	0	T/T	C/C	A/A
control-0922	2	40	0	0	0	C/T	C/C	A/A
control-0923	1	50	0	1	0	C/C	C/C	A/A
control-0924	1	50	1	0	0	C/T	G/C	A/A
control-0925	2	53	0	0	1	T/T	G/C	G/A
control-0926	2	55	0	0	0	C/T	G/C	A/A
control-0927	2	57	0	0	1	T/T	C/C	A/A
control-0928	2	56	0	0	0	C/T	C/C	A/A
control-0929	2	60	0	0	1	C/T	C/C	G/A
control-0930	2	51	0	0	1	C/T	G/G	A/A
control-0931	2	61	0	0	1	C/C	C/C	A/A
control-0932	2	52	0	0	0	C/T	G/C	G/A
control-0933	2	56	0	0	0	T/T	G/C	A/A
control-0934	2	60	0	0	0	T/T	G/C	G/G
control-0935	2	58	0	0	0	C/T	C/C	A/A
control-0936	2	59	0	0	0	C/T	C/C	A/A
control-0937	2	60	0	0	1	C/T	G/C	A/A
control-0938	2	53	0	0	1	T/T	C/C	A/A
control-0939	2	56	0	0	0	T/T	G/C	A/A
control-0940	2	61	0	0	1	C/C	G/C	A/A
control-0941	2	59	0	0	0	C/T	G/C	G/A
control-0942	2	57	0	0	0	T/T	C/C	A/A
control-0943	2	60	0	0	0	C/T	C/C	A/A
control-0944	2	32	0	0	0	T/T	C/C	G/A
control-0945	2	50	0	0	1	C/T	C/C	A/A
control-0946	2	56	0	0	0	C/T	G/G	G/A
control-0947	2	60	0	0	1	C/C	G/C	A/A
control-0948	2	58	0	0	1	T/T	C/C	A/A
control-0949	2	50	0	0	1	C/C	C/C	A/A
control-0950	2	52	0	0	1	T/T	G/C	G/A
control-0951	1	69	0	0	0	C/T	C/C	A/A
control-0952	2	79	0	0	1	C/T	G/C	G/A
control-0953	1	63	0	0	1	C/T	G/G	A/A
control-0954	1	61	0	0	0	C/C	C/C	G/A
control-0955	1	55	0	0	1	C/T	G/C	A/A
control-0956	1	77	0	0	0	C/C	C/C	A/A
control-0957	1	63	0	0	0	T/T	G/G	G/G
control-0958	2	61	0	0	1	C/T	G/C	A/A
control-0959	2	69	0	0	0	C/T	C/C	A/A
control-0960	2	47	0	0	0	C/T	C/C	A/A
control-0961	1	46	0	0	0	T/T	C/C	G/A
control-0962	1	65	1	1	0	C/T	G/C	G/A
control-0963	1	50	0	0	1	C/C	G/C	G/A
control-0964	2	76	0	0	1	T/T	G/C	A/A
control-0965	2	53	0	0	1	C/T	G/C	A/A
control-0966	1	65	0	0	1	C/T	G/C	A/A

control-0967	1	38	0	0	1	C/C	C/C	A/A
control-0968	2	46	0	0	0	C/T	G/G	A/A
control-0969	1	32	1	1	0	C/C	C/C	G/A
control-0970	2	69	0	0	0	C/T	C/C	G/A
control-0971	1	74	0	0	0	C/T	G/G	A/A
control-0972	1	58	1	0	1	C/T	C/C	A/A
control-0973	2	45	0	0	0	C/T	G/C	A/A
control-0974	1	61	1	0	1	T/T	G/C	A/A
control-0975	1	34	0	0	0	C/C	C/C	A/A
control-0976	1	68	0	0	0	C/T	G/C	A/A
control-0977	2	47	0	0	1	C/C	G/C	G/A
control-0978	2	30	0	0	0	C/T	C/C	A/A
control-0979	1	57	0	1	0	C/T	G/C	A/A
control-0980	2	46	0	0	1	T/T	C/C	A/A
control-0981	2	58	0	0	0	C/T	C/C	A/A
control-0982	1	44	1	1	1	T/T	G/G	G/A
control-0983	1	58	0	1	1	?	?	?
control-0984	1	61	1	0	0	C/T	C/C	G/A
control-0985	1	57	0	0	0	T/T	G/C	G/A
control-0986	2	63	0	0	1	C/T	G/C	G/A
control-0987	2	49	1	1	0	T/T	G/C	A/A
control-0988	2	64	0	0	0	C/T	C/C	A/A
control-0989	2	36	0	0	0	C/T	C/C	A/A
control-0990	1	59	0	0	0	C/T	G/G	A/A
control-0991	2	44	0	0	0	C/T	G/C	G/A
control-0992	2	62	0	0	0	C/C	C/C	A/A
control-0993	2	73	0	0	1	C/T	G/C	G/A
control-0994	1	60	1	0	0	T/T	C/C	A/A
control-0995	2	62	0	0	0	C/T	G/C	G/A
control-0996	1	51	1	0	1	C/T	C/C	A/A
control-0997	1	73	0	0	1	C/T	C/C	G/A
control-0998	1	75	0	0	0	C/C	C/C	A/A
control-0999	2	47	0	0	1	C/C	G/C	G/A
control-1000	1	44	0	0	0	C/C	C/C	A/A
control-1001	1	79	0	0	0	T/T	C/C	G/A
control-1002	2	65	0	0	1	C/T	C/C	A/A
control-1003	1	68	0	0	0	C/T	C/C	A/A
control-1004	2	43	0	0	0	C/C	G/C	A/A
control-1005	1	79	0	0	1	C/T	G/C	A/A
control-1006	2	51	0	0	0	T/T	G/C	A/A
control-1007	1	39	0	0	1	C/T	C/C	A/A
control-1008	2	68	0	0	0	T/T	G/C	A/A
control-1009	2	75	0	0	0	?	?	?
control-1010	2	63	0	0	0	T/T	G/C	G/A
control-1011	2	44	0	0	1	C/T	G/C	A/A
control-1012	1	60	0	0	0	T/T	C/C	A/A
control-1013	1	86	0	0	0	C/T	G/C	A/A
control-1014	1	47	0	0	0	C/C	C/C	A/A

control-1015	2	41	0	1	1	T/T	G/C	A/A
control-1016	2	79	0	0	0	C/T	G/G	A/A
control-1017	1	67	1	1	0	C/T	C/C	A/A
control-1018	1	46	0	0	0	C/C	G/C	A/A
control-1019	2	56	0	0	1	C/T	G/C	G/A
control-1020	2	74	0	0	0	C/C	C/C	G/G
control-1021	1	76	0	0	0	T/T	C/C	G/A
control-1022	1	44	1	0	0	C/T	G/C	G/A
control-1023	1	43	0	0	1	T/T	C/C	G/A
control-1024	1	74	1	1	1	C/C	G/C	G/A
control-1025	2	68	0	0	0	C/C	G/C	A/A
control-1026	1	82	0	0	0	T/T	C/C	G/A
control-1027	2	78	0	0	1	C/C	C/C	A/A
control-1028	1	64	1	0	0	C/C	C/C	A/A
control-1029	1	36	1	1	0	C/C	G/G	A/A
control-1030	1	35	0	0	1	C/T	C/C	A/A
control-1031	1	63	0	0	0	T/T	G/C	G/A
control-1032	2	74	0	0	0	C/T	G/C	A/A
control-1033	2	63	0	0	0	C/T	G/C	A/A
control-1034	2	70	0	0	1	C/C	C/C	A/A
control-1035	1	63	0	0	0	C/T	G/C	A/A
control-1036	1	65	0	0	0	C/T	G/C	A/A
control-1037	1	80	0	0	1	T/T	G/C	G/A
control-1038	1	74	1	1	0	C/T	C/C	G/A
control-1039	1	55	0	0	1	C/C	G/C	A/A
control-1040	1	24	0	0	0	C/T	G/G	A/A
control-1041	2	71	0	0	0	C/T	G/C	A/A
control-1042	1	58	0	0	1	C/T	G/C	G/A
control-1043	1	53	1	1	0	C/T	G/C	G/G
control-1044	2	60	0	0	0	C/T	G/C	A/A
control-1045	1	78	0	0	1	C/C	C/C	A/A
control-1046	1	69	1	1	0	T/T	G/C	A/A
control-1047	1	53	0	0	1	T/T	G/G	A/A
control-1048	1	49	0	0	0	T/T	C/C	A/A
control-1049	2	78	0	0	1	C/T	G/G	A/A
control-1050	1	38	0	0	0	C/T	C/C	G/A
control-1051	1	47	1	1	0	C/T	G/C	A/A
control-1052	1	77	0	0	0	C/T	G/G	A/A
control-1053	2	62	0	0	0	C/T	C/C	A/A
control-1054	2	67	0	0	0	T/T	G/G	A/A
control-1055	1	54	1	0	1	C/C	C/C	A/A
control-1056	1	64	0	0	1	C/C	G/G	G/G

?: not available

Table S3 Detailed information and genotypes for non-SCC cases and controls

SUBJECTS	SEX(1: male, 2: female)	AGE (Years)	SMOKING (1: Yes, 2: No)	DRINKING (1: Yes, 2: No)	BMI (1: ≥ 24kg/m^2, 0: < 24kg/m^2)	PATHOLOGY	rs11614913	rs2910164	rs3746444
Non-SCC-0001	2	47	0	0	0	Non-SCC	T/T	G/C	G/G
Non-SCC-0002	1	60	0	0	0	Non-SCC	T/T	C/C	A/A
Non-SCC-0003	1	61	0	0	0	Non-SCC	C/T	C/C	A/A
Non-SCC-0004	2	63	0	0	0	Non-SCC	C/T	G/G	A/A
Non-SCC-0005	2	52	0	0	0	Non-SCC	T/T	G/C	G/A
Non-SCC-0006	2	57	0	0	1	Non-SCC	T/T	G/C	A/A
Non-SCC-0007	1	71	1	0	0	Non-SCC	C/C	G/G	A/A
Non-SCC-0008	2	45	0	0	0	Non-SCC	C/C	G/C	A/A
Non-SCC-0009	1	75	1	0	1	Non-SCC	C/T	C/C	A/A
Non-SCC-0010	2	53	0	0	1	Non-SCC	C/T	G/C	A/A
Non-SCC-0011	2	46	0	0	0	Non-SCC	T/T	C/C	A/A
Non-SCC-0012	1	48	1	0	0	Non-SCC	C/C	C/C	A/A
Non-SCC-0013	2	56	0	0	1	Non-SCC	T/T	C/C	A/A
Non-SCC-0014	2	43	0	0	0	Non-SCC	T/T	C/C	G/A
Non-SCC-0015	2	53	0	0	0	Non-SCC	T/T	C/C	A/A
Non-SCC-0016	2	62	0	0	1	Non-SCC	C/T	G/C	G/A
Non-SCC-0017	1	67	1	0	0	Non-SCC	C/T	C/C	A/A
Non-SCC-0018	1	53	0	0	1	Non-SCC	C/T	G/C	A/A
Non-SCC-0019	2	47	0	0	0	Non-SCC	C/T	G/C	A/A
Non-SCC-0020	2	49	0	0	1	Non-SCC	C/T	C/C	A/A
Non-SCC-0021	2	64	0	0	1	Non-SCC	C/C	G/C	G/A
Non-SCC-0022	2	57	0	0	0	Non-SCC	T/T	C/C	A/A
Non-SCC-0023	2	62	0	0	0	Non-SCC	C/T	C/C	A/A
Non-SCC-0024	2	53	0	0	0	Non-SCC	T/T	G/C	A/A
Non-SCC-0025	2	64	0	0	0	Non-SCC	T/T	G/G	A/A
Non-SCC-0026	2	59	0	0	0	Non-SCC	C/T	C/C	A/A
Non-SCC-0027	2	52	0	0	1	Non-SCC	C/T	C/C	A/A
Non-SCC-0028	2	55	0	0	1	Non-SCC	T/T	C/C	A/A
Non-SCC-0029	2	63	0	0	0	Non-SCC	C/C	C/C	A/A
Non-SCC-0030	1	42	1	0	0	Non-SCC	C/T	G/C	A/A
Non-SCC-0031	1	56	1	1	1	Non-SCC	T/T	G/C	A/A
Non-SCC-0032	1	61	1	0	0	Non-SCC	T/T	C/C	G/A
Non-SCC-0033	2	39	0	0	0	Non-SCC	C/C	G/C	A/A
Non-SCC-0034	1	65	1	0	1	Non-SCC	C/C	G/G	G/A
Non-SCC-0035	1	48	1	1	0	Non-SCC	C/T	G/C	A/A
Non-SCC-0036	2	54	0	0	0	Non-SCC	C/T	C/C	G/A
Non-SCC-0037	1	59	1	0	1	Non-SCC	T/T	C/C	G/A
Non-SCC-0038	1	52	1	0	0	Non-SCC	T/T	C/C	A/A
Non-SCC-0039	2	61	0	0	0	Non-SCC	C/T	C/C	A/A
Non-SCC-0040	2	59	0	0	1	Non-SCC	C/C	C/C	A/A
Non-SCC-0041	2	64	0	0	0	Non-SCC	C/T	C/C	A/A
Non-SCC-0042	1	51	1	0	0	Non-SCC	C/C	C/C	G/A
Non-SCC-0043	1	73	0	0	1	Non-SCC	C/T	G/G	G/A
Non-SCC-0044	1	76	0	0	0	Non-SCC	C/T	G/C	A/A
Non-SCC-0045	2	70	0	0	0	Non-SCC	C/T	C/C	G/A
Non-SCC-0046	1	67	0	0	0	Non-SCC	T/T	G/C	A/A
Non-SCC-0047	2	65	0	0	0	Non-SCC	C/T	G/G	G/A
Non-SCC-0048	1	44	1	1	0	Non-SCC	C/T	C/C	A/A
Non-SCC-0049	2	72	0	0	1	Non-SCC	T/T	C/C	A/A
Non-SCC-0050	2	48	0	0	1	Non-SCC	T/T	G/C	A/A
Non-SCC-0051	2	65	0	0	0	Non-SCC	C/C	G/C	A/A
Non-SCC-0052	2	53	0	0	0	Non-SCC	C/T	C/C	G/A
Non-SCC-0053	1	68	1	0	0	Non-SCC	C/T	C/C	G/A
Non-SCC-0054	1	53	1	0	0	Non-SCC	T/T	G/C	G/A
Non-SCC-0055	1	53	1	0	0	Non-SCC	C/C	G/G	G/A
Non-SCC-0056	1	59	1	0	0	Non-SCC	C/T	G/G	G/A
Non-SCC-0057	1	59	1	0	1	Non-SCC	C/T	C/C	G/A
Non-SCC-0058	2	59	0	0	1	Non-SCC	C/T	G/G	A/A
Non-SCC-0059	1	78	1	0	1	Non-SCC	C/T	G/C	A/A
Non-SCC-0060	1	61	0	0	0	Non-SCC	C/T	G/G	A/A
Non-SCC-0061	1	53	0	0	0	Non-SCC	C/C	G/C	G/A
Non-SCC-0062	2	74	0	0	1	Non-SCC	T/T	G/C	A/A
Non-SCC-0063	2	73	0	0	0	Non-SCC	C/T	C/C	G/A
Non-SCC-0064	2	61	0	0	0	Non-SCC	T/T	G/C	A/A
Non-SCC-0065	1	60	1	0	0	Non-SCC	C/T	C/C	A/A
Non-SCC-0066	1	70	1	1	1	Non-SCC	T/T	C/C	A/A
Non-SCC-0067	1	80	0	0	1	Non-SCC	C/T	G/C	G/A
Non-SCC-0068	1	62	1	1	0	Non-SCC	C/C	C/C	A/A
Non-SCC-0069	2	64	0	0	1	Non-SCC	C/T	C/C	A/A
Non-SCC-0070	2	45	0	0	1	Non-SCC	C/T	G/C	A/A

Non-SCC-0071	2	67	0	0	0	Non-SCC	T/T	C/C	G/A
Non-SCC-0072	2	53	0	0	0	Non-SCC	T/T	G/C	G/A
Non-SCC-0073	2	57	0	0	0	Non-SCC	C/T	C/C	A/A
Non-SCC-0074	2	62	0	0	0	Non-SCC	C/C	G/C	A/A
Non-SCC-0075	2	66	0	0	1	Non-SCC	C/C	C/C	A/A
Non-SCC-0076	1	49	0	0	1	Non-SCC	T/T	C/C	A/A
Non-SCC-0077	1	70	1	0	0	Non-SCC	C/T	C/C	A/A
Non-SCC-0078	1	71	1	0	0	Non-SCC	T/T	G/C	A/A
Non-SCC-0079	1	47	1	0	0	Non-SCC	T/T	G/C	A/A
Non-SCC-0080	1	64	1	0	0	Non-SCC	C/C	G/C	G/A
Non-SCC-0081	2	55	0	0	1	Non-SCC	T/T	C/C	G/A
Non-SCC-0082	2	63	0	0	0	Non-SCC	T/T	G/G	A/A
Non-SCC-0083	2	64	0	0	0	Non-SCC	C/T	G/C	A/A
Non-SCC-0084	1	70	1	0	1	Non-SCC	T/T	G/C	G/A
Non-SCC-0085	1	64	0	0	0	Non-SCC	C/T	C/C	G/A
Non-SCC-0086	1	62	1	0	1	Non-SCC	T/T	G/C	A/A
Non-SCC-0087	2	72	0	0	0	Non-SCC	C/T	G/C	G/G
Non-SCC-0088	1	59	0	0	1	Non-SCC	T/T	G/C	A/A
Non-SCC-0089	1	73	1	0	0	Non-SCC	C/T	C/C	A/A
Non-SCC-0090	2	68	0	0	1	Non-SCC	C/T	G/C	A/A
Non-SCC-0091	1	44	1	0	0	Non-SCC	C/T	C/C	A/A
Non-SCC-0092	2	48	0	0	1	Non-SCC	C/T	G/C	A/A
Non-SCC-0093	1	87	0	0	1	Non-SCC	C/T	G/G	A/A
Non-SCC-0094	2	60	0	0	1	Non-SCC	T/T	G/G	G/A
Non-SCC-0095	2	58	0	0	0	Non-SCC	C/T	G/C	A/A
Non-SCC-0096	2	61	0	0	0	Non-SCC	T/T	C/C	A/A
Non-SCC-0097	2	57	0	0	0	Non-SCC	T/T	G/C	A/A
Non-SCC-0098	1	50	0	0	1	Non-SCC	C/T	G/C	A/A
Non-SCC-0099	2	69	0	0	0	Non-SCC	T/T	C/C	A/A
Non-SCC-0100	1	58	1	0	0	Non-SCC	C/T	C/C	A/A
Non-SCC-0101	2	52	0	0	0	Non-SCC	C/T	G/C	A/A
Non-SCC-0102	2	56	0	0	1	Non-SCC	C/C	G/C	A/A
Non-SCC-0103	1	34	1	0	1	Non-SCC	C/C	C/C	A/A
Non-SCC-0104	2	70	0	0	0	Non-SCC	C/T	G/C	A/A
Non-SCC-0105	2	36	0	0	0	Non-SCC	C/T	G/G	G/A
Non-SCC-0106	2	65	0	0	1	Non-SCC	C/T	G/C	A/A
Non-SCC-0107	2	40	0	0	1	Non-SCC	T/T	C/C	G/A
Non-SCC-0108	2	42	0	0	0	Non-SCC	C/T	G/C	A/A
Non-SCC-0109	1	62	0	0	1	Non-SCC	C/T	G/C	A/A
Non-SCC-0110	2	58	0	0	0	Non-SCC	C/C	G/G	A/A
Non-SCC-0111	1	53	1	0	0	Non-SCC	T/T	G/G	A/A
Non-SCC-0112	1	66	1	0	1	Non-SCC	T/T	G/C	A/A
Non-SCC-0113	1	58	1	1	0	Non-SCC	T/T	G/C	G/A
Non-SCC-0114	2	57	0	0	1	Non-SCC	C/T	G/C	A/A
Non-SCC-0115	2	73	0	0	1	Non-SCC	T/T	C/C	A/A
Non-SCC-0116	2	61	0	0	0	Non-SCC	T/T	C/C	A/A
Non-SCC-0117	2	63	0	0	1	Non-SCC	C/C	G/C	A/A
Non-SCC-0118	2	60	0	0	1	Non-SCC	C/T	G/G	A/A
Non-SCC-0119	2	67	0	0	0	Non-SCC	C/T	G/C	A/A
Non-SCC-0120	2	64	0	0	0	Non-SCC	T/T	C/C	A/A
Non-SCC-0121	2	70	0	0	1	Non-SCC	T/T	G/C	A/A
Non-SCC-0122	1	87	0	0	1	Non-SCC	C/T	G/G	A/A
Non-SCC-0123	1	66	1	0	1	Non-SCC	C/T	G/C	G/A
Non-SCC-0124	1	46	1	0	0	Non-SCC	T/T	G/C	A/A
Non-SCC-0125	2	59	0	0	1	Non-SCC	C/C	G/C	G/A
Non-SCC-0126	2	58	0	0	0	Non-SCC	T/T	C/C	G/A
Non-SCC-0127	1	46	0	0	0	Non-SCC	C/T	G/C	G/A
Non-SCC-0128	1	62	1	0	0	Non-SCC	C/T	C/C	A/A
Non-SCC-0129	1	76	0	0	1	Non-SCC	C/T	C/C	G/G
Non-SCC-0130	2	46	0	0	1	Non-SCC	T/T	C/C	A/A
Non-SCC-0131	2	78	0	0	0	Non-SCC	T/T	G/C	G/A
Non-SCC-0132	2	51	0	0	0	Non-SCC	T/T	G/C	A/A
Non-SCC-0133	1	60	1	0	0	Non-SCC	T/T	G/C	A/A
Non-SCC-0134	1	43	0	0	0	Non-SCC	C/T	G/C	G/A
Non-SCC-0135	1	53	1	0	1	Non-SCC	C/T	G/G	A/A
Non-SCC-0136	1	52	1	0	0	Non-SCC	C/T	G/C	G/A
Non-SCC-0137	1	58	0	1	0	Non-SCC	C/C	G/G	A/A
Non-SCC-0138	1	60	0	0	1	Non-SCC	T/T	C/C	A/A
Non-SCC-0139	2	53	0	0	1	Non-SCC	C/C	G/C	A/A
Non-SCC-0140	2	81	0	0	0	Non-SCC	C/T	G/C	G/A
Non-SCC-0141	2	66	0	0	0	Non-SCC	T/T	G/G	G/A
Non-SCC-0142	2	62	0	0	0	Non-SCC	C/T	C/C	A/A
Non-SCC-0143	2	48	0	0	0	Non-SCC	T/T	G/C	A/A
Non-SCC-0144	2	39	0	0	0	Non-SCC	T/T	C/C	A/A
Non-SCC-0145	2	39	0	0	0	Non-SCC	C/T	G/C	A/A
Non-SCC-0146	1	66	1	0	0	Non-SCC	C/T	G/C	G/A
Non-SCC-0147	1	37	0	0	1	Non-SCC	C/C	C/C	A/A

Non-SCC-0148	1	61	1	0	0	Non-SCC	T/T	G/C	G/A
Non-SCC-0149	1	43	1	1	1	Non-SCC	C/T	G/C	G/A
Non-SCC-0150	1	73	1	0	0	Non-SCC	C/T	C/C	G/A
Non-SCC-0151	1	61	1	0	0	Non-SCC	T/T	G/C	G/A
Non-SCC-0152	1	76	0	0	1	Non-SCC	C/T	G/C	A/A
Non-SCC-0153	2	68	0	0	0	Non-SCC	C/C	G/C	A/A
Non-SCC-0154	2	63	0	0	0	Non-SCC	C/T	G/G	A/A
Non-SCC-0155	1	52	1	0	0	Non-SCC	C/T	C/C	A/A
Non-SCC-0156	1	74	1	0	0	Non-SCC	C/C	C/C	A/A
Non-SCC-0157	1	43	1	0	1	Non-SCC	C/T	G/C	A/A
Non-SCC-0158	1	61	1	0	1	Non-SCC	T/T	C/C	G/A
Non-SCC-0159	2	75	0	0	1	Non-SCC	T/T	G/G	A/A
Non-SCC-0160	2	53	0	0	0	Non-SCC	C/C	G/G	A/A
Non-SCC-0161	2	38	0	0	0	Non-SCC	C/T	G/C	A/A
Non-SCC-0162	2	68	0	0	0	Non-SCC	C/T	G/C	A/A
Non-SCC-0163	2	28	0	0	0	Non-SCC	C/T	C/C	A/A
Non-SCC-0164	1	56	1	0	0	Non-SCC	C/C	G/C	G/A
Non-SCC-0165	2	68	0	0	1	Non-SCC	T/T	G/C	A/A
Non-SCC-0166	1	63	1	0	1	Non-SCC	T/T	G/C	A/A
Non-SCC-0167	2	50	0	0	1	Non-SCC	C/T	C/C	G/A
Non-SCC-0168	2	50	0	0	1	Non-SCC	C/T	G/G	A/A
Non-SCC-0169	2	70	0	0	0	Non-SCC	C/T	G/C	A/A
Non-SCC-0170	2	46	0	0	0	Non-SCC	T/T	C/C	G/A
Non-SCC-0171	1	62	1	0	0	Non-SCC	T/T	G/C	A/A
Non-SCC-0172	1	44	1	0	1	Non-SCC	T/T	C/C	A/A
Non-SCC-0173	2	55	0	0	0	Non-SCC	C/C	C/C	A/A
Non-SCC-0174	1	61	1	0	0	Non-SCC	C/T	G/C	G/A
Non-SCC-0175	1	50	1	0	0	Non-SCC	C/C	G/C	A/A
Non-SCC-0176	2	61	0	0	0	Non-SCC	C/C	C/C	A/A
Non-SCC-0177	1	58	1	0	1	Non-SCC	T/T	G/G	A/A
Non-SCC-0178	2	76	0	0	1	Non-SCC	T/T	G/G	G/A
Non-SCC-0179	2	63	0	0	0	Non-SCC	C/T	G/C	G/A
Non-SCC-0180	1	59	0	0	0	Non-SCC	C/T	G/C	A/A
Non-SCC-0181	1	70	1	0	1	Non-SCC	C/T	C/C	A/A
Non-SCC-0182	2	57	0	0	1	Non-SCC	C/T	G/C	A/A
Non-SCC-0183	1	56	1	0	0	Non-SCC	C/T	C/C	A/A
Non-SCC-0184	1	52	1	0	1	Non-SCC	T/T	C/C	G/A
Non-SCC-0185	2	50	0	0	0	Non-SCC	T/T	G/C	G/A
Non-SCC-0186	2	62	0	0	0	Non-SCC	C/T	G/C	G/G
Non-SCC-0187	1	51	1	0	1	Non-SCC	C/T	C/C	A/A
Non-SCC-0188	2	45	0	0	0	Non-SCC	C/T	C/C	G/A
Non-SCC-0189	1	60	1	0	1	Non-SCC	T/T	G/C	A/A
Non-SCC-0190	1	53	0	0	1	Non-SCC	T/T	G/G	A/A
Non-SCC-0191	1	59	0	0	1	Non-SCC	C/C	C/C	G/G
Non-SCC-0192	2	52	0	0	0	Non-SCC	C/C	C/C	A/A
Non-SCC-0193	2	52	0	0	1	Non-SCC	C/T	C/C	A/A
Non-SCC-0194	1	61	1	0	0	Non-SCC	T/T	G/G	A/A
Non-SCC-0195	1	44	1	0	0	Non-SCC	C/C	C/C	G/A
Non-SCC-0196	2	60	0	0	1	Non-SCC	T/T	C/C	A/A
Non-SCC-0197	1	59	0	0	0	Non-SCC	T/T	C/C	A/A
Non-SCC-0198	1	52	1	0	1	Non-SCC	C/T	G/C	G/A
Non-SCC-0199	1	67	1	1	1	Non-SCC	T/T	G/C	A/A
Non-SCC-0200	1	75	0	0	0	Non-SCC	T/T	G/C	G/A
Non-SCC-0201	2	63	0	0	1	Non-SCC	T/T	C/C	G/G
Non-SCC-0202	2	49	0	0	1	Non-SCC	T/T	G/C	G/A
Non-SCC-0203	2	36	0	0	0	Non-SCC	T/T	G/C	G/A
Non-SCC-0204	2	56	0	0	0	Non-SCC	T/T	G/C	A/A
Non-SCC-0205	2	65	0	0	0	Non-SCC	T/T	C/C	G/A
Non-SCC-0206	1	48	1	0	1	Non-SCC	C/C	G/G	A/A
Non-SCC-0207	2	49	0	0	0	Non-SCC	T/T	C/C	A/A
Non-SCC-0208	2	60	0	0	0	Non-SCC	C/T	C/C	A/A
Non-SCC-0209	1	69	1	0	0	Non-SCC	C/T	G/C	A/A
Non-SCC-0210	2	59	0	0	0	Non-SCC	C/T	G/C	G/A
Non-SCC-0211	1	76	1	0	0	Non-SCC	C/T	C/C	A/A
Non-SCC-0212	1	63	1	1	0	Non-SCC	C/T	C/C	A/A
Non-SCC-0213	2	60	0	0	0	Non-SCC	T/T	G/C	G/A
Non-SCC-0214	2	59	0	0	0	Non-SCC	C/T	G/C	G/A
Non-SCC-0215	2	35	0	0	0	Non-SCC	C/T	G/C	A/A
Non-SCC-0216	2	64	0	0	1	Non-SCC	T/T	G/C	A/A
Non-SCC-0217	2	53	0	0	1	Non-SCC	C/T	C/C	A/A
Non-SCC-0218	2	62	0	0	0	Non-SCC	C/T	G/C	A/A
Non-SCC-0219	1	69	0	0	0	Non-SCC	T/T	C/C	A/A
Non-SCC-0220	1	40	1	0	1	Non-SCC	C/T	G/C	A/A
Non-SCC-0221	1	45	0	0	1	Non-SCC	C/C	G/C	A/A
Non-SCC-0222	2	52	0	0	1	Non-SCC	C/T	C/C	A/A
Non-SCC-0223	1	46	1	0	1	Non-SCC	C/T	C/C	G/A
Non-SCC-0224	2	44	0	0	1	Non-SCC	C/T	G/C	A/A

Non-SCC-0225	1	49	1	1	0	Non-SCC	C/T	G/C	A/A
Non-SCC-0226	1	58	0	0	0	Non-SCC	T/T	C/C	G/A
Non-SCC-0227	2	52	0	0	1	Non-SCC	C/T	G/C	G/A
Non-SCC-0228	2	67	0	0	0	Non-SCC	C/T	C/C	A/A
Non-SCC-0229	1	52	1	1	0	Non-SCC	C/T	C/C	A/A
Non-SCC-0230	2	67	0	0	0	Non-SCC	T/T	G/G	G/A
Non-SCC-0231	2	67	0	0	0	Non-SCC	C/C	C/C	A/A
Non-SCC-0232	1	59	1	0	0	Non-SCC	C/C	C/C	A/A
Non-SCC-0233	2	63	0	0	0	Non-SCC	C/T	C/C	G/A
Non-SCC-0234	1	54	1	0	1	Non-SCC	T/T	C/C	A/A
Non-SCC-0235	2	61	0	0	0	Non-SCC	T/T	G/C	A/A
Non-SCC-0236	1	57	0	0	1	Non-SCC	C/T	C/C	A/A
Non-SCC-0237	2	69	0	0	0	Non-SCC	C/C	G/G	A/A
Non-SCC-0238	1	67	0	0	0	Non-SCC	C/C	C/C	G/A
Non-SCC-0239	2	60	0	0	0	Non-SCC	C/T	C/C	A/A
Non-SCC-0240	2	72	0	0	0	Non-SCC	T/T	G/G	A/A
Non-SCC-0241	1	70	0	0	1	Non-SCC	T/T	G/C	A/A
Non-SCC-0242	2	46	0	0	0	Non-SCC	T/T	G/C	G/A
Non-SCC-0243	1	61	0	0	1	Non-SCC	C/T	G/C	A/A
Non-SCC-0244	1	62	1	1	0	Non-SCC	C/T	C/C	A/A
Non-SCC-0245	1	65	1	0	1	Non-SCC	C/C	C/C	A/A
Non-SCC-0246	1	77	1	1	0	Non-SCC	C/C	C/C	A/A
Non-SCC-0247	2	38	0	0	0	Non-SCC	C/C	G/C	A/A
Non-SCC-0248	2	44	0	0	0	Non-SCC	C/T	C/C	A/A
Non-SCC-0249	1	60	0	1	1	Non-SCC	C/C	G/C	A/A
Non-SCC-0250	2	43	0	0	1	Non-SCC	C/T	G/G	A/A
Non-SCC-0251	2	57	0	0	0	Non-SCC	T/T	C/C	G/A
Non-SCC-0252	2	69	0	0	0	Non-SCC	T/T	C/C	A/A
Non-SCC-0253	2	65	0	0	1	Non-SCC	C/C	C/C	G/A
Non-SCC-0254	2	62	0	0	1	Non-SCC	C/T	G/C	A/A
Non-SCC-0255	1	58	1	0	0	Non-SCC	C/T	C/C	A/A
Non-SCC-0256	1	74	1	0	1	Non-SCC	T/T	G/C	A/A
Non-SCC-0257	1	56	1	1	1	Non-SCC	C/T	C/C	A/A
Non-SCC-0258	2	60	0	0	0	Non-SCC	C/C	G/C	A/A
Non-SCC-0259	1	27	0	1	0	Non-SCC	C/T	G/C	A/A
Non-SCC-0260	1	48	1	0	0	Non-SCC	T/T	G/C	G/A
Non-SCC-0261	2	73	0	0	0	Non-SCC	C/C	G/C	A/A
Non-SCC-0262	2	59	0	0	1	Non-SCC	C/T	G/C	G/G
Non-SCC-0263	1	64	0	0	0	Non-SCC	C/T	C/C	G/A
Non-SCC-0264	2	59	0	0	0	Non-SCC	C/C	G/G	G/A
Non-SCC-0265	1	63	1	1	0	Non-SCC	C/C	C/C	A/A
Non-SCC-0266	2	59	0	0	0	Non-SCC	C/C	G/C	G/A
Non-SCC-0267	2	60	0	0	0	Non-SCC	T/T	G/G	A/A
Non-SCC-0268	1	62	1	1	0	Non-SCC	T/T	G/C	A/A
Non-SCC-0269	2	66	0	0	1	Non-SCC	C/T	C/C	G/A
Non-SCC-0270	2	74	0	0	0	Non-SCC	C/T	C/C	A/A
Non-SCC-0271	1	65	1	1	0	Non-SCC	T/T	G/C	A/A
Non-SCC-0272	1	63	0	0	1	Non-SCC	T/T	G/C	A/A
Non-SCC-0273	2	67	0	0	1	Non-SCC	C/T	C/C	G/G
Non-SCC-0274	1	52	1	0	0	Non-SCC	C/C	C/C	A/A
Non-SCC-0275	2	61	0	0	0	Non-SCC	C/T	G/G	A/A
Non-SCC-0276	1	67	0	0	0	Non-SCC	C/T	G/C	G/A
Non-SCC-0277	1	72	0	0	0	Non-SCC	C/T	G/C	G/A
Non-SCC-0278	1	70	1	1	1	Non-SCC	C/C	G/C	A/A
Non-SCC-0279	2	59	0	0	0	Non-SCC	C/T	G/C	G/A
Non-SCC-0280	1	56	0	1	1	Non-SCC	C/T	C/C	G/A
Non-SCC-0281	1	76	1	1	0	Non-SCC	T/T	C/C	G/A
Non-SCC-0282	1	74	0	0	0	Non-SCC	C/T	C/C	A/A
Non-SCC-0283	2	26	0	0	1	Non-SCC	T/T	C/C	A/A
Non-SCC-0284	2	56	0	0	1	Non-SCC	T/T	C/C	G/G
Non-SCC-0285	1	48	0	0	1	Non-SCC	C/T	G/C	A/A
Non-SCC-0286	2	65	0	0	0	Non-SCC	C/T	G/C	G/A
Non-SCC-0287	2	51	0	0	0	Non-SCC	C/T	G/C	A/A
Non-SCC-0288	1	58	1	1	1	Non-SCC	C/T	G/C	A/A
Non-SCC-0289	2	44	0	0	1	Non-SCC	C/T	G/C	A/A
Non-SCC-0290	1	73	0	0	1	Non-SCC	C/T	G/C	G/A
Non-SCC-0291	2	70	0	0	1	Non-SCC	C/T	G/C	G/A
Non-SCC-0292	1	64	1	0	0	Non-SCC	T/T	C/C	A/A
Non-SCC-0293	2	49	0	0	0	Non-SCC	C/T	G/C	A/A
Non-SCC-0294	1	42	0	1	1	Non-SCC	C/C	G/C	A/A
Non-SCC-0295	1	79	0	0	1	Non-SCC	C/T	C/C	A/A
Non-SCC-0296	1	52	1	0	0	Non-SCC	C/T	G/C	G/A
Non-SCC-0297	2	41	0	0	0	Non-SCC	C/T	G/G	G/A
Non-SCC-0298	1	40	0	1	0	Non-SCC	C/C	G/G	G/A
Non-SCC-0299	2	61	0	0	1	Non-SCC	T/T	G/C	A/A
Non-SCC-0300	1	63	1	1	1	Non-SCC	C/T	G/G	G/A
Non-SCC-0301	1	62	1	1	0	Non-SCC	T/T	G/C	A/A

Non-SCC-0302	2	40	0	0	1	Non-SCC	C/C	G/C	G/A
Non-SCC-0303	2	49	0	0	0	Non-SCC	T/T	G/C	A/A
Non-SCC-0304	2	44	0	0	0	Non-SCC	C/T	G/C	A/A
Non-SCC-0305	2	54	0	0	1	Non-SCC	C/T	G/C	A/A
Non-SCC-0306	2	61	0	0	1	Non-SCC	C/T	G/C	A/A
Non-SCC-0307	2	74	0	0	0	Non-SCC	C/T	G/C	A/A
Non-SCC-0308	1	59	1	1	1	Non-SCC	T/T	C/C	A/A
Non-SCC-0309	1	68	0	0	0	Non-SCC	C/C	G/C	A/A
Non-SCC-0310	1	72	1	0	1	Non-SCC	C/C	G/C	G/A
Non-SCC-0311	1	52	1	1	1	Non-SCC	T/T	C/C	A/A
Non-SCC-0312	1	59	1	1	1	Non-SCC	C/T	G/C	A/A
Non-SCC-0313	2	42	0	0	0	Non-SCC	C/C	C/C	A/A
Non-SCC-0314	2	62	0	0	0	Non-SCC	C/C	G/C	G/A
Non-SCC-0315	2	28	0	0	0	Non-SCC	C/T	G/C	A/A
Non-SCC-0316	2	66	0	0	1	Non-SCC	T/T	C/C	A/A
Non-SCC-0317	2	45	0	0	0	Non-SCC	T/T	G/C	A/A
Non-SCC-0318	1	41	0	0	0	Non-SCC	T/T	C/C	A/A
Non-SCC-0319	1	66	1	1	0	Non-SCC	C/T	G/C	A/A
Non-SCC-0320	2	57	0	0	0	Non-SCC	C/T	C/C	A/A
Non-SCC-0321	1	72	1	0	1	Non-SCC	C/T	G/C	A/A
Non-SCC-0322	1	66	0	0	0	Non-SCC	C/T	G/C	A/A
Non-SCC-0323	2	46	0	0	1	Non-SCC	T/T	G/C	A/A
Non-SCC-0324	1	66	0	0	1	Non-SCC	C/C	G/C	A/A
Non-SCC-0325	1	70	0	0	1	Non-SCC	C/T	G/G	A/A
Non-SCC-0326	1	48	1	0	0	Non-SCC	C/T	G/C	A/A
Non-SCC-0327	2	57	0	0	0	Non-SCC	C/T	G/G	G/A
Non-SCC-0328	1	64	1	0	0	Non-SCC	C/T	G/G	A/A
Non-SCC-0329	2	53	0	0	0	Non-SCC	T/T	G/G	A/A
Non-SCC-0330	1	67	1	1	1	Non-SCC	C/T	G/C	A/A
Non-SCC-0331	2	55	0	0	1	Non-SCC	C/T	G/C	G/A
Non-SCC-0332	2	69	0	0	0	Non-SCC	C/C	G/G	A/A
Non-SCC-0333	2	48	0	0	1	Non-SCC	C/T	G/C	G/A
Non-SCC-0334	2	81	0	0	0	Non-SCC	C/T	G/C	A/A
Non-SCC-0335	2	43	0	0	0	Non-SCC	T/T	G/C	A/A
Non-SCC-0336	1	57	1	0	1	Non-SCC	C/C	G/C	A/A
Non-SCC-0337	2	46	0	0	0	Non-SCC	T/T	C/C	A/A
Non-SCC-0338	2	61	0	0	0	Non-SCC	C/T	C/C	A/A
Non-SCC-0339	2	36	0	0	0	Non-SCC	C/C	C/C	A/A
Non-SCC-0340	2	70	0	0	0	Non-SCC	T/T	G/C	G/A
Non-SCC-0341	1	67	1	1	0	Non-SCC	T/T	G/C	A/A
Non-SCC-0342	1	56	1	1	1	Non-SCC	T/T	C/C	G/A
Non-SCC-0343	2	60	1	1	1	Non-SCC	T/T	C/C	A/A
Non-SCC-0344	2	66	0	0	0	Non-SCC	C/T	G/C	A/A
Non-SCC-0345	1	69	1	1	0	Non-SCC	C/T	C/C	G/A
Non-SCC-0346	1	55	1	1	0	Non-SCC	T/T	G/G	A/A
Non-SCC-0347	2	58	0	0	0	Non-SCC	T/T	C/C	A/A
Non-SCC-0348	2	54	0	0	1	Non-SCC	C/T	G/C	A/A
Non-SCC-0349	1	54	1	1	0	Non-SCC	T/T	G/C	G/A
Non-SCC-0350	1	59	0	0	0	Non-SCC	C/T	C/C	A/A
Non-SCC-0351	2	53	0	0	0	Non-SCC	C/C	C/C	A/A
Non-SCC-0352	1	71	1	1	0	Non-SCC	C/T	G/C	G/A
Non-SCC-0353	1	63	1	1	1	Non-SCC	T/T	G/C	A/A
Non-SCC-0354	2	80	0	0	0	Non-SCC	C/T	C/C	G/A
Non-SCC-0355	1	80	1	1	0	Non-SCC	C/T	G/C	A/A
Non-SCC-0356	1	40	0	0	0	Non-SCC	C/C	G/C	A/A
Non-SCC-0357	2	51	0	0	1	Non-SCC	T/T	G/G	A/A
Non-SCC-0358	2	49	0	0	1	Non-SCC	C/C	G/C	G/A
Non-SCC-0359	2	62	0	0	0	Non-SCC	T/T	C/C	G/A
Non-SCC-0360	1	59	0	1	0	Non-SCC	T/T	C/C	A/A
Non-SCC-0361	2	68	0	0	1	Non-SCC	T/T	G/C	A/A
Non-SCC-0362	2	63	0	0	1	Non-SCC	C/T	C/C	A/A
Non-SCC-0363	1	58	0	0	0	Non-SCC	C/C	G/C	A/A
Non-SCC-0364	2	32	0	0	0	Non-SCC	C/C	C/C	A/A
Non-SCC-0365	1	79	0	0	0	Non-SCC	T/T	G/G	G/A
Non-SCC-0366	2	74	0	0	1	Non-SCC	C/C	G/C	A/A
Non-SCC-0367	1	74	1	0	0	Non-SCC	C/C	G/C	A/A
Non-SCC-0368	1	60	1	0	1	Non-SCC	C/T	C/C	A/A
Non-SCC-0369	2	55	0	0	0	Non-SCC	T/T	G/C	G/A
Non-SCC-0370	1	64	1	1	0	Non-SCC	C/T	C/C	A/A
Non-SCC-0371	1	70	1	1	0	Non-SCC	T/T	G/C	G/A
Non-SCC-0372	1	76	0	0	0	Non-SCC	C/C	C/C	G/G
Non-SCC-0373	1	62	0	1	0	Non-SCC	T/T	G/C	G/A
Non-SCC-0374	1	78	0	0	1	Non-SCC	T/T	G/C	A/A
Non-SCC-0375	1	69	0	0	0	Non-SCC	C/T	G/C	G/A
Non-SCC-0376	2	74	0	0	0	Non-SCC	C/T	G/C	G/A
Non-SCC-0377	1	57	0	0	0	Non-SCC	C/T	G/C	A/A
Non-SCC-0378	1	67	1	0	0	Non-SCC	T/T	G/G	A/A

Non-SCC-0379	2	57	0	0	0	Non-SCC	T/T	G/C	G/A
Non-SCC-0380	2	26	0	0	1	Non-SCC	C/T	G/C	A/A
Non-SCC-0381	1	61	1	1	0	Non-SCC	T/T	G/C	A/A
Non-SCC-0382	2	79	0	0	0	Non-SCC	C/T	G/C	A/A
Non-SCC-0383	1	49	1	1	1	Non-SCC	T/T	G/C	A/A
Non-SCC-0384	1	61	0	1	0	Non-SCC	C/T	G/G	A/A
Non-SCC-0385	1	58	1	1	0	Non-SCC	C/T	C/C	A/A
Non-SCC-0386	1	66	1	0	0	Non-SCC	C/T	C/C	G/A
Non-SCC-0387	2	46	0	0	0	Non-SCC	C/C	C/C	A/A
Non-SCC-0388	2	75	0	0	0	Non-SCC	T/T	G/C	A/A
Non-SCC-0389	1	61	0	0	0	Non-SCC	C/T	C/C	A/A
Non-SCC-0390	1	64	0	0	0	Non-SCC	C/T	C/C	A/A
Non-SCC-0391	2	46	0	0	0	Non-SCC	C/T	G/C	G/A
Non-SCC-0392	1	82	0	0	1	Non-SCC	T/T	C/C	G/A
Non-SCC-0393	1	71	1	0	0	Non-SCC	C/T	G/C	A/A
Non-SCC-0394	1	61	0	0	1	Non-SCC	C/T	G/C	G/A
Non-SCC-0395	2	71	0	0	0	Non-SCC	T/T	G/G	A/A
Non-SCC-0396	2	67	0	1	1	Non-SCC	C/C	C/C	A/A
Non-SCC-0397	1	52	1	1	0	Non-SCC	C/T	G/C	A/A
Non-SCC-0398	2	64	0	0	0	Non-SCC	C/T	G/C	G/A
Non-SCC-0399	2	46	0	0	0	Non-SCC	C/C	G/C	G/A
Non-SCC-0400	2	61	0	0	1	Non-SCC	T/T	G/C	G/A
Non-SCC-0401	2	60	0	0	0	Non-SCC	C/T	G/C	G/A
Non-SCC-0402	1	53	1	1	1	Non-SCC	C/T	C/C	A/A
Non-SCC-0403	1	75	0	0	0	Non-SCC	T/T	G/C	A/A
Non-SCC-0404	1	79	0	0	1	Non-SCC	C/T	G/C	A/A
Non-SCC-0405	1	70	1	1	0	Non-SCC	C/T	G/C	G/A
Non-SCC-0406	1	61	1	0	0	Non-SCC	C/T	C/C	A/A
Non-SCC-0407	1	68	1	1	0	Non-SCC	C/T	G/C	A/A
Non-SCC-0408	2	59	0	0	0	Non-SCC	C/T	G/C	A/A
Non-SCC-0409	2	55	0	0	1	Non-SCC	C/T	C/C	A/A
Non-SCC-0410	2	59	0	0	1	Non-SCC	C/T	C/C	A/A
Non-SCC-0411	1	70	0	0	1	Non-SCC	T/T	G/C	A/A
Non-SCC-0412	1	52	1	0	0	Non-SCC	C/T	G/C	A/A
Non-SCC-0413	1	65	1	0	0	Non-SCC	T/T	G/C	G/A
Non-SCC-0414	2	58	0	0	0	Non-SCC	T/T	G/C	A/A
Non-SCC-0415	1	74	1	1	0	Non-SCC	C/C	G/C	A/A
Non-SCC-0416	2	66	0	0	0	Non-SCC	C/T	C/C	A/A
Non-SCC-0417	2	56	0	0	0	Non-SCC	C/C	C/C	A/A
Non-SCC-0418	2	67	0	0	0	Non-SCC	C/T	G/C	A/A
Non-SCC-0419	1	54	1	0	0	Non-SCC	T/T	C/C	A/A
Non-SCC-0420	2	59	0	0	1	Non-SCC	C/C	C/C	A/A
Non-SCC-0421	1	63	0	0	0	Non-SCC	C/T	G/C	G/A
Non-SCC-0422	2	65	1	0	1	Non-SCC	T/T	G/G	A/A
Non-SCC-0423	1	67	0	0	1	Non-SCC	C/T	G/C	A/A
Non-SCC-0424	1	63	0	0	1	Non-SCC	C/T	G/C	A/A
Non-SCC-0425	1	62	1	0	1	Non-SCC	C/T	G/G	G/A
Non-SCC-0426	1	81	0	0	1	Non-SCC	T/T	C/C	A/A
Non-SCC-0427	2	51	0	0	1	Non-SCC	T/T	G/G	A/A
Non-SCC-0428	2	52	0	0	0	Non-SCC	T/T	G/C	A/A
Non-SCC-0429	2	48	0	0	1	Non-SCC	T/T	G/G	G/A
Non-SCC-0430	1	67	1	1	0	Non-SCC	C/T	G/C	G/A
Non-SCC-0431	1	62	1	1	0	Non-SCC	T/T	G/C	A/A
Non-SCC-0432	1	66	1	1	0	Non-SCC	C/T	G/C	A/A
Non-SCC-0433	2	71	0	0	0	Non-SCC	T/T	C/C	A/A
Non-SCC-0434	2	61	0	0	1	Non-SCC	C/C	G/C	A/A
Non-SCC-0435	1	43	0	1	0	Non-SCC	C/T	C/C	A/A
Non-SCC-0436	1	66	1	1	0	Non-SCC	T/T	C/C	A/A
Non-SCC-0437	1	71	1	0	1	Non-SCC	C/C	G/G	A/A
Non-SCC-0438	2	72	0	0	0	Non-SCC	C/T	C/C	A/A
Non-SCC-0439	1	69	1	0	0	Non-SCC	T/T	C/C	A/A
Non-SCC-0440	1	85	0	1	1	Non-SCC	C/C	C/C	A/A
Non-SCC-0441	2	65	0	0	1	Non-SCC	C/T	G/C	A/A
Non-SCC-0442	2	64	0	0	1	Non-SCC	T/T	G/C	A/A
Non-SCC-0443	1	35	1	0	0	Non-SCC	T/T	C/C	G/A
Non-SCC-0444	2	48	0	0	1	Non-SCC	?	?	?
Non-SCC-0445	2	55	0	0	1	Non-SCC	C/T	C/C	A/A
Non-SCC-0446	2	40	0	0	0	Non-SCC	?	?	?
Non-SCC-0447	2	46	0	0	0	Non-SCC	C/T	G/C	G/A
Non-SCC-0448	1	68	0	1	0	Non-SCC	C/T	G/G	A/A
Non-SCC-0449	2	71	0	0	1	Non-SCC	C/T	C/C	A/A
Non-SCC-0450	2	37	0	0	1	Non-SCC	C/T	G/G	G/G
Non-SCC-0451	2	64	0	0	0	Non-SCC	T/T	G/G	A/A
Non-SCC-0452	1	44	1	1	1	Non-SCC	T/T	C/C	A/A
Non-SCC-0453	1	46	0	0	1	Non-SCC	T/T	C/C	A/A
Non-SCC-0454	1	64	1	1	0	Non-SCC	T/T	G/C	A/A
Non-SCC-0455	1	54	1	0	1	Non-SCC	C/T	G/C	A/A

Non-SCC-0456	2	63	0	0	0	Non-SCC	C/T	G/C	A/A
Non-SCC-0457	2	71	0	0	0	Non-SCC	C/T	C/C	A/A
Non-SCC-0458	2	53	0	0	0	Non-SCC	C/T	G/G	G/A
Non-SCC-0459	2	49	0	0	0	Non-SCC	C/T	G/C	G/A
Non-SCC-0460	2	35	0	0	0	Non-SCC	C/C	G/C	G/A
Non-SCC-0461	2	48	0	0	0	Non-SCC	C/T	C/C	G/A
Non-SCC-0462	1	68	0	1	0	Non-SCC	T/T	G/C	A/A
Non-SCC-0463	2	80	0	0	1	Non-SCC	C/T	G/G	A/A
Non-SCC-0464	2	53	0	0	0	Non-SCC	C/T	G/C	A/A
Non-SCC-0465	1	55	0	0	1	Non-SCC	T/T	C/C	A/A
Non-SCC-0466	1	33	0	0	0	Non-SCC	C/C	C/C	A/A
Non-SCC-0467	2	64	0	0	1	Non-SCC	C/T	G/G	A/A
Non-SCC-0468	2	63	0	0	1	Non-SCC	C/T	G/G	A/A
Non-SCC-0469	1	81	0	0	0	Non-SCC	C/T	C/C	G/A
Non-SCC-0470	2	68	0	0	0	Non-SCC	C/T	G/C	A/A
Non-SCC-0471	1	63	0	0	1	Non-SCC	C/T	C/C	G/A
Non-SCC-0472	2	62	0	0	0	Non-SCC	C/T	G/C	A/A
Non-SCC-0473	2	53	0	0	0	Non-SCC	C/C	C/C	G/A
Non-SCC-0474	2	56	0	0	0	Non-SCC	T/T	C/C	A/A
Non-SCC-0475	1	61	1	1	0	Non-SCC	C/C	G/C	A/A
Non-SCC-0476	1	55	0	0	0	Non-SCC	T/T	C/C	G/G
Non-SCC-0477	1	43	0	0	0	Non-SCC	T/T	C/C	A/A
Non-SCC-0478	1	67	1	1	1	Non-SCC	C/T	C/C	A/A
Non-SCC-0479	2	49	0	0	0	Non-SCC	T/T	G/G	A/A
Non-SCC-0480	2	63	0	0	0	Non-SCC	C/T	C/C	A/A
Non-SCC-0481	1	64	1	1	0	Non-SCC	C/T	C/C	A/A
Non-SCC-0482	2	50	0	0	0	Non-SCC	T/T	G/C	A/A
Non-SCC-0483	1	72	0	0	1	Non-SCC	T/T	G/C	G/A
Non-SCC-0484	2	66	0	0	1	Non-SCC	T/T	C/C	A/A
Non-SCC-0485	2	57	0	0	0	Non-SCC	C/T	G/C	G/A
Non-SCC-0486	1	43	1	0	0	Non-SCC	T/T	G/G	G/A
Non-SCC-0487	2	62	0	0	1	Non-SCC	T/T	C/C	A/A
Non-SCC-0488	1	52	0	0	0	Non-SCC	T/T	G/C	A/A
Non-SCC-0489	2	54	0	0	0	Non-SCC	T/T	G/C	A/A
Non-SCC-0490	1	42	0	0	1	Non-SCC	C/T	G/C	A/A
Non-SCC-0491	1	62	1	1	0	Non-SCC	C/T	G/C	A/A
Non-SCC-0492	1	54	0	0	0	Non-SCC	C/T	G/C	G/A
Non-SCC-0493	1	61	0	1	0	Non-SCC	T/T	G/C	A/A
Non-SCC-0494	1	75	1	1	0	Non-SCC	T/T	G/C	A/A
Non-SCC-0495	2	48	0	0	0	Non-SCC	C/T	G/C	A/A
Non-SCC-0496	1	66	1	1	1	Non-SCC	T/T	C/C	A/A
Non-SCC-0497	2	52	1	1	0	Non-SCC	C/T	G/C	G/A
Non-SCC-0498	1	50	0	0	0	Non-SCC	C/T	C/C	G/A
Non-SCC-0499	2	55	0	0	0	Non-SCC	T/T	G/C	A/A
Non-SCC-0500	1	61	1	1	0	Non-SCC	C/T	C/C	A/A
Non-SCC-0501	2	53	0	0	0	Non-SCC	C/C	G/G	G/A
Non-SCC-0502	2	53	0	0	1	Non-SCC	C/T	G/C	G/A
Non-SCC-0503	1	66	0	0	0	Non-SCC	C/C	G/C	A/A
Non-SCC-0504	1	68	0	0	0	Non-SCC	C/C	G/C	A/A
Non-SCC-0505	2	62	0	0	0	Non-SCC	T/T	G/C	A/A
Non-SCC-0506	2	48	0	0	0	Non-SCC	T/T	G/C	G/A
Non-SCC-0507	1	57	1	1	0	Non-SCC	C/T	G/C	A/A
Non-SCC-0508	2	44	0	0	1	Non-SCC	C/T	C/C	A/A
Non-SCC-0509	2	64	0	0	1	Non-SCC	C/T	G/C	G/A
Non-SCC-0510	1	55	1	1	1	Non-SCC	C/T	C/C	A/A
Non-SCC-0511	2	63	0	0	0	Non-SCC	C/C	G/C	G/G
Non-SCC-0512	2	66	0	0	1	Non-SCC	T/T	G/C	A/A
Non-SCC-0513	1	33	1	1	1	Non-SCC	C/T	C/C	G/A
Non-SCC-0514	2	53	0	0	1	Non-SCC	T/T	C/C	A/A
Non-SCC-0515	2	76	0	0	0	Non-SCC	C/T	C/C	A/A
Non-SCC-0516	2	54	0	0	0	Non-SCC	C/T	C/C	A/A
Non-SCC-0517	2	53	0	0	1	Non-SCC	T/T	G/C	G/A
Non-SCC-0518	2	57	0	0	0	Non-SCC	C/T	G/G	A/A
Non-SCC-0519	1	62	0	0	0	Non-SCC	C/T	C/C	A/A
Non-SCC-0520	1	78	1	1	0	Non-SCC	C/C	C/C	A/A
Non-SCC-0521	1	51	0	0	1	Non-SCC	C/T	C/C	A/A
Non-SCC-0522	2	48	0	0	0	Non-SCC	C/T	C/C	A/A
Non-SCC-0523	2	78	0	0	1	Non-SCC	C/C	C/C	A/A
Non-SCC-0524	2	63	0	0	1	Non-SCC	C/T	C/C	G/A
Non-SCC-0525	2	75	0	0	1	Non-SCC	C/T	G/G	A/A
Non-SCC-0526	2	46	0	0	0	Non-SCC	T/T	G/G	A/A
Non-SCC-0527	1	47	1	0	0	Non-SCC	C/T	C/C	A/A
Non-SCC-0528	2	65	0	0	0	Non-SCC	C/T	G/C	G/A
Non-SCC-0529	2	81	0	0	1	Non-SCC	C/T	G/C	G/A
Non-SCC-0530	2	54	0	0	0	Non-SCC	C/T	G/C	G/A
Non-SCC-0531	1	54	0	1	0	Non-SCC	C/T	C/C	A/A
Non-SCC-0532	1	62	1	0	0	Non-SCC	C/C	C/C	A/A

Non-SCC-0533	2	39	0	0	0	Non-SCC	C/C	G/C	G/G
Non-SCC-0534	1	54	1	1	1	Non-SCC	C/C	G/C	A/A
Non-SCC-0535	1	54	1	1	0	Non-SCC	C/T	G/C	A/A
Non-SCC-0536	2	48	0	0	1	Non-SCC	C/T	G/C	G/A
Non-SCC-0537	1	59	1	1	0	Non-SCC	C/T	G/C	A/A
Non-SCC-0538	1	77	0	0	0	Non-SCC	C/C	G/G	A/A
Non-SCC-0539	2	51	0	0	0	Non-SCC	C/T	G/G	A/A
Non-SCC-0540	2	45	0	0	0	Non-SCC	T/T	G/C	A/A
Non-SCC-0541	1	53	1	1	0	Non-SCC	C/T	G/C	A/A
Non-SCC-0542	2	57	0	0	0	Non-SCC	T/T	C/C	G/A
Non-SCC-0543	1	37	1	0	0	Non-SCC	T/T	G/C	A/A
Non-SCC-0544	1	70	0	0	0	Non-SCC	C/T	C/C	G/A
Non-SCC-0545	1	54	0	1	0	Non-SCC	T/T	G/C	A/A
Non-SCC-0546	2	64	0	0	0	Non-SCC	C/T	C/C	A/A
Non-SCC-0547	1	53	1	1	1	Non-SCC	T/T	C/C	G/A
Non-SCC-0548	2	55	0	0	0	Non-SCC	?	?	?
Non-SCC-0549	2	59	0	0	1	Non-SCC	T/T	G/C	A/A
Non-SCC-0550	2	71	0	0	0	Non-SCC	C/C	G/C	G/A
Non-SCC-0551	1	62	0	0	0	Non-SCC	T/T	G/C	G/G
Non-SCC-0552	1	44	0	0	1	Non-SCC	T/T	G/C	A/A
Non-SCC-0553	1	57	0	0	1	Non-SCC	?	?	?
Non-SCC-0554	1	61	0	0	1	Non-SCC	C/C	C/C	A/A
Non-SCC-0555	1	61	0	0	0	Non-SCC	C/T	G/C	A/A
Non-SCC-0556	2	54	0	0	0	Non-SCC	C/T	G/G	A/A
Non-SCC-0557	1	71	1	1	0	Non-SCC	C/C	G/C	A/A
Non-SCC-0558	2	62	0	0	0	Non-SCC	T/T	C/C	A/A
Non-SCC-0559	1	30	0	0	1	Non-SCC	T/T	G/C	A/A
Non-SCC-0560	2	61	0	0	0	Non-SCC	T/T	C/C	A/A
Non-SCC-0561	2	51	0	0	0	Non-SCC	T/T	C/C	A/A
Non-SCC-0562	2	65	0	0	0	Non-SCC	C/C	G/C	A/A
Non-SCC-0563	2	72	0	0	0	Non-SCC	C/T	G/C	A/A
Non-SCC-0564	2	71	0	0	1	Non-SCC	C/T	C/C	A/A
Non-SCC-0565	2	66	0	0	0	Non-SCC	T/T	G/C	A/A
Non-SCC-0566	1	60	1	1	1	Non-SCC	C/C	C/C	G/A
Non-SCC-0567	1	68	1	1	0	Non-SCC	C/T	C/C	G/A
Non-SCC-0568	1	65	1	0	0	Non-SCC	C/T	C/C	G/A
Non-SCC-0569	1	51	0	1	1	Non-SCC	C/C	C/C	A/A
Non-SCC-0570	2	45	0	0	0	Non-SCC	T/T	C/C	A/A
Non-SCC-0571	1	66	0	1	0	Non-SCC	T/T	G/C	G/A
Non-SCC-0572	2	49	0	0	0	Non-SCC	T/T	G/C	A/A
Non-SCC-0573	2	59	0	0	0	Non-SCC	C/T	C/C	A/A
Non-SCC-0574	2	59	0	0	0	Non-SCC	T/T	G/C	G/A
Non-SCC-0575	1	71	1	0	1	Non-SCC	C/C	G/G	A/A
Non-SCC-0576	2	79	0	0	0	Non-SCC	C/C	G/C	A/A
Non-SCC-0577	1	80	0	0	0	Non-SCC	C/T	C/C	A/A
Non-SCC-0578	1	64	1	1	1	Non-SCC	C/C	G/C	G/A
Non-SCC-0579	1	67	1	0	0	Non-SCC	C/C	C/C	G/A
Non-SCC-0580	2	69	0	0	1	Non-SCC	C/C	C/C	G/A
Non-SCC-0581	2	60	0	0	0	Non-SCC	T/T	G/C	G/A
Non-SCC-0582	2	46	0	0	0	Non-SCC	C/T	G/C	A/A
Non-SCC-0583	2	59	0	0	0	Non-SCC	C/T	C/C	A/A
Non-SCC-0584	1	59	1	0	0	Non-SCC	C/C	C/C	A/A
Non-SCC-0585	2	63	0	0	0	Non-SCC	T/T	G/C	G/A
Non-SCC-0586	1	42	0	0	0	Non-SCC	C/T	G/C	G/A
Non-SCC-0587	2	52	0	0	1	Non-SCC	C/T	G/C	A/A
Non-SCC-0588	1	47	0	0	1	Non-SCC	T/T	C/C	A/A
Non-SCC-0589	2	70	0	0	1	Non-SCC	C/T	C/C	A/A
Non-SCC-0590	1	62	1	1	1	Non-SCC	C/T	C/C	G/A
Non-SCC-0591	2	45	0	0	0	Non-SCC	T/T	C/C	A/A
Non-SCC-0592	2	52	0	0	0	Non-SCC	T/T	G/C	G/A
Non-SCC-0593	2	72	0	0	1	Non-SCC	C/C	C/C	A/A
Non-SCC-0594	2	57	0	0	0	Non-SCC	T/T	C/C	A/A
Non-SCC-0595	2	58	0	0	1	Non-SCC	T/T	G/C	G/A
Non-SCC-0596	1	58	1	1	0	Non-SCC	C/T	C/C	A/A
Non-SCC-0597	1	59	1	0	1	Non-SCC	C/T	G/G	G/A
Non-SCC-0598	2	61	0	0	1	Non-SCC	C/C	C/C	A/A
Non-SCC-0599	2	67	0	0	0	Non-SCC	C/T	C/C	A/A
Non-SCC-0600	2	35	0	0	0	Non-SCC	C/T	C/C	G/A
Non-SCC-0601	2	61	0	0	1	Non-SCC	T/T	C/C	G/A
Non-SCC-0602	2	48	0	0	0	Non-SCC	T/T	C/C	G/A
Non-SCC-0603	1	60	1	1	1	Non-SCC	T/T	C/C	G/A
Non-SCC-0604	2	53	0	0	1	Non-SCC	C/C	G/C	G/A
Non-SCC-0605	2	65	0	0	0	Non-SCC	T/T	C/C	A/A
Non-SCC-0606	2	45	0	0	0	Non-SCC	C/C	G/C	A/A
Non-SCC-0607	2	43	0	0	0	Non-SCC	C/T	G/C	A/A
Non-SCC-0608	2	66	0	0	0	Non-SCC	T/T	G/C	A/A
Non-SCC-0609	1	55	1	1	1	Non-SCC	T/T	G/G	A/A

Non-SCC-0610	2	45	0	0	0	Non-SCC	T/T	G/C	A/A
Non-SCC-0611	1	27	0	0	0	Non-SCC	C/T	G/C	A/A
Non-SCC-0612	2	32	0	0	0	Non-SCC	T/T	C/C	A/A
Non-SCC-0613	1	68	1	1	1	Non-SCC	T/T	G/G	A/A
Non-SCC-0614	2	61	0	0	0	Non-SCC	C/C	G/C	A/A
Non-SCC-0615	2	42	0	0	1	Non-SCC	T/T	C/C	A/A
Non-SCC-0616	1	67	1	1	1	Non-SCC	T/T	G/G	A/A
Non-SCC-0617	2	59	0	0	0	Non-SCC	T/T	G/G	G/A
Non-SCC-0618	2	57	0	0	0	Non-SCC	T/T	C/C	A/A
Non-SCC-0619	1	51	0	1	1	Non-SCC	C/T	C/C	A/A
Non-SCC-0620	2	40	0	0	0	Non-SCC	C/T	G/C	A/A
Non-SCC-0621	1	71	0	0	0	Non-SCC	C/T	C/C	A/A
Non-SCC-0622	2	50	0	0	0	Non-SCC	T/T	C/C	A/A
Non-SCC-0623	2	55	0	0	0	Non-SCC	C/T	G/C	G/A
Non-SCC-0624	2	40	0	0	0	Non-SCC	T/T	G/C	A/A
Non-SCC-0625	1	66	0	1	1	Non-SCC	C/C	C/C	A/A
Non-SCC-0626	2	37	0	0	0	Non-SCC	C/T	G/C	A/A
Non-SCC-0627	2	61	0	0	0	Non-SCC	C/T	G/C	A/A
Non-SCC-0628	2	44	0	0	0	Non-SCC	C/T	C/C	G/A
Non-SCC-0629	1	59	0	0	0	Non-SCC	T/T	G/C	A/A
Non-SCC-0630	1	75	1	1	0	Non-SCC	?	?	?
Non-SCC-0631	1	53	1	1	0	Non-SCC	T/T	G/C	A/A
Non-SCC-0632	2	64	0	0	0	Non-SCC	T/T	G/C	G/A
Non-SCC-0633	2	54	0	0	0	Non-SCC	T/T	C/C	G/A
Non-SCC-0634	2	53	0	0	0	Non-SCC	C/T	C/C	G/A
Non-SCC-0635	2	32	0	0	0	Non-SCC	C/T	C/C	A/A
Non-SCC-0636	2	53	0	0	0	Non-SCC	C/T	G/G	G/A
Non-SCC-0637	1	62	1	1	0	Non-SCC	T/T	C/C	A/A
Non-SCC-0638	1	70	0	0	0	Non-SCC	C/T	C/C	A/A
Non-SCC-0639	2	48	0	0	1	Non-SCC	C/T	C/C	A/A
Non-SCC-0640	2	59	0	0	0	Non-SCC	T/T	G/C	G/A
Non-SCC-0641	2	54	0	0	0	Non-SCC	C/C	G/C	A/A
Non-SCC-0642	1	59	1	1	0	Non-SCC	T/T	C/C	A/A
Non-SCC-0643	2	62	0	0	0	Non-SCC	C/C	G/C	A/A
Non-SCC-0644	2	65	0	0	0	Non-SCC	C/T	G/G	A/A
Non-SCC-0645	2	40	0	0	0	Non-SCC	T/T	C/C	A/A
Non-SCC-0646	2	45	0	0	0	Non-SCC	C/C	G/C	A/A
Non-SCC-0647	1	64	1	0	0	Non-SCC	T/T	G/C	A/A
Non-SCC-0648	1	70	1	0	0	Non-SCC	C/T	G/C	G/A
Non-SCC-0649	1	52	1	0	0	Non-SCC	T/T	G/G	A/A
Non-SCC-0650	1	48	1	0	1	Non-SCC	T/T	G/C	A/A
Non-SCC-0651	1	64	1	0	1	Non-SCC	C/C	C/C	A/A
Non-SCC-0652	1	43	0	1	0	Non-SCC	C/T	C/C	A/A
Non-SCC-0653	1	61	1	0	0	Non-SCC	T/T	G/G	A/A
Non-SCC-0654	2	54	0	0	0	Non-SCC	T/T	C/C	A/A
Non-SCC-0655	1	69	1	1	1	Non-SCC	T/T	G/C	G/A
Non-SCC-0656	2	60	0	0	0	Non-SCC	C/T	G/C	G/A
Non-SCC-0657	2	54	0	0	0	Non-SCC	C/T	G/C	A/A
Non-SCC-0658	2	65	0	0	1	Non-SCC	C/C	G/G	A/A
Non-SCC-0659	2	63	1	1	0	Non-SCC	C/T	G/C	A/A
Non-SCC-0660	2	50	0	0	0	Non-SCC	C/T	G/C	?
Non-SCC-0661	1	61	0	0	0	Non-SCC	C/T	G/C	A/A
Non-SCC-0662	2	49	0	0	0	Non-SCC	C/T	G/C	A/A
Non-SCC-0663	2	52	0	0	0	Non-SCC	T/T	G/C	A/A
Non-SCC-0664	2	44	0	0	0	Non-SCC	C/T	C/C	A/A
Non-SCC-0665	2	60	0	0	0	Non-SCC	C/T	G/C	G/A
Non-SCC-0666	2	71	0	0	1	Non-SCC	C/T	C/C	G/A
Non-SCC-0667	1	54	0	0	0	Non-SCC	C/T	G/C	G/A
Non-SCC-0668	2	54	0	0	0	Non-SCC	C/T	C/C	A/A
Non-SCC-0669	1	55	1	1	1	Non-SCC	C/T	G/C	G/A
Non-SCC-0670	1	72	1	1	0	Non-SCC	C/T	C/C	A/A
Non-SCC-0671	1	45	0	0	0	Non-SCC	C/T	G/G	A/A
Non-SCC-0672	1	60	0	0	1	Non-SCC	T/T	C/C	G/G
Non-SCC-0673	2	28	0	0	0	Non-SCC	C/C	C/C	G/A
Non-SCC-0674	2	73	0	0	0	Non-SCC	C/C	G/G	G/A
Non-SCC-0675	2	65	0	0	0	Non-SCC	T/T	C/C	A/A
Non-SCC-0676	1	58	1	1	0	Non-SCC	C/T	G/C	A/A
Non-SCC-0677	2	70	0	0	0	Non-SCC	T/T	C/C	G/A
Non-SCC-0678	1	51	1	1	0	Non-SCC	C/T	C/C	G/A
Non-SCC-0679	2	62	0	0	0	Non-SCC	C/T	C/C	A/A
Non-SCC-0680	2	44	0	0	0	Non-SCC	T/T	G/C	G/A
Non-SCC-0681	1	58	1	0	1	Non-SCC	C/T	C/C	A/A
Non-SCC-0682	1	60	0	1	0	Non-SCC	C/T	C/C	A/A
Non-SCC-0683	2	66	0	0	1	Non-SCC	C/C	G/C	A/A
Non-SCC-0684	1	63	0	0	0	Non-SCC	T/T	C/C	A/A
Non-SCC-0685	2	65	0	0	0	Non-SCC	C/T	G/C	A/A
Non-SCC-0686	2	72	0	0	0	Non-SCC	T/T	G/C	G/G

Non-SCC-0687	1	60	0	0	0	Non-SCC	C/C	G/C	A/A
Non-SCC-0688	2	47	0	0	1	Non-SCC	C/T	G/C	A/A
Non-SCC-0689	1	73	0	1	0	Non-SCC	C/T	G/G	A/A
Non-SCC-0690	1	70	1	0	0	Non-SCC	C/T	C/C	G/A
Non-SCC-0691	2	63	0	0	0	Non-SCC	C/T	C/C	G/A
Non-SCC-0692	1	40	0	1	1	Non-SCC	T/T	G/C	G/A
Non-SCC-0693	2	46	0	0	0	Non-SCC	C/C	G/C	A/A
Non-SCC-0694	1	56	1	1	0	Non-SCC	T/T	C/C	A/A
Non-SCC-0695	1	53	1	1	1	Non-SCC	C/T	C/C	A/A
Non-SCC-0696	1	76	1	1	0	Non-SCC	T/T	C/C	A/A
Non-SCC-0697	1	29	0	0	1	Non-SCC	T/T	C/C	A/A
Non-SCC-0698	1	50	1	0	0	Non-SCC	C/T	G/C	A/A
Non-SCC-0699	2	54	0	0	0	Non-SCC	C/T	G/C	A/A
Non-SCC-0700	1	57	0	1	0	Non-SCC	C/T	C/C	A/A
Non-SCC-0701	1	38	1	1	1	Non-SCC	C/T	G/C	A/A
Non-SCC-0702	1	30	0	0	0	Non-SCC	C/T	C/C	G/G
Non-SCC-0703	2	51	0	0	1	Non-SCC	C/T	G/C	A/A
Non-SCC-0704	2	49	0	0	0	Non-SCC	C/C	G/C	G/A
Non-SCC-0705	1	66	0	1	1	Non-SCC	C/T	C/C	G/A
Non-SCC-0706	1	64	1	0	0	Non-SCC	C/C	C/C	G/A
Non-SCC-0707	2	45	0	0	0	Non-SCC	C/T	G/C	A/A
Non-SCC-0708	2	54	0	0	1	Non-SCC	T/T	C/C	A/A
Non-SCC-0709	2	50	0	0	0	Non-SCC	T/T	C/C	G/A
Non-SCC-0710	2	40	0	0	0	Non-SCC	T/T	G/C	A/A
Non-SCC-0711	1	71	1	0	0	Non-SCC	C/T	C/C	A/A
Non-SCC-0712	1	57	1	1	1	Non-SCC	C/C	C/C	G/A
Non-SCC-0713	2	65	0	0	0	Non-SCC	C/T	G/C	A/A
Non-SCC-0714	2	62	0	0	1	Non-SCC	C/C	C/C	A/A
Non-SCC-0715	2	68	0	0	0	Non-SCC	T/T	G/G	G/A
Non-SCC-0716	1	48	0	0	1	Non-SCC	C/C	G/G	G/A
Non-SCC-0717	1	60	1	0	1	Non-SCC	C/T	G/C	G/A
Non-SCC-0718	1	57	0	1	1	Non-SCC	T/T	G/C	A/A
Non-SCC-0719	1	59	0	1	1	Non-SCC	C/T	G/G	A/A
Non-SCC-0720	2	55	0	0	0	Non-SCC	C/C	G/C	A/A
Non-SCC-0721	2	46	0	0	1	Non-SCC	C/T	G/C	G/A
Non-SCC-0722	1	59	1	1	0	Non-SCC	C/C	G/C	A/A
Non-SCC-0723	1	51	1	1	1	Non-SCC	C/T	G/G	A/A
Non-SCC-0724	1	51	1	1	0	Non-SCC	C/C	G/G	G/A
Non-SCC-0725	2	67	0	1	0	Non-SCC	C/C	G/G	G/A
Non-SCC-0726	2	56	0	0	0	Non-SCC	T/T	G/C	A/A
Non-SCC-0727	2	60	0	0	0	Non-SCC	C/T	C/C	A/A
Non-SCC-0728	2	68	0	0	0	Non-SCC	C/T	G/C	A/A
Non-SCC-0729	2	51	0	0	0	Non-SCC	C/C	G/G	G/A
Non-SCC-0730	2	58	0	0	0	Non-SCC	T/T	C/C	A/A
Non-SCC-0731	2	53	0	0	1	Non-SCC	T/T	G/C	A/A
Non-SCC-0732	2	53	0	0	1	Non-SCC	T/T	G/C	A/A
Non-SCC-0733	2	58	0	0	0	Non-SCC	C/C	G/C	G/A
Non-SCC-0734	1	66	1	1	0	Non-SCC	T/T	G/C	A/A
Non-SCC-0735	1	78	0	0	0	Non-SCC	C/T	C/C	A/A
Non-SCC-0736	1	54	1	1	1	Non-SCC	T/T	G/C	A/A
Non-SCC-0737	1	38	0	0	1	Non-SCC	T/T	C/C	A/A
Non-SCC-0738	2	67	0	0	1	Non-SCC	C/T	C/C	G/A
Non-SCC-0739	2	51	0	0	1	Non-SCC	C/C	C/C	A/A
Non-SCC-0740	2	46	0	0	1	Non-SCC	C/T	G/C	G/A
Non-SCC-0741	1	27	0	0	1	Non-SCC	C/T	G/C	A/A
Non-SCC-0742	2	51	0	0	0	Non-SCC	C/C	G/G	A/A
Non-SCC-0743	1	28	1	1	0	Non-SCC	T/T	G/C	A/A
Non-SCC-0744	1	62	1	1	1	Non-SCC	C/T	G/G	A/A
Non-SCC-0745	2	59	0	0	0	Non-SCC	C/T	G/C	A/A
Non-SCC-0746	1	40	0	1	0	Non-SCC	C/T	G/G	A/A
Non-SCC-0747	2	40	0	0	0	Non-SCC	T/T	G/G	A/A
Non-SCC-0748	2	49	0	0	0	Non-SCC	C/T	G/C	G/A
Non-SCC-0749	1	69	1	1	0	Non-SCC	T/T	G/C	A/A
Non-SCC-0750	2	53	0	0	0	Non-SCC	C/T	C/C	A/A
Non-SCC-0751	2	51	0	0	0	Non-SCC	C/T	G/C	A/A
Non-SCC-0752	2	48	0	0	1	Non-SCC	C/T	G/C	A/A
Non-SCC-0753	1	64	1	0	1	Non-SCC	T/T	G/C	A/A
Non-SCC-0754	1	54	1	1	0	Non-SCC	C/T	G/C	A/A
Non-SCC-0755	1	61	0	0	0	Non-SCC	C/C	G/C	A/A
Non-SCC-0756	2	43	0	0	0	Non-SCC	T/T	G/C	A/A
Non-SCC-0757	1	43	1	1	1	Non-SCC	C/T	G/C	A/A
Non-SCC-0758	2	63	0	0	0	Non-SCC	C/T	G/C	G/A
Non-SCC-0759	1	65	1	1	0	Non-SCC	C/T	C/C	G/G
Non-SCC-0760	2	62	0	0	0	Non-SCC	C/T	C/C	A/A
Non-SCC-0761	1	82	1	0	0	Non-SCC	T/T	C/C	A/A
Non-SCC-0762	2	50	0	0	0	Non-SCC	C/T	G/C	G/A
Non-SCC-0763	2	68	0	0	0	Non-SCC	T/T	C/C	A/A

Non-SCC-0764	2	53	0	0	0	Non-SCC	C/C	G/G	A/A
Non-SCC-0765	2	52	0	0	0	Non-SCC	T/T	C/C	G/A
Non-SCC-0766	2	60	0	0	0	Non-SCC	C/T	C/C	A/A
Non-SCC-0767	1	48	1	1	0	Non-SCC	T/T	C/C	G/A
Non-SCC-0768	2	60	0	0	1	Non-SCC	C/T	G/C	G/A
Non-SCC-0769	1	47	0	0	0	Non-SCC	T/T	C/C	A/A
Non-SCC-0770	1	52	1	1	1	Non-SCC	C/T	G/G	A/A
Non-SCC-0771	1	66	1	1	0	Non-SCC	C/C	G/G	G/A
Non-SCC-0772	1	79	1	1	1	Non-SCC	C/T	G/G	A/A
Non-SCC-0773	1	63	1	0	1	Non-SCC	T/T	C/C	A/A
Non-SCC-0774	1	58	1	0	0	Non-SCC	C/T	G/C	A/A
Non-SCC-0775	2	66	0	0	0	Non-SCC	T/T	G/C	A/A
Non-SCC-0776	1	55	1	0	0	Non-SCC	C/T	G/C	G/G
Non-SCC-0777	2	71	0	0	0	Non-SCC	T/T	G/C	A/A
Non-SCC-0778	2	55	0	0	0	Non-SCC	C/C	C/C	A/A
Non-SCC-0779	2	57	0	0	0	Non-SCC	C/C	G/C	A/A
Non-SCC-0780	1	55	1	0	0	Non-SCC	T/T	C/C	A/A
Non-SCC-0781	2	62	0	0	0	Non-SCC	C/C	G/C	A/A
Non-SCC-0782	1	59	1	1	0	Non-SCC	C/T	G/C	G/G
Non-SCC-0783	2	59	0	0	0	Non-SCC	C/T	G/G	A/A
Non-SCC-0784	1	62	1	1	0	Non-SCC	C/T	G/C	A/A
Non-SCC-0785	1	53	1	1	0	Non-SCC	C/T	G/C	A/A
Non-SCC-0786	1	66	1	0	1	Non-SCC	C/T	G/G	A/A
Non-SCC-0787	2	63	0	0	0	Non-SCC	T/T	G/G	A/A
Non-SCC-0788	2	56	0	0	0	Non-SCC	T/T	G/C	A/A
Non-SCC-0789	1	70	0	0	0	Non-SCC	C/T	G/C	G/A
Non-SCC-0790	1	52	0	0	1	Non-SCC	C/T	C/C	A/A
Non-SCC-0791	2	64	1	1	0	Non-SCC	C/T	G/G	A/A
Non-SCC-0792	2	55	0	0	0	Non-SCC	C/T	G/C	G/A
Non-SCC-0793	1	53	1	1	1	Non-SCC	T/T	G/C	A/A
Non-SCC-0794	2	54	0	0	0	Non-SCC	C/T	C/C	A/A
Non-SCC-0795	1	51	1	1	0	Non-SCC	C/C	C/C	G/A
Non-SCC-0796	1	55	1	1	1	Non-SCC	C/T	C/C	G/A
Non-SCC-0797	1	64	1	1	1	Non-SCC	C/T	G/C	A/A
Non-SCC-0798	1	52	1	1	1	Non-SCC	C/T	G/C	A/A
Non-SCC-0799	1	52	0	0	0	Non-SCC	T/T	G/G	A/A
Non-SCC-0800	1	59	0	0	0	Non-SCC	C/T	G/C	A/A
Non-SCC-0801	2	76	0	0	0	Non-SCC	T/T	G/C	A/A
Non-SCC-0802	1	70	0	0	1	Non-SCC	C/T	G/C	G/G
Non-SCC-0803	2	51	0	0	1	Non-SCC	C/T	G/C	G/A
Non-SCC-0804	2	59	0	0	0	Non-SCC	C/T	C/C	A/A
Non-SCC-0805	2	55	0	0	1	Non-SCC	C/T	G/C	A/A
Non-SCC-0806	1	56	0	0	1	Non-SCC	T/T	C/C	G/A
Non-SCC-0807	1	59	1	1	0	Non-SCC	C/T	C/C	A/A
Non-SCC-0808	1	52	1	1	0	Non-SCC	C/T	C/C	A/A
Non-SCC-0809	2	52	0	0	1	Non-SCC	C/T	C/C	A/A
Non-SCC-0810	2	54	0	0	0	Non-SCC	T/T	G/C	G/A
Non-SCC-0811	1	39	1	0	0	Non-SCC	C/T	C/C	A/A
Non-SCC-0812	2	55	0	0	0	Non-SCC	C/T	G/G	G/A
Non-SCC-0813	1	52	1	1	0	Non-SCC	T/T	G/G	A/A
Non-SCC-0814	2	54	0	0	1	Non-SCC	C/T	G/C	A/A
Non-SCC-0815	2	75	0	0	1	Non-SCC	T/T	C/C	A/A
Non-SCC-0816	2	71	0	0	0	Non-SCC	T/T	C/C	A/A
Non-SCC-0817	2	56	0	0	0	Non-SCC	C/C	G/C	G/A
Non-SCC-0818	2	71	0	0	1	Non-SCC	C/T	C/C	A/A
Non-SCC-0819	1	69	1	0	0	Non-SCC	C/C	G/C	A/A
Non-SCC-0820	2	71	0	0	0	Non-SCC	C/C	G/C	A/A
Non-SCC-0821	2	60	0	0	0	Non-SCC	C/T	C/C	A/A
Non-SCC-0822	2	53	0	0	1	Non-SCC	T/T	G/C	A/A
Non-SCC-0823	2	57	0	0	0	Non-SCC	C/C	G/C	G/A
Non-SCC-0824	1	69	1	1	1	Non-SCC	T/T	G/G	A/A
Non-SCC-0825	2	67	0	0	0	Non-SCC	T/T	G/G	A/A
Non-SCC-0826	2	53	0	0	0	Non-SCC	C/C	C/C	A/A
Non-SCC-0827	2	52	0	0	0	Non-SCC	C/T	G/G	A/A
Non-SCC-0828	2	62	0	0	1	Non-SCC	C/T	G/C	A/A
Non-SCC-0829	2	65	0	0	0	Non-SCC	C/T	G/C	A/A
Non-SCC-0830	1	49	1	1	0	Non-SCC	T/T	C/C	A/A
Non-SCC-0831	2	59	0	0	1	Non-SCC	C/T	C/C	G/A
Non-SCC-0832	2	46	0	0	0	Non-SCC	C/T	G/C	A/A
Non-SCC-0833	2	67	0	0	0	Non-SCC	T/T	G/G	A/A
Non-SCC-0834	1	55	0	0	0	Non-SCC	C/T	C/C	A/A
Non-SCC-0835	1	76	0	0	0	Non-SCC	T/T	C/C	G/A
Non-SCC-0836	1	60	0	0	0	Non-SCC	T/T	G/C	G/A
Non-SCC-0837	1	67	1	1	0	Non-SCC	C/C	G/G	A/A
Non-SCC-0838	2	63	0	0	0	Non-SCC	C/T	G/C	G/A
Non-SCC-0839	1	60	0	1	1	Non-SCC	C/C	C/C	G/A
Non-SCC-0840	1	59	0	0	1	Non-SCC	C/T	C/C	G/A

Non-SCC-0841	2	57	0	0	0	Non-SCC	C/T	G/C	A/A
Non-SCC-0842	2	56	0	0	1	Non-SCC	C/C	G/C	A/A
Non-SCC-0843	1	60	0	0	0	Non-SCC	C/T	C/C	A/A
Non-SCC-0844	2	72	0	0	0	Non-SCC	T/T	C/C	A/A
Non-SCC-0845	2	73	0	0	1	Non-SCC	T/T	G/C	A/A
Non-SCC-0846	2	59	0	0	1	Non-SCC	C/T	G/C	G/A
Non-SCC-0847	2	54	0	0	1	Non-SCC	C/T	C/C	A/A
Non-SCC-0848	1	62	1	1	0	Non-SCC	C/C	G/C	G/A
Non-SCC-0849	2	62	0	0	0	Non-SCC	T/T	G/G	A/A
Non-SCC-0850	1	75	0	0	1	Non-SCC	T/T	C/C	A/A
Non-SCC-0851	2	72	0	0	0	Non-SCC	C/C	G/C	A/A
Non-SCC-0852	1	46	1	1	1	Non-SCC	C/C	G/G	G/A
Non-SCC-0853	1	58	1	0	1	Non-SCC	C/T	G/G	G/A
Non-SCC-0854	2	66	0	0	1	Non-SCC	C/T	C/C	A/A
Non-SCC-0855	2	64	0	0	0	Non-SCC	T/T	G/G	A/A
Non-SCC-0856	2	53	0	0	1	Non-SCC	C/C	G/C	A/A
Non-SCC-0857	1	62	0	0	1	Non-SCC	C/T	G/C	G/A
Non-SCC-0858	1	63	0	0	1	Non-SCC	C/T	G/C	G/G
Non-SCC-0859	2	60	0	0	1	Non-SCC	C/T	C/C	A/A
Non-SCC-0860	1	57	0	0	0	Non-SCC	C/T	G/G	G/G
Non-SCC-0861	2	67	0	0	1	Non-SCC	T/T	G/C	A/A
Non-SCC-0862	2	54	0	0	1	Non-SCC	C/C	G/C	G/A
Non-SCC-0863	2	79	0	0	0	Non-SCC	C/T	G/G	G/A
Non-SCC-0864	1	63	1	0	1	Non-SCC	C/T	G/C	A/A
Non-SCC-0865	2	69	0	0	1	Non-SCC	C/T	G/G	A/A
Non-SCC-0866	1	69	1	1	0	Non-SCC	C/T	C/C	G/A
Non-SCC-0867	1	45	0	0	0	Non-SCC	C/C	G/C	A/A
Non-SCC-0868	2	69	0	0	1	Non-SCC	T/T	C/C	A/A
Non-SCC-0869	1	62	1	0	0	Non-SCC	C/C	G/C	A/A
Non-SCC-0870	1	55	1	0	0	Non-SCC	C/C	C/C	A/A
Non-SCC-0871	2	55	0	0	1	Non-SCC	C/T	C/C	G/A
Non-SCC-0872	2	50	0	0	1	Non-SCC	T/T	C/C	A/A
Non-SCC-0873	2	65	0	0	0	Non-SCC	C/T	C/C	A/A
Non-SCC-0874	2	62	0	0	0	Non-SCC	C/T	C/C	A/A
Non-SCC-0875	1	67	1	0	1	Non-SCC	T/T	C/C	G/A
Non-SCC-0876	2	75	0	0	1	Non-SCC	C/T	C/C	A/A
Non-SCC-0877	1	63	0	0	1	Non-SCC	C/T	G/C	A/A
Non-SCC-0878	2	66	0	0	0	Non-SCC	C/C	C/C	A/A
Non-SCC-0879	1	66	1	1	0	Non-SCC	C/T	C/C	A/A
Non-SCC-0880	2	54	0	0	0	Non-SCC	C/C	G/C	A/A
Non-SCC-0881	1	60	0	0	0	Non-SCC	T/T	G/C	A/A
Non-SCC-0882	1	75	1	1	0	Non-SCC	C/C	G/G	A/A
Non-SCC-0883	1	49	0	0	1	Non-SCC	T/T	G/G	A/A
Non-SCC-0884	2	60	0	0	1	Non-SCC	C/T	G/C	A/A
Non-SCC-0885	1	62	0	0	0	Non-SCC	C/C	G/C	A/A
Non-SCC-0886	2	53	0	0	0	Non-SCC	C/T	C/C	A/A
Non-SCC-0887	2	58	0	0	0	Non-SCC	C/T	C/C	G/A
Non-SCC-0888	2	58	0	0	0	Non-SCC	C/T	G/C	G/A
Non-SCC-0889	2	64	0	0	0	Non-SCC	C/T	G/G	G/G
Non-SCC-0890	2	54	0	0	1	Non-SCC	C/C	G/C	G/A
Non-SCC-0891	1	72	0	0	0	Non-SCC	C/C	G/G	A/A
Non-SCC-0892	1	62	0	0	0	Non-SCC	C/T	G/G	A/A
Non-SCC-0893	1	64	0	0	1	Non-SCC	C/T	G/G	A/A
Non-SCC-0894	2	64	0	0	0	Non-SCC	C/T	G/G	G/A
Non-SCC-0895	1	65	0	0	0	Non-SCC	C/T	G/G	A/A
Non-SCC-0896	1	66	1	0	0	Non-SCC	C/C	G/C	G/A
Non-SCC-0897	2	74	0	0	0	Non-SCC	C/T	G/C	G/A
Non-SCC-0898	1	68	0	0	0	Non-SCC	C/C	C/C	A/A
Non-SCC-0899	2	49	0	0	0	Non-SCC	T/T	G/C	A/A
Non-SCC-0900	1	69	1	0	0	Non-SCC	C/C	G/G	A/A
Non-SCC-0901	1	50	1	0	1	Non-SCC	C/T	G/G	G/A
Non-SCC-0902	1	66	0	0	1	Non-SCC	C/T	G/G	A/A
Non-SCC-0903	1	53	1	0	0	Non-SCC	T/T	G/C	A/A
Non-SCC-0904	1	69	0	0	0	Non-SCC	C/T	G/C	G/A
Non-SCC-0905	2	60	0	0	1	Non-SCC	C/T	C/C	A/A
Non-SCC-0906	1	63	1	1	1	Non-SCC	C/T	C/C	A/A
Non-SCC-0907	1	58	1	1	0	Non-SCC	C/T	G/C	A/A
Non-SCC-0908	1	69	0	0	1	Non-SCC	C/T	G/G	A/A
Non-SCC-0909	2	70	0	0	0	Non-SCC	C/C	G/C	G/G
Non-SCC-0910	2	46	0	0	1	Non-SCC	T/T	C/C	G/A
Non-SCC-0911	2	49	0	0	0	Non-SCC	C/T	G/C	A/A
Non-SCC-0912	2	59	0	0	0	Non-SCC	C/T	C/C	G/A
Non-SCC-0913	1	63	0	0	1	Non-SCC	C/T	C/C	G/A
Non-SCC-0914	1	67	1	1	0	Non-SCC	C/C	C/C	G/A
Non-SCC-0915	1	58	0	0	1	Non-SCC	C/C	G/C	A/A
Non-SCC-0916	2	63	0	0	1	Non-SCC	T/T	G/C	A/A
Non-SCC-0917	1	37	1	1	0	Non-SCC	T/T	C/C	G/A

Non-SCC-0918	1	50	1	1	0	Non-SCC	C/C	C/C	A/A
Non-SCC-0919	2	44	0	0	0	Non-SCC	C/C	G/C	A/A
Non-SCC-0920	2	55	0	0	0	Non-SCC	C/C	G/G	A/A
Non-SCC-0921	1	57	1	1	0	Non-SCC	C/T	G/C	A/A
Non-SCC-0922	1	51	1	0	1	Non-SCC	C/T	G/C	A/A
Non-SCC-0923	1	53	0	0	1	Non-SCC	C/T	G/C	A/A
Non-SCC-0924	1	60	1	0	0	Non-SCC	C/T	G/G	A/A
Non-SCC-0925	2	37	0	0	0	Non-SCC	T/T	G/G	G/G
Non-SCC-0926	2	66	0	0	0	Non-SCC	T/T	G/G	A/A
Non-SCC-0927	1	66	0	0	0	Non-SCC	C/T	C/C	G/A
Non-SCC-0928	1	55	0	0	0	Non-SCC	C/T	C/C	G/A
Non-SCC-0929	2	52	0	0	0	Non-SCC	C/C	G/G	G/A
Non-SCC-0930	1	52	1	1	0	Non-SCC	T/T	C/C	A/A
Non-SCC-0931	2	46	0	0	0	Non-SCC	T/T	G/G	G/A
Non-SCC-0932	2	77	0	0	1	Non-SCC	C/T	G/G	A/A
Non-SCC-0933	2	47	0	0	0	Non-SCC	C/C	C/C	G/A
Non-SCC-0934	2	51	0	0	0	Non-SCC	C/T	G/C	A/A
Non-SCC-0935	1	42	0	0	0	Non-SCC	C/C	G/C	A/A
Non-SCC-0936	2	55	0	0	1	Non-SCC	T/T	C/C	A/A
Non-SCC-0937	2	65	0	0	0	Non-SCC	C/T	G/C	A/A
Non-SCC-0938	1	49	0	0	0	Non-SCC	T/T	G/C	A/A
Non-SCC-0939	2	48	0	0	0	Non-SCC	C/T	G/G	A/A
Non-SCC-0940	1	77	0	0	0	Non-SCC	C/C	G/C	G/A
Non-SCC-0941	1	76	1	1	1	Non-SCC	C/T	C/C	A/A
Non-SCC-0942	1	57	0	1	1	Non-SCC	T/T	C/C	A/A
Non-SCC-0943	1	63	1	1	1	Non-SCC	C/T	G/G	A/A
Non-SCC-0944	1	53	0	0	1	Non-SCC	C/T	G/C	A/A
Non-SCC-0945	1	51	1	0	1	Non-SCC	C/T	G/C	A/A
Non-SCC-0946	2	48	0	0	0	Non-SCC	T/T	G/C	A/A
Non-SCC-0947	1	70	1	0	0	Non-SCC	C/T	G/C	A/A
Non-SCC-0948	2	44	0	0	0	Non-SCC	C/C	G/G	A/A
Non-SCC-0949	1	70	0	0	0	Non-SCC	C/T	C/C	A/A
Non-SCC-0950	2	58	0	0	0	Non-SCC	C/C	G/C	G/A
Non-SCC-0951	1	55	0	0	0	Non-SCC	C/T	C/C	G/A
Non-SCC-0952	2	61	0	0	0	Non-SCC	C/T	G/C	G/A
Non-SCC-0953	1	48	1	1	1	Non-SCC	C/C	C/C	G/A
Non-SCC-0954	1	43	0	0	0	Non-SCC	T/T	G/G	?
Non-SCC-0955	1	59	1	0	0	Non-SCC	T/T	G/C	A/A
Non-SCC-0956	1	46	1	1	1	Non-SCC	C/C	G/C	A/A
Non-SCC-0957	2	61	0	0	0	Non-SCC	C/C	G/C	G/A
Non-SCC-0958	1	68	1	0	1	Non-SCC	C/T	C/C	A/A
Non-SCC-0959	1	60	1	1	1	Non-SCC	T/T	G/C	A/A
Non-SCC-0960	2	68	0	0	1	Non-SCC	T/T	G/G	A/A
Non-SCC-0961	2	65	0	0	0	Non-SCC	C/T	C/C	G/G
Non-SCC-0962	2	62	0	0	1	Non-SCC	C/T	G/G	G/A
Non-SCC-0963	1	70	1	1	0	Non-SCC	?	?	?
Non-SCC-0964	1	70	1	0	0	Non-SCC	C/T	G/C	A/A
Non-SCC-0965	2	39	0	0	0	Non-SCC	C/T	C/C	G/A
Non-SCC-0966	1	57	0	0	1	Non-SCC	T/T	C/C	A/A
Non-SCC-0967	2	68	0	0	1	Non-SCC	C/C	C/C	A/A
Non-SCC-0968	2	57	0	0	0	Non-SCC	C/T	C/C	A/A
Non-SCC-0969	1	64	1	1	0	Non-SCC	T/T	G/C	A/A
Non-SCC-0970	1	65	1	1	0	Non-SCC	T/T	C/C	A/A
Non-SCC-0971	2	45	0	0	0	Non-SCC	C/T	G/C	?
Non-SCC-0972	2	33	0	0	0	Non-SCC	C/T	C/C	G/A
Non-SCC-0973	1	29	0	0	0	Non-SCC	C/T	C/C	A/A
Non-SCC-0974	2	66	0	0	0	Non-SCC	C/T	C/C	A/A
Non-SCC-0975	2	82	0	0	0	Non-SCC	?	?	?
Non-SCC-0976	1	62	0	0	1	Non-SCC	C/T	C/C	A/A
Non-SCC-0977	2	50	0	0	0	Non-SCC	?	?	?
Non-SCC-0978	1	51	1	0	0	Non-SCC	C/C	G/C	G/G
Non-SCC-0979	2	55	0	0	0	Non-SCC	C/T	C/C	G/A
Non-SCC-0980	1	51	1	1	1	Non-SCC	C/T	C/C	A/A
Non-SCC-0981	2	66	0	0	1	Non-SCC	C/T	G/C	A/A
Non-SCC-0982	2	48	0	0	0	Non-SCC	T/T	G/G	A/A
Non-SCC-0983	2	71	0	0	1	Non-SCC	T/T	C/C	A/A
Non-SCC-0984	2	47	0	0	0	Non-SCC	C/T	C/C	A/A
Non-SCC-0985	1	51	1	0	1	Non-SCC	C/T	C/C	A/A
Non-SCC-0986	2	45	0	0	1	Non-SCC	C/T	C/C	A/A
Non-SCC-0987	1	57	0	0	0	Non-SCC	C/T	G/C	A/A
Non-SCC-0988	2	54	0	0	0	Non-SCC	C/T	C/C	A/A
Non-SCC-0989	2	61	0	0	0	Non-SCC	T/T	G/C	G/A
Non-SCC-0990	1	73	1	1	0	Non-SCC	C/T	C/C	G/A
Non-SCC-0991	1	29	0	0	0	Non-SCC	T/T	G/C	A/A
Non-SCC-0992	2	62	0	0	0	Non-SCC	C/T	C/C	A/A
Non-SCC-0993	2	29	0	0	0	Non-SCC	T/T	G/C	A/A
Non-SCC-0994	2	54	0	0	1	Non-SCC	C/C	G/C	G/A

Non-SCC-0995	2	73	0	0	0	Non-SCC	T/T	G/C	A/A
Non-SCC-0996	2	54	0	0	0	Non-SCC	T/T	G/C	G/G
Non-SCC-0997	1	53	1	0	0	Non-SCC	C/T	G/G	G/A
Non-SCC-0998	1	62	1	0	0	Non-SCC	T/T	C/C	A/A
Non-SCC-0999	2	67	0	0	0	Non-SCC	C/C	G/C	A/A
Non-SCC-1000	2	45	0	0	0	Non-SCC	T/T	C/C	A/A
Non-SCC-1001	1	65	1	1	0	Non-SCC	C/C	C/C	A/A
Non-SCC-1002	1	54	1	0	0	Non-SCC	C/T	G/C	G/A
Non-SCC-1003	1	51	1	1	0	Non-SCC	T/T	G/C	A/A
Non-SCC-1004	1	60	0	0	0	Non-SCC	C/T	C/C	G/G
Non-SCC-1005	1	52	0	0	0	Non-SCC	C/T	C/C	G/A
Non-SCC-1006	1	71	1	0	0	Non-SCC	C/T	C/C	G/A
Non-SCC-1007	1	64	1	1	1	Non-SCC	C/T	C/C	A/A
Non-SCC-1008	2	67	0	0	1	Non-SCC	C/T	G/C	A/A
Non-SCC-1009	2	63	0	0	0	Non-SCC	T/T	C/C	G/A
Non-SCC-1010	2	29	0	0	0	Non-SCC	T/T	G/C	A/A
Non-SCC-1011	2	56	0	0	0	Non-SCC	C/C	G/C	G/A
Control-0001	1	55	0	0	1		C/T	G/G	A/A
Control-0002	2	53	0	0	1		T/T	G/C	A/A
Control-0003	1	59	0	0	1		T/T	G/C	A/A
Control-0004	2	54	0	0	0		C/T	G/C	G/A
Control-0005	2	70	0	0	1		C/T	C/C	G/A
Control-0006	2	56	0	0	0		C/C	G/G	A/A
Control-0007	1	51	0	0	1		T/T	C/C	G/A
Control-0008	2	61	0	0	0		C/T	G/C	A/A
Control-0009	1	57	1	0	1		T/T	G/C	A/A
Control-0010	1	53	1	0	0		C/C	G/C	G/A
Control-0011	1	56	0	1	1		C/T	G/C	A/A
Control-0012	1	64	1	1	1		T/T	G/G	A/A
Control-0013	1	62	0	0	1		C/T	C/C	A/A
Control-0014	1	66	0	0	1		C/T	G/G	A/A
Control-0015	1	29	1	0	1		C/T	G/G	G/A
Control-0016	1	38	0	0	1		C/T	C/C	A/A
Control-0017	1	60	1	0	1		T/T	C/C	A/A
Control-0018	2	53	0	0	1		T/T	C/C	A/A
Control-0019	1	58	0	0	0		T/T	G/C	A/A
Control-0020	2	52	0	0	0		T/T	G/C	A/A
Control-0021	2	38	0	0	0		T/T	G/C	A/A
Control-0022	1	28	0	0	0		T/T	G/C	G/A
Control-0023	2	65	0	0	1		C/T	C/C	A/A
Control-0024	2	52	0	0	0		T/T	C/C	A/A
Control-0025	2	46	0	0	0		C/T	G/G	G/A
Control-0026	1	56	0	0	1		C/T	G/C	A/A
Control-0027	2	68	0	0	1		T/T	C/C	A/A
Control-0028	1	59	0	0	0		C/T	G/C	A/A
Control-0029	1	62	0	0	1		C/T	C/C	A/A
Control-0030	1	71	0	0	0		C/C	G/C	A/A
Control-0031	1	60	1	1	0		C/T	G/C	A/A
Control-0032	1	56	1	0	0		C/T	C/C	G/A
Control-0033	1	60	0	0	0		C/T	G/C	A/A
Control-0034	2	72	0	0	0		C/T	G/C	A/A
Control-0035	2	61	0	0	0		C/C	G/C	A/A
Control-0036	2	66	0	0	0		C/T	G/C	A/A
Control-0037	1	61	1	0	0		C/C	C/C	A/A
Control-0038	2	54	0	0	0		C/T	G/C	G/A
Control-0039	2	56	0	0	0		C/T	G/G	A/A
Control-0040	2	71	0	0	1		T/T	G/C	A/A
Control-0041	1	72	0	0	0		C/T	G/C	A/A
Control-0042	1	46	0	0	0		C/T	G/G	A/A
Control-0043	1	62	1	0	0		C/T	C/C	A/A
Control-0044	2	59	0	0	0		C/T	C/C	A/A
Control-0045	2	60	0	0	1		C/T	G/G	A/A
Control-0046	2	52	0	0	1		C/C	C/C	A/A
Control-0047	1	56	1	0	0		T/T	G/C	A/A
Control-0048	1	59	1	0	0		T/T	G/C	G/A
Control-0049	1	61	0	0	1		C/T	C/C	A/A
Control-0050	1	63	0	0	1		T/T	C/C	A/A
Control-0051	1	72	0	0	0		C/T	G/C	G/A
Control-0052	1	64	0	1	0		C/T	C/C	A/A
Control-0053	2	67	0	0	1		C/C	C/C	A/A
Control-0054	1	58	1	0	0		C/T	C/C	A/A
Control-0055	2	61	0	0	0		C/T	G/C	A/A
Control-0056	1	41	1	0	1		C/T	G/C	A/A
Control-0057	2	69	0	0	0		C/C	G/G	A/A
Control-0058	1	62	0	0	0		C/C	G/C	A/A
Control-0059	1	81	0	0	0		C/T	C/C	A/A
Control-0060	1	57	0	0	0		T/T	C/C	G/A

Control-0061	2	51	0	0	1	C/T	G/C	A/A
Control-0062	1	55	1	0	0	C/C	G/C	A/A
Control-0063	2	37	0	0	0	T/T	C/C	A/A
Control-0064	1	65	1	1	0	C/T	G/C	A/A
Control-0065	1	57	0	0	1	C/T	G/C	G/A
Control-0066	1	80	0	0	0	C/C	G/C	A/A
Control-0067	2	49	0	0	0	C/C	G/C	A/A
Control-0068	2	46	0	0	0	C/T	G/G	A/A
Control-0069	2	64	0	0	1	C/C	G/C	G/A
Control-0070	2	65	0	0	0	C/C	G/C	A/A
Control-0071	1	78	0	0	1	C/T	C/C	A/A
Control-0072	1	43	0	0	0	C/C	G/G	A/A
Control-0073	1	64	0	0	0	C/C	C/C	G/A
Control-0074	2	56	0	0	1	C/T	G/C	G/A
Control-0075	2	52	0	0	1	T/T	C/C	A/A
Control-0076	2	57	0	0	0	C/T	C/C	A/A
Control-0077	2	67	0	0	0	C/T	G/G	G/A
Control-0078	2	53	0	0	0	C/T	G/C	A/A
Control-0079	1	48	1	0	1	C/T	C/C	A/A
Control-0080	1	57	1	0	0	C/C	C/C	A/A
Control-0081	1	62	1	0	1	C/T	G/C	A/A
Control-0082	2	71	0	0	0	C/C	G/C	A/A
Control-0083	2	53	0	0	0	C/T	G/C	A/A
Control-0084	2	49	0	0	1	C/T	G/C	G/A
Control-0085	2	61	0	0	0	C/T	G/C	A/A
Control-0086	2	62	0	0	1	T/T	C/C	A/A
Control-0087	1	53	1	0	1	T/T	G/C	A/A
Control-0088	1	62	0	0	1	C/C	G/C	G/A
Control-0089	1	63	0	0	0	C/T	G/C	A/A
Control-0090	1	33	0	0	0	C/T	G/G	A/A
Control-0091	1	81	0	0	1	C/T	G/G	A/A
Control-0092	2	59	0	0	1	C/T	C/C	A/A
Control-0093	2	70	0	0	0	C/T	G/C	G/A
Control-0094	2	56	0	0	0	C/T	C/C	A/A
Control-0095	1	58	0	0	0	C/C	G/C	A/A
Control-0096	1	60	1	0	0	C/T	C/C	A/A
Control-0097	1	59	0	0	1	C/C	C/C	A/A
Control-0098	1	76	1	0	0	C/C	C/C	G/G
Control-0099	1	52	0	0	0	C/T	G/G	A/A
Control-0100	2	34	0	0	0	C/C	C/C	A/A
Control-0101	1	60	1	0	1	C/C	C/C	A/A
Control-0102	1	76	1	0	0	C/T	G/C	A/A
Control-0103	2	50	0	0	1	T/T	G/C	G/A
Control-0104	1	57	0	0	0	C/C	G/G	A/A
Control-0105	1	51	0	0	0	C/C	C/C	G/G
Control-0106	1	60	0	0	0	T/T	C/C	A/A
Control-0107	2	65	0	0	1	C/C	G/G	A/A
Control-0108	1	55	0	0	1	T/T	G/C	G/A
Control-0109	1	38	1	0	0	C/T	C/C	A/A
Control-0110	1	64	0	0	1	T/T	G/G	A/A
Control-0111	1	62	0	0	0	C/T	G/G	A/A
Control-0112	1	54	1	0	0	T/T	C/C	G/A
Control-0113	1	64	0	0	0	C/T	C/C	A/A
Control-0114	1	56	0	0	0	T/T	G/G	A/A
Control-0115	2	60	0	0	1	C/T	C/C	A/A
Control-0116	1	64	0	0	0	C/C	G/C	A/A
Control-0117	1	58	0	0	0	C/T	C/C	A/A
Control-0118	1	46	0	0	0	T/T	G/G	A/A
Control-0119	2	72	0	0	0	C/T	G/C	G/A
Control-0120	1	44	1	0	1	C/T	C/C	A/A
Control-0121	2	41	0	0	0	C/C	G/G	A/A
Control-0122	2	67	0	0	0	T/T	C/C	G/A
Control-0123	2	65	0	0	0	T/T	G/G	G/A
Control-0124	2	66	0	0	1	T/T	G/C	G/A
Control-0125	1	61	1	0	0	T/T	C/C	A/A
Control-0126	1	52	0	0	0	C/T	C/C	A/A
Control-0127	2	67	0	0	1	C/T	G/G	A/A
Control-0128	1	60	0	0	1	C/T	G/G	A/A
Control-0129	1	56	1	0	0	C/C	C/C	A/A
Control-0130	1	60	0	0	1	C/T	C/C	G/A
Control-0131	1	57	0	0	1	C/C	C/C	A/A
Control-0132	2	65	0	0	0	C/T	G/G	A/A
Control-0133	2	70	0	0	0	C/T	G/C	G/G
Control-0134	2	69	0	0	1	C/T	G/C	G/A
Control-0135	1	53	1	0	0	C/T	C/C	A/A
Control-0136	2	25	0	0	0	T/T	G/C	A/A
Control-0137	2	43	0	0	0	C/T	C/C	A/A

Control-0138	1	62	1	0	1	C/T	C/C	G/A
Control-0139	2	74	0	0	1	C/T	C/C	G/A
Control-0140	2	73	0	0	0	T/T	G/C	G/G
Control-0141	2	62	0	0	0	C/T	C/C	A/A
Control-0142	1	58	0	0	0	T/T	C/C	A/A
Control-0143	2	69	0	0	1	C/T	G/G	A/A
Control-0144	2	51	0	0	0	C/C	G/C	A/A
Control-0145	1	61	0	0	1	C/C	C/C	A/A
Control-0146	1	74	0	0	0	C/T	G/C	A/A
Control-0147	1	55	0	0	1	C/T	G/C	A/A
Control-0148	2	72	0	0	0	C/T	C/C	A/A
Control-0149	1	56	0	0	0	C/T	G/C	A/A
Control-0150	2	78	0	0	1	C/T	G/G	G/A
Control-0151	2	63	0	0	1	C/T	C/C	G/A
Control-0152	2	61	0	0	1	C/T	G/C	A/A
Control-0153	1	59	1	0	1	T/T	C/C	A/A
Control-0154	2	63	0	0	1	C/T	G/C	A/A
Control-0155	2	39	0	0	1	T/T	G/C	A/A
Control-0156	1	58	0	0	0	C/T	C/C	A/A
Control-0157	1	54	0	0	0	T/T	G/G	A/A
Control-0158	1	57	1	0	1	C/T	G/C	A/A
Control-0159	1	66	0	0	0	T/T	C/C	G/A
Control-0160	1	74	0	0	1	C/T	C/C	A/A
Control-0161	2	50	0	0	0	T/T	G/C	A/A
Control-0162	1	68	0	0	1	C/T	G/G	A/A
Control-0163	1	60	0	0	1	T/T	C/C	G/A
Control-0164	2	59	0	0	1	T/T	G/C	G/A
Control-0165	1	73	0	0	1	C/C	G/C	A/A
Control-0166	1	75	1	0	1	T/T	C/C	A/A
Control-0167	1	65	0	0	0	C/C	C/C	G/A
Control-0168	1	62	0	0	0	C/T	G/C	A/A
Control-0169	1	57	0	0	0	T/T	C/C	A/A
Control-0170	2	76	0	0	1	C/T	G/G	A/A
Control-0171	2	60	0	0	1	T/T	G/C	A/A
Control-0172	1	59	0	0	0	C/C	G/C	A/A
Control-0173	2	52	0	0	0	C/T	G/C	A/A
Control-0174	1	52	0	0	1	T/T	C/C	A/A
Control-0175	1	76	0	0	1	C/C	G/C	A/A
Control-0176	1	75	0	0	1	C/T	C/C	A/A
Control-0177	2	51	0	0	1	C/T	C/C	A/A
Control-0178	1	66	0	0	1	C/C	C/C	A/A
Control-0179	2	61	0	0	0	C/T	C/C	A/A
Control-0180	2	62	0	0	1	T/T	G/C	A/A
Control-0181	1	40	0	0	1	C/T	G/C	A/A
Control-0182	1	50	0	1	1	T/T	C/C	A/A
Control-0183	2	59	0	0	0	T/T	C/C	G/A
Control-0184	1	48	0	0	1	C/T	C/C	A/A
Control-0185	1	74	0	1	1	T/T	C/C	A/A
Control-0186	1	84	0	0	0	C/T	G/C	G/G
Control-0187	1	57	0	0	0	C/C	C/C	G/A
Control-0188	1	69	0	0	1	C/T	C/C	A/A
Control-0189	1	62	0	0	1	C/T	C/C	A/A
Control-0190	2	57	0	0	1	C/T	C/C	A/A
Control-0191	1	37	0	0	0	C/T	G/C	A/A
Control-0192	1	64	0	0	1	T/T	G/C	A/A
Control-0193	1	64	0	0	0	C/T	G/G	A/A
Control-0194	1	77	0	0	0	T/T	G/C	G/A
Control-0195	1	60	0	0	0	C/T	C/C	A/A
Control-0196	1	68	0	0	0	C/T	G/G	G/A
Control-0197	1	81	0	0	1	C/C	G/C	G/A
Control-0198	2	55	0	0	1	T/T	C/C	A/A
Control-0199	1	79	1	1	0	T/T	C/C	G/A
Control-0200	1	71	1	1	0	C/T	G/C	A/A
Control-0201	1	64	0	0	0	C/T	G/C	A/A
Control-0202	2	57	0	0	0	C/T	C/C	G/A
Control-0203	2	78	0	0	1	C/C	G/G	G/A
Control-0204	1	58	1	0	1	C/T	C/C	A/A
Control-0205	1	74	0	0	0	C/C	G/C	A/A
Control-0206	2	54	0	0	1	C/C	G/C	A/A
Control-0207	1	59	0	1	0	C/T	C/C	A/A
Control-0208	1	70	0	0	0	C/C	C/C	A/A
Control-0209	1	55	0	0	1	C/C	G/C	G/A
Control-0210	1	58	0	0	0	T/T	C/C	A/A
Control-0211	1	59	0	0	1	T/T	G/G	A/A
Control-0212	1	60	0	0	0	C/T	C/C	G/A
Control-0213	1	58	0	0	0	T/T	G/C	A/A
Control-0214	2	60	0	0	0	T/T	C/C	A/A

Control-0215	2	61	0	0	0	C/T	C/C	A/A
Control-0216	2	67	0	0	0	C/T	C/C	G/A
Control-0217	1	59	1	0	1	C/T	C/C	A/A
Control-0218	2	55	0	0	0	C/T	C/C	A/A
Control-0219	1	66	0	0	0	T/T	G/C	A/A
Control-0220	1	64	0	0	1	C/T	C/C	A/A
Control-0221	1	56	1	0	1	C/C	C/C	A/A
Control-0222	2	61	0	0	1	T/T	C/C	A/A
Control-0223	1	60	1	0	0	C/T	C/C	G/A
Control-0224	2	66	0	0	0	C/T	G/C	G/A
Control-0225	2	63	0	0	1	C/T	G/C	G/A
Control-0226	1	65	1	0	1	C/C	G/G	A/A
Control-0227	1	60	0	0	0	C/T	C/C	G/A
Control-0228	2	51	0	0	0	C/C	C/C	A/A
Control-0229	1	57	0	0	1	C/T	G/C	A/A
Control-0230	2	79	0	0	1	T/T	C/C	G/A
Control-0231	1	55	1	1	0	C/T	G/C	A/A
Control-0232	1	51	0	0	1	T/T	C/C	A/A
Control-0233	2	66	0	0	0	C/T	C/C	A/A
Control-0234	1	53	1	0	0	C/T	G/G	A/A
Control-0235	1	61	0	0	1	C/C	G/C	G/G
Control-0236	1	62	0	0	1	C/T	C/C	A/A
Control-0237	1	60	1	0	1	T/T	G/C	G/A
Control-0238	1	71	1	1	1	C/T	G/C	G/A
Control-0239	1	67	0	0	0	C/C	C/C	A/A
Control-0240	2	69	0	0	0	C/C	G/C	A/A
Control-0241	1	62	0	0	0	T/T	G/C	A/A
Control-0242	1	61	1	0	0	C/C	G/C	G/A
Control-0243	1	62	1	0	0	C/T	G/C	A/A
Control-0244	2	70	0	0	1	C/T	C/C	A/A
Control-0245	2	56	0	0	1	C/C	G/C	A/A
Control-0246	2	68	0	0	1	T/T	C/C	G/A
Control-0247	2	53	0	0	1	C/T	G/C	A/A
Control-0248	2	56	0	0	1	C/C	G/G	A/A
Control-0249	2	48	0	0	0	C/C	G/C	A/A
Control-0250	1	61	0	0	1	C/C	G/C	G/A
Control-0251	1	67	0	0	1	C/T	G/C	A/A
Control-0252	1	57	0	0	0	C/T	C/C	A/A
Control-0253	1	63	0	0	0	C/T	G/C	A/A
Control-0254	1	74	1	0	0	T/T	C/C	G/A
Control-0255	1	67	0	0	0	C/T	C/C	A/A
Control-0256	1	61	0	0	0	C/T	C/C	A/A
Control-0257	1	74	0	1	0	T/T	G/C	G/A
Control-0258	2	65	0	0	1	C/C	G/C	A/A
Control-0259	1	71	1	0	0	T/T	C/C	A/A
Control-0260	1	59	1	0	0	T/T	G/C	G/A
Control-0261	1	69	1	0	0	C/T	G/G	A/A
Control-0262	2	78	0	0	0	C/C	C/C	A/A
Control-0263	1	59	0	0	1	C/T	G/C	A/A
Control-0264	1	63	0	0	1	C/C	G/C	G/A
Control-0265	2	63	0	0	1	T/T	G/C	G/A
Control-0266	1	71	0	0	1	C/T	G/G	G/A
Control-0267	1	64	0	1	0	C/T	G/G	A/A
Control-0268	1	56	0	0	0	C/C	C/C	G/A
Control-0269	2	72	0	0	1	C/T	C/C	A/A
Control-0270	1	62	1	0	0	C/T	G/G	A/A
Control-0271	2	51	0	0	0	C/T	G/G	A/A
Control-0272	1	58	1	0	0	T/T	G/C	G/A
Control-0273	1	60	0	0	0	T/T	G/C	G/A
Control-0274	2	66	0	0	0	C/T	G/C	G/A
Control-0275	2	70	0	0	0	C/T	C/C	G/A
Control-0276	1	57	0	0	1	T/T	C/C	A/A
Control-0277	2	61	0	0	0	C/T	C/C	G/A
Control-0278	2	59	0	0	0	C/C	C/C	A/A
Control-0279	2	54	0	0	0	C/T	C/C	A/A
Control-0280	1	64	0	0	0	T/T	G/C	A/A
Control-0281	2	48	0	0	1	C/T	G/C	A/A
Control-0282	1	63	1	0	0	C/C	G/C	A/A
Control-0283	2	69	0	0	0	T/T	G/C	A/A
Control-0284	2	74	0	0	1	C/T	G/C	G/A
Control-0285	1	59	0	0	1	C/C	G/G	A/A
Control-0286	2	63	0	0	1	T/T	C/C	A/A
Control-0287	1	73	0	0	1	C/C	C/C	G/A
Control-0288	2	68	0	0	0	T/T	G/C	A/A
Control-0289	1	65	0	0	0	T/T	G/G	G/A
Control-0290	1	73	0	0	1	C/T	G/G	G/G
Control-0291	2	59	0	0	0	C/T	G/G	A/A

Control-0292	1	65	1	0	0	T/T	G/C	A/A
Control-0293	1	66	0	0	0	T/T	C/C	G/A
Control-0294	2	67	0	0	0	C/T	C/C	A/A
Control-0295	2	55	0	0	0	C/C	C/C	A/A
Control-0296	1	71	0	0	1	C/C	G/C	G/A
Control-0297	2	67	0	0	0	C/C	C/C	A/A
Control-0298	1	53	0	0	1	C/T	C/C	A/A
Control-0299	2	67	0	0	1	T/T	C/C	A/A
Control-0300	2	68	0	0	1	C/C	G/C	G/A
Control-0301	1	55	1	0	0	C/T	G/C	A/A
Control-0302	2	63	0	0	1	T/T	G/C	A/A
Control-0303	2	72	0	0	0	C/T	G/C	A/A
Control-0304	1	56	1	0	1	C/T	G/C	A/A
Control-0305	1	56	0	0	1	C/T	G/C	A/A
Control-0306	1	65	0	0	0	T/T	G/C	A/A
Control-0307	1	59	0	0	1	C/C	G/C	A/A
Control-0308	1	72	0	0	0	C/T	C/C	A/A
Control-0309	1	59	1	0	1	C/C	C/C	A/A
Control-0310	1	77	1	0	0	C/T	G/C	A/A
Control-0311	1	60	0	0	0	T/T	C/C	G/A
Control-0312	1	56	1	0	0	C/T	G/C	G/A
Control-0313	1	62	0	0	0	T/T	C/C	A/A
Control-0314	2	65	0	0	1	C/T	G/C	A/A
Control-0315	1	49	1	0	0	C/T	G/C	A/A
Control-0316	1	71	1	0	1	T/T	C/C	A/A
Control-0317	1	52	0	0	0	C/T	G/C	A/A
Control-0318	1	85	1	0	1	T/T	G/C	G/A
Control-0319	1	47	0	0	0	T/T	C/C	G/A
Control-0320	1	48	0	0	1	T/T	C/C	A/A
Control-0321	2	46	0	0	0	C/T	C/C	A/A
Control-0322	2	51	0	0	0	C/T	C/C	G/A
Control-0323	1	67	1	0	1	T/T	G/C	A/A
Control-0324	1	78	0	0	0	T/T	C/C	A/A
Control-0325	1	48	1	0	0	T/T	C/C	G/A
Control-0326	2	56	0	0	0	T/T	G/C	A/A
Control-0327	2	51	0	0	1	C/T	G/G	A/A
Control-0328	2	63	0	0	1	C/T	G/C	A/A
Control-0329	2	54	0	0	1	C/T	C/C	A/A
Control-0330	2	52	0	0	1	T/T	G/C	A/A
Control-0331	1	52	0	0	1	C/T	G/C	G/A
Control-0332	1	58	0	0	0	C/C	C/C	A/A
Control-0333	1	51	0	0	0	C/T	G/C	A/A
Control-0334	2	68	0	0	1	C/T	G/C	G/A
Control-0335	1	52	0	0	0	C/C	C/C	A/A
Control-0336	2	51	0	0	1	C/T	C/C	A/A
Control-0337	2	66	0	0	1	T/T	C/C	A/A
Control-0338	2	62	0	0	0	C/T	C/C	A/A
Control-0339	1	66	0	0	0	T/T	G/C	G/A
Control-0340	1	65	0	0	1	T/T	C/C	A/A
Control-0341	2	60	0	0	0	T/T	G/C	A/A
Control-0342	1	48	0	0	1	C/T	G/C	A/A
Control-0343	2	53	0	0	1	C/T	G/C	A/A
Control-0344	1	70	1	0	1	C/T	G/C	A/A
Control-0345	2	60	0	0	1	C/T	G/C	A/A
Control-0346	2	63	0	0	0	T/T	G/C	A/A
Control-0347	2	61	0	0	1	C/T	G/C	A/A
Control-0348	1	55	0	0	0	C/T	G/C	A/A
Control-0349	1	69	0	0	1	T/T	C/C	A/A
Control-0350	1	67	0	0	1	C/T	C/C	G/A
Control-0351	1	62	0	0	0	C/C	G/C	A/A
Control-0352	2	49	0	0	0	C/T	C/C	G/A
Control-0353	1	55	0	0	1	C/T	C/C	A/A
Control-0354	1	62	1	0	0	C/T	G/C	A/A
Control-0355	1	48	1	0	1	T/T	G/C	A/A
Control-0356	1	49	1	0	1	C/T	G/C	G/A
Control-0357	1	61	0	0	1	C/T	C/C	A/A
Control-0358	2	53	0	0	0	C/T	C/C	A/A
Control-0359	1	52	0	0	0	T/T	G/C	A/A
Control-0360	2	67	0	0	0	C/C	C/C	A/A
Control-0361	1	30	0	0	0	T/T	G/G	G/G
Control-0362	1	58	0	0	0	C/C	C/C	G/A
Control-0363	2	61	0	0	1	C/T	C/C	A/A
Control-0364	1	54	0	0	0	C/T	C/C	A/A
Control-0365	2	42	0	0	0	T/T	C/C	A/A
Control-0366	2	61	0	0	0	C/T	G/C	G/A
Control-0367	1	57	0	0	0	T/T	G/G	A/A
Control-0368	1	50	1	0	1	C/T	C/C	A/A

Control-0369	2	66	0	0	0	C/T	C/C	A/A
Control-0370	1	55	1	0	1	C/T	G/G	A/A
Control-0371	2	52	0	0	1	T/T	G/C	A/A
Control-0372	1	41	0	0	0	C/C	C/C	A/A
Control-0373	2	63	0	0	1	T/T	G/C	A/A
Control-0374	1	59	0	0	1	C/T	G/C	A/A
Control-0375	1	74	1	1	0	T/T	G/C	A/A
Control-0376	1	68	0	0	0	C/T	G/C	G/A
Control-0377	1	63	1	1	1	T/T	G/C	G/A
Control-0378	1	60	1	0	0	C/T	C/C	G/A
Control-0379	1	59	0	0	1	T/T	G/C	G/A
Control-0380	2	57	0	0	1	C/C	C/C	A/A
Control-0381	1	60	1	0	1	C/T	C/C	G/A
Control-0382	2	42	0	0	0	C/T	G/C	A/A
Control-0383	1	54	1	0	0	C/C	G/C	A/A
Control-0384	2	42	0	0	1	C/T	C/C	A/A
Control-0385	1	51	0	0	0	T/T	G/C	A/A
Control-0386	1	50	0	0	1	T/T	G/C	A/A
Control-0387	1	58	0	0	1	T/T	C/C	G/A
Control-0388	2	65	1	0	1	C/T	C/C	G/A
Control-0389	1	51	0	0	0	C/T	G/C	A/A
Control-0390	1	64	0	0	1	T/T	G/C	A/A
Control-0391	1	27	0	0	0	C/T	C/C	G/A
Control-0392	1	63	0	0	0	C/T	G/C	A/A
Control-0393	1	53	0	0	0	T/T	G/C	A/A
Control-0394	2	64	0	0	0	T/T	C/C	A/A
Control-0395	1	55	1	0	0	T/T	C/C	A/A
Control-0396	1	39	1	0	0	T/T	C/C	G/A
Control-0397	1	69	0	0	1	C/C	C/C	A/A
Control-0398	1	50	1	0	0	C/T	C/C	A/A
Control-0399	1	76	0	0	0	T/T	G/C	A/A
Control-0400	1	79	1	0	0	C/T	C/C	A/A
Control-0401	1	52	0	0	0	C/C	C/C	A/A
Control-0402	2	58	0	0	1	C/C	G/C	A/A
Control-0403	1	43	0	0	0	C/C	G/C	A/A
Control-0404	2	53	0	0	0	C/T	C/C	A/A
Control-0405	1	78	0	0	0	C/T	G/C	G/A
Control-0406	1	63	1	0	0	C/T	G/C	G/A
Control-0407	1	57	1	0	1	C/T	G/C	G/A
Control-0408	2	60	0	0	0	C/T	G/G	A/A
Control-0409	1	67	0	0	1	T/T	G/C	G/A
Control-0410	1	66	0	0	1	C/T	G/C	A/A
Control-0411	1	62	0	0	1	T/T	G/G	G/A
Control-0412	2	61	0	0	1	C/T	G/G	A/A
Control-0413	1	61	0	0	1	C/T	G/C	A/A
Control-0414	1	66	0	0	1	C/C	G/G	A/A
Control-0415	1	52	0	0	1	C/C	C/C	G/A
Control-0416	1	60	1	0	0	T/T	C/C	G/A
Control-0417	1	72	1	1	0	C/C	G/C	A/A
Control-0418	1	70	0	0	0	C/C	G/C	A/A
Control-0419	1	68	0	0	0	T/T	C/C	A/A
Control-0420	1	71	1	0	0	C/T	G/C	A/A
Control-0421	2	69	0	0	1	C/T	G/C	A/A
Control-0422	2	61	0	0	1	C/C	C/C	G/A
Control-0423	1	69	0	0	1	C/T	G/C	A/A
Control-0424	1	74	1	0	1	C/C	G/C	A/A
Control-0425	2	52	0	0	1	T/T	G/G	A/A
Control-0426	1	41	0	0	0	T/T	C/C	A/A
Control-0427	1	52	0	0	0	T/T	G/C	A/A
Control-0428	2	44	0	0	0	C/C	G/C	G/G
Control-0429	2	50	0	0	0	C/T	G/C	A/A
Control-0430	1	63	0	0	1	C/T	G/C	G/A
Control-0431	1	59	1	0	1	T/T	G/G	A/A
Control-0432	1	44	0	0	1	C/T	C/C	G/A
Control-0433	2	66	0	0	0	C/C	C/C	G/A
Control-0434	1	59	1	1	0	T/T	C/C	A/A
Control-0435	1	61	1	0	1	C/T	G/C	G/A
Control-0436	2	53	0	0	1	T/T	C/C	A/A
Control-0437	1	66	1	0	1	C/T	G/C	A/A
Control-0438	2	54	0	0	0	C/T	C/C	A/A
Control-0439	2	51	0	0	1	C/C	G/C	G/A
Control-0440	2	61	0	0	0	C/T	G/G	A/A
Control-0441	1	56	0	0	1	T/T	G/C	A/A
Control-0442	1	52	0	0	1	C/T	C/C	G/A
Control-0443	2	49	0	0	1	C/T	C/C	A/A
Control-0444	2	48	0	0	0	C/T	C/C	A/A
Control-0445	2	45	0	0	1	C/C	G/C	A/A

Control-0446	2	47	0	0	0	C/C	C/C	G/A
Control-0447	2	65	0	0	1	T/T	C/C	A/A
Control-0448	1	73	1	0	0	C/T	G/C	A/A
Control-0449	2	59	0	0	0	C/C	C/C	A/A
Control-0450	1	59	0	0	0	C/T	G/C	G/A
Control-0451	1	67	0	0	0	C/T	C/C	A/A
Control-0452	1	59	0	0	1	T/T	C/C	G/A
Control-0453	1	59	0	0	1	C/T	G/C	A/A
Control-0454	1	47	1	0	1	C/T	G/C	A/A
Control-0455	2	68	0	0	0	C/T	G/G	A/A
Control-0456	1	64	0	1	0	C/C	G/C	A/A
Control-0457	2	50	0	0	1	C/T	G/C	G/A
Control-0458	1	74	1	0	0	C/C	G/G	A/A
Control-0459	2	50	0	0	0	T/T	G/C	A/A
Control-0460	1	70	0	0	0	C/T	C/C	A/A
Control-0461	2	63	0	0	0	C/T	G/C	A/A
Control-0462	1	53	0	0	0	C/C	G/C	A/A
Control-0463	2	59	0	0	0	C/T	G/C	G/A
Control-0464	1	58	0	0	0	C/T	C/C	G/A
Control-0465	1	68	0	0	0	T/T	G/C	A/A
Control-0466	2	53	0	0	1	C/T	C/C	A/A
Control-0467	2	65	0	0	1	C/T	C/C	G/A
Control-0468	1	73	1	0	0	C/T	C/C	A/A
Control-0469	2	56	0	0	1	C/T	G/C	A/A
Control-0470	1	59	0	0	0	C/C	G/C	A/A
Control-0471	2	51	0	0	1	T/T	C/C	A/A
Control-0472	1	62	0	0	1	T/T	G/C	G/A
Control-0473	1	55	0	0	0	T/T	G/C	A/A
Control-0474	2	59	0	0	1	C/T	G/C	A/A
Control-0475	1	62	0	0	1	C/T	C/C	A/A
Control-0476	1	69	0	0	0	T/T	G/C	G/A
Control-0477	1	29	0	0	1	C/T	G/G	A/A
Control-0478	1	59	0	0	0	C/T	G/C	A/A
Control-0479	1	52	1	0	0	C/T	G/C	A/A
Control-0480	1	60	0	0	1	C/T	G/C	A/A
Control-0481	2	61	0	0	0	T/T	C/C	A/A
Control-0482	2	69	0	0	1	C/T	C/C	A/A
Control-0483	1	59	0	0	1	C/C	C/C	A/A
Control-0484	2	63	0	0	1	C/C	C/C	A/A
Control-0485	2	66	0	0	0	C/T	G/C	G/A
Control-0486	1	63	0	0	1	C/T	G/C	A/A
Control-0487	1	74	0	0	1	C/C	C/C	A/A
Control-0488	2	59	0	0	1	C/C	C/C	A/A
Control-0489	1	57	0	0	1	C/T	C/C	A/A
Control-0490	1	63	0	0	0	C/T	C/C	A/A
Control-0491	2	68	0	0	1	C/T	G/C	A/A
Control-0492	1	61	1	0	1	T/T	C/C	A/A
Control-0493	1	53	0	0	1	C/T	G/C	G/A
Control-0494	2	58	0	0	0	C/T	G/C	A/A
Control-0495	1	62	0	0	1	C/T	G/C	A/A
Control-0496	1	57	1	0	0	C/T	G/C	A/A
Control-0497	1	57	1	0	1	C/C	C/C	G/A
Control-0498	1	46	1	1	1	C/T	G/C	G/A
Control-0499	1	60	1	1	0	C/T	G/G	A/A
Control-0500	2	65	0	0	0	C/T	G/C	G/A
Control-0501	1	62	0	0	0	T/T	C/C	A/A
Control-0502	1	54	1	0	0	T/T	C/C	G/A
Control-0503	1	53	0	0	0	T/T	C/C	A/A
Control-0504	2	64	0	0	1	C/T	G/C	A/A
Control-0505	1	64	1	1	1	C/C	G/C	A/A
Control-0506	1	55	0	0	1	T/T	C/C	A/A
Control-0507	2	60	0	0	0	T/T	G/C	A/A
Control-0508	1	59	1	0	0	C/T	C/C	A/A
Control-0509	1	59	1	0	1	C/T	G/C	G/A
Control-0510	1	63	1	0	0	T/T	C/C	A/A
Control-0511	1	62	0	1	1	T/T	C/C	A/A
Control-0512	1	53	0	0	1	T/T	G/C	A/A
Control-0513	1	52	0	0	0	C/C	G/C	A/A
Control-0514	1	54	1	0	1	C/T	G/C	A/A
Control-0515	1	55	0	0	0	C/T	C/C	A/A
Control-0516	2	69	0	0	1	C/T	G/C	G/A
Control-0517	1	65	0	1	0	C/T	C/C	A/A
Control-0518	1	58	0	1	1	C/T	C/C	G/A
Control-0519	1	51	1	0	1	C/T	C/C	A/A
Control-0520	1	48	1	0	1	T/T	C/C	G/A
Control-0521	2	54	0	0	0	C/C	G/C	A/A
Control-0522	2	60	0	0	1	C/T	G/G	G/A

Control-0523	2	64	0	0	0	C/C	C/C	A/A
Control-0524	1	59	1	1	1	C/T	G/C	G/A
Control-0525	2	59	0	0	0	C/T	G/C	A/A
Control-0526	1	56	1	0	0	C/T	G/C	A/A
Control-0527	1	60	0	0	0	T/T	C/C	A/A
Control-0528	1	51	0	0	0	C/C	C/C	A/A
Control-0529	1	58	1	0	0	C/T	C/C	A/A
Control-0530	1	65	0	0	0	T/T	C/C	A/A
Control-0531	1	63	0	0	0	C/T	C/C	A/A
Control-0532	2	58	0	0	0	T/T	G/C	A/A
Control-0533	2	61	0	0	0	C/T	G/C	A/A
Control-0534	1	62	1	1	1	T/T	C/C	G/A
Control-0535	1	54	0	0	0	C/T	G/G	G/A
Control-0536	1	58	0	0	1	T/T	C/C	A/A
Control-0537	2	60	0	0	1	C/T	G/C	A/A
Control-0538	1	59	1	0	1	C/T	G/C	A/A
Control-0539	2	59	0	0	1	C/C	G/G	A/A
Control-0540	1	64	0	0	1	C/T	C/C	A/A
Control-0541	1	58	1	0	0	T/T	G/C	A/A
Control-0542	1	60	0	0	1	C/T	G/C	G/A
Control-0543	1	60	0	1	1	T/T	C/C	A/A
Control-0544	2	50	0	0	0	C/T	G/C	A/A
Control-0545	1	54	1	1	1	T/T	C/C	G/A
Control-0546	1	58	0	0	1	C/C	G/G	A/A
Control-0547	1	57	0	0	0	C/C	G/G	G/A
Control-0548	1	62	0	0	1	C/T	C/C	G/A
Control-0549	1	65	0	0	1	T/T	G/C	A/A
Control-0550	1	61	0	0	1	C/T	C/C	G/A
Control-0551	2	61	0	0	0	C/T	C/C	A/A
Control-0552	2	57	0	0	0	C/T	G/C	G/A
Control-0553	1	56	1	1	1	C/T	C/C	A/A
Control-0554	2	59	0	0	1	T/T	G/G	A/A
Control-0555	2	61	0	0	1	T/T	G/G	A/A
Control-0556	2	54	0	1	0	T/T	G/C	A/A
Control-0557	1	65	0	0	1	C/T	C/C	A/A
Control-0558	1	64	1	0	0	C/T	C/C	G/A
Control-0559	2	66	0	0	1	C/T	G/C	A/A
Control-0560	1	56	1	1	0	C/T	G/C	A/A
Control-0561	1	51	1	0	1	C/T	G/C	A/A
Control-0562	1	54	0	0	1	C/T	G/G	G/A
Control-0563	1	59	1	1	0	C/T	G/C	G/A
Control-0564	1	57	1	0	1	C/C	C/C	A/A
Control-0565	1	52	1	1	0	C/C	G/C	A/A
Control-0566	2	65	0	0	0	T/T	G/C	A/A
Control-0567	1	59	1	0	0	C/T	G/C	A/A
Control-0568	1	64	0	1	1	C/T	C/C	A/A
Control-0569	1	57	1	0	1	C/T	C/C	A/A
Control-0570	1	52	0	0	1	C/T	G/C	A/A
Control-0571	1	51	0	0	1	C/T	G/G	A/A
Control-0572	1	63	0	0	0	T/T	C/C	A/A
Control-0573	1	63	1	0	1	C/T	G/C	G/A
Control-0574	2	68	0	0	1	C/T	G/G	A/A
Control-0575	2	55	0	0	1	T/T	C/C	A/A
Control-0576	1	55	1	1	1	C/T	G/C	A/A
Control-0577	1	64	1	1	0	T/T	G/G	G/A
Control-0578	1	60	0	0	0	C/C	C/C	G/A
Control-0579	1	50	0	0	0	C/T	G/G	A/A
Control-0580	1	51	0	1	1	C/C	G/C	G/A
Control-0581	1	58	1	1	0	T/T	G/C	A/A
Control-0582	1	57	0	0	0	C/T	C/C	G/G
Control-0583	1	61	1	1	1	C/T	C/C	G/A
Control-0584	1	63	1	0	0	T/T	C/C	G/A
Control-0585	1	60	1	0	0	C/C	C/C	A/A
Control-0586	2	66	0	0	1	C/C	G/C	A/A
Control-0587	1	65	1	0	0	C/T	G/C	G/A
Control-0588	2	63	0	0	0	T/T	C/C	G/A
Control-0589	2	59	0	0	1	C/T	G/C	A/A
Control-0590	1	59	1	1	0	C/C	G/C	G/A
Control-0591	1	61	1	1	0	C/T	G/C	A/A
Control-0592	1	50	0	1	1	C/C	C/C	G/A
Control-0593	2	50	0	0	1	T/T	G/C	A/A
Control-0594	2	56	0	0	0	C/T	G/C	G/A
Control-0595	1	55	0	0	0	T/T	G/C	G/A
Control-0596	2	59	0	0	1	C/T	C/C	G/G
Control-0597	1	59	0	0	0	T/T	G/C	A/A
Control-0598	1	59	1	0	0	C/T	G/G	A/A
Control-0599	1	56	0	0	1	T/T	G/C	A/A

Control-0600	2	63	0	0	1	C/T	G/G	A/A
Control-0601	1	52	1	0	1	C/T	C/C	A/A
Control-0602	2	68	0	0	1	C/T	C/C	A/A
Control-0603	2	53	0	0	1	C/T	G/C	A/A
Control-0604	1	61	1	1	0	C/C	C/C	A/A
Control-0605	2	68	0	0	0	C/T	G/G	G/A
Control-0606	1	56	0	0	0	C/C	G/C	A/A
Control-0607	1	64	1	1	0	C/C	G/C	A/A
Control-0608	2	53	0	0	1	T/T	G/G	A/A
Control-0609	1	52	0	0	1	C/T	C/C	A/A
Control-0610	1	52	1	0	1	T/T	G/C	A/A
Control-0611	1	66	0	0	0	C/T	C/C	A/A
Control-0612	2	57	0	0	0	T/T	C/C	A/A
Control-0613	1	56	1	1	0	C/T	G/G	A/A
Control-0614	2	62	0	0	0	T/T	G/C	G/A
Control-0615	2	61	0	0	0	C/T	C/C	A/A
Control-0616	2	57	0	0	0	C/C	C/C	A/A
Control-0617	2	63	0	0	1	C/T	C/C	A/A
Control-0618	2	58	0	0	1	C/T	C/C	G/A
Control-0619	1	50	0	0	0	C/C	G/G	A/A
Control-0620	2	55	0	0	1	C/T	G/C	A/A
Control-0621	1	56	0	0	1	C/T	G/C	A/A
Control-0622	2	59	0	0	0	T/T	G/C	A/A
Control-0623	1	65	1	1	0	T/T	G/C	G/G
Control-0624	2	47	0	0	1	C/C	G/C	G/A
Control-0625	1	58	1	1	0	C/T	C/C	A/A
Control-0626	1	60	1	1	0	T/T	G/C	A/A
Control-0627	1	69	0	0	1	C/T	C/C	A/A
Control-0628	2	62	0	0	0	C/T	C/C	G/A
Control-0629	2	61	0	0	1	C/T	G/G	A/A
Control-0630	2	69	0	0	1	C/T	G/C	A/A
Control-0631	2	53	0	0	0	C/T	G/C	A/A
Control-0632	1	58	0	0	1	C/T	G/C	A/A
Control-0633	2	61	0	0	0	C/C	C/C	A/A
Control-0634	1	61	0	0	0	T/T	C/C	A/A
Control-0635	2	53	0	0	0	C/C	C/C	A/A
Control-0636	1	69	0	0	1	C/T	G/C	A/A
Control-0637	2	45	0	0	1	C/T	C/C	A/A
Control-0638	1	55	1	1	0	C/T	G/C	A/A
Control-0639	2	52	0	0	1	T/T	G/C	G/A
Control-0640	1	67	0	0	1	C/C	G/C	A/A
Control-0641	1	53	1	0	1	C/C	G/C	G/A
Control-0642	2	59	0	0	1	C/T	C/C	A/A
Control-0643	1	49	1	1	0	C/T	C/C	A/A
Control-0644	2	59	0	0	1	C/C	G/C	G/A
Control-0645	1	55	1	0	1	T/T	G/C	G/G
Control-0646	1	62	0	0	1	C/C	G/C	A/A
Control-0647	2	63	0	0	1	T/T	G/C	A/A
Control-0648	1	57	0	1	0	C/T	G/C	A/A
Control-0649	1	53	0	0	0	T/T	G/C	A/A
Control-0650	2	68	0	0	0	C/T	G/C	A/A
Control-0651	1	45	0	0	1	T/T	G/C	A/A
Control-0652	1	67	1	1	0	T/T	C/C	A/A
Control-0653	1	52	0	0	1	C/T	C/C	G/A
Control-0654	2	47	0	0	0	T/T	G/C	A/A
Control-0655	1	53	0	0	1	C/T	C/C	G/A
Control-0656	2	56	0	0	0	T/T	G/G	G/A
Control-0657	2	53	0	0	0	C/C	G/C	A/A
Control-0658	1	52	0	1	0	C/C	C/C	A/A
Control-0659	2	67	0	0	1	C/T	C/C	G/A
Control-0660	2	48	0	0	1	C/T	C/C	A/A
Control-0661	1	52	0	0	1	T/T	G/G	A/A
Control-0662	2	65	0	0	1	C/T	C/C	A/A
Control-0663	2	48	0	0	0	C/T	C/C	A/A
Control-0664	2	59	0	0	0	T/T	C/C	G/A
Control-0665	1	69	1	1	0	C/T	G/C	A/A
Control-0666	1	50	0	0	0	C/T	G/C	A/A
Control-0667	2	53	0	0	1	C/C	C/C	A/A
Control-0668	1	54	0	0	1	C/T	C/C	A/A
Control-0669	1	56	0	0	1	C/T	C/C	A/A
Control-0670	2	58	0	0	1	C/C	C/C	A/A
Control-0671	1	60	0	0	0	C/T	G/C	A/A
Control-0672	1	82	1	0	0	C/T	C/C	A/A
Control-0673	1	60	1	0	0	C/T	G/G	A/A
Control-0674	2	57	0	0	1	C/T	C/C	A/A
Control-0675	1	55	0	0	0	T/T	C/C	A/A
Control-0676	2	68	0	0	0	C/T	C/C	G/A

Control-0677	2	61	0	0	1	C/T	G/G	A/A
Control-0678	1	45	1	1	0	C/T	G/C	G/A
Control-0679	2	64	0	0	0	C/C	G/C	A/A
Control-0680	2	66	0	0	0	T/T	G/C	G/A
Control-0681	2	67	0	0	0	C/T	G/C	A/A
Control-0682	2	51	0	0	1	C/T	G/G	A/A
Control-0683	2	67	0	0	1	C/T	G/G	A/A
Control-0684	1	51	1	0	0	C/T	C/C	A/A
Control-0685	1	65	0	0	0	T/T	G/C	A/A
Control-0686	2	61	0	0	1	T/T	G/G	G/A
Control-0687	2	65	0	0	0	C/T	G/C	A/A
Control-0688	1	65	0	0	1	C/T	C/C	G/A
Control-0689	1	60	1	0	1	T/T	C/C	A/A
Control-0690	1	58	1	1	1	C/T	G/C	G/A
Control-0691	2	67	0	0	0	T/T	G/C	A/A
Control-0692	2	66	0	0	1	C/T	C/C	A/A
Control-0693	1	68	0	0	1	C/T	G/C	A/A
Control-0694	1	43	0	0	1	C/C	G/G	A/A
Control-0695	2	47	0	0	1	C/T	G/C	A/A
Control-0696	2	52	0	0	1	C/T	G/C	G/A
Control-0697	1	52	0	0	1	C/C	C/C	A/A
Control-0698	2	61	0	0	0	C/C	G/C	A/A
Control-0699	2	66	0	0	0	C/C	G/C	A/A
Control-0700	2	59	0	0	0	C/T	G/C	A/A
Control-0701	1	63	1	0	1	C/C	G/G	G/A
Control-0702	1	59	1	1	0	C/T	G/C	A/A
Control-0703	2	56	0	0	1	C/C	G/C	A/A
Control-0704	2	69	0	0	1	C/T	C/C	A/A
Control-0705	1	58	1	1	1	C/C	G/C	A/A
Control-0706	1	49	1	1	1	T/T	G/C	G/A
Control-0707	2	60	0	0	1	C/T	C/C	A/A
Control-0708	1	51	1	1	0	C/T	G/C	A/A
Control-0709	2	51	0	0	1	C/C	G/G	A/A
Control-0710	1	46	1	0	1	C/T	C/C	A/A
Control-0711	1	64	1	0	0	C/T	G/C	A/A
Control-0712	1	56	1	1	1	T/T	G/G	A/A
Control-0713	2	46	0	0	0	T/T	C/C	A/A
Control-0714	2	56	0	0	0	T/T	G/C	A/A
Control-0715	1	53	0	0	1	C/T	G/C	A/A
Control-0716	2	66	0	0	1	T/T	C/C	A/A
Control-0717	2	54	0	0	1	C/T	C/C	A/A
Control-0718	1	53	0	0	0	T/T	G/C	A/A
Control-0719	1	65	1	0	1	C/C	G/C	A/A
Control-0720	2	60	0	0	0	C/T	G/C	G/A
Control-0721	2	48	0	0	0	C/T	C/C	A/A
Control-0722	1	52	0	0	0	T/T	G/C	A/A
Control-0723	2	62	0	0	0	T/T	G/C	A/A
Control-0724	2	61	0	0	0	C/T	C/C	G/A
Control-0725	1	56	0	0	1	T/T	C/C	A/A
Control-0726	1	57	0	0	1	T/T	G/C	G/A
Control-0727	1	61	1	1	0	T/T	G/G	A/A
Control-0728	2	65	0	0	0	C/T	C/C	A/A
Control-0729	2	68	0	0	0	C/T	G/G	A/A
Control-0730	1	60	1	0	0	C/T	C/C	A/A
Control-0731	1	65	0	0	1	T/T	G/C	G/A
Control-0732	2	56	0	0	0	C/C	G/C	A/A
Control-0733	1	50	0	1	0	C/C	C/C	A/A
Control-0734	1	49	0	0	0	C/C	G/C	A/A
Control-0735	2	47	0	0	1	C/T	G/C	A/A
Control-0736	2	71	0	0	1	T/T	C/C	G/A
Control-0737	2	64	0	0	0	C/T	G/C	A/A
Control-0738	2	61	0	0	1	C/C	G/G	G/A
Control-0739	2	60	0	0	0	T/T	G/G	A/A
Control-0740	2	66	0	0	1	C/T	C/C	A/A
Control-0741	2	77	0	0	0	T/T	C/C	G/A
Control-0742	2	75	0	0	1	C/T	C/C	A/A
Control-0743	2	43	0	0	1	C/C	G/G	G/A
Control-0744	2	66	0	0	1	C/T	G/G	G/A
Control-0745	1	58	0	0	0	T/T	C/C	A/A
Control-0746	1	58	0	1	1	T/T	G/C	A/A
Control-0747	1	62	0	0	1	C/T	G/G	A/A
Control-0748	2	66	0	0	1	C/C	C/C	G/A
Control-0749	2	61	0	0	0	C/C	G/C	A/A
Control-0750	1	66	1	0	0	C/T	G/C	A/A
Control-0751	2	61	0	0	0	C/T	C/C	G/A
Control-0752	2	70	0	0	1	C/T	C/C	A/A
Control-0753	1	70	0	0	1	C/C	C/C	A/A

Control-0754	1	69	1	1	1	C/T	C/C	A/A
Control-0755	1	60	0	0	0	C/T	G/G	A/A
Control-0756	2	72	0	0	1	C/T	C/C	A/A
Control-0757	1	56	0	0	1	T/T	G/C	G/A
Control-0758	1	66	0	0	0	C/T	C/C	A/A
Control-0759	2	51	0	0	0	C/C	C/C	A/A
Control-0760	1	47	1	1	1	C/T	G/C	A/A
Control-0761	2	72	0	0	0	C/C	G/C	A/A
Control-0762	2	74	0	0	0	C/C	G/C	G/A
Control-0763	2	59	0	0	0	C/C	G/G	A/A
Control-0764	1	69	0	0	1	C/T	G/C	A/A
Control-0765	1	64	0	0	0	T/T	C/C	A/A
Control-0766	2	66	0	0	0	T/T	G/C	A/A
Control-0767	2	72	0	0	1	C/T	C/C	A/A
Control-0768	2	67	0	0	1	T/T	G/C	A/A
Control-0769	2	79	0	0	1	C/T	G/C	G/A
Control-0770	2	59	0	0	0	C/T	G/C	G/A
Control-0771	2	53	0	0	0	C/C	C/C	A/A
Control-0772	1	66	0	0	1	C/T	C/C	G/A
Control-0773	1	50	0	0	1	T/T	C/C	A/A
Control-0774	1	67	0	0	1	C/T	C/C	G/A
Control-0775	1	83	0	0	1	C/T	G/G	A/A
Control-0776	2	50	0	0	0	C/T	G/C	G/A
Control-0777	2	69	0	0	0	C/C	G/C	A/A
Control-0778	1	52	0	0	0	T/T	G/G	G/A
Control-0779	2	69	0	0	0	C/T	C/C	A/A
Control-0780	2	83	0	0	1	C/T	G/G	A/A
Control-0781	2	68	0	0	0	C/C	G/C	A/A
Control-0782	2	61	0	0	0	C/T	G/C	G/A
Control-0783	2	53	0	0	1	C/T	G/C	A/A
Control-0784	2	74	0	0	1	C/T	G/G	A/A
Control-0785	2	67	0	0	1	C/C	G/C	A/A
Control-0786	2	73	0	0	0	T/T	C/C	A/A
Control-0787	2	64	0	0	0	T/T	G/G	A/A
Control-0788	2	61	0	0	1	C/C	G/C	A/A
Control-0789	2	68	0	0	1	C/T	C/C	A/A
Control-0790	2	62	0	0	1	T/T	G/G	G/A
Control-0791	2	75	0	0	1	C/T	C/C	A/A
Control-0792	2	65	0	0	1	C/T	G/C	G/A
Control-0793	2	69	0	0	1	C/T	G/C	A/A
Control-0794	2	48	0	0	0	T/T	C/C	A/A
Control-0795	2	63	0	0	1	C/T	C/C	A/A
Control-0796	2	41	0	0	0	T/T	C/C	A/A
Control-0797	2	49	0	0	0	C/T	C/C	A/A
Control-0798	2	66	0	0	0	C/T	G/G	A/A
Control-0799	2	45	0	0	0	C/T	C/C	G/A
Control-0800	2	45	0	0	1	C/T	G/C	A/A
Control-0801	2	46	0	0	1	C/T	G/C	A/A
Control-0802	2	67	0	0	0	C/T	C/C	G/A
Control-0803	2	65	0	0	0	C/T	G/C	A/A
Control-0804	2	74	0	0	0	C/C	C/C	A/A
Control-0805	2	62	0	0	0	C/T	G/C	A/A
Control-0806	2	55	0	0	0	C/T	G/C	A/A
Control-0807	2	59	0	0	1	C/T	C/C	A/A
Control-0808	2	59	1	1	1	C/T	C/C	A/A
Control-0809	2	57	0	0	1	C/T	C/C	G/A
Control-0810	2	64	0	0	0	C/C	C/C	G/A
Control-0811	2	58	0	0	1	T/T	C/C	A/A
Control-0812	2	64	0	0	1	C/C	C/C	A/A
Control-0813	2	62	0	0	0	C/C	G/C	G/A
Control-0814	2	63	0	0	0	T/T	C/C	A/A
Control-0815	2	56	0	0	1	T/T	G/C	A/A
Control-0816	1	57	0	0	0	T/T	G/C	A/A
Control-0817	1	54	0	0	1	T/T	C/C	A/A
Control-0818	1	52	0	0	0	C/T	G/G	A/A
Control-0819	2	52	0	0	0	T/T	G/G	A/A
Control-0820	1	53	0	0	0	C/C	G/G	A/A
Control-0821	1	59	0	0	0	T/T	G/C	A/A
Control-0822	1	61	0	0	0	T/T	G/C	G/A
Control-0823	2	**66**	0	**0**	0	C/C	C/C	A/A
Control-0824	1	63	0	0	0	C/C	G/C	A/A
Control-0825	1	57	0	0	1	C/T	G/C	G/A
Control-0826	1	67	0	0	0	C/T	C/C	G/A
Control-0827	1	65	0	0	1	C/T	G/C	G/A
Control-0828	1	56	0	0	1	C/T	G/C	G/A
Control-0829	1	67	0	0	0	C/T	G/C	A/A
Control-0830	1	71	1	1	0	C/T	G/C	A/A

Control-0831	1	65	0	0	0	C/C	C/C	A/A
Control-0832	1	55	0	0	0	C/T	G/C	A/A
Control-0833	1	68	0	0	1	C/T	C/C	A/A
Control-0834	2	53	0	0	0	C/C	G/C	A/A
Control-0835	2	65	0	0	0	C/T	G/C	A/A
Control-0836	2	72	0	0	0	C/T	G/C	G/A
Control-0837	1	65	1	0	1	C/C	G/C	A/A
Control-0838	1	65	1	0	0	C/T	C/C	A/A
Control-0839	2	68	0	0	1	C/T	C/C	A/A
Control-0840	1	62	0	0	1	C/T	G/G	A/A
Control-0841	1	52	0	0	1	C/T	C/C	G/A
Control-0842	1	70	1	1	0	T/T	G/G	A/A
Control-0843	2	49	0	0	1	C/T	C/C	A/A
Control-0844	2	56	0	0	0	T/T	G/C	A/A
Control-0845	2	64	0	0	1	C/T	C/C	A/A
Control-0846	2	68	0	0	0	C/T	G/G	A/A
Control-0847	2	53	0	0	0	C/T	C/C	A/A
Control-0848	2	54	0	0	0	C/T	G/C	G/A
Control-0849	2	68	0	0	0	C/C	G/C	G/A
Control-0850	2	49	0	0	1	T/T	C/C	A/A
Control-0851	2	61	0	0	0	C/T	G/C	G/A
Control-0852	2	59	0	0	0	C/T	G/C	A/A
Control-0853	2	57	0	0	0	C/T	C/C	A/A
Control-0854	2	52	0	0	1	C/T	G/C	A/A
Control-0855	2	58	0	0	0	T/T	G/C	G/A
Control-0856	2	64	0	0	0	C/C	C/C	A/A
Control-0857	2	59	0	0	1	C/T	G/G	A/A
Control-0858	2	58	0	0	0	T/T	C/C	A/A
Control-0859	2	62	0	0	0	T/T	G/G	G/A
Control-0860	2	63	0	0	1	C/T	G/C	G/A
Control-0861	2	73	0	0	0	C/T	G/C	G/A
Control-0862	2	59	0	0	0	C/C	G/C	G/A
Control-0863	2	77	0	0	1	C/C	C/C	A/A
Control-0864	2	59	0	0	0	C/T	C/C	G/G
Control-0865	2	59	0	0	0	T/T	G/C	A/A
Control-0866	2	73	0	0	1	C/C	G/G	A/A
Control-0867	2	60	0	0	1	C/C	G/C	A/A
Control-0868	2	73	0	0	0	C/C	G/C	A/A
Control-0869	2	59	0	0	1	T/T	G/C	G/A
Control-0870	2	71	0	0	1	C/T	G/G	A/A
Control-0871	2	59	0	0	1	C/C	C/C	A/A
Control-0872	2	62	0	0	0	C/T	C/C	G/A
Control-0873	2	76	0	0	1	C/T	G/C	A/A
Control-0874	2	56	0	0	0	T/T	C/C	G/A
Control-0875	2	55	0	0	1	C/T	C/C	A/A
Control-0876	2	62	0	0	0	C/T	G/C	G/G
Control-0877	2	62	0	0	1	C/T	C/C	A/A
Control-0878	1	57	0	0	0	C/T	G/C	A/A
Control-0879	1	52	0	0	0	?	?	?
Control-0880	1	54	0	0	1	T/T	C/C	G/G
Control-0881	1	56	0	0	1	T/T	C/C	A/A
Control-0882	1	52	0	0	0	C/T	G/C	A/A
Control-0883	1	50	0	0	0	C/T	C/C	A/A
Control-0884	1	62	0	0	0	C/T	G/C	A/A
Control-0885	1	40	0	0	1	C/T	C/C	A/A
Control-0886	1	49	0	0	1	T/T	G/C	A/A
Control-0887	1	53	0	0	1	C/C	G/C	G/G
Control-0888	1	45	0	0	0	C/T	G/G	G/G
Control-0889	1	48	0	0	0	T/T	G/G	A/A
Control-0890	1	43	0	0	0	C/C	C/C	G/G
Control-0891	1	61	0	0	1	C/C	G/G	A/A
Control-0892	1	51	0	0	1	C/T	G/C	G/G
Control-0893	1	69	0	0	1	C/T	G/C	A/A
Control-0894	1	55	0	0	1	C/T	G/G	A/A
Control-0895	2	50	0	0	1	T/T	G/C	G/A
Control-0896	2	56	0	0	1	C/T	G/G	A/A
Control-0897	1	53	1	1	0	C/T	C/C	A/A
Control-0898	2	55	0	0	0	T/T	G/C	A/A
Control-0899	1	62	0	0	1	C/T	G/C	A/A
Control-0900	1	49	0	0	1	T/T	G/G	A/A
Control-0901	1	63	0	0	0	T/T	C/C	A/A
Control-0902	2	61	0	0	1	T/T	G/C	A/A
Control-0903	1	51	0	0	1	T/T	G/C	A/A
Control-0904	1	47	0	0	0	C/T	G/G	A/A
Control-0905	1	49	0	0	0	T/T	G/C	G/A
Control-0906	2	62	0	0	1	C/T	G/G	A/A
Control-0907	2	68	0	0	1	C/T	C/C	A/A

Control-0908	1	40	0	0	0	C/T	C/C	A/A
Control-0909	1	59	0	0	0	T/T	G/C	G/A
Control-0910	1	49	0	0	0	C/C	C/C	G/A
Control-0911	1	63	0	0	0	C/C	C/C	G/A
Control-0912	1	49	0	0	1	C/T	C/C	G/A
Control-0913	2	67	0	0	1	C/T	C/C	G/A
Control-0914	1	66	0	0	0	C/T	G/C	G/A
Control-0915	1	42	1	1	0	T/T	G/G	A/A
Control-0916	1	41	0	0	0	T/T	G/G	A/A
Control-0917	2	56	0	0	1	T/T	G/G	G/A
Control-0918	1	60	1	1	0	C/T	G/C	G/A
Control-0919	1	65	1	0	0	C/C	C/C	A/A
Control-0920	1	55	0	0	0	T/T	C/C	A/A
Control-0921	1	63	1	1	0	T/T	C/C	A/A
Control-0922	2	40	0	0	0	C/T	C/C	A/A
Control-0923	1	50	0	1	0	C/C	C/C	A/A
Control-0924	1	50	1	0	0	C/T	G/C	A/A
Control-0925	2	53	0	0	1	T/T	G/C	G/A
Control-0926	2	55	0	0	0	C/T	G/C	A/A
Control-0927	2	57	0	0	1	T/T	C/C	A/A
Control-0928	2	56	0	0	0	C/T	C/C	A/A
Control-0929	2	60	0	0	1	C/T	C/C	G/A
Control-0930	2	51	0	0	1	C/T	G/G	A/A
Control-0931	2	61	0	0	1	C/C	C/C	A/A
Control-0932	2	52	0	0	0	C/T	G/C	G/A
Control-0933	2	56	0	0	0	T/T	G/C	A/A
Control-0934	2	60	0	0	0	T/T	G/C	G/G
Control-0935	2	58	0	0	0	C/T	C/C	A/A
Control-0936	2	59	0	0	0	C/T	C/C	A/A
Control-0937	2	60	0	0	1	C/T	G/C	A/A
Control-0938	2	53	0	0	1	T/T	C/C	A/A
Control-0939	2	56	0	0	0	T/T	G/C	A/A
Control-0940	2	61	0	0	1	C/C	G/C	A/A
Control-0941	2	59	0	0	0	C/T	G/C	G/A
Control-0942	2	57	0	0	0	T/T	C/C	A/A
Control-0943	2	60	0	0	0	C/T	C/C	A/A
Control-0944	2	32	0	0	0	T/T	C/C	G/A
Control-0945	2	50	0	0	1	C/T	C/C	A/A
Control-0946	2	56	0	0	0	C/T	G/G	G/A
Control-0947	2	60	0	0	1	C/C	G/C	A/A
Control-0948	2	58	0	0	1	T/T	C/C	A/A
Control-0949	2	50	0	0	1	C/C	C/C	A/A
Control-0950	2	52	0	0	1	T/T	G/C	G/A
Control-0951	1	69	0	0	0	C/T	C/C	A/A
Control-0952	2	79	0	0	1	C/T	G/C	G/A
Control-0953	1	63	0	0	1	C/T	G/G	A/A
Control-0954	1	61	0	0	0	C/C	C/C	G/A
Control-0955	1	55	0	0	1	C/T	G/C	A/A
Control-0956	1	77	0	0	0	C/C	C/C	A/A
Control-0957	1	63	0	0	0	T/T	G/G	G/G
Control-0958	2	61	0	0	1	C/T	G/C	A/A
Control-0959	2	69	0	0	0	C/T	C/C	A/A
Control-0960	2	47	0	0	0	C/T	C/C	A/A
Control-0961	1	46	0	0	0	T/T	C/C	G/A
Control-0962	1	65	1	1	0	C/T	G/C	G/A
Control-0963	1	50	0	0	1	C/C	G/C	G/A
Control-0964	2	76	0	0	1	T/T	G/C	A/A
Control-0965	2	53	0	0	1	C/T	G/C	A/A
Control-0966	1	65	0	0	1	C/T	G/C	A/A
Control-0967	1	38	0	0	1	C/C	C/C	A/A
Control-0968	2	46	0	0	0	C/T	G/G	A/A
Control-0969	1	32	1	1	0	C/C	C/C	G/A
Control-0970	2	69	0	0	0	C/T	C/C	G/A
Control-0971	1	74	0	0	0	C/T	G/G	A/A
Control-0972	1	58	1	0	1	C/T	C/C	A/A
Control-0973	2	45	0	0	0	C/T	G/C	A/A
Control-0974	1	61	1	0	1	T/T	G/C	A/A
Control-0975	1	34	0	0	0	C/C	C/C	A/A
Control-0976	1	68	0	0	0	C/T	G/C	A/A
Control-0977	2	47	0	0	1	C/C	G/C	G/A
Control-0978	2	30	0	0	0	C/T	C/C	A/A
Control-0979	1	57	0	1	0	C/T	G/C	A/A
Control-0980	2	46	0	0	1	T/T	C/C	A/A
Control-0981	2	58	0	0	0	C/T	C/C	A/A
Control-0982	1	44	1	1	1	T/T	G/G	G/A
Control-0983	1	58	0	1	1	?	?	?
Control-0984	1	61	1	0	0	C/T	C/C	G/A

Control-0985	1	57	0	0	0	T/T	G/C	G/A
Control-0986	2	63	0	0	1	C/T	G/C	G/A
Control-0987	2	49	1	1	0	T/T	G/C	A/A
Control-0988	2	64	0	0	0	C/T	C/C	A/A
Control-0989	2	36	0	0	0	C/T	C/C	A/A
Control-0990	1	59	0	0	0	C/T	G/G	A/A
Control-0991	2	44	0	0	0	C/T	G/C	G/A
Control-0992	2	62	0	0	0	C/C	C/C	A/A
Control-0993	2	73	0	0	1	C/T	G/C	G/A
Control-0994	1	60	1	0	0	T/T	C/C	A/A
Control-0995	2	62	0	0	0	C/T	G/C	G/A
Control-0996	1	51	1	0	1	C/T	C/C	A/A
Control-0997	1	73	0	0	1	C/T	C/C	G/A
Control-0998	1	75	0	0	0	C/C	C/C	A/A
Control-0999	2	47	0	0	1	C/C	G/C	G/A
Control-1000	1	44	0	0	0	C/C	C/C	A/A
Control-1001	1	79	0	0	0	T/T	C/C	G/A
Control-1002	2	65	0	0	1	C/T	C/C	A/A
Control-1003	1	68	0	0	0	C/T	C/C	A/A
Control-1004	2	43	0	0	0	C/C	G/C	A/A
Control-1005	1	79	0	0	1	C/T	G/C	A/A
Control-1006	2	51	0	0	0	T/T	G/C	A/A
Control-1007	1	39	0	0	1	C/T	C/C	A/A
Control-1008	2	68	0	0	0	T/T	G/C	A/A
Control-1009	2	75	0	0	0	?	?	?
Control-1010	2	63	0	0	0	T/T	G/C	G/A
Control-1011	2	44	0	0	1	C/T	G/C	A/A
Control-1012	1	60	0	0	0	T/T	C/C	A/A
Control-1013	1	86	0	0	0	C/T	G/C	A/A
Control-1014	1	47	0	0	0	C/C	C/C	A/A
Control-1015	2	41	0	1	1	T/T	G/C	A/A
Control-1016	2	79	0	0	0	C/T	G/G	A/A
Control-1017	1	67	1	1	0	C/T	C/C	A/A
Control-1018	1	46	0	0	0	C/C	G/C	A/A
Control-1019	2	56	0	0	1	C/T	G/C	G/A
Control-1020	2	74	0	0	0	C/C	C/C	G/G
Control-1021	1	76	0	0	0	T/T	C/C	G/A
Control-1022	1	44	1	0	0	C/T	G/C	G/A
Control-1023	1	43	0	0	1	T/T	C/C	G/A
Control-1024	1	74	1	1	1	C/C	G/C	G/A
Control-1025	2	68	0	0	0	C/C	G/C	A/A
Control-1026	1	82	0	0	0	T/T	C/C	G/A
Control-1027	2	78	0	0	1	C/C	C/C	A/A
Control-1028	1	64	1	0	0	C/C	C/C	A/A
Control-1029	1	36	1	1	0	C/C	G/G	A/A
Control-1030	1	35	0	0	1	C/T	C/C	A/A
Control-1031	1	63	0	0	0	T/T	G/C	G/A
Control-1032	2	74	0	0	0	C/T	G/C	A/A
Control-1033	2	63	0	0	0	C/T	G/C	A/A
Control-1034	2	70	0	0	1	C/C	C/C	A/A
Control-1035	1	63	0	0	0	C/T	G/C	A/A
Control-1036	1	65	0	0	0	C/T	G/C	A/A
Control-1037	1	80	0	0	1	T/T	G/C	G/A
Control-1038	1	74	1	1	0	C/T	C/C	G/A
Control-1039	1	55	0	0	1	C/C	G/C	A/A
Control-1040	1	24	0	0	0	C/T	G/G	A/A
Control-1041	2	71	0	0	0	C/T	G/C	A/A
Control-1042	1	58	0	0	1	C/T	G/C	G/A
Control-1043	1	53	1	1	0	C/T	G/C	G/G
Control-1044	2	60	0	0	0	C/T	G/C	A/A
Control-1045	1	78	0	0	1	C/C	C/C	A/A
Control-1046	1	69	1	1	0	T/T	G/C	A/A
Control-1047	1	53	0	0	1	T/T	G/G	A/A
Control-1048	1	49	0	0	0	T/T	C/C	A/A
Control-1049	2	78	0	0	1	C/T	G/G	A/A
Control-1050	1	38	0	0	0	C/T	C/C	G/A
Control-1051	1	47	1	1	0	C/T	G/C	A/A
Control-1052	1	77	0	0	0	C/T	G/G	A/A
Control-1053	2	62	0	0	0	C/T	C/C	A/A
Control-1054	2	67	0	0	0	T/T	G/G	A/A
Control-1055	1	54	1	0	1	C/C	C/C	A/A
Control-1056	1	64	0	0	1	C/C	G/G	G/G

?: not available
